# Effect of sample stratification on dairy GWAS results

**DOI:** 10.1186/1471-2164-13-536

**Published:** 2012-10-06

**Authors:** Li Ma, George R Wiggans, Shengwen Wang, Tad S Sonstegard, Jing Yang, Brian A Crooker, John B Cole, Curtis P Van Tassell, Thomas J Lawlor, Yang Da

**Affiliations:** 1 Department of Animal Science, University of Minnesota, St. Paul, Minnesota, USA; 2 Animal Improvement Programs Laboratory, Agricultural Research Service, USDA, Beltsville, Maryland, USA; 3 Bovine Functional Genomics Laboratory, Agricultural Research Service, USDA, Beltsville, Maryland, USA; 4 Holstein Association USA, Brattleboro, Vermont, USA; 5 Department of Biological Statistics and Computational Biology, Cornell University, Ithaca, New York, USA

## Abstract

**Background:**

Artificial insemination and genetic selection are major factors contributing to population stratification in dairy cattle. In this study, we analyzed the effect of sample stratification and the effect of stratification correction on results of a dairy genome-wide association study (GWAS). Three methods for stratification correction were used: the efficient mixed-model association expedited (EMMAX) method accounting for correlation among all individuals, a generalized least squares (GLS) method based on half-sib intraclass correlation, and a principal component analysis (PCA) approach.

**Results:**

Historical pedigree data revealed that the 1,654 contemporary cows in the GWAS were all related when traced through approximately 10–15 generations of ancestors. Genome and phenotype stratifications had a striking overlap with the half-sib structure. A large elite half-sib family of cows contributed to the detection of favorable alleles that had low frequencies in the general population and high frequencies in the elite cows and contributed to the detection of X chromosome effects. All three methods for stratification correction reduced the number of significant effects. EMMAX method had the most severe reduction in the number of significant effects, and the PCA method using 20 principal components and GLS had similar significance levels. Removal of the elite cows from the analysis without using stratification correction removed many effects that were also removed by the three methods for stratification correction, indicating that stratification correction could have removed some true effects due to the elite cows. SNP effects with good consensus between different methods and effect size distributions from USDA’s Holstein genomic evaluation included the *DGAT1-NIBP* region of BTA14 for production traits, a SNP 45kb upstream from *PIGY* on BTA6 and two SNPs in *NIBP* on BTA14 for protein percentage. However, most of these consensus effects had similar frequencies in the elite and average cows.

**Conclusions:**

Genetic selection and extensive use of artificial insemination contributed to overlapped genome, pedigree and phenotype stratifications. The presence of an elite cluster of cows was related to the detection of rare favorable alleles that had high frequencies in the elite cluster and low frequencies in the remaining cows. Methods for stratification correction could have removed some true effects associated with genetic selection.

## Background

Genome-wide association study (GWAS) is a powerful tool for identifying genetic factors associated with phenotypes. Population stratification refers to systematic differences in allele frequencies between subpopulations and is a source for false positive results in GWAS [1-4]. In human populations, geographical separation followed by genetic drift is the basic cause of population stratification. In dairy cattle, population stratification could be a result of several causes, genetic selection and hitchhiking, artificial selection, and genetic sampling or drift. In U.S. dairy cattle breeding, artificial insemination has been widely used and this has increased the likelihood of the presence of related individuals in randomly selected samples and the presence of large half-sib families; both of which contribute to population stratification. Many years of genetic selection in dairy cattle has caused substantial allele frequency changes [5] that also contribute to population stratification. Methods proposed to address population or sample stratification in GWAS include the genomic control approach [1], principal component analysis (PCA) [2], and a mixed model approach [3,4]. Our dairy GWAS report identified highly significant SNP effects with minor favorable allele frequencies and a large number of X chromosome effects [6]. This article is a follow-up study of our GWAS report to analyze potential sample stratification of the study population, the relationship between the significant effects and sample stratification, and the effect of sample stratification correction on our reported GWAS results. Effect of stratification correction was evaluated with three methods: the efficient mixed-model association expedited (EMMAX) method which accounted for correlation among all individuals [3], a generalized least squares (GLS) method based on half-sib intraclass correlation [7,8], and a PCA method [2] that used the top 20 principal components as covariables.

## Results

### Overlap between genome, pedigree and phenotype stratifications

The genome stratification based on 45,878 SNP markers was shown using the multidimensional scaling (MDS) method [9] for two sets of data: the association data set of 1,654 contemporary Holstein cows used in the GWAS by Cole et al. [6], and the selection signature data set [5] that included the 1,654 contemporary cows and historical U.S. Holstein cattle for a total of 2,366 cows and bulls. The majority of the historical cattle (301 cows and bulls) were the University of Minnesota Holstein control line that remained unselected since 1964 [5].

**Figure 1:**
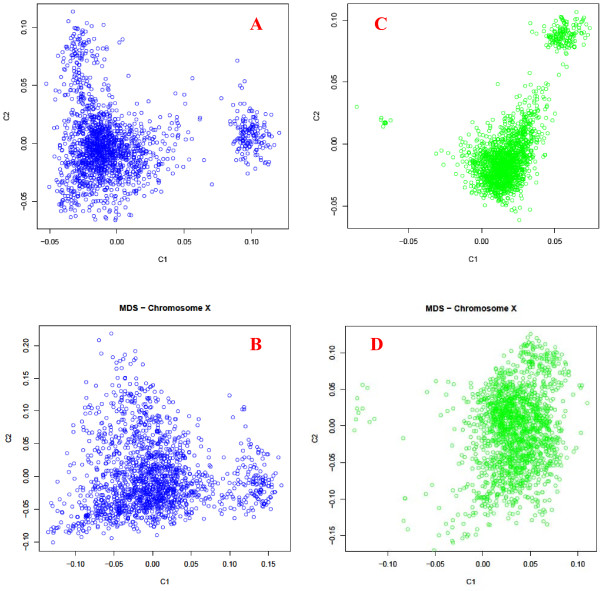
**Multidimensional scaling (MDS) plots of SNP genotypes of 1,654 contemporary Holstein cows.A**) All 45,878 SNP markers: C1 and C2 values were calculated using 1,654 contemporary Holstein cows. **B**) X chromosome: C1 and C2 values were calculated using 1,654 contemporary Holstein cows. **C**) All 45,878 SNP markers: C1 and C2 values were calculated using 2,366 Holstein cattle, including the University of Minnesota Holstein control line that remained unselected since 1964. **D**) X chromosome: C1 and C2 values were calculated using 2,366 Holstein cattle, including the University of Minnesota Holstein control line that remained unselected since 1964. C1 = dimension 1, C2 = dimension 2. Graphs for individual chromosomes are given in Additional file 1: Figure S1.

The association data of 1,654 cows showed some genome stratification, with a large cluster on the left and a small cluster on the right of Figure 1A. The X chromosome is the only individual chromosome with a similar stratification pattern (Figure 1B) as the stratification for all chromosomes (Figure 1A), and no individual autosome had such a similar pattern (Additional file 1: Figure S1).

The selection signature data set of 2,366 contemporary and historical cattle showed more stratification with three clusters (Figure 1C). The third cluster on the far left of Figure 1C had only eight cows, which were not separated as a cluster by Figure 1A. The X chromosome stratification pattern in Figure 1D did not resemble the stratification pattern of all chromosomes shown in Figure 1C but showed more stratification than any individual autosomes (Additional file 1: Figure S1).

Despite the fact that the cow DNA samples in our GWAS were from diverse academic and industry donors, analysis of USDA’s historical Holstein pedigree data revealed that the 1,654 cows were all related when traced through approximately 10 to 15 generations (back to the 1930’s) of ancestors (Additional file 2: Figure S2). Of the 1,654 cows, 1,600 cows were related to a bull born in 1947, and 1382 cows were related to another bull also born in 1947. These two bulls had 1360 common descendents among the 1,654 cows, and only 32 of the 1,654 cows were not descendents of these two bulls.

**Figure 2:**
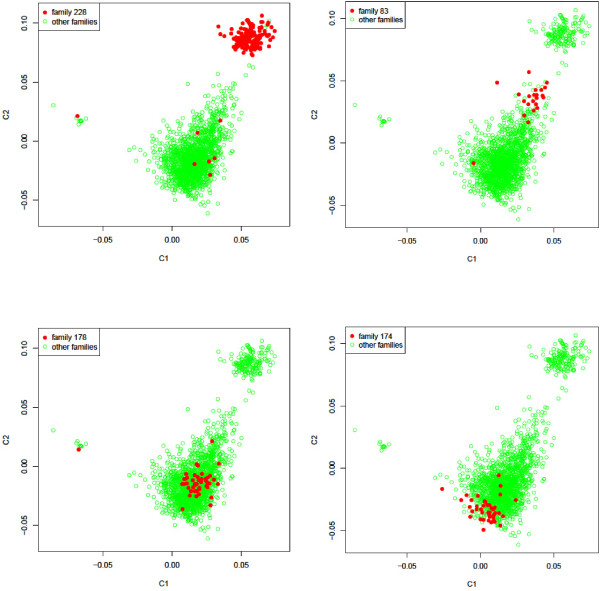
**Examples of overlap between genome stratification and half-sib family structure.** C1 = dimension 1, C2 = dimension 2. C1 and C2 values were calculated using 2,366 Holstein cattle, including the University of Minnesota Holstein control line that remained unselected since 1964. Graphs for more selected families are given in Additional file 3: Figure S3.

Although all 1,654 cows were related, the genome stratification patterns shown in Figure 1 had a striking overlap with the half-sib pedigree structure from 355 sires (see Figure 2 for four examples of sire families and Additional file 3: Figure S3 for 18 sire families). The number of cows per half-sib family (per sire) ranged from 1 to 160. The upper-right cluster of Figure 1C had 160 cows, of which 153 cows were half-sibs from one sire (family 228 in Figure 2) that had 160 daughters among the 1,654 cows. The other seven daughters in family 228 were classified into the left two clusters of Figure 1C (family 228 in Figure 2). The genome from this sire, including autosomes and the X chromosome, is responsible for the stratification of the upper-right cluster of Figure 1C (or the right cluster of Figure 1A). The 153 half-sib cows should carry the same sire X chromosome (except the pseudo-autosomal region), and this should be the reason for the strong X stratification pattern shown in Figure 1B. In contrast, the 153 half-sib cows should have different sire autosomes due to recombination between each pair of sire autosomes. Collectively, the 29 autosomes had nearly identical stratification pattern as in Figure 1A (data not shown).

**Figure 3:**
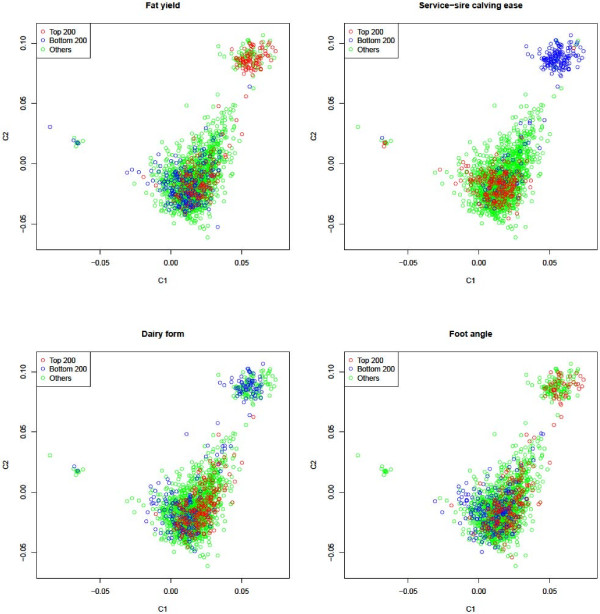
**Examples of overlap between genome and phenotype stratifications.** C1 = dimension 1, C2 = dimension 2. C1 and C2 values were calculated using 2,366 Holstein cattle, including the University of Minnesota Holstein control line that remained unselected since 1964. Graphs for all 31 traits are given in Additional file 4: Figure S4.

The genome and pedigree stratifications had various degrees of overlap with the phenotypic stratification for many of the 31 traits (see Figure 3 for the examples of four traits and Additional file 4: Figure S4 for all 31 traits). Fat, protein and milk yields had the strongest overlap between genome and phenotype stratifications. The genome stratification pattern using the data set of 2,366 contemporary and historical cattle shown in Figure 1C had a stronger overlap with the phenotype stratification than the genome stratification pattern using the 1,654 contemporary cows shown in Figure 1A. Dimension I (C1) of Figure 1C is strongly related to milk, fat and protein yields, with low-producing cows on the left and high-producing cows on the right. Fitting C1 calculated from the 2,366 historical and contemporary cattle in the statistical model virtually removed all significant effects for milk, fat and protein yields detected by the same method without C1 in the model, whereas the C1 values calculated from the 1,654 contemporary cows essentially had no effect on SNP effects (data not shown).

**Figure 4:**
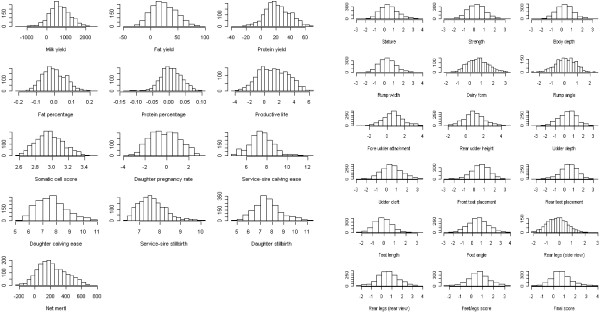
Phenotypic distributions of PTA values of 31 dairy traits in 1,654 contemporary Holstein cows.

The 160 cows that comprised the small cluster in the upper right of Figure 1C (or the small cluster on the right of Figure 1A) were primarily from one sire family (family 228 in Figure 2A) of elite cows. This cluster had a high frequency of cows with high PTA values for fat, protein and milk yields, fat and protein percentages, productive life, daughter pregnancy rate, net merit, strength, stature, body depth, rump angle, fore udder attachment, udder depth, foot angle, rear legs (rear view), feet-legs score, final score, and had low PTA values for somatic cell score, service-sire calving ease, daughter calving ease, service-sire stillbirth, dairy form, udder cleft, teat length, front teat placement, and rear legs (side view) (Additional file 4: Figure S4). This cluster had the most obvious overlap between the genome and phenotype stratifications for many of the 31 traits. The presence of the 160 elite cows in this small cluster and the presence of the 153 elite cows from one sire did not result in deviations from normal phenotypic distributions (Figure 4).

The X chromosome’s genetic stratification of the 1,654 contemporary cows (Figures 1B and 1D) had a similar overlap with phenotypic stratification as all chromosomes discussed above. In contrast, individual autosomes did not show such a strong overlap (a comparison between chromosome 1 and the X chromosome is given in Additional file 5: Figure S5). The overlap between the X chromosome stratification and the phenotypic stratification could be a major reason why many X chromosome effects were detected in Cole et al. [6]. This overlap should be an opportunity for detecting potential X chromosome SNP effects associated with the elite cows, although it is unclear whether this overlap contributed to detecting true or spurious X chromosome SNP effects.

Stratification analysis showed that the genome, pedigree and phenotype stratifications of the 1,654 contemporary Holstein cows used in the GWAS [6] overlapped. The implication of this overlap is that the removal of population stratification effects could remove some true SNP effects confounded with the population stratification that likely was a result of genetic selection, drift, and genetic hitchhiking of selection.

### Association results from three methods for stratification correction

The three methods for stratification correction included a mixed model method implemented by EMMAX [3] using IBS (EMMAX-IBS; IBS = identify by state) or the Balding-Nichols (BN) kinship matrix [10] (EMMAX-BN) among all 1,654 cows, a GLS method using intraclass correlation among half sibs [7], and a PCA approach [2] using the top 20 principal components as covariables. The EMMAX method (including EMMAX-IBS and EMMAX-BN) was most severe in removing stratification effects or in reducing the number of significant effects, the PCA method using 20 components and GLS method had similar significance levels for most traits. For 31 dairy traits, EMMAX-IBS had 15, and EMMAX-BN had six SNP effects reaching genome-wide significance, in addition to a cluster of SNP effects for fat percentage in and near the *DGAT1-NIBP* region of BTA14 identified by all three methods. The 15 significant effects of EMMAX-IBS (other than those for fat percentage) included two effects for fat yield, two effects for protein percentage, three effects for service-sire calving ease, one effect for daughter calving ease, three effects for service-sire stillbirth, one effect for front teat placement, one effect for teat length, one effect for rear legs (rear view), and two effects for foot angle. GLS and PCA had more effects reaching genome-wide significance, generally dozens more per trait than EMMAX. A global view of all SNP effects for each trait from the three methods is presented in Manhattan plots [11,12] in Additional file 6: Figure S6. The top 100 effects of each trait from EMMAX-IBS and EMMAX-BN are given in Additional file 7: Table S1, the top 100 effects of each trait from GLS are given in Additional file 8: Table S2, and the top 100 effects of each trait from PCA are given in Additional file 9: Table S3.

#### Effects from EMMAX, GLS, and PCA methods with genome-wide significance

The three methods had a small number of common effects reaching genome-wide significance. The common effects included a cluster of SNP effects for fat percentage in and near the *DGAT1-NIBP* region of BTA14, a SNP in *DGAT1* and a SNP in *A5D786* on BTA14 for fat yield (ranked #1 and #2 by all three methods), and a SNP 45kb upstream from *PIGY* on BTA6 with a 97% frequency of the favorable allele for protein percentage (#1 by EMMAX, GLS and PCA), and a SNP in *NIBP* on BTA14 for protein percentage (ranked #1 by EMMAX and PCA, and #16 by GLS).

**Figure 5:**
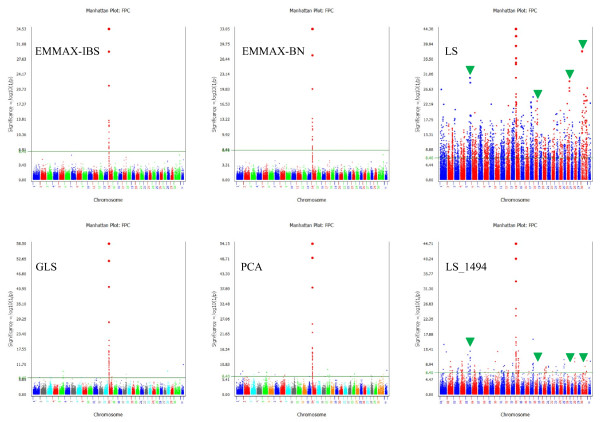
**Global view of test results from four method for fat percentage.** EMMAX-IBS is the EMMAX method using correlation measured by identity by state (IBS) among all individuals. EMMAX-BN is the EMMAX using the Balding-Nichols kinship matrix among all individuals. GLS is the generalized least squares method accounting for half-sib intraclass correlation. PCA is the method of principal component analysis for stratification correction using to top 20 principal components as covariables. LS is the least squares method without stratification correction reported in Cole et al. [6]. LS_1494 is the LS analysis without the 160 elite cows.

All three methods identified the *DGAT1-NIBP* region as the most significant region for fat percentage and eliminated most significant effects of other regions detected by the least squares (LS) method without stratification correction (Figure 5; Additional file 6: Figure S6), and identified a SNP in *DGAT1* as the most significant and two *NIBP* markers among the top 15 effects for fat percentage (Additional file 7, Additional file 8, Additional file 9: Tables S1, S2, S3). EMMAX (EMMAX-IBS and EMMAX-BN) identified the *DGAT1-NIBP* region as the only significant region for fat percentage, while GLS and PCA also had SNP effects on other chromosomes that reached genome-wide significance but were far less significant than the *DGAT1-NIBP* region (Figure 5). All three methods for stratification correction identified *DGAT1* as the most significant for fat yield, and GLS also identified the *A5D786-CYHR1*-*VPS28-DGAT1* region as the most significant for milk yield.

The three methods for stratification correction had no overlapping effects with genome-wide significance for protein yield by any two of the three methods, but EMMAX and GLS identified a 0.5 Mb region upstream of *PLCB1* as highly significant for protein yield (#1 by EMMAX and #3 by GLS at 739,720 bp), with the EMMAX effect below the genome-wide significance level. EMMAX-IBS had three effects for service-sire calving ease and EMMAX-BN had a SNP in *MBTPS2* of BTAX for fat yield reaching genome-wide significance, but none of these was among the top 100 effects by the other methods. For body conformation traits, EMMAX-IBS had five SNP effects with genome-wide significance for front teat placement (#78 from GLS and #75 from PCA), teat length (#8 from GLS), foot angle (#24 from GLS), and rear legs (rear view). EMMAX-BN generally had similar results as EMMAX-IBS with slightly lower significance (higher p-values).

#### Common effects among top 100 effects/trait from methods with and without stratification correction

The two EMMAX models had only a small number of SNP effects reaching genome-wide significance so that significant results of this method had minimal overlap with results from the other methods. Since the exact threshold p-value that should be used for declaring significance for various methods under various conditions is unknown, the top 100 effects of each trait from the three methods with stratification correction (EMMAX-IBS, GLS and PCA) were compared with those from the LS method without stratification correction [6] (Table 1, Additional file 10: Table S4). EMMAX-IBS had 270 SNP effects, GLS had 364 and PCA had 95 effects overlapping with results from the LS method without stratification correction [6] (Table 1). The four methods with and without stratification correction had limited common effects among the top 100 effects per trait, a total of 41 common effects for 13 of the 31 traits (Table 1).

**Table 1:** Number of top 100 SNP effects from the least squares analysis without stratification correction that overlapped with the top 100 effects from EMMAX-IBS (E), GLS (G), and PCA (P) methods for stratification correction

**Traits**	**E**	**G**	**P**	**EG**	**EP**	**GP**	**EGP**
MY	7	15	6	2	2	4	1
FY	4	2	0	1	0	0	0
PY	1	5	0	0	0	0	0
FPC	28	23	22	23	22	22	22
PPC	8	3	3	1	1	3	1
PL	6	10	1	3	0	1	0
SCS	6	7	0	0	0	0	0
DPR	7	8	2	3	2	2	2
SCE	8	3	2	0	2	0	0
DCE	3	3	0	1	0	0	0
SSB	8	6	6	2	6	2	2
DSB	6	11	2	0	0	0	0
NM	4	1	0	0	0	0	0
Total	96	97	44	36	35	34	28
STA	11	24	1	5	1	0	0
STR	5	12	0	1	0	0	0
BD	7	14	0	2	0	0	0
RW	12	31	2	6	1	1	1
DF	8	7	1	1	1	1	1
RA	19	22	6	10	4	6	4
FUA	6	13	1	1	1	0	0
RUH	9	13	4	2	2	0	0
UD	5	14	0	3	0	0	0
UC	13	10	3	2	3	0	0
FTP	17	16	5	6	4	2	2
RTP	12	13	3	2	3	0	0
TL	16	8	5	3	5	1	1
FA	6	15	4	4	1	2	1
RLS	2	2	6	2	1	1	1
RLR	5	18	3	4	0	1	0
FL	11	14	3	6	2	2	2
FS	10	21	4	0	2	0	0
Total	174	267	51	60	31	17	13

E, EMMAX-IBS; G, GLS; P, PCA; MY, milk yield; FY, fat yield; PY, protein yield; FPC, fat percentage; PPC, protein percentage; PL, productive life; SCS, somatic cell score; DPR, daughter pregnancy rate; SCE, service-sire calving ease; DCE, daughter calving ease; SSB, service-sire stillbirth; DSB, daughter stillbirth; NM, net merit; STA, stature; STR, strength; BD, body depth; DF, dairy form; RA, rump angle; RW, rump width; FUA, fore udder attachment; RUH, rear udder height; UD, udder depth; UC, udder cleft; FTP, front teat placement; RTP, rear teat placement; TL, teat length; FA, foot angle; RLS, rear legs (side view); RLR, rear legs (rear view); FL, feet/legs score; FS, final score.

For milk yield, Hapmap42685-BTA-81134 of BTA8 about 87 kb downstream of *ANXA1* was ranked #1 by PCA, #4 by EMMAX-BN, #5 by EMMAX-IBS, #12 by GLS, and #19 by LS. The favorable allele frequency was 0.92 in the elite cluster of 160 cows (to be referred to as ‘elite cluster’, the upper-right cluster of Figure 1C) and was 0.83 in the remaining 1,494 cows in the left two clusters of Figure 1C (to be referred to as ‘average cluster’). For protein percentage, ARS-BFGL-NGS-56327 in *NIBP* was #2 by EMMAX-IBS and EMMAX-BN, #3 by GLS, #7 by PCA, and #19 by LS. The favorable allele frequency was 0.65 in the elite cluster and 0.29 in the average cluster. For daughter pregnancy rate, the four methods had two common SNP effects on the X chromosome. Hapmap55242-rs29011414 was #1 by EMMAX-IBS, #2 by EMMAX-BN and GLS, #31 by LS and #37 by PCA, and ARS-BFGL-NGS-94205 in *GRIA3* was #2 by EMMAX-IBS, #11 by EMMAX-BN, #7 by LS, #11 by GLS, and #40 by PCA. The favorable allele frequency of Hapmap55242-rs29011414 was 0.88 in the elite cluster and 0.74 in the average cluster, and the favorable allele frequency of ARS-BFGL-NGS-94205 was 0.71 in the elite cluster and 0.44 in the average cluster. For service-sire stillbirth, the four methods had two common SNP effects on the X chromosome: Hapmap28373-BTA-160078 was #2 by EMMAX-IBS and PCA, #4 by LS, and #87 by GLS, and ARS-BFGL-NGS-61325 was #3 by EMMAX-IBS, #4 by PCA, #6 by LS and #91 by GLS. The favorable allele frequency was 0.58 in the elite cluster and 0.18 in the average cluster for both markers. For body conformation traits, eight traits had overlapping effects among the four methods with and without stratification correction (Table 1). Allele frequency differences between the elite and average clusters generally were not as large as those discussed above (Additional file 10: Table S4).

### Comparison with effect size distribution from USDA genomic prediction

The USDA genomic prediction uses genomic correlations among all individuals [13] and routinely publishes the SNP effect size distributions at the website of Animal Improvement Programs Laboratory (AIPL) [14,15]. Effect size distributions from the April 2012 Holstein genomic evaluation based on 18,181 bulls and 21,118 cows were compared with the effect significance in this study for consensus SNP effects of the 31 dairy traits. A relatively small number of large effects (AIPL effects) involving an even smaller number of genes were observed from the USDA genomic evaluation. The three gene regions that accounted for most of the large AIPL effects included *DGAT1* of BTA14, *SIGLEC12* of BTA18 and the *MIR584-8~KAL1* pseudo-autosomal region of BTAX. Of these three gene regions, *DGAT1* and *SIGLEC12* were reported in an AIPL study [14]. The five methods for SNP testing (LS, EMMAX-IBS, EMMAX-BN, GLS and PCA) had confirmation with the AIPL effects for 17 of the 31 traits (Table 2, Additional file 11: Figure S7), and the LS method had two BTA18 markers that were the most significant for three traits (daughter pregnancy rate, daughter calving ease and net merit) with the largest AIPL effects at *SIGLEC12*. All five methods confirmed the large effects for fat percentage in the first 4.5 Mb region of BTA14. EMMAX, GLS and PCA confirmed the largest effect for fat yield in the *DGAT1* region and the largest effect for protein percentage at a SNP 45kb upstream from *PIGY* on BTA6. Other confirmations included those by GLS and EMMAX for milk yield at *DGAT1*; LS and GLS for somatic cell score in the *GC-NPFFR2* region of BTA6, EMMAX and PCA for productive life, service-sire calving ease, body depth and front teat placement at *SIGLEC12*; LS for stature, fore udder attachment, rear udder height and final score in the *MIR584-8~KAL1* region of BTAX; EMMAX for rump width at *SIGLEC12* and dairy form in the *MIR584-8~KAL1* region of BTAX, and PCA for service-sire stillbirth at *SIGLEC12*. GLS had the best confirmation of the AIPL effects for milk yield (Table 2) and had the strongest milk effects with seven markers among the top 10 effects for milk yield in the *A5D786-GML* region of BTA14 that had 13 genes including *DGAT1.* GLS had significant SNP effects for protein yield upstream of *DGAT1* in *VPS28* (#4) and *CYHR1* (#6) but did not confirm *DGAT1* which had the largest AIPL effect for protein yield and was located next to *VPS28*. LS confirmed a large AIPL effect at 93.2 Mb of BTA5 for fat percentage. LS had highly significant effects at 53.95 Mb and 58.7 Mb for productive life, net merit, service-sire calving ease and daughter calving ease, and at 58.7 Mb for daughter pregnancy rate. For these traits, the AIPL effects and the EMMAX and PCA effects for service-sire calving ease and the PCA effect for service-sire stillbirth were at *SIGLEC12* (Table 2, Additional file 11: Figure S7).

**Table 2:** Consensus between the top 20 AIPL effects and the top 20 significant effects of the four methods of SNP testing, LS, EMMAX-IBS, GLS, and PCA

**Traits**	**Range of top 20 AIPL effects**	**LS**^**1**^	**EMMAX -IBS**^**1**^	**GLS**^**1**^	**PCA**^**1**^	**Gene region**
MY	9.1540 – 10.3909	-	1-16	2-1, 1–2,	-	BTA14: *A5D786-CYHR1*-
				4-4, 7–5,		*VPS28-DGAT1,*
				3,6~8^2^		*A5D786-GML*^2^
FY	0.3717 – 3.1325	-	1-1	1-1	1-1	BTA14: *DGAT1*
PY	0.2409 – 0.6798	5-1	5-5	3-4,6	-	BTA18: *LOC100139758*
						53.95 Mb
						BTA14: *DGAT1*
						BTA14:*A5D786,VPS28*
FPC	0.0022 – 0.0191	1-1, 11-9	1-1	1-1	1-1	BTA14: *DGAT1*
						BTA5: 93.2 Mb
PPC	9.00E-04 – 0.0047	-	1-1	1-1	1-1	BTA6: *HERC3-PIGY*
PL	0.0240 – 0.1608	2-16,2	1-2	-	1-1	BTA18: *SIGLEC12*
						53.95 Mb and*LOC787057*
SCS	0.0026 – 0.0139	1-10	-	1-16	-	BTA6: *GC-NPFFR2*
DPR	0.0175 – 0.0517	3-6	-	-	-	BTA18: *SIGLEC12*, *LOC787057*
SCE	0.0231 – 0.0139	1-1,4	1-4	-	3-4	BTA18:*SIGLEC12*,
						53.95 Mb and*LOC787057*
SSB	0.0096 – 0.0554	-	-	-	3-17	BTA18: *SIGLEC12*
DCE	0.0173 – 0.1061	1-1,2	-	-	-	BTA18: *SIGLEC12*,
						53.95 Mb and*LOC787057*
NM	2.2525 – 16.2685	2-1,4	-	-	-	BTA18: *SIGLEC12*,
						53.95 Mb and*LOC787057*
STA	0.0160 – 0.0453	3-19,	-	3-14,	-	BTAX:*MIR584-8~KAL1*,
		12-12		7,12-9,10		BTA11: *LOC529399*
						BTA11:*LOC521556*
						BTAX: 145.06 Mb
BD	0.0087 – 0.0458	-	3-1	-	5-2	BTA18: *SIGLEC12*
RW	0.0144 – 0.0394	1-4	5-1	-	-	BTAX: 145.24 Mb,
						BTA18: *SIGLEC12*
DF	0.0140 – 0.0544	-	1-1	-	-	BTAX: *MIR584-8~KAL1*
FUA	0.0146 – 0.0465	2-16	-	-	-	BTAX: *MIR584-8~KAL1*
RUH	0.0146 – 0.0767	1-6	-	-	-	BTAX: *MIR584-8~KAL1*
FTP	0.0126 – 0.0482	-	17-7	-	17-2	BTA7: *SNCAIP-ZNF474*
FS	0.0100 – 0.0673	1-7	-	-	-	BTAX: *MIR584-8~KAL1*

MY, milk yield; FY, fat yield; PY, protein yield; FPC, fat percentage; PPC, protein percentage; PL, productive life; SCS, somatic cell score; DPR, daughter pregnancy rate; SCE, service-sire calving ease; SSB, service-sire stillbirth; DCE, daughter calving ease; NM, net merit; STA, stature; BD, body depth; DF, dairy form; RW, rump width; FUA, fore udder attachment; RUH, rear udder height; FTP, front teat placement; FS, final score.

^1^ The number on the left is the AIPL effect rank and the number on the right is the significance rank of this method for the same marker effect.

^2^ Underlined ranking is not an exact match to an AIPL effect and the exact location is the underlined location in the same row under ‘Gene region’.

The SNP effects confirmed by AIPL’s large effect sizes and statistical significance in this study could be considered as consensus SNP effects in U.S. Holstein cattle given the large sample size in the USDA Holstein genomic evaluation.

### Consensus effects in elite cows

Genetic variants relevant to the unique genetics of the elite cows should have high frequencies in the elite cows and low frequencies in the average cows. Similarly, SNP markers with similar frequencies between the elite and average clusters should be less likely to be the genetic factors separating the elite cows from the average cows. Allele frequency differences (AFD) between the elite and average cows showed that the consensus effects unlikely were the most important genetic effects for the unique genetics of the elite cows.

**Figure 6:**
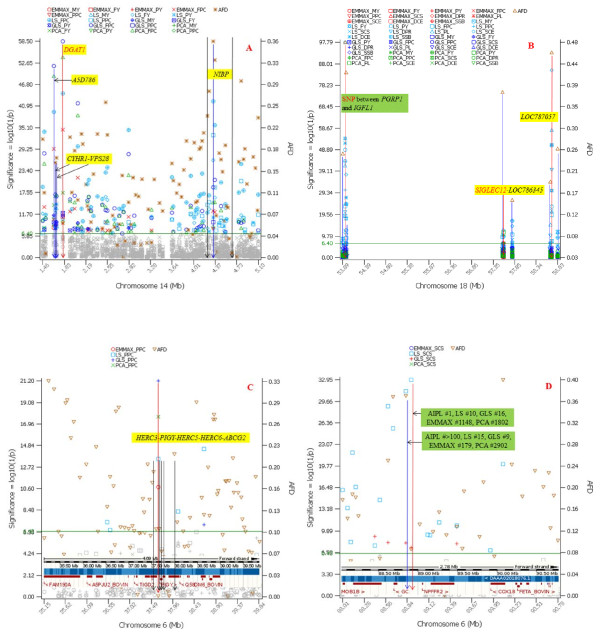
**SNP effects and allele frequency differences (AFD) between the elite cows and the average cows for four chromosome regions with confirmation between GWAS and effect size distribution from USDA genomic evaluation.A**) The *DGAT1-NIBP* region for production traits, showing that the consensus effect in *DGAT1* had a low AFD of 0.05 while a SNP in *NIBP* had the largest AFD (0.36) in this region and also was also highly significant for protein percentage (#2 by EMMAX-IBS and EMMAX-BN, #3 by GLS, #7 by PCA, and #19 by LS). **B**) The BTA18 region for production, somatic cell score, daughter pregnancy rate and calving traits, showing that the two SNP markers detected by the LS method for many traits had the largest AFD (0.48 at 53.95 Mb and 0.46 at 58.7 Mb) in this region while the consensus effect in *SIGLEC12* had a low AFD of 0.03. In this figure, the vertical red line indicates the significant marker, and the vertical blue line indicates an adjacent marker. **C**) The BTA6 region for protein percentage, showing that the consensus effect between *HERC3* and *PIGY* had nearly identical frequencies in the elite and the average cows. **D**) The BTA6 region for somatic cell score, showing that the consensus effect had a low AFD of 0.09, while the upstream marker identified by LS and GLS as highly significant for somatic cell had a high AFD of 0.37.

The widely confirmed *DGAT1* gene had the most significant SNP (ARS-BFGL-NGS-4939) for fat percentage. The ‘*A*’ allele of this SNP was favorable for milk yield but was unfavorable for fat yield and fat percentage, and the ‘*G*’ allele was the opposite, favorable for fat percentage and fat yield and unfavorable for milk yield. This SNP had AFD of 0.05 between the elite and average clusters, noting that more than half of the 45,878 SNP loci had AFD greater than 0.10 between the two clusters. Moreover, the favorable allele frequency in the elite cluster for fat yield was 0.05 less than in the average cluster, 0.13 in the elite cluster and 0.18 in the average cluster. Therefore, it is questionable whether *DGAT1* had a major role in the elite cows for fat yield given that 87% of the *DGAT1* alleles in the elite cluster had unfavorable effects for fat yield. The 87% frequency of the favorable *DGAT1* allele for milk yield (unfavorable for fat yield) was consistent with the high milk production levels of the elite cows but again was unlikely to have a major role in the milk production levels of the elite cows because the frequency of the favorable allele for milk yield was only 5% higher in the elite than in the average. In contrast, the *NIBP* region with highly significant LS effects for fat percentage, fat yield, protein yield and protein percentage had the largest AFD (0.36) between the elite and the average cows in the *DGAT1-NIBP* region (Figure 6A).

The *SIGLEC12* gene had large AIPL effects for six functional traits, productive life, daughter pregnancy rate, service-sire calving ease, service-sire stillbirth, daughter calving ease, and net merit. The effect of service-sire calving ease was confirmed by EMMAX and PCA, and the effect of service-sire stillbirth was confirmed by PCA (Table 2). However, the favorable allele of *SIGLEC12* had similar frequencies in the elite cows (0.945) and the average cows (0.914). The SNP immediately upstream of the *SIGLEC12* effect had large AFD (0.376) but was insignificant by any of the five methods of SNP testing. The AIPL effect size distributions ranked this marker #3 for daughter calving ease, #18 for service-sire calving ease, and #20 for body depth. For five of the six functional traits (except service-sire stillbirth) with large AIPL effects at the *SIGLEC12* marker, the most significant markers by LS had large AFD between the elite and the average at 53.95 Mb (AFD = 0.48) and *LOC787057* (AFD = 0.46) (Figure 6B).

For protein percentage, the favorable allele of Hapmap24324-BTC-062449), #1 by all methods for stratification correction and by the AIPL effect size distribution, had nearly identical frequency in both elite and average, about 0.967 in each cluster (Figure 6C). For somatic cell score, the favorable allele of the SNP marker (BTB-01654826) between *GC* and *NPFFR2* (#1 by AIPL effect size, #10 by LS and #16 by GLS) had an AFD 0.08 (allele frequency of 0.48 in the elite and 0.40 in the average). The favorable allele of the SNP (ARS-BFGL-NGS-17376) immediately upstream of BTB-01654826 had an AFD of 0.37 (0.64 in the elite and 0.27 in the average) and was ranked #15 by LS and #9 by GLS, but this marker was not among the top 100 effects for AIPL, EMMAX (#179) and PCA (#2902) (Figure 6D).

The results of AFD for the favorable alleles of the consensus effect showed that the consensus effects for production, fertility and calving traits had similar frequencies for the favorable alleles in the elite and the average cows. These results indicate that the consensus effects shown in Figure 6 may not explain the unique genetics of the elite cows and that the unique genetics of the elite cows likely involved additional genes not represented by genes with consensus effects.

### Elite cows and favorable alleles with minor frequencies

Dairy GWAS results in Cole et al. [6] had highly significant effects with low frequencies for the favorable alleles in the study population of 1,654 cows and had a large number of significant effects from the X chromosome. The analysis of genome, pedigree and phenotype stratifications identified the most likely reason for the rare favorable alleles: the rare favorable alleles had high frequencies in the elite cluster of 160 cows and low frequencies in the remaining 1,494 cows in the average cluster. Since each sire contributes 50% of the alleles to its daughters, sire alleles that had low population allele frequencies could have allele frequencies around 50% among the 153 daughters in the elite cluster, creating substantial comparison of allelic effects between the elite and the rest. This type of allelic comparison could identify true effects because elite cows are a small percentage of the population and should have high frequencies of favorable alleles that were rare in general populations. Although alleles satisfying this condition could have false positive effects due to chance, alleles that do not satisfy this condition should be less likely to be relevant to the elite status. The SNP markers with favorable alleles overwhelmingly possessed by the elite cluster should have provided a pool of SNP markers that are potentially responsible for the unique genetics of the elite cows.

Many of the highly significant SNP markers with rare favorable alleles had high frequencies in the elite cluster and low frequencies in the average cluster. A total of 298 SNP markers had AFD of 0.4 or greater and 2,248 SNP markers had AFD of 0.3 or greater between the elite and average clusters. Most of these markers had some effects on the 31 dairy traits by the LS method [6], particularly those with larger AFD. This fact indicates that the presence of the elite cluster contributed to the detection of significant SNP effects, although it is unknown which effects are true or false positive effects.

A notable SNP is BTA18’s BFGL-NGS-117985 with a minor allele frequency of 0.091 (for allele *G*) in the 1,654 cows. Among the 160 elite cows, the same *G* allele had a frequency of 0.53, compared to 0.05 in the average cluster of 1,494 cows (Additional file 10: Table S4). The *G* allele was the favorable allele for fat, protein and milk yields, fat and protein percentages, productive life, and net merit (high PTA values); and was the favorable allele for somatic cell score, service-sire calving ease, daughter calving ease and service-sire stillbirth (low PTA values). This SNP had the eighth largest AFD (0.48) between the elite and average clusters. Other highly significant SNP with large AFD included the most significant SNP for daughter pregnancy rate and somatic cell score (ARS-BFGL-NGS-4774) near *INSR* on BTA7 with AFD of 0.34, the second most significant SNP on BTA2 (ARS-BFGL-NGS-77438) for somatic cell score with AFD of 0.41, the most significant SNP for milk yield on BTA13 (ARS-BFGL-NGS-4774) with AFD of 0.38, the SNP with the largest number of highly significant effects for body conformation traits in *REN* on BTA16 (ARS-BFGL-NGS-83607) with AFD of 0.23, the most significant SNP on BTA26 near *MGMT* for foot angle (Hapmap28514-BTA-163525) with AFD of 0.43, and many SNP loci for dairy form, rear legs (side view), foot angle, strength, udder depth, and teat length (Additional file 10: Table S4). The unequal allele frequencies between the elite and average clusters indicate that the above SNP markers could be relevant to the elite cows. Nearly all SNP markers highly significant for fat yield without stratification correction [6] had large AFD between the elite and average clusters. For example, the *NIBP* marker that was ranked #76 by the LS method had AFD of 0.36, with favorable allele frequency of 0.65 in the elite cluster and 0.29 in the average cluster (Additional file 10: Table S4). This marker was also highly significant for protein percentage (#2 by EMMAX-IBS and EMMAX-BN, #3 by GLS, #7 by PCA, and #19 by LS).

### Elite large half-sib family and X chromosome effects

Some of the highly significant X chromosome SNP markers reported in Cole et al. [6] had large AFD between the elite and average clusters. However, not all X chromosome markers with large AFD were highly significant. The 153 half sibs of the 160 elite cows should have identical sire X chromosomes except the pseudo-autosomal region. Therefore, X chromosome allele frequencies in the elite cluster could be substantially higher than in the average cluster. Among the 298 SNP markers with AFD of 0.4 or greater, 50 markers were the X chromosome markers (16.8%). In Cole et al. [6], ARS-BFGL-NGS-1096 was in a gene desert on chromosome X but was highly significant for protein percentage (#1), fat percentage (#4), service-sire calving ease (#5), fat yield (#9), protein yield (#11) and productive life (#12). This marker had AFD of 0.51 (second largest AFD), with favorable allele frequency of 0.53 in the elite cluster and 0.02 in the average cluster. The marker with the largest AFD (0.54) was also on the X chromosome (ARS-BFGL-NGS-19641), with favorable allele frequency of 0.545 in the elite and 0.032 in the average, but this marker was not among the top 20 effects for any of the 31 traits, indicating that large AFD alone was not guaranteed to be highly significant. The X chromosome SNP (Hapmap50291-BTA-25028) with the fourth largest AFD also was not among the top 20 effects for any trait. The SNP (ARS-BFGL-NGS-18028) 30.1 kb upstream of *LOC52005* had AFD of 0.47, and was #1 for productive life and #4 for somatic cell score. The X chromosome SNP that was highly significant for several body conformation traits, Hapmap46795-BTA-30632 in *PHKA2*, had AFD of 0.32.

### LS analysis without the elite cows

To test the hypothesis that the significant SNP results from the LS analysis was influenced by the elite cows and that stratification correction may have removed some true SNP effects we performed a separate LS analysis for 1,494 of the 1,654 cows by removing the 160 elite cows from the data set. Without the elite cows, many of the highly significant LS results for dairy functional traits disappeared, including BTA18′s BFGL-NGS-117985 for a large number of traits, and X chromosome markers ARS-BFGL-NGS-1096 and ARS-BFGL-NGS-18028 (Additional file 11: Figure S7). For the widely confirmed fat percentage effects on BTA14, the LS analysis without the elite cows nearly achieved the effect of stratification correction and eliminated most significant LS results with the elite cows except the *DGAT1-NIBP* region of BTA14. The highly significant BTA18, BTA26 and BTAX SNP effects with the elite cows were eliminated and the highly significant BTA5 and BTA17 effects became much less visible when the elite cows were removed (Figure 5). Similarly, many highly significant LS results for fat yield, protein yield, protein percentage, service-sire sire calving ease, daughter calving ease and foot angle were eliminated once the elite cows were removed. This analysis showed that many of the significant LS results for fat percentage and the other traits were due to the elite cows and that stratification correction removed effects associated with the elite cows. Without the LS analysis, genetic information unique to the elite cows would have been left undetected.

The GLS and EMMAX methods for stratification correction were little affected by the removal of the elite cows, further indicating that the SNP effects removed by stratification corrections were largely associated with the elite cows. A few exceptions were increased significance for some traits with the removal of the 160 elite cows. Increased significance of EMMAX-IBS included the BTA26 effects for milk yield, somatic cell score and feet and legs, and BTA23 effects for fore udder attachment and rear teat placement, and increased GLS significance included some BTA20 effects for udder cleft (Additional file 11: Figure S7).

### Combined analysis of correlation based methods and PCA

The methods of correlation based, EMMAX and GLS, were combined with PCA by adding the top 20 PC’s to the statistical model for EMMAX-IBS and GLS. EMMAX-PCA generally had slight decreases in significance, and GLS-PCA analysis substantially reduced the GLS significance for most traits. Two exceptions to the reduced significance resulting from adding PCA included the increased significance of the BTA18 SNP effect for PL by EMMAX-PCA and for GLS-PCA, and the increased significance for the BTA18 effect on RW by GLS-PCA that were consistent with the AIPL results. However, adding PCA diminished several significant effects from GLS that were consistent with the AIPL results, including the BTA14 effects for protein yield and the BTA6 effect for somatic cell score (Additional file 11: Figure S7).

## Discussion

### Stratification correction may remove true effects associated with selection

Genetic selection and artificial insemination are major factors contributing to population stratification in dairy cattle. Genetic selection since 1964 has resulted in phenotypic changes for many traits. Most of the 31 traits in this study had phenotypic changes in one direction since 1964 (Table 3). Associated with phenotypic changes and breeding practice are gene frequency changes due to genetic selection. The widespread use of artificial insemination in dairy breeding accelerates the replication of selected male genomes. Genome sampling (drift) in breeding practice inevitably would contribute to genome stratification and spurious effects because only a fraction of the population is maintained for producing the next generation. Genetic hitchhiking of selection, which refers to allele frequency changes due to linkage disequilibrium with loci subjected to selection [16], also contributes to genome stratification and spurious association effects.

**Table 3:** **Predicted transmitting ability (PTA) values (mean** ± **standard deviation) for three groups of Holstein cattle representing three stages of artificial selection since 1964**

**Trait**	**Group I (n = 301) Unselected since 1964**	**Group II (n = 215) Born 1975-1985**	**Group III (n = 1,654) Contemporary cows Born 1990-2008**	**Direction of change (II–I, III–II)**
MY	−1118.6±552.3	−403.5±246.8	301.3±239.3	↑↑
FY	−39.2±19.4	−12.2±8.2	11.2±10.3	↑↑
PY	−31.7±15.7	−13.6±6.4	10.3±7.5	↑↑
FPC	0.015±0.035	0.023±0.066	0.002±0.065	↑↓
PPC	0.016±0.018	−0.014±0.037	0.011±0.032	↓↑
PL	−3.093±1.785	−0.327±1.403	1.227±1.982	↓↑
SCS	2.854±0.075	2.948±0.103	2.974±0.165	↑↑
DPR	2.909±1.170	0.558±0.943	−0.151±1.322	↓↓
SCE	8.654±0.329	8.149±0.999	7.508±1.185	↓↓
DCE	7.884±0.175	9.015±0.918	7.664±1.052	↑↓
SSB	7.860±0.192	7.369±0.488	7.657±0.648	↓↑
DSB	8.483±0.694	9.987±0.708	7.661±1.098	↑↓
NM	−549.045±257.56	−211.581±107.18	223.822±178.13	↑↑
STA	−1.840±1.357	−1.146±0.775	0.413±0.990	↑↑
STR	−0.875±0.871	−0.629±0.853	0.198±0.872	↑↑
BD	−1.508±1.225	−0.855±0.866	0.271±0.876	↑↑
RW	−1.705±1.220	−0.918±0.837	0.276±0.903	↑↑
DF	−3.232±2.482	−1.638±0.957	0.703±0.906	↑↑
RA	0.474±0.622	0.143±0.688	0.069±0.792	↓↓
FUA	−1.754±1.278	−1.479±0.825	0.641±1.110	↑↑
RUH	−2.749±2.013	−1.704±0.954	0.942±1.171	↑↑
UD	−0.782±0.730	−0.857±0.738	0.316±0.962	↓↑
UC	−2.114±1.538	−1.607±0.887	0.470±1.013	↑↑
FTP	−1.665±1.307	−1.447±0.906	0.564±0.916	↑↑
RTP	−1.797±1.461	−1.607±0.908	0.477±0.929	↑↑
TL	0.063±0.555	0.251±0.783	−0.076±0.758	↑↓
FA	−0.893±0.866	−0.867±0.964	0.622±1.052	↑↑
RLS	−0.447±0.616	−0.249±0.811	−0.163±0.769	↑↑
RLR	−1.241±1.134	−0.980±0.911	0.611±0.974	↑↑
FL	−1.176±1.033	−1.045±0.916	0.677±0.919	↑↑
FS	−2.173±1.607	−1.378±0.766	0.695±0.910	↑↑

MY, milk yield in units of kilograms; FY, fat yield in units of kilograms; PY, protein yield in units of kilograms; FPC, fat percentage; PPC, protein percentage; PL, productive life; SCS, somatic cell score; DPR, daughter pregnancy rate; SCE, service-sire calving ease; DCE, daughter calving ease; SSB, service-sire stillbirth; DSB, daughter stillbirth; NM, net merit; STA, stature; STR, strength; BD, body depth; DF, dairy form; RA, rump angle; RW, rump width; FUA, fore udder attachment; RUH, rear udder height; UD, udder depth; UC, udder cleft; FTP, front teat placement; RTP, rear teat placement; TL, teat length; FA, foot angle; RLS, rear legs (side view); RLR, rear legs (rear view); FL, feet/legs score; FS, final score.

The three methods used in this study for stratification correction eliminated most effects associated with the elite cluster, indicating that the removal of stratification effects likely have removed some true effects associated with genetic selection.

The mechanism of removing true effects associated with selection is different for different methods. For methods using correlation among individuals such as EMMAX and GLS, the mechanism of removing true effects associated with selection is the correction of an individual’s phenotypic value by phenotypic values of all related individuals so that the corrected individual phenotypic values may become less different from each other. For the PCA approach, the mechanism of removing true effects associated with selection is the correlation between principal components and phenotypic values. The stratification analysis in this study showed that genome and phenotype stratifications overlapped (Additional file 4: Figure S4). Consequently, the removal of genome stratification by PCA likely also removes true genetic effects associated with the phenotypic stratification. It should be noted that the PCA approach has a problem of subjectivity in choosing the number of principal components because the use of different numbers of principal components may result in different results. For the dairy GWAS data in this study, the use of the first component (data not shown) and the use of the first 20 components (Additional file 9: Table S3) had drastically different results. Current methods for stratification correction in association analysis do not separate drift and hitchhiking from selection. Selection signature analysis may provide evidence for genes and chromosome regions affected by selection [17,18]. Sample structure may differ for different genomic regions, and adjustment for local stratification may be instructive to accurately localize true signals [19,20].

### Cleaner signals from stratification correction for large effects without genetic selection

The methods for stratification correction in association analysis could yield cleaner signals of SNP effects and could be used for detecting potential large effects for traits without genetic selection. Two of the 31 traits in our study clearly were not subjected to genetic selection since 1964: fat and protein percentages. These two traits were unchanged since 1964, with slight increase before 1985 and slight decrease after 1985 for fat percentage, and slight decrease before 1985 and slight increase after 1985 for protein percentage (Table 3). The fat percentage effects of BTA14 is an excellent example showing how stratification correction could reduce potential noise and produce much cleaner signals of significant effects (Figure 5). The two models of EMMAX, EMMAX-IBS and EMMAX-BN, had the cleanest signals, virtually eliminating all effects except those in and near the *DGAT1-NIBP* region of BTA14. However, it is unknown whether such clean signals came at the expense of the elimination of some less significant true effects. Another unknown is whether the fat percentage effects could have been detected with similar statistical confidence had fat percentage been subjected to many years of directional selection. Comparing protein percentage that also was not subjected to selection with fat percentage, it should be apparent that protein percentage did not have similar major effects to those for fat percentage in our study population of 1,654 contemporary Holstein cows. This assumption was supported by the effect sizes from the USDA Holstein genomic evaluation. The effect size range of the top 20 effects for fat percentage was 3.9 times as large as the effect size range of the top 20 effects for protein percentage (Table 2).

## Conclusions

Genetic selection and artificial insemination were major factors associated with Holstein genome stratification that overlapped with pedigree and phenotype stratifications. The confounding between genome and phenotype stratifications may provide opportunity to discover genetic effects and cause difficulty in separating true and spurious effects. Stratification correction in genome-wide association analysis in Holstein cattle could remove true effects associated with stratification due to genetic selection.

## Methods

### Phenotypic data, study population and SNP genotyping

Thirty one production, health, and reproduction traits were studied, including 13 production, health, reproduction traits, and 18 body conformation traits. Traditional predicted transmitting abilities (PTAs) for each trait calculated by the U.S. Department of Agriculture (USDA; Beltsville, MD) were phenotypic data for association with SNPs. The association analysis included 1,654 contemporary U.S. Holstein cows, the same study population as in Cole et al. [6]. The analysis of sample stratification also used a selection signature data set that included historical and contemporary Holstein cattle for a total of 2,366 cows and bulls. The historical Holstein cattle were the University of Minnesota Holstein control line and bulls born before 1964 (301 cows and bulls). The historical pedigree of the 1,654 Holstein cows from Animal Improvement Programs Laboratory of USDA included 34,668 individuals, with the oldest cattle born in 1930. The pedigree figure of this data set was produced by Pedigraph 2.3 [21]. DNA extraction and SNP genotyping for 45,878 SNP markers using the BovineSNP50 BeadChip (Illumina, San Diego, CA) were performed at the Bovine Functional Genomics Laboratory (Agricultural Research Service, U.S. Department of Agriculture, Beltsville, MD).

### Three methods for stratification correction

Three methods for stratification correction were evaluated, including a mixed model method implemented by EMMAX [3], a GLS method based on intraclass correlation [7], and a PCA method [2].

EMMAX was used in our analysis because of its optimized algorithm and fast computational speed in solving large mixed model systems [3]. The EMMAX method uses a linear mixed model approach that includes correlation among all individuals. The statistical model can be expressed as: **y** = μ + **Zβ**_Z_ + **Gβ** + **e**_,_ where **y** = column vector of phenotypic values, μ = population mean of the phenotypic values, **Z** = incidence matrix for a random additive effects, **β**_Z_ = column vector of the random additive effects, **G** = the genotype value of the candidate SNP, **β** = the regression coefficient of the candidate SNP, and **e** = random residual. The phenotypic variance-covariance matrix is: var(**y**) = Var(**β**_Z_) + var(**e**) = **K**σ_a_^2^ + **I**σ_e_^2^, where **K** = IBS [3] or the Balding-Nichols kinship matrix [10], **I** = identify matrix, σ_a_^2^ = additive variance, and σ_e_^2^ = variance of random residuals.

Since the genome stratification overlapped with half-sib families, we used a GLS approach [7] to account for correlation within half-sib families based on intraclass correlation [22]. The phenotypic values of a quantitative trait is assumed to be: **y** = **Xg** + **Zf** + **e**, where **y** = vector of phenotypic values, **g** = effects of SNP genotypes, **X** = model matrix of **g**, **f** = random family effects with a common variance σ_f_^2^ for sibs in the same family that could include common genetic and environmental effects, **Z** = model matrix of **f**. The variance-covariance matrix of the family effects is assumed to be **G** = var(**f**) = **I**σ_f_^2^. Then, the phenotypic variance-covariance matrix is Var(**y**) = **V** = **ZGZ’** + **I**σ_e_^2^. The phenotypic values are assumed to follow a normal distribution with mean **Xg** and variance-covariance matrix **V**, which can also be expressed as $\text{Var}\left(y\right)=V=\overset{m}{\underset{i=1}{⊕}}V_{i}$, where **V**_i_ = variance-covariance matrix of phenotypic values of sibs in family i, and m = number of families. To improve computational efficiency in the setting of genome-wide SNP analysis, a simple formula of the **V** inverse was developed so that the direct inversion of the **V** matrix is no longer needed [7,8]. Let σ^2^ = σ_f_^2^ + σ_e_^2^, ρ = σ_f_^2^/σ^2^ = intraclass correlation, n = total number of observations, r = ρ/[1 + (n_i_ – 1)ρ], and λ = r/(1 – r), where n_i_ = the number of sibs in family i. Also let **V**^–1^ = **L’L**, where **L** is an upper triangular matrix. Then, each element in **L** can be calculated as

$$L_{\text{ii}}=\sqrt{\frac{\left(1+\lambda \right)\left[1-\left(i-1\right)\lambda \right]}{1-\left(i-2\right)\lambda }\frac{1-r}{\sigma_{e}^{2}}}\;i=1,\ldots ,n$$

$$L_{\text{ij}}=-\lambda L_{\text{ii}}/\left[1-\left(i-1\right)\lambda \right],\;i=2,\ldots ,n;\;j=i+1,\ldots ,n-1$$

With the above formulae, the **V**^–1^ can be constructed without actually inverting the **V** matrix. Variance components in the intraclass correlation, σ_f_^2^ and σ_e_^2^, were estimated using a maximum likelihood approach [7,23]. Estimates of the three SNP genotypic values were obtained by the following GLS solution:

$$\hat{g}={\left({\text{X}}_{2}^{*'}{\text{X}}_{2}^{*}\right)}^{-1}\left({\text{X}}_{2}^{*'}y_{2}^{*}\right)={\left({\text{X}}_{2}^{'}{\text{V}}^{-1}{\text{X}}_{2}^{*}\right)}^{-1}\left({\text{X}}_{2}^{'}{\text{V}}^{-1}\text{y}\right)$$

where **X**_2_* = **L’X**_2_ and y* = **L’y** = corrected phenotypic values after removing fixed non-genetic effects. Let k = the rank of **X** = **X**_1,_**X**_2_, s^2^ = residual mean sum of squares, and **s**_i_ = row vector of contrast coefficient for defining additive or dominance effects based on the extended Kempthorne model [24]. Then, the following test statistic for testing each genetic effect (additive or dominance effect) follows a Student-t distribution with n – k degrees of freedom:

T^x=LxVa^rLx=sig^siX2'V−1X2−1si'=sig^siX2∗'X2∗−1si'

Stratification correction to the phenotypic value of each individual was also applied using the approach of principal component analysis (PCA) [2] as a comparison to the two methods using correlation among individuals. The top 20 principal components were used as covariables for stratification correction. The statistical model was: y = μ + Σx_i_b_i_ + g + e, where Σx_i_b_i_ = sum of principal component effects, with x_i_ = eigenvector of principal component i, and b_i_ = regression coefficient of principal component i, i = 1,…20. Statistical tests of SNP effects by the GLS and PCA methods were implemented using the epiSNP computer package [25]. A genome-wide 5% type-I error with the Bonferroni correction was considered as the threshold P value (10^−6.4^) for genome-wide significance. Gene and SNP locations were identified based on the University of Maryland bovine genome assembly [26,27]. SNP positions were based on UMD3.1 and gene locations were based on ENSEMBL [28] and NCBI [29].

## Abbreviations

GWAS: Genome-wide association study; SNP: Single nucleotide polymorphism; Mb: Mega bases pairs = 1000 kb = 1 million base pairs; PTA: Predicted transmitting ability; QTL: Quantitative trait locus; BTA: *Bos taurus*; EMMAX: Efficient mixed-model association eXpedited; LS: Least squares; GLS: Generalized least squares; PCA: Principal component analysis; IBS: Identify by state; AFD: Allele frequency difference; MY: Milk yield; FY: Fat yield; PY: Protein yield; F%: Fat percentage; P%: Protein percentage; PL: Productive life; SCS: Somatic cell score; DPR: Daughter pregnancy rate; SCE: Service-sire calving ease; DCE: Daughter calving ease; SSB: Service-sire stillbirth; DSB: Daughter stillbirth; NM: Net merit; STA: Stature; STR: Strength; BD: Body depth; RW: Rump width; DF: Dairy form; RA: Rump angle; FUA: Fore udder attachment; RUH: Rear udder height; UD: Udder depth; UC: Udder cleft; FTP: Front teat placement; RTP: Rear teat placement; TL: Teat length; FA: Foot angle; RLS: Rear legs (side view); RLR: Rear legs (rear view); FL: Feet/legs score; FS: Final score.

## Competing interests

The authors declare that they have no competing interests.

## Authors’ contributions

YD, TSS, GRW, JBC, CPVT, TJL and BAC organized and implemented this study. YD, LM, and SW led the data analysis. LM, GRW, SW and YD led the manuscript preparation. GRW provided the USDA historical Holstein pedigree data, JBC provided the effect size distribution data from USDA Holstein genomic evaluation, and TJL provided the Holstein body conformation data. TSS directed the genotyping work. All authors read and approved this manuscript.

## Supplementary Material

Additional file 1 **Figure S1.** Multidimensional scaling (MDS) plots of SNP genotypes of 1,654 contemporary Holstein cows by chromosome. C1 = dimension 1, C2 = dimension 2. Left column: C1 and C2 values were calculated using 1,654 contemporary cows. Right column: C1 and C2 values were calculated using 2,366 Holstein cattle, including the University of Minnesota Holstein control line that remained unselected since 1964. Click here for file

Additional file 2 **Figure S2.** Pedigree of the 1,654 contemporary cows tracing back to ancestors born in 1930’s (approximately 10–15 generations). Circles in gold color are the 1,654 cows used in the genome-wide association analysis. The pedigree shows that all 1,654 cows are related. Click here for file

Additional file 3 **Figure S3.** Overlap between genome stratification and half-sib family structure. C1 = dimension 1, C2 = dimension 2. Left column: C1 and C2 values were calculated using 1,654 contemporary Holstein cows. Right column: C1 and C2 values were calculated using 2,366 Holstein cattle, including the University of Minnesota Holstein control line that remained unselected since 1964. Click here for file

Additional file 4 **Figure S4.** Overlap between genome stratification and phenotypic stratification of 31 traits. C1 = dimension 1, C2 = dimension 2. Left column: C1 and C2 values were calculated using 1,654 contemporary Holstein cows. Right column: C1 and C2 values were calculated using 2,366 contemporary and historical Holstein cattle, including the University of Minnesota Holstein control line that remained unselected since 1964. ‘Top 200’ are the 200 cows with the highest PTA values for the trait, ‘Bottom 200’ are the 200 cows with the lowest PTA values for the trait, and ‘Other’ are cows with PTA values between top 200 and bottom 200. Click here for file

Additional file 5 **Figure S5.** Overlap between genome stratification and phenotypic stratification for chromosome 1 and the X chromosome. Column 1: chromosome 1; Column 2: X chromosome; C1 and C2 values were calculated using 1,654 contemporary Holstein cows. Column 3: chromosome 1; Column 4: X chromosome; C1 and C2 values were calculated using 2,366 Holstein cattle, including the University of Minnesota Holstein control line that remained unselected since 1964. C1 = dimension 1, C2 = dimension 2; ‘Top 200’ are the 200 cows with the highest PTA values for the trait, ‘Bottom 200’ the 200 cows with the lowest PTA values for the trait, and ‘Other’ are cows with PTA values between top 200 and bottom 200. Click here for file

Additional file 6 **Figure S6.** Global view of P-values of 45,878 SNP effects per trait for 31 production, health, reproduction and body conformation traits by three methods for stratification correction. MY, milk yield; FY, fat yield; PY, protein yield; FPC, fat percentage; PPC, protein percentage; PL, productive life; SCS, somatic cell score; DPR, daughter pregnancy rate; SCE, service-sire calving ease; DCE, daughter calving ease; SSB, service-sire stillbirth; DSB, daughter stillbirth; NM, net merit; STA, stature; STR, strength; BD, body depth; DF, dairy form; RA, rump angle; RW, rump width; FUA, fore udder attachment; RUH, rear udder height; UD, udder depth; UC, udder cleft; FTP, front teat placement; RTP, rear teat placement; TL, teat length; FA, foot angle; RLS, rear legs (side view); RLR, rear legs (rear view); FL, feet and legs; FS, final score. Yellow triangle indicates confirmation among all for methods for stratification correction. Click here for file

Additional file 7 **Table S1.** (Excel file) Output file of top 100 effects on 31 dairy traits by EMMAX tests. Sheet 1: Results of EMMAX using identify by descent (IBS) among all individuals. Sheet 2: Results of EMMAX using the Balding-Nichols kinship matrix among all individuals. Chr30 is the X chromosome, and Chr32 indicates markers with unknown chromosome locations. MY, milk yield; FY, fat yield; PY, protein yield; FPC, fat percentage; PPC, protein percentage; SCS, somatic cell score; DPR, daughter pregnancy rate; PL, productive life; SCE, service-sire calving ease; DCE, daughter calving ease; SSB, service-sire stillbirth; DSB, daughter stillbirth; NM, net merit; STA, stature; STR, strength; BD, body depth; RW, rump width; DF, dairy form; RA, rump angle; FUA, fore udder attachment; RUH, rear udder height; UD, udder depth; UC, udder cleft; FTP, front teat placement; RTP, rear teat placement; TL, teat length; FA, foot angle; RLS, rear legs (side view); RLR, rear legs (rear view); FL, feet and legs; FS, final score. Click here for file

Additional file 8 **Table S2.** (Excel file) Output file of top 100 effects on 31 dairy traits by generalized least squares (GLS) tests. Chr30 is the X chromosome, and Chr32 indicates markers with unknown chromosome locations. MY, milk yield; FY, fat yield; PY, protein yield; FPC, fat percentage; PPC, protein percentage; SCS, somatic cell score; DPR, daughter pregnancy rate; PL, productive life; SCE, service-sire calving ease; DCE, daughter calving ease; SSB, service-sire stillbirth; DSB, daughter stillbirth; NM, net merit; STA, stature; STR, strength; BD, body depth; RW, rump width; DF, dairy form; RA, rump angle; FUA, fore udder attachment; RUH, rear udder height; UD, udder depth; UC, udder cleft; FTP, front teat placement; RTP, rear teat placement; TL, teat length; FA, foot angle; RLS, rear legs (side view); RLR, rear legs (rear view); FL, feet and legs; FS, final score. Click here for file

Additional file 9 **Table S3.** (Excel file) Output file of top 100 effects on 31 dairy traits with stratification correction based on principal component analysis (PCA) using to top 20 principal components as covariables. Chr30 is the X chromosome, and Chr32 indicates markers with unknown chromosome locations. MY, milk yield; FY, fat yield; PY, protein yield; FPC, fat percentage; PPC, protein percentage; SCS, somatic cell score; DPR, daughter pregnancy rate; PL, productive life; SCE, service-sire calving ease; DCE, daughter calving ease; SSB, service-sire stillbirth; DSB, daughter stillbirth; NM, net merit; STA, stature; STR, strength; BD, body depth; RW, rump width; DF, dairy form; RA, rump angle; FUA, fore udder attachment; RUH, rear udder height; UD, udder depth; UC, udder cleft; FTP, front teat placement; RTP, rear teat placement; TL, teat length; FA, foot angle; RLS, rear legs (side view); RLR, rear legs (rear view); FL, feet and legs; FS, final score. Click here for file

Additional file 10 **Table S4.** (Excel file) Overlap between top 100 effects per trait for 31 dairy traits from methods for stratification correction and the top 100 effects from the analysis without stratification correction. A1_elite160: frequency of allele 1 in the elite cluster of 160 cows; A1_1494: frequency of allele 1 in the remaining 1,494 cows excluding the elite cluster; Allele 1 = *A* for *AC*, *AG* and *AT*, = *C* for *CG* and *CT*, = *G* for *GT* (*GT* not observed in our SNP data set). MY, milk yield; FY, fat yield; PY, protein yield; FPC, fat percentage; PPC, protein percentage; SCS, somatic cell score; DPR, daughter pregnancy rate; PL, productive life; SCE, service-sire calving ease; DCE, daughter calving ease; SSB, service-sire stillbirth; DSB, daughter stillbirth; NM, net merit; STA, stature; STR, strength; BD, body depth; RW, rump width; DF, dairy form; RA, rump angle; FUA, fore udder attachment; RUH, rear udder height; UD, udder depth; UC, udder cleft; FTP, front teat placement; RTP, rear teat placement; TL, teat length; FA, foot angle; RLS, rear legs (side view); RLR, rear legs (rear view); FL, feet and legs; FS, final score. E: the effect from the method without stratification correction [6] was among the top 100 effects from EMMAX-IBS; G: the effect from the method without stratification correction [6] was among the top 100 effects from the GLS method; E: the effect from the method without stratification correction [6] was among the top 100 effects from PCA methods; EG: the effect from the method without stratification correction [6] was among the top 100 effects from EMMAX-IBS and GLS; EP: the effect from the method without stratification correction [6] was among the top 100 effects from EMMAX-IBS and PCA; GP: the effect from the method without stratification correction [6] was among the top 100 effects from GLS and PCA; EGP: the effect from the method without stratification correction [6] was among the top 100 effects from EMMAX-IBS, GLS and PCA. ‘0’ indicates this top 100 effect was not detected by EMMAX-IBS, GLS or PCA. Click here for file

Additional file 11 **Figure S7.** Manhattan plots of the AIPL effect distribution, and results from three sets of analysis: 1) LS, GLS, EMMAX-IBS using the full data set of 1,654 cows; 2) adding PCA to GLS and EMMAX-IBS using 1,654 cows; and 3) LS, GLS and EMMAX using 1,494 cows by removing the 160 elite cows. Red triangle indicates confirmation between effect size and significance test(s). Black triangle indicates confirmation of the AIPL effect by a nearby SNP marker. Black triangle indicates confirmation of the AIPL effect by a nearby SNP marker. Yellow triangle indicates confirmation between EMMAX and GLS. Green triangle indicates eliminated or reduced significance due to add PCA to GLS or EMMAX, or due to removing the 160 elite cows from the analysis. Blue triangle indicates increased significance due to add PCA to GLS or EMMAX, or due to removing the 160 elite cows from the analysis. Click here for file

## References

[B1] Devlin B Roeder K Genomic control for association studies Biometrics 1999 55 997 1004 10.1111/j.0006-341X.1999.00997.x 11315092

[B2] Price AL Patterson NJ Plenge RM Weinblatt ME Shadick NA Reich D Principal components analysis corrects for stratification in genome-wide association studies Nat Genet 2006 38 904 909 10.1038/ng1847 16862161

[B3] Kang HM Sul JH Service SK Zaitlen NA Kong S Freimer NB Sabatti S Eskin E Variance component model to account for sample structure in genome-wide association studies Nat Genet 2010 42 348 354 10.1038/ng.548 20208533 PMC3092069

[B4] Zhang Z Ersoz E Lai C-Q Todhunter RJ Tiwari HK Gore MA Bradbury PJ Yu J Arnett DK Ordovas JM Buckler ES Mixed linear model approach adapted for genome-wide association studies Nat Genet 2010 42 355 360 10.1038/ng.546 20208535 PMC2931336

[B5] Sonstegard TS Ma L Van Tassell CP Kim E-S Cole JB Wiggans GR Crooker BA Mariani BD Matukumalli LK Garbe JR Fahrenkrug SC Liu G Da Y Forty years of artificial selection in U.S. Holstein cattle had genome-wide signatures 2010 Leipzig, Germany: Poster presentation at 9th World Congr. Genet. Appl. Livest. Prod [ http://aipl.arsusda.gov/publish/presentations/WC9_10/WC9_10_yang_da.pdf]

[B6] Cole JB Wiggans GR Ma L Sonstegard TS Lawlor TJ Crooker BA Van Tassell CP Yang J Wang S Matukumalli LK Da Y Genome-wide association analysis of thirty one production, health, reproduction and body conformation traits in contemporary US Holstein cows BMC Genomics 2011 12 1 408 10.1186/1471-2164-12-408 21831322 PMC3176260

[B7] Ma L Generalized least squares method to account for sib correlation for testing SNP single-locus and epistasis effects in genome-wide association analysis 2010 University of Minnesota: Ph.D. thesis (Chapter 3). Department of Animal Science

[B8] Ma L Amos CI Da Y Accounting for correlations among individuals for testing SNP single-locus and epistasis effects in genome-wide association analysis [abstract] Plant anim genome XVIII conf abstr [online] 2008 International Plant & Animal Genome Conference http://www.intl-pag.org/16/abstracts/PAG16_P11_903.html

[B9] Purcell S Neale B Todd-Brown K Thomas L Ferreira MAR Bender D Maller J Sklar P de Bakker PIW Daly MJ Sham PC PLINK: a toolset for whole-genome association and population-based linkage analysis Am J Hum Genet 2007 81 559 575 10.1086/519795 17701901 PMC1950838

[B10] Balding DJ Nichols RA A method for quantifying differentiation between populations at multi-allelic loci and its implications for investigating identity and paternity Genetica 1995 96 3 12 10.1007/BF01441146 7607457

[B11] Zhao JH gap: Genetic analysis package J Stat Softw 2007 23 i08 http://www.jstatsoft.org/v23/i08/paper

[B12] Wang S Dvorkin D Da Y SNPEVG: A graphical tool for SNP effect viewing and graphing [ http://animalgene.umn.edu/snpevg/index.html],Version 3.1, June 6, 2012 10.1186/1471-2105-13-319 PMC3527193 23199373

[B13] VanRaden PM Efficient methods to compute genomic predictions J Dairy Sci 2008 91 4414 4423 10.3168/jds.2007-0980 18946147

[B14] Cole JB VanRaden PM O’Connell JR Van Tassell CP Sonstegard TS Schnabel RD Taylor JF Wiggans GR Distribution and location of genetic effects for dairy traits J Dairy Sci 2009 92 2931 2946 10.3168/jds.2008-1762 19448026

[B15] Wiggans GR VanRaden PM Cooper TA The genomic evaluation system in the united states: past, present, future J Dairy Sci 2011 94 3202 3211 10.3168/jds.2010-3866 21605789

[B16] Barton NH Genetic hitchhiking Phil. Trans. R. Soc. Lond. B 2000 55 1553 1562 11127900 10.1098/rstb.2000.0716 PMC1692896

[B17] Sabeti PC Varilly P Fry B Lohmueller J Elizabeth Hostetter E Cotsapas C Xie X Byrne EH McCarroll SA Gaudet R Schaffner SF Lander E & The International HapMap Consortium Genome-wide detection and characterization of positive selection in human populations Nature 2007 449 913 918 10.1038/nature06250 17943131 PMC2687721

[B18] Rubin CJ Zody MC Eriksson J Meadows JRS Sherwood E Webster MT Jiang L Ingman M Sharpe T Ka S Hallböök F Besnier F Carlborg Ö Bed’hom B Tixier-Boichard M Jensen P Siegel P Lindblad-Toh K Andersson L Whole-genome resequencing reveals loci under selection during chicken domestication Nature 2010 464 587 591 10.1038/nature08832 20220755

[B19] Qin H Morris N Kang SJ Li M Tayo B Lyon H Hirschhorn J Cooper RS Zhu X Interrogating local population structure for fine mapping in genome-wide association studies Bioinformatics 2010 26 2961 2968 10.1093/bioinformatics/btq560 20889494 PMC2982153

[B20] Shriner D Adeyemo A Ramos E Chen G Rotimi CN Mapping of disease-associated variants in admixed populations Genome Biol 2011 12 1 8 10.1186/gb-2011-12-5-223 PMC3219963 21635713

[B21] Garbe JR Da Y Pedigraph: A pedigree and genealogy visualization program for drawing large complex pedigrees User manual version 2.3 2004 University of Minnesota: Department of Animal Science

[B22] Donner A Koval JJ The estimation of intraclass correlation in the analysis of family data Biometrics 1980 36 19 25 10.2307/2530491 7370372

[B23] Hartley HO Rao JNK Maximum likelihood estimation for mixed analysis of variance model Biometrika 1967 54 93 108 6049561

[B24] Mao Y London NR Ma L Dvorkin D Da Y Detection of SNP epistasis effects of quantitative traits using an extended kempthorne model Physiol Genomics 2007 28 46 52 16940430 10.1152/physiolgenomics.00096.2006

[B25] Ma L Runesha HB Dvorkin D Garbe JR Da Y Parallel and serial computing tools for testing single-locus and epistatic SNP effects of quantitative traits in genome-wide association studies BMC Bioinforma 2008 9 315 10.1186/1471-2105-9-315 PMC2503991 18644146

[B26] Zimin AV Delcher AL Florea L Kelley DR Schatz MC Puiu D Hanrahan F Pertea G Van Tassell CP Sonstegard TS Marçais G Roberts M Subramanian P Yorke JA Salzberg SL A whole-genome assembly of the domestic cow Bos taurus. Genome Biol 2009 10 4 R42 10.1186/gb-2009-10-4-r42 PMC2688933 19393038

[B27] Zimin AV Puiu D Marcais G Delcher A Yorke JA Salzberg SL The latest high-quality bovine genome assembly, UMD Bos Taurus 3.0 [Abstract] Plant anim genome XVIII conf abstr [online] 2010 International Plant & Animal Genome Conference http://www.intl-pag.org/18/abstracts/W17_PAGXVIII_135.html

[B28] ENSEMBL Genome Browser. Release 63, June 2011 http://www.ensembl.org/index.html

[B29] National Center for Biotechnology Information (NCBI) [ http://www.ncbi.nlm.nih.gov]

