# Supplementary figures and images for: Effect of sample stratification on dairy GWAS results
Source: BMC Genomics. 2012 Oct 6;13:536. doi: 10.1186/1471-2164-13-536 (PMC3496570; doi:10.1186/1471-2164-13-536)

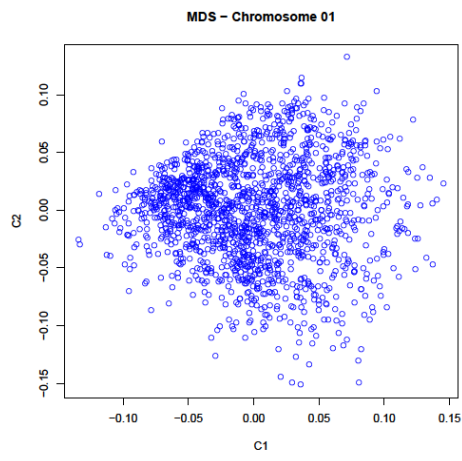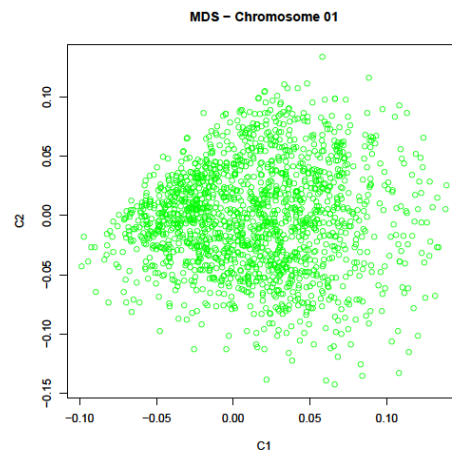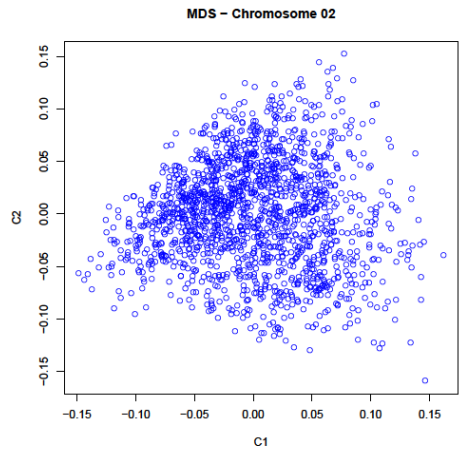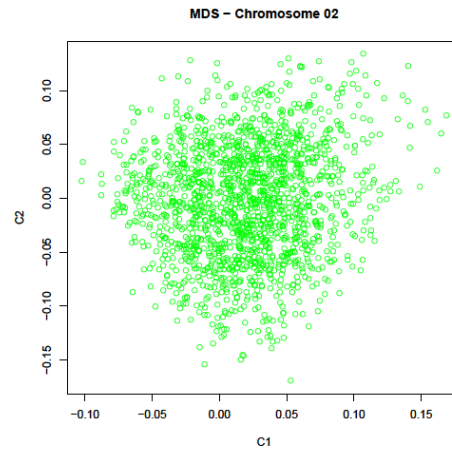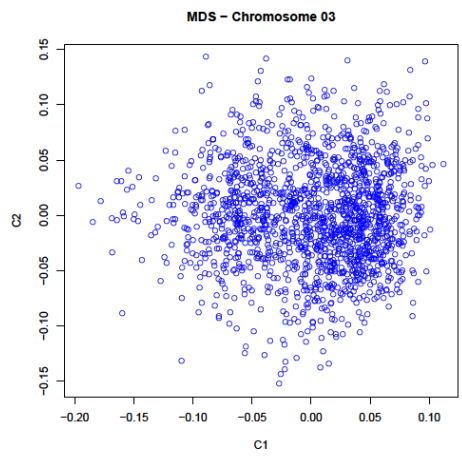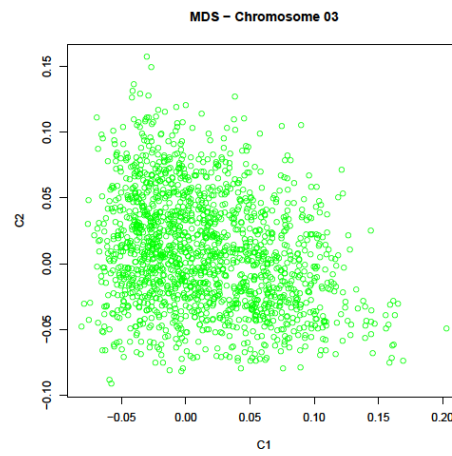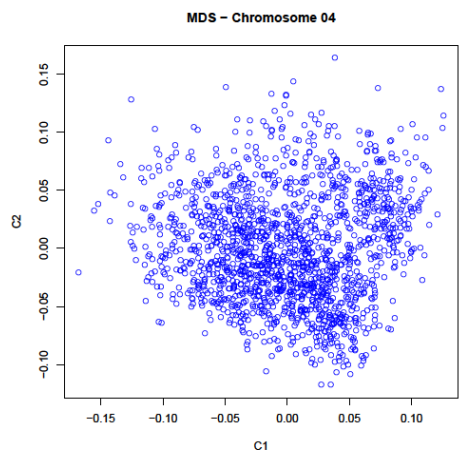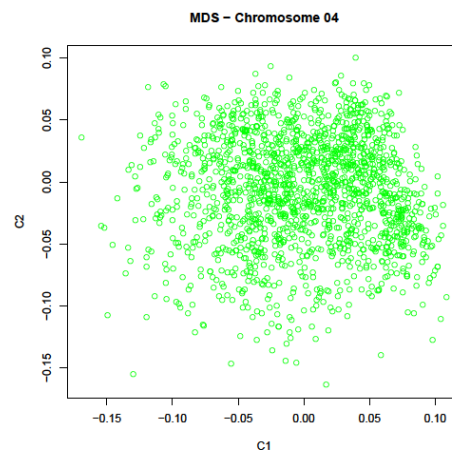

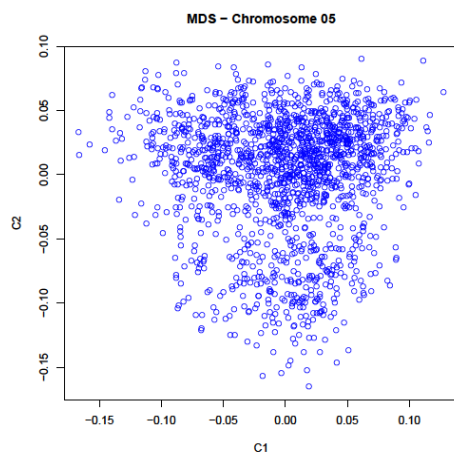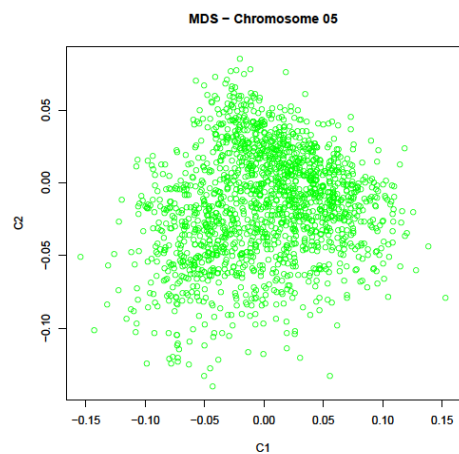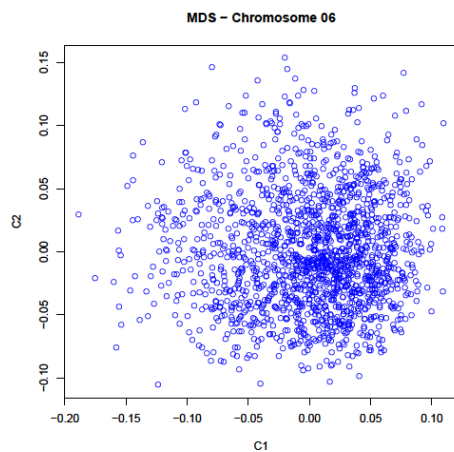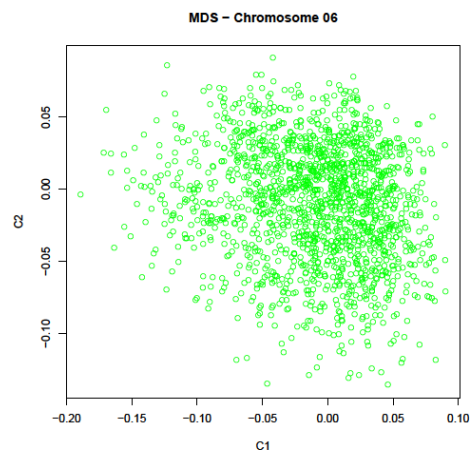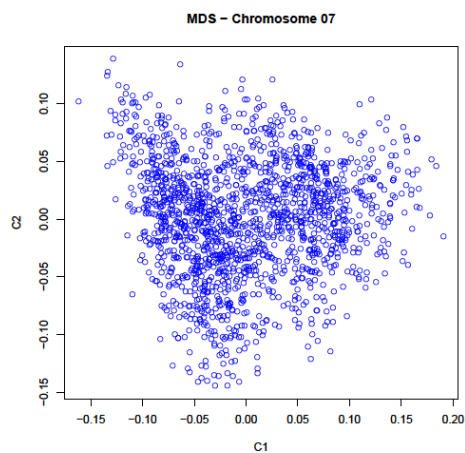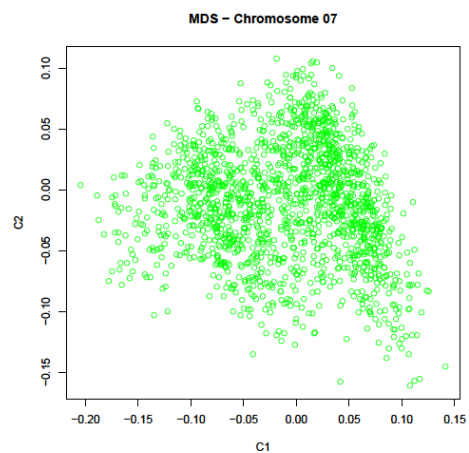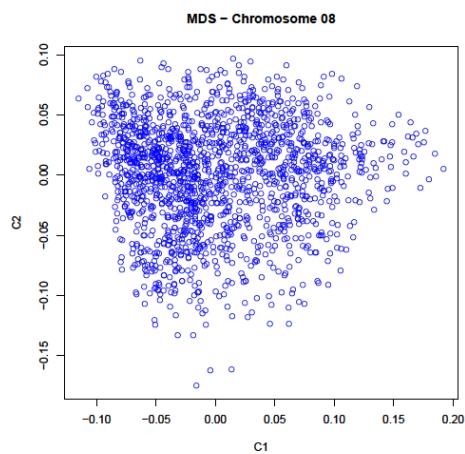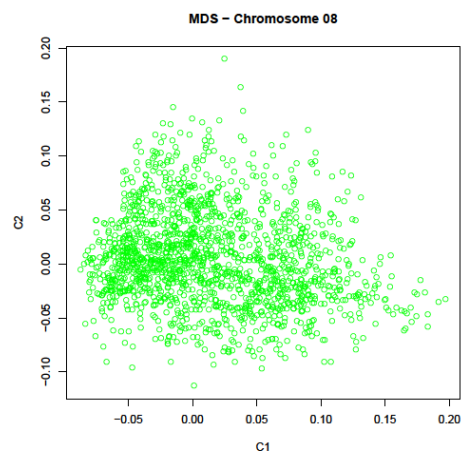

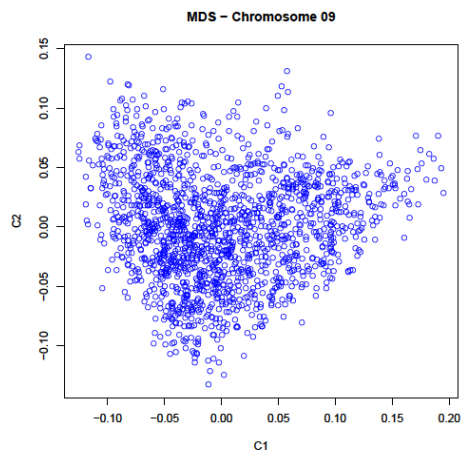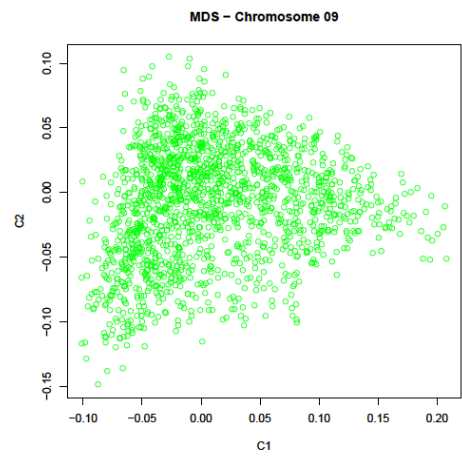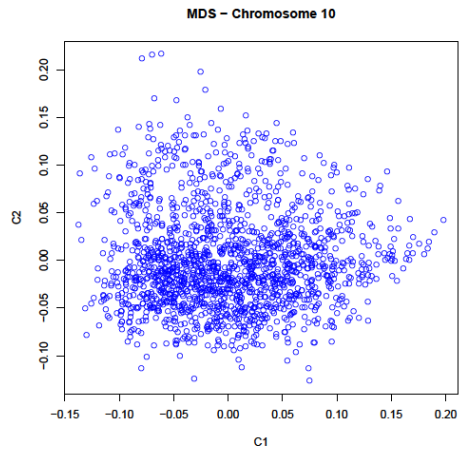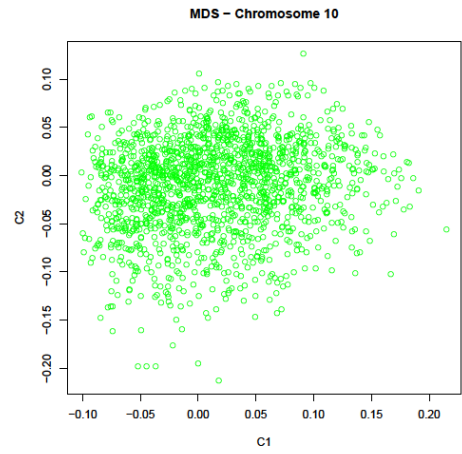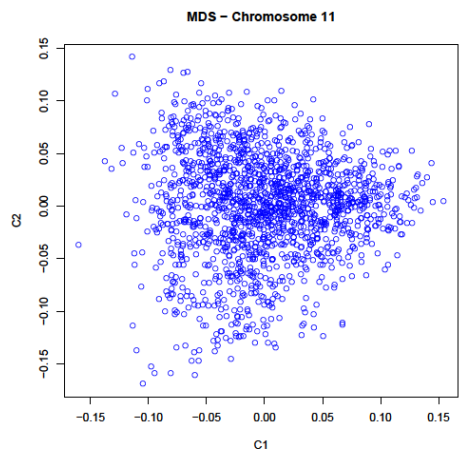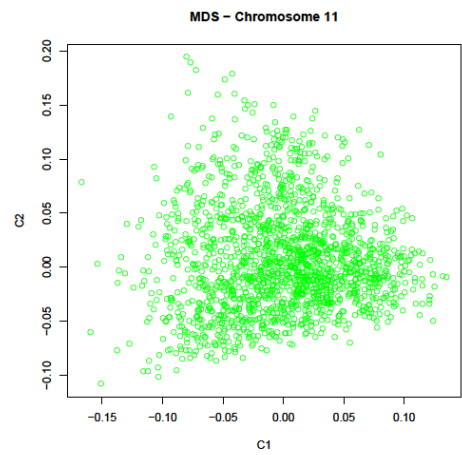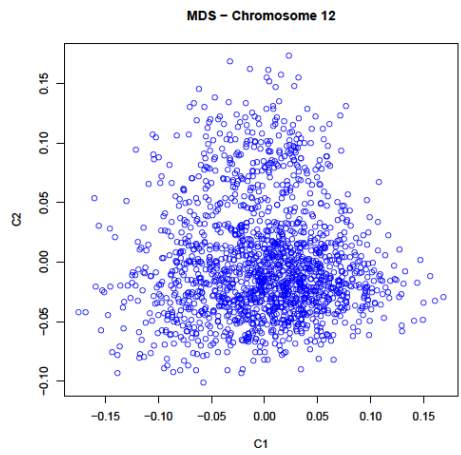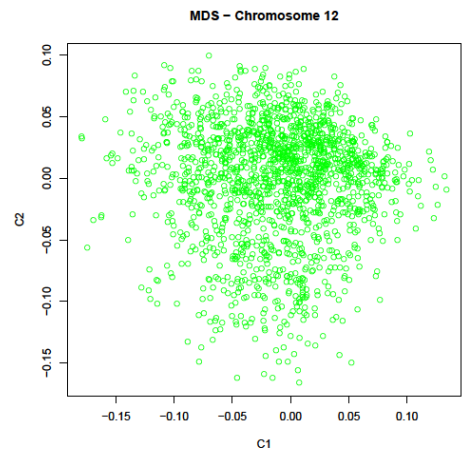

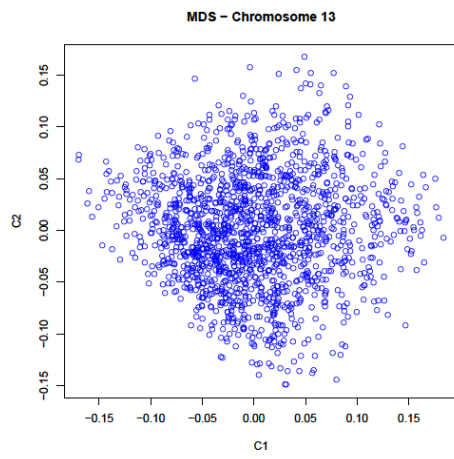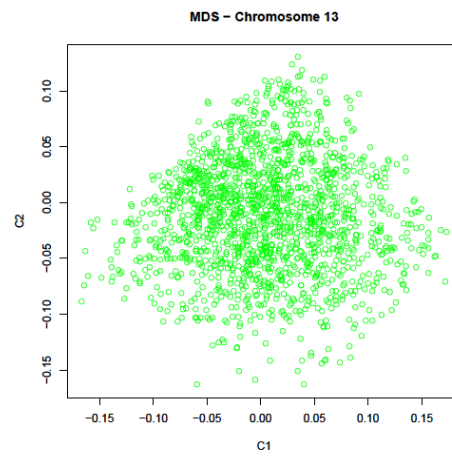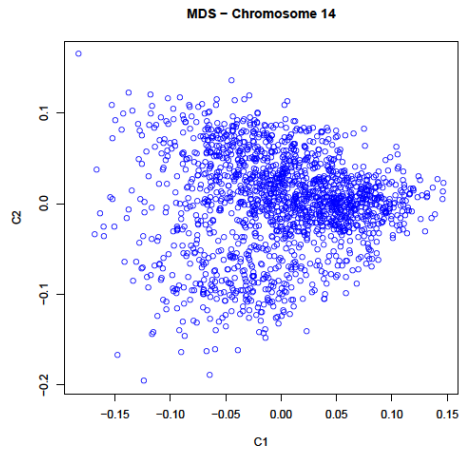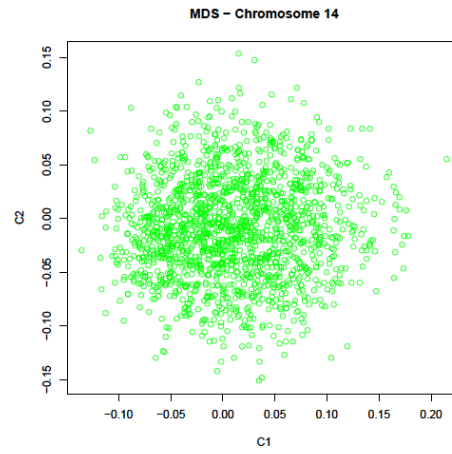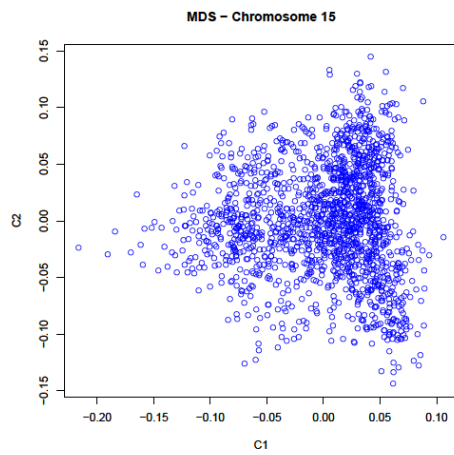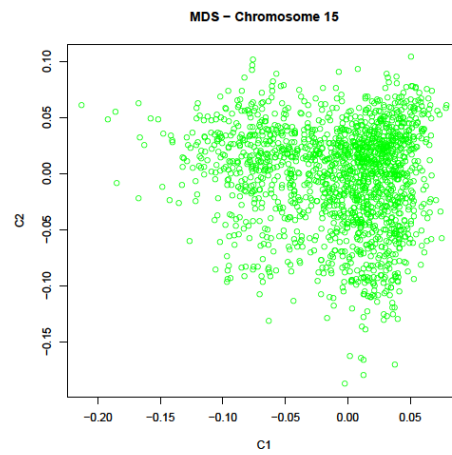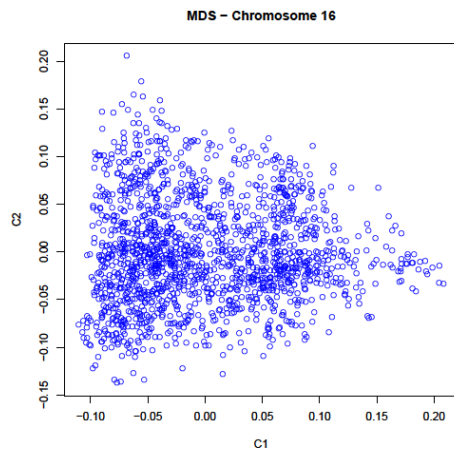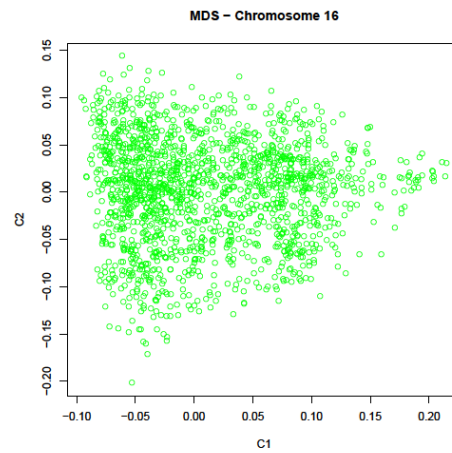

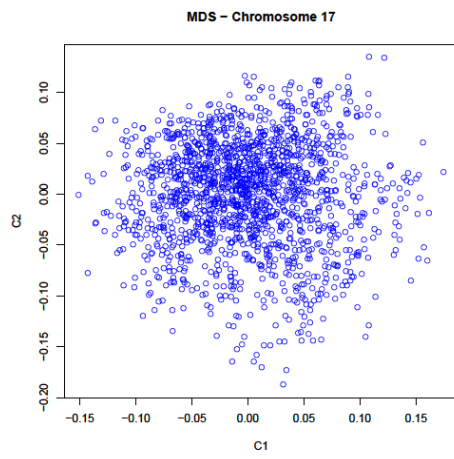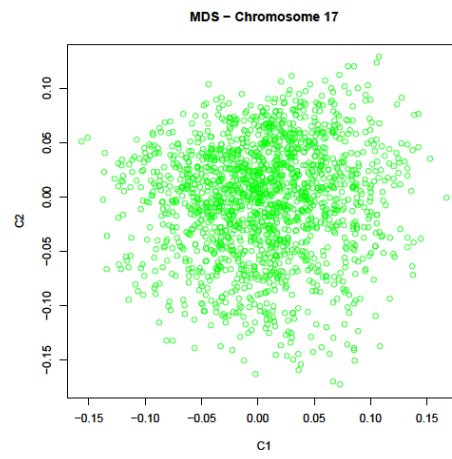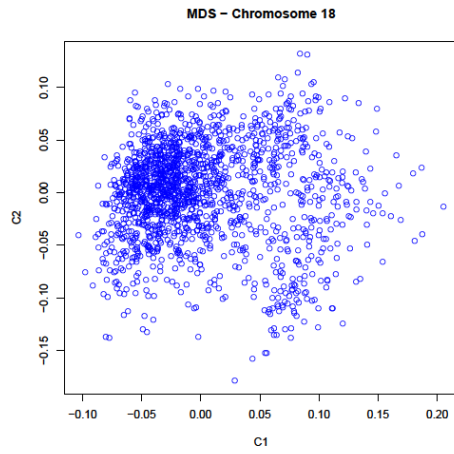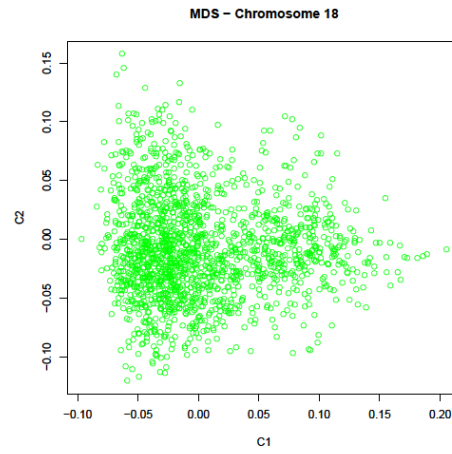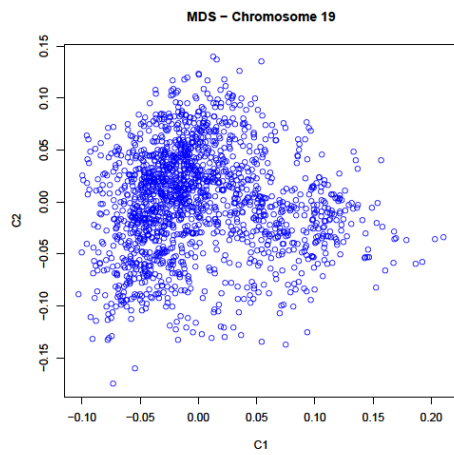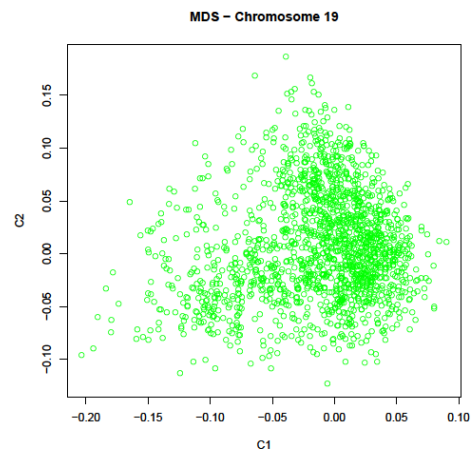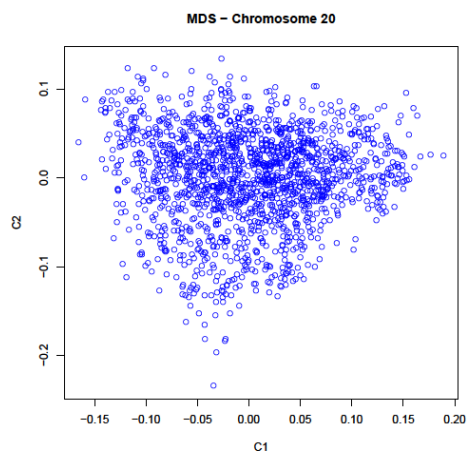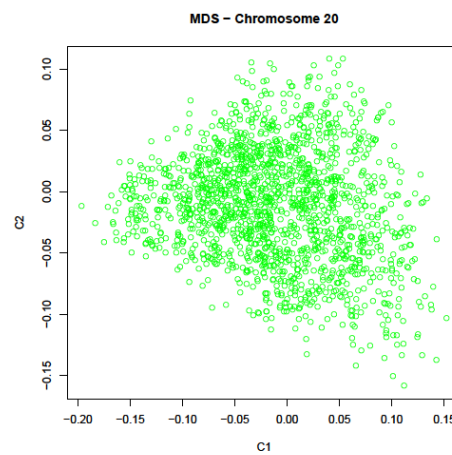

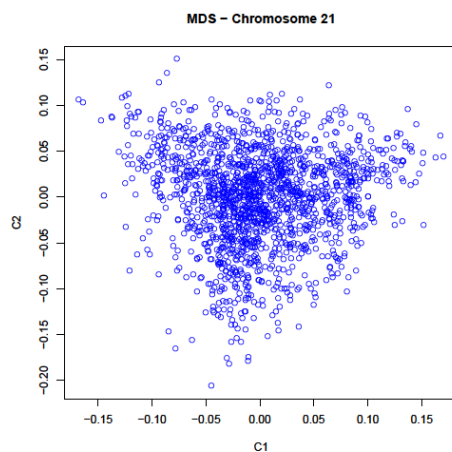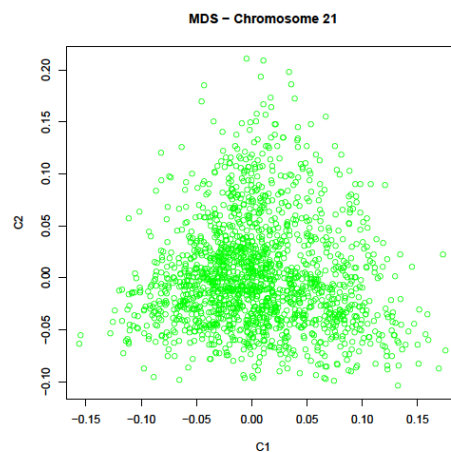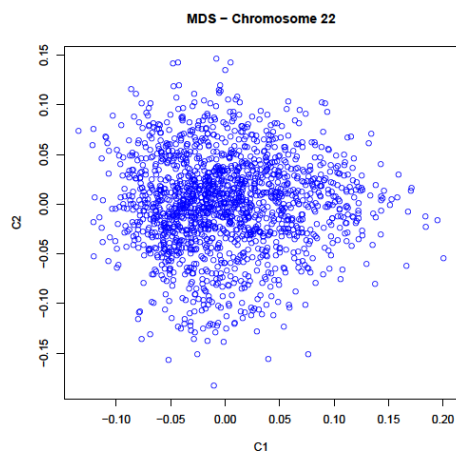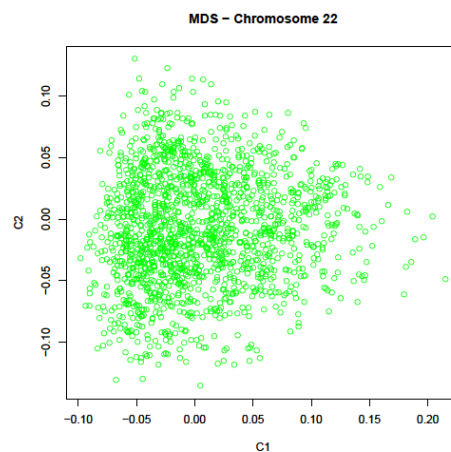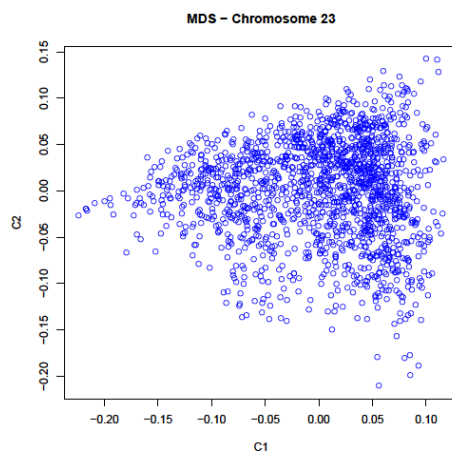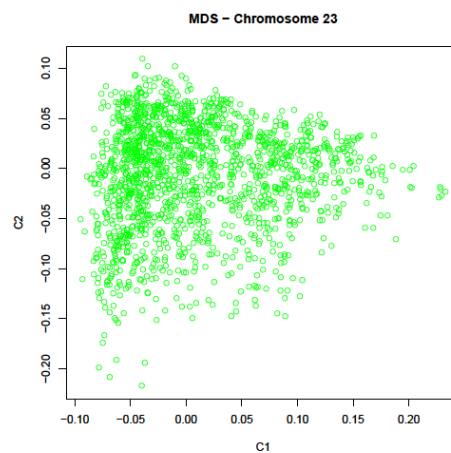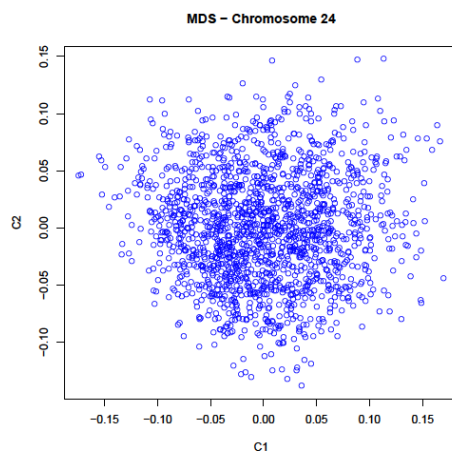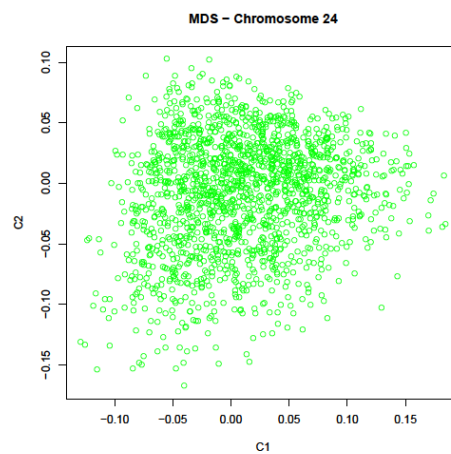

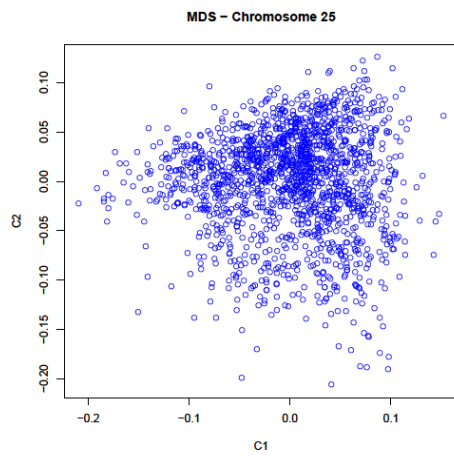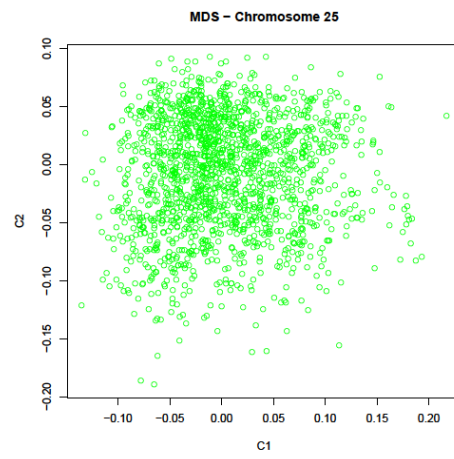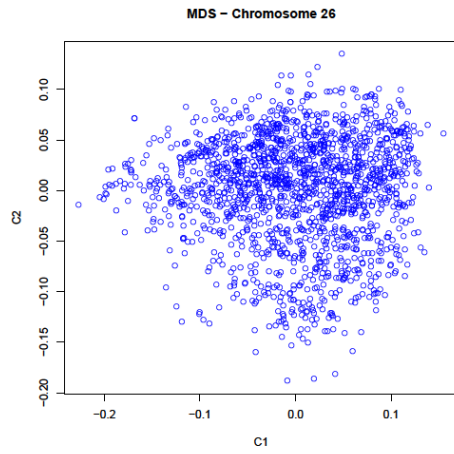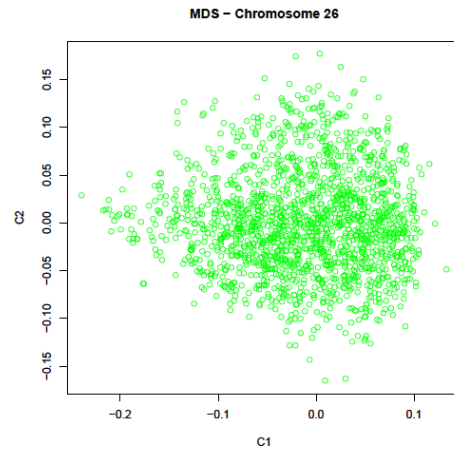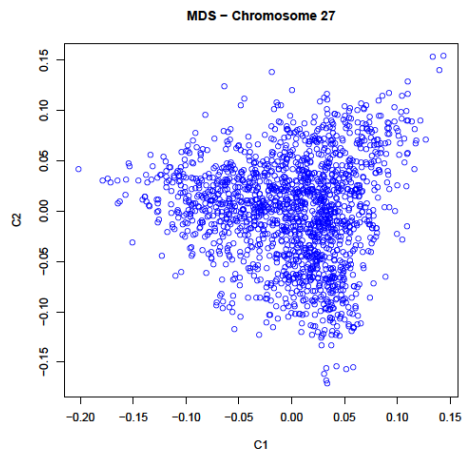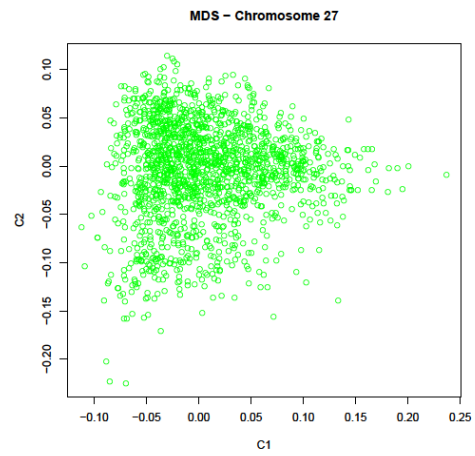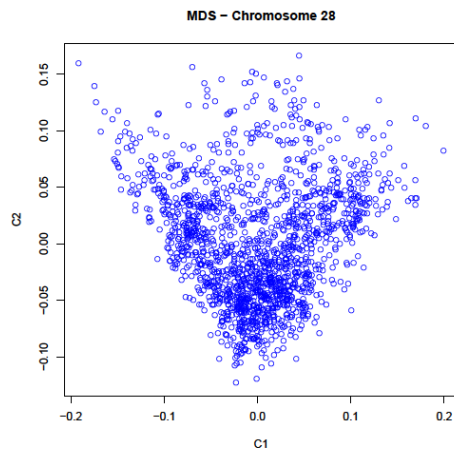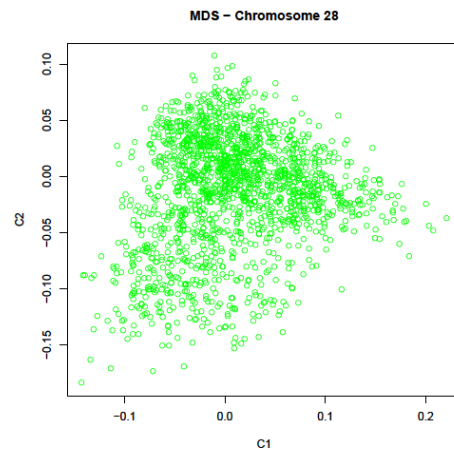

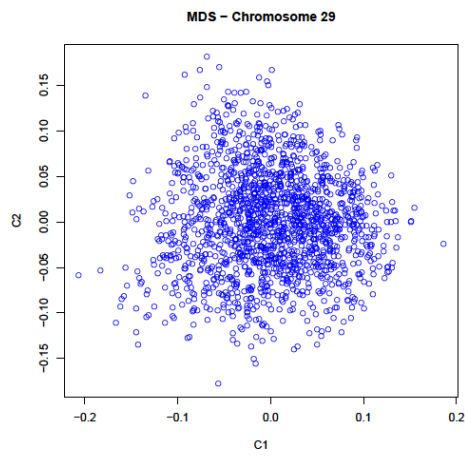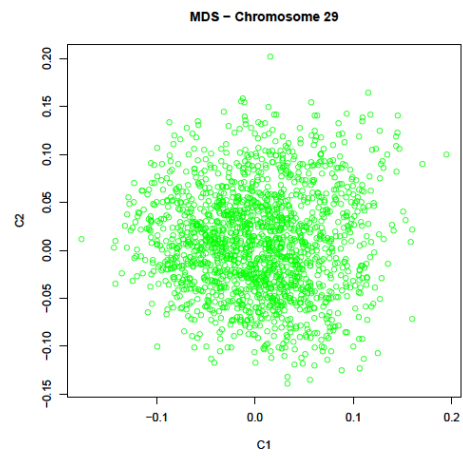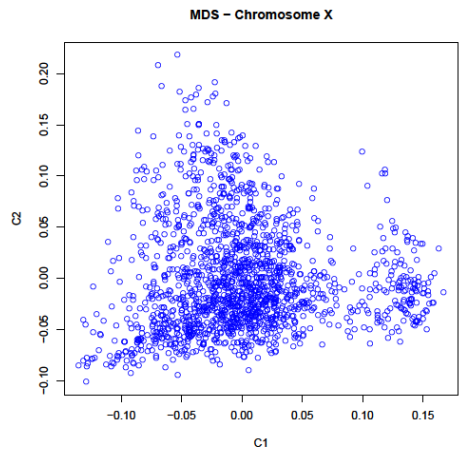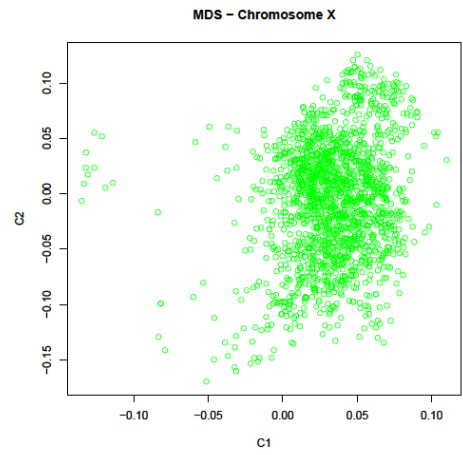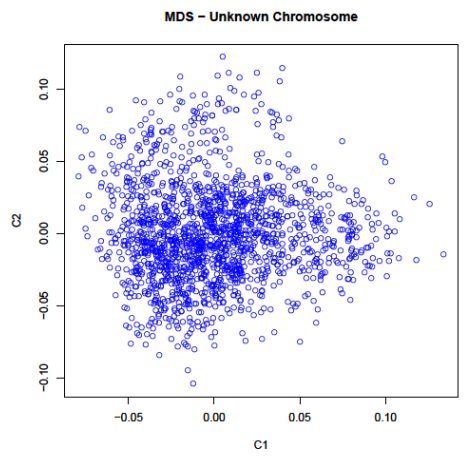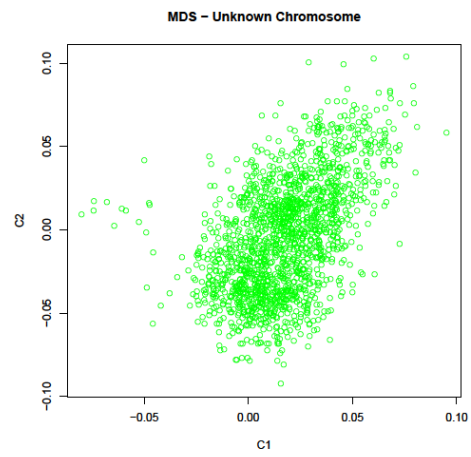

Supplement: Additional file 1 — Figure S1. Multidimensional scaling (MDS) plots of SNP genotypes of 1,654 contemporary Holstein cows by chromosome. C1 = dimension 1, C2 = dimension 2. Left column: C1 and C2 values were calculated using 1,654 contemporary cows. Right column: C1 and C2 values were calculated using 2,366 Holstein cattle, including the University of Minnesota Holstein control line that remained unselected since 1964. [file 1471-2164-13-536-S1.pdf]

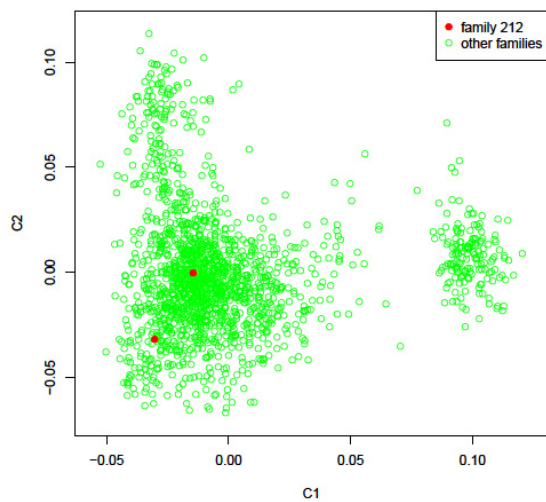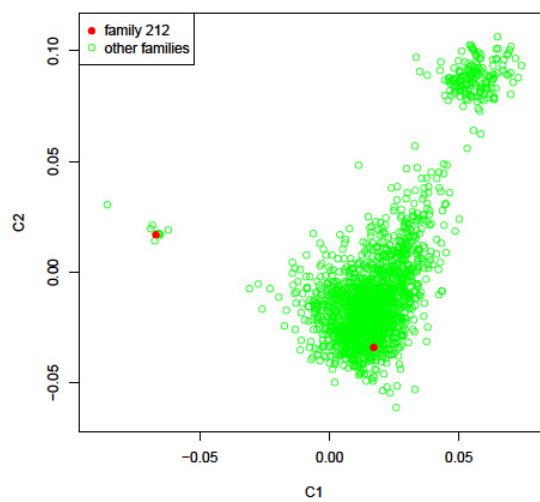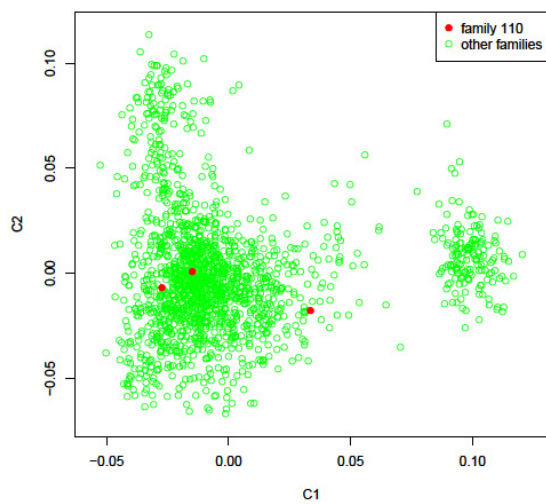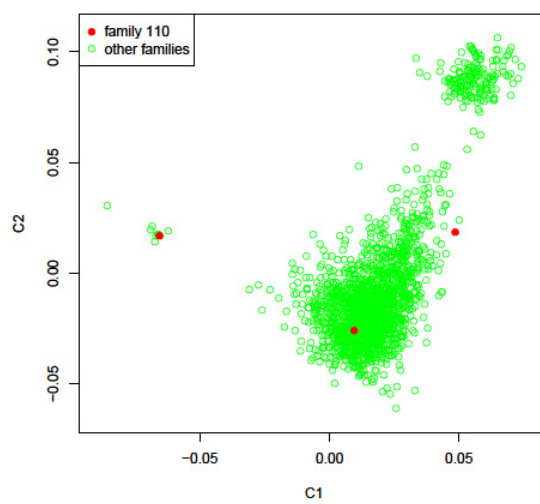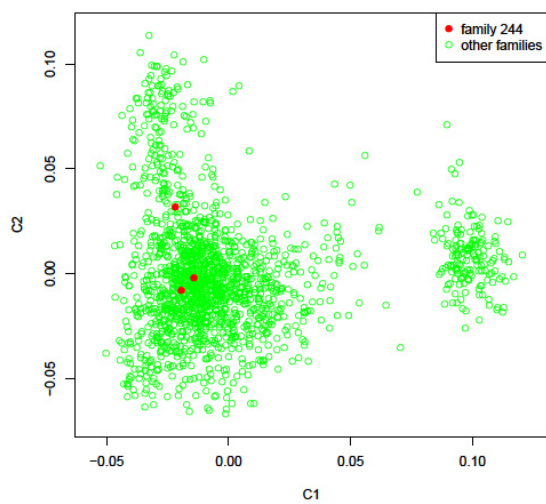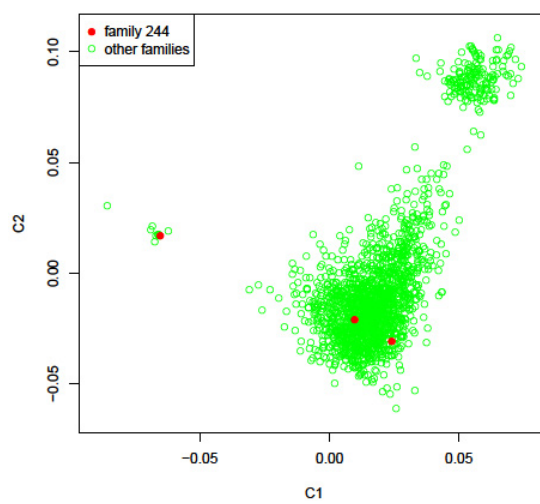

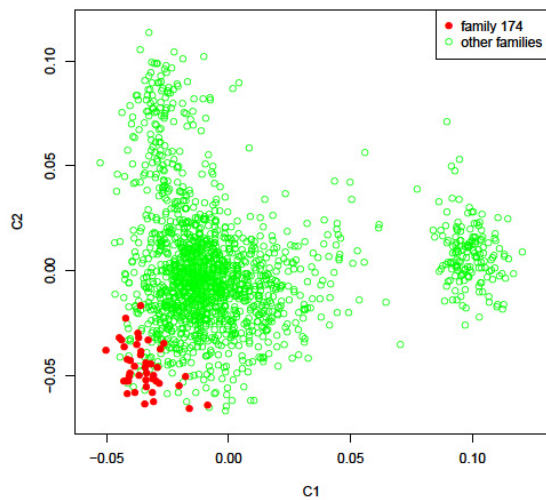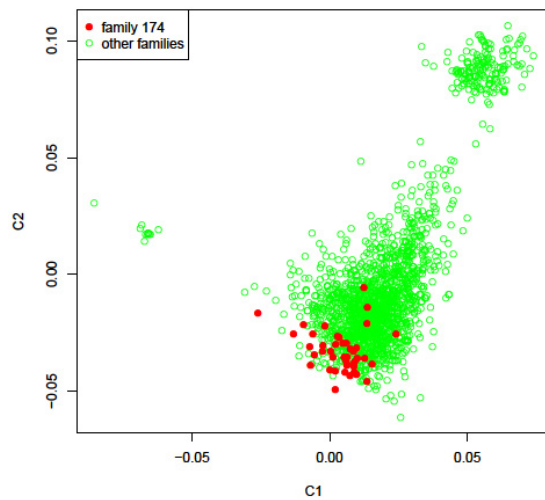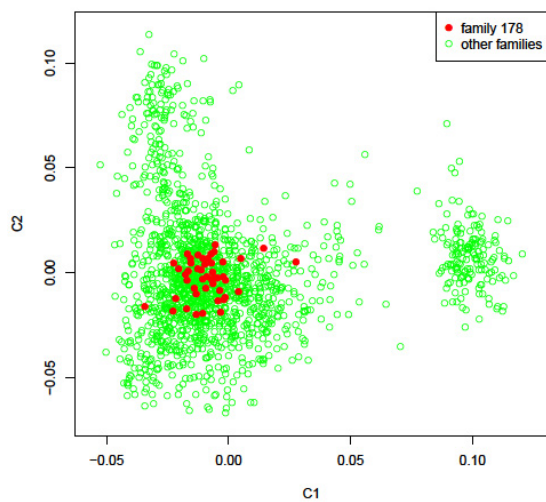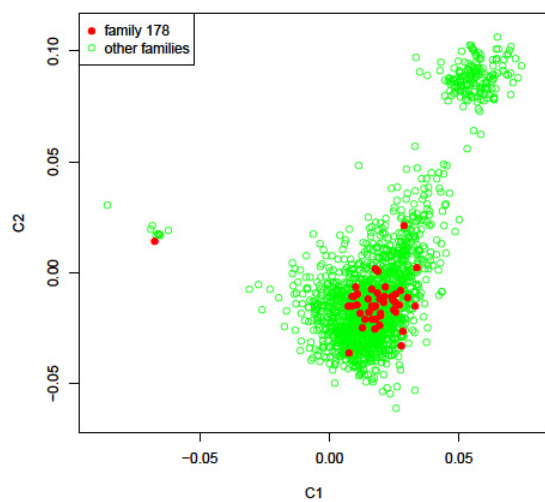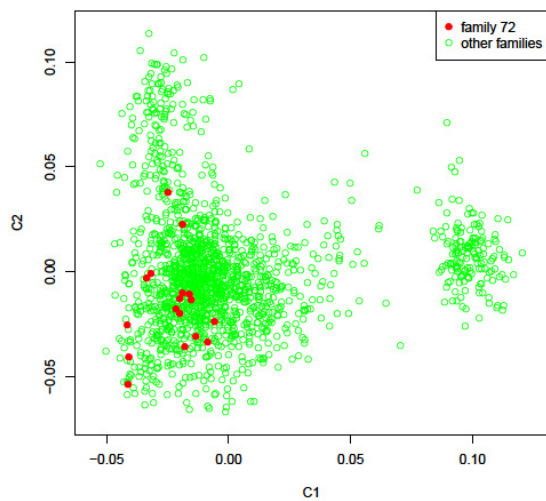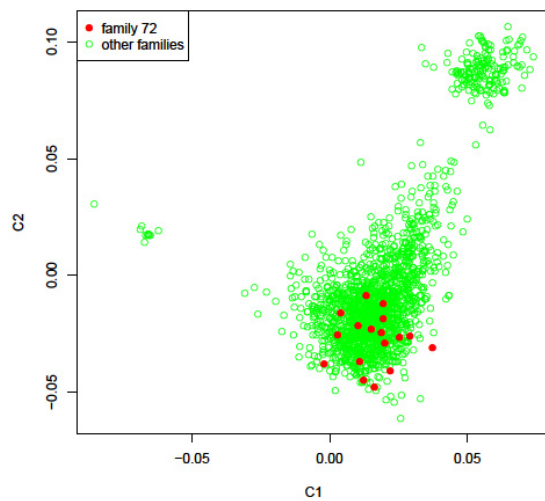

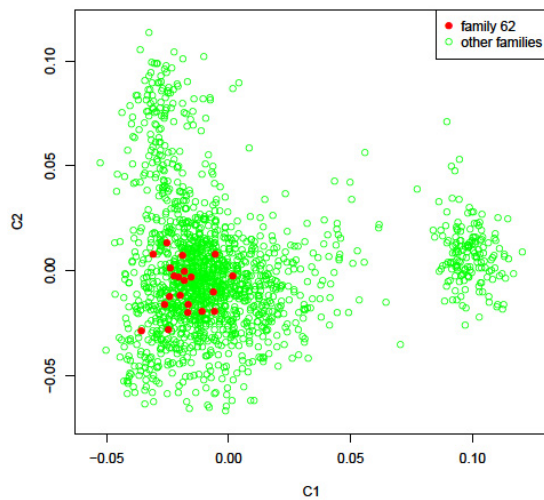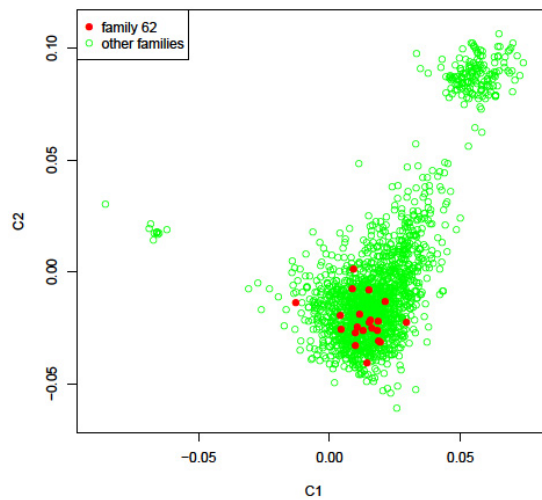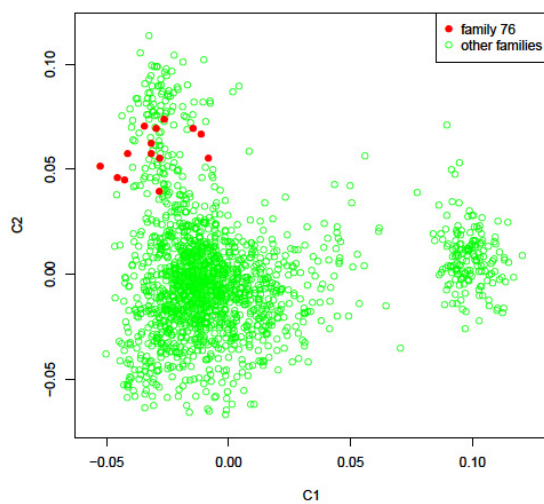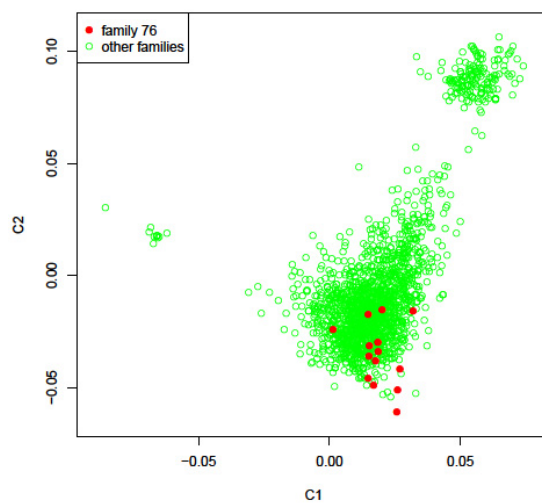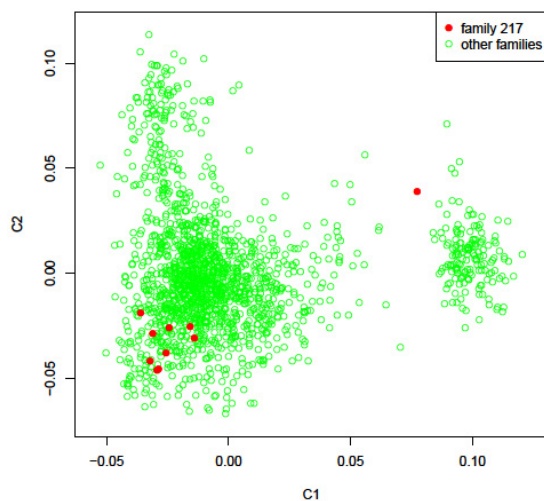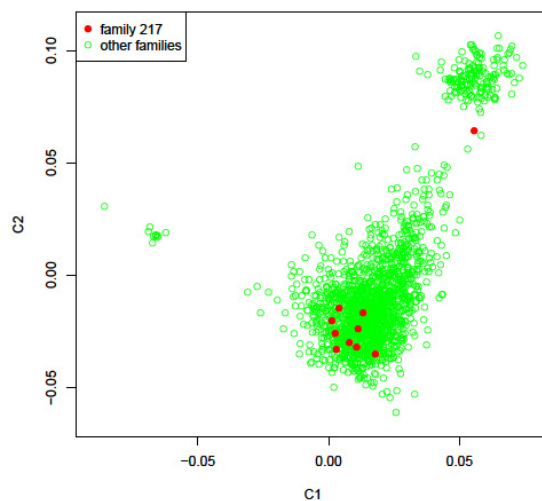

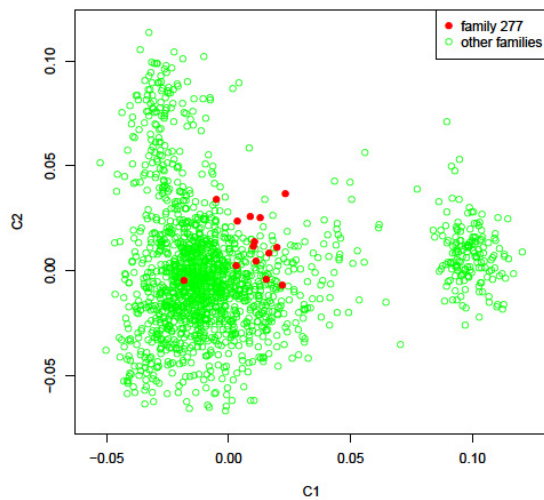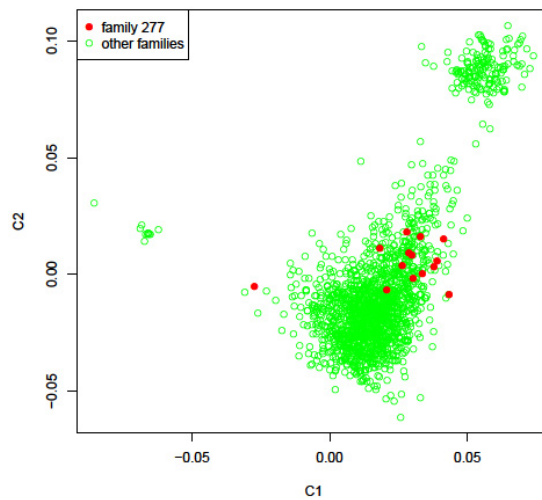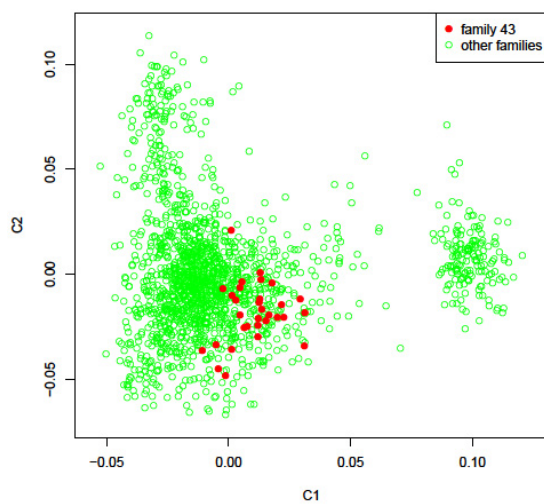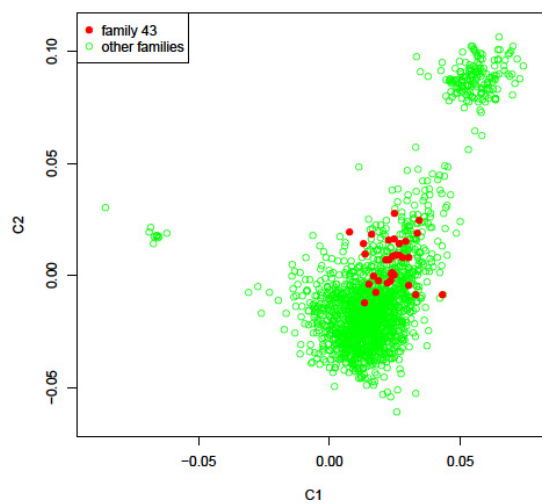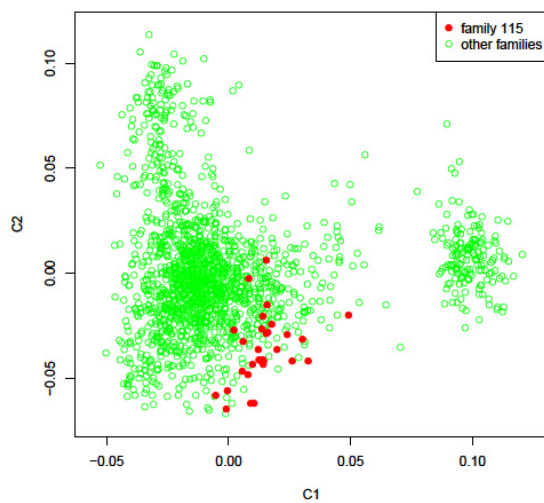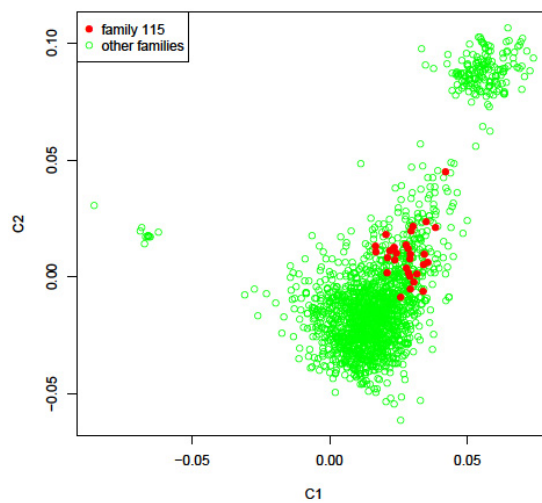

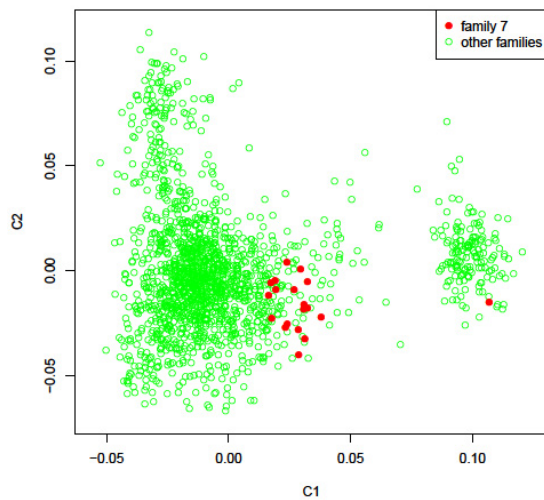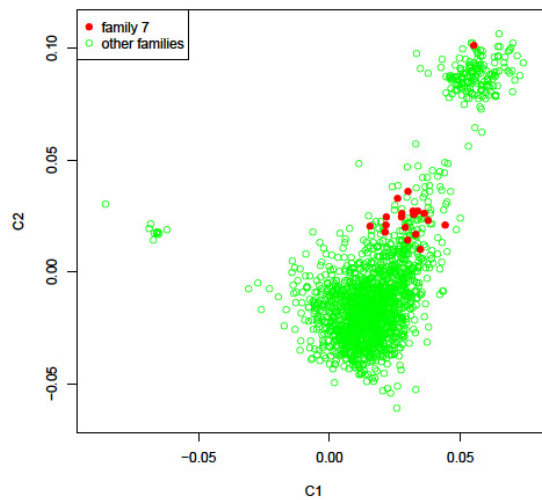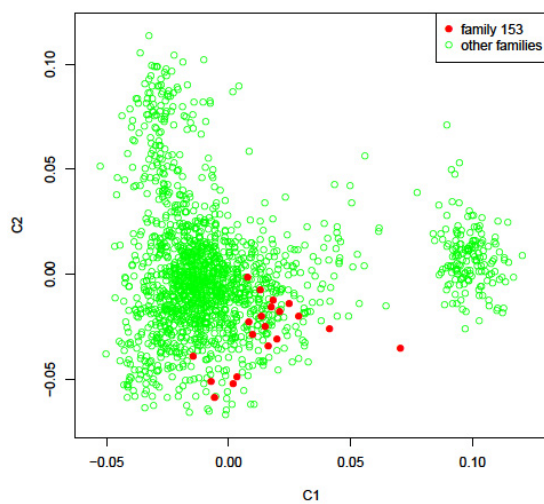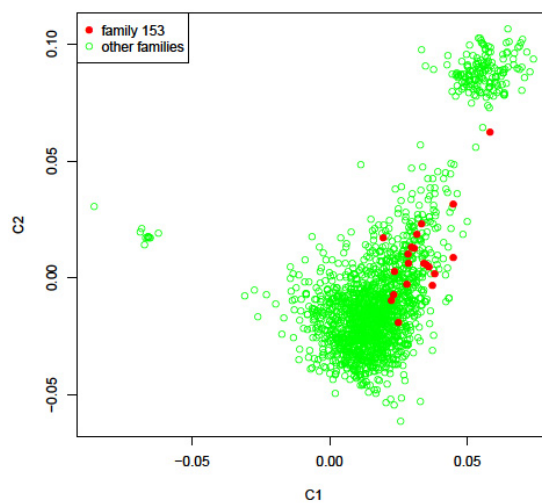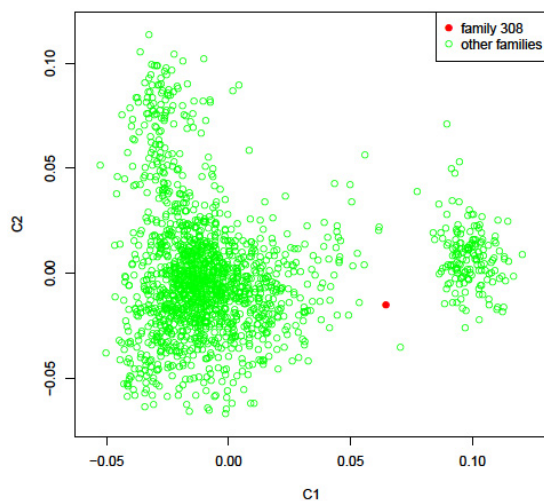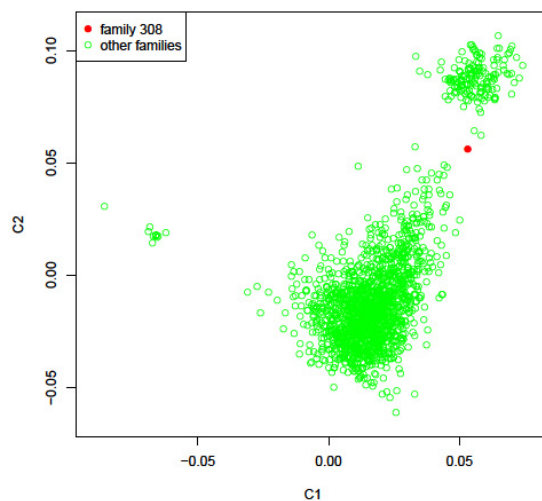

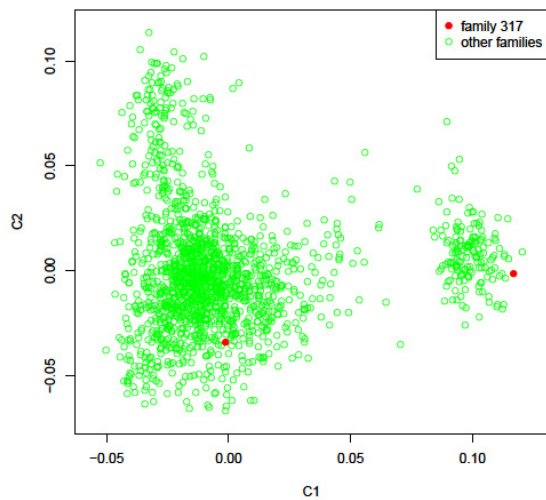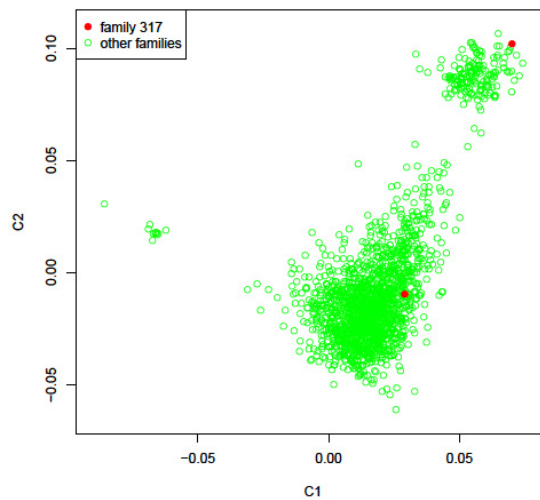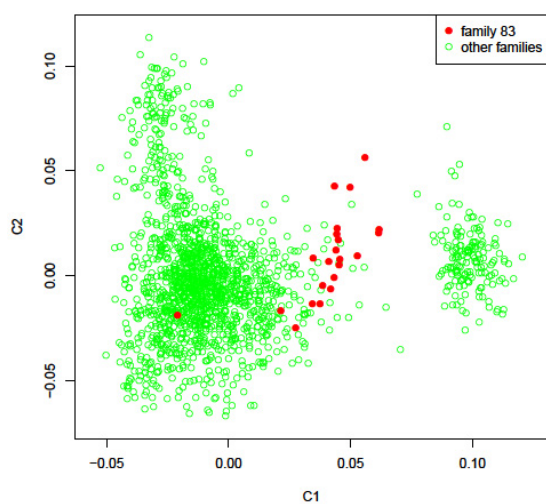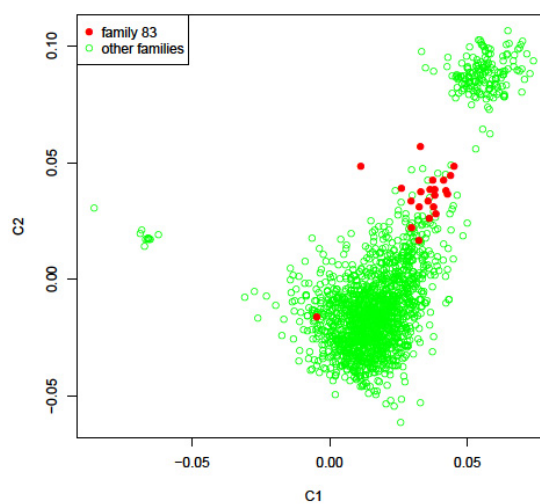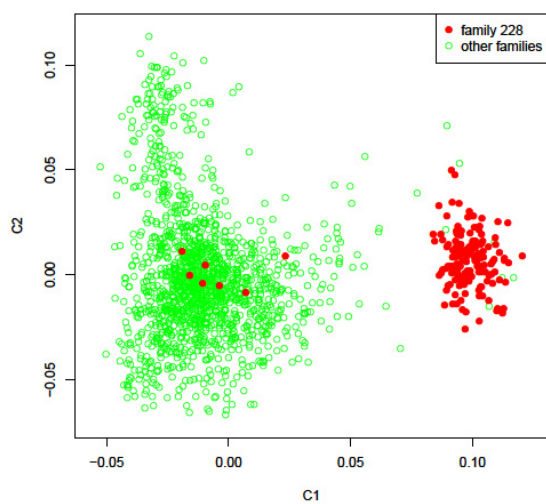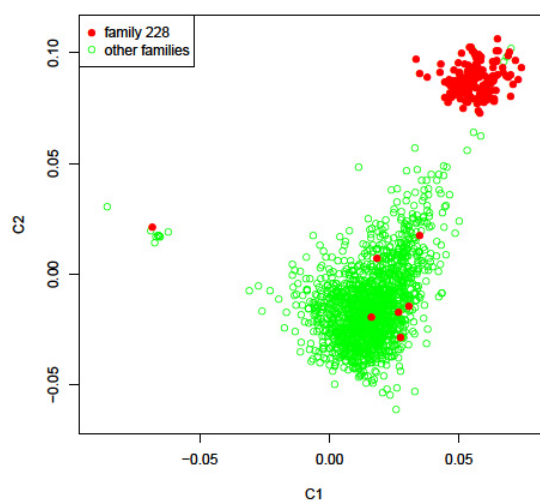

Supplement: Additional file 3 — Figure S3. Overlap between genome stratification and half-sib family structure. C1 = dimension 1, C2 = dimension 2. Left column: C1 and C2 values were calculated using 1,654 contemporary Holstein cows. Right column: C1 and C2 values were calculated using 2,366 Holstein cattle, including the University of Minnesota Holstein control line that remained unselected since 1964. [file 1471-2164-13-536-S3.pdf]

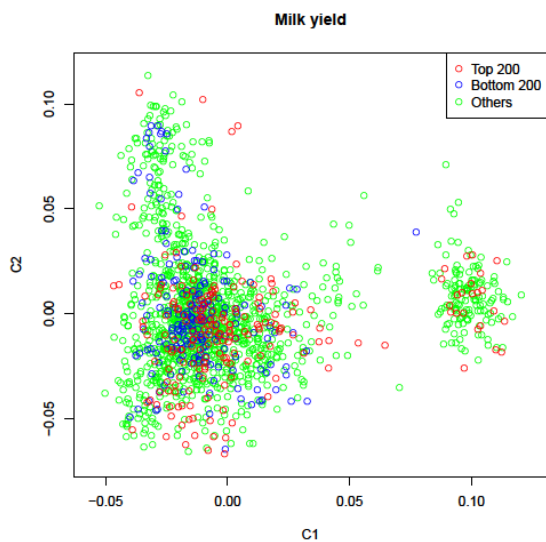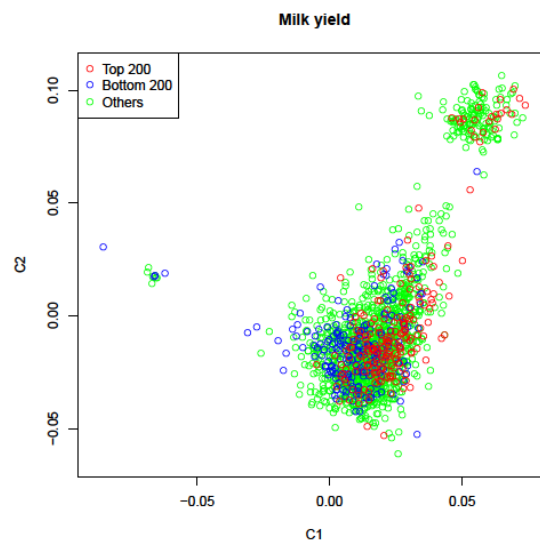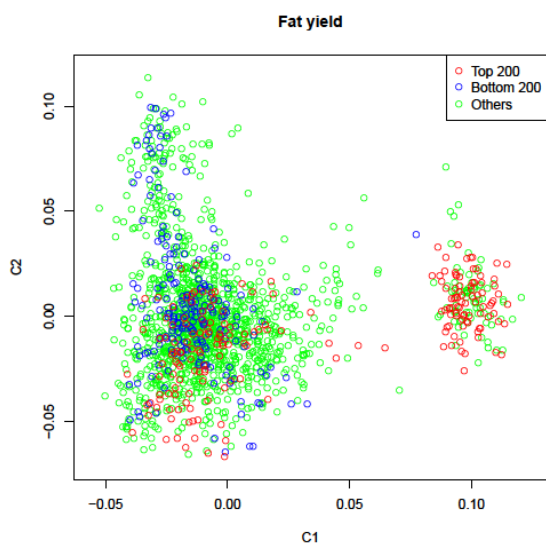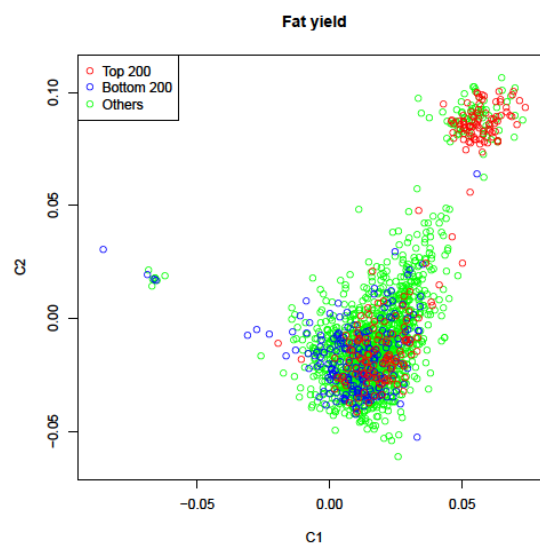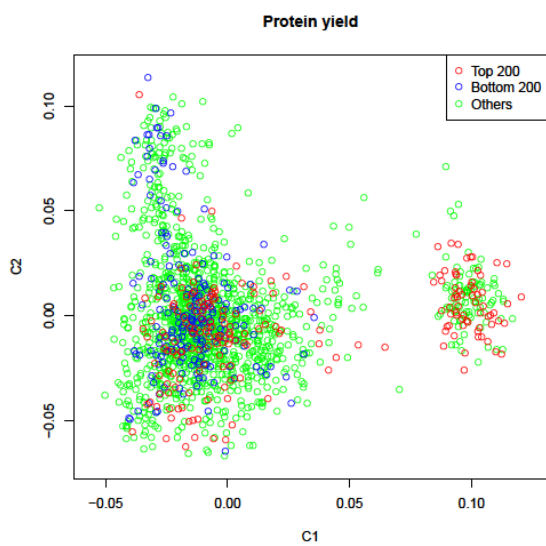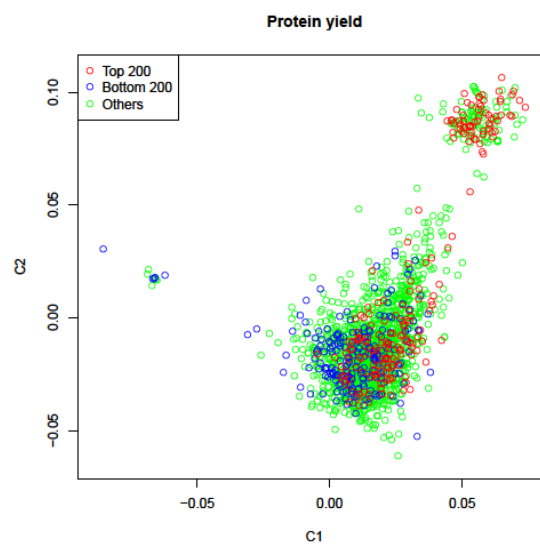

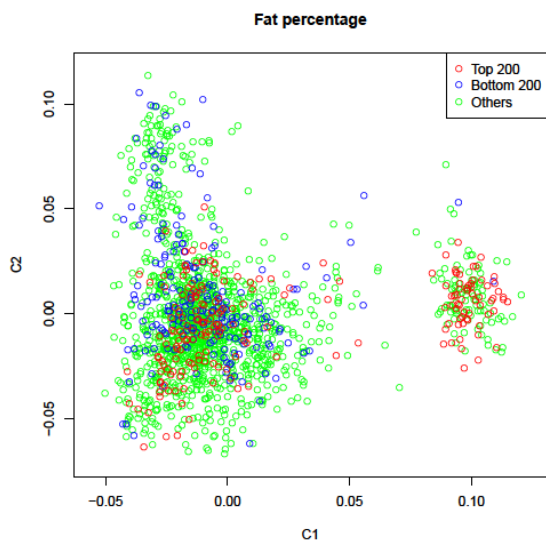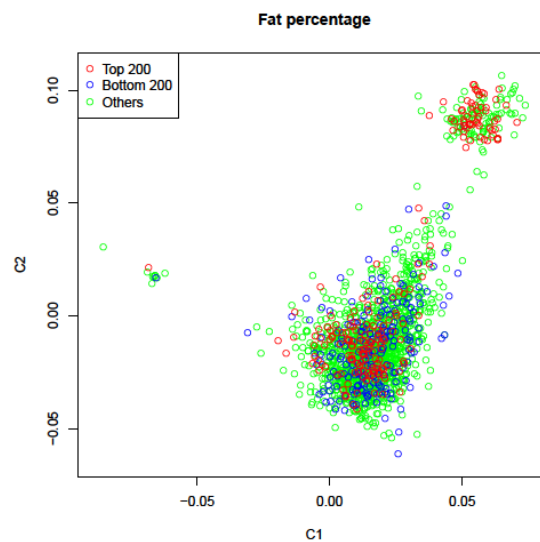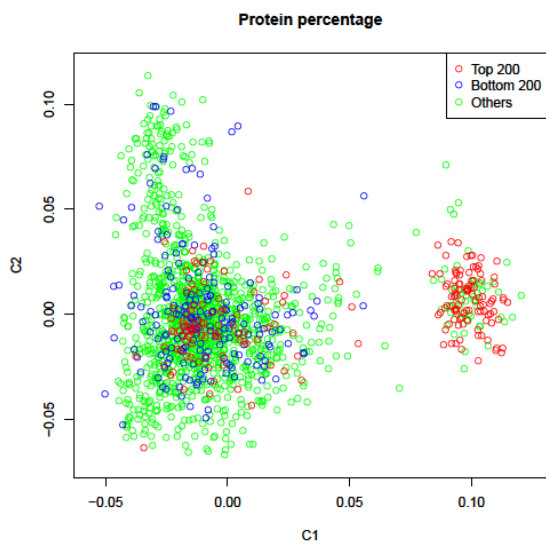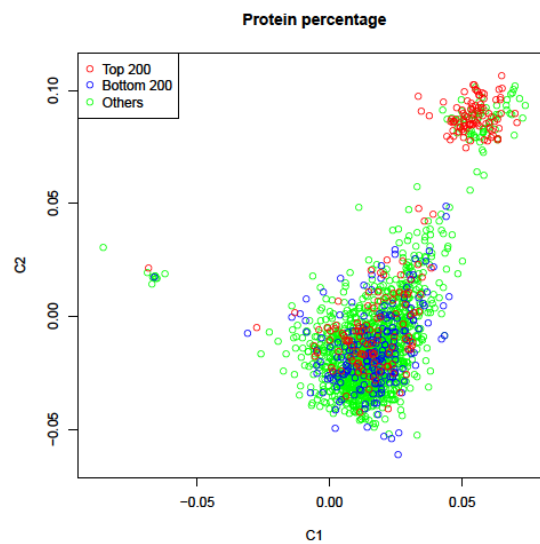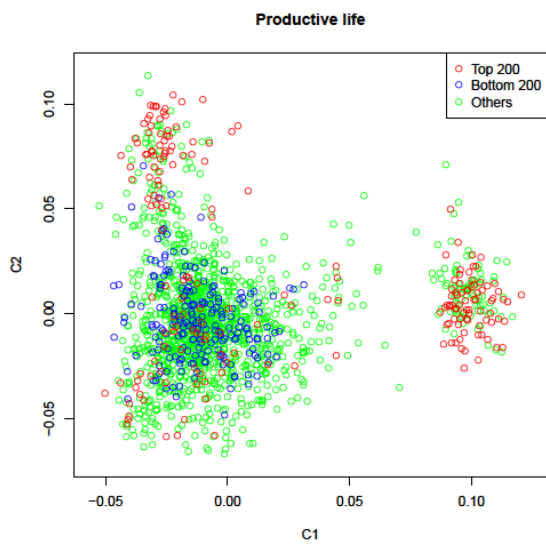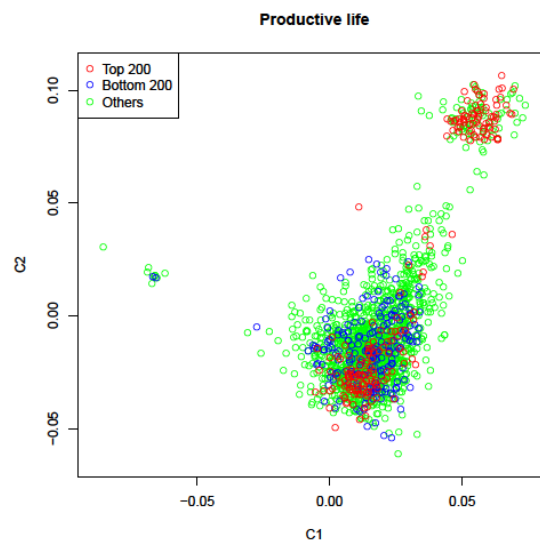

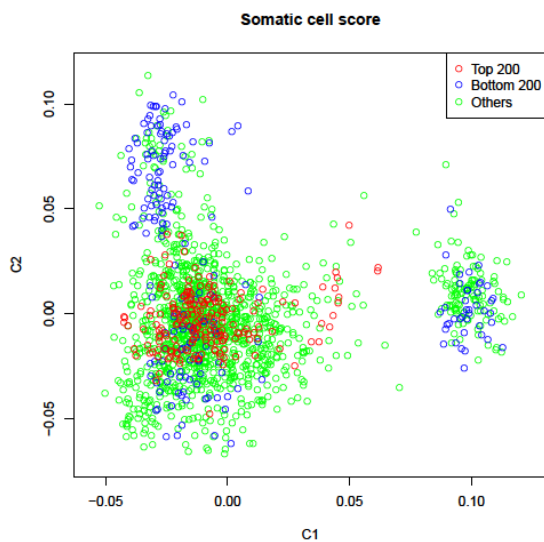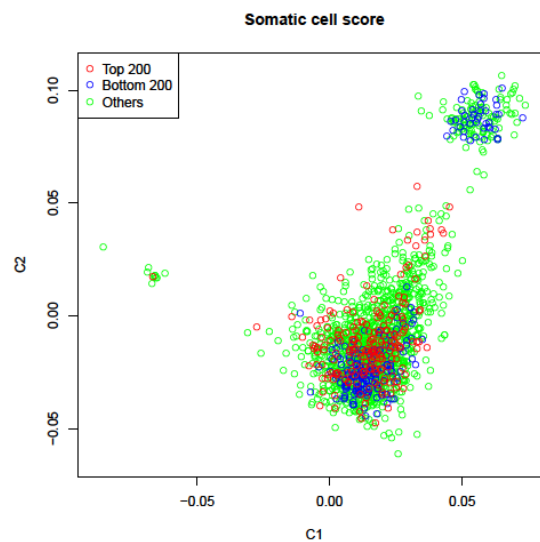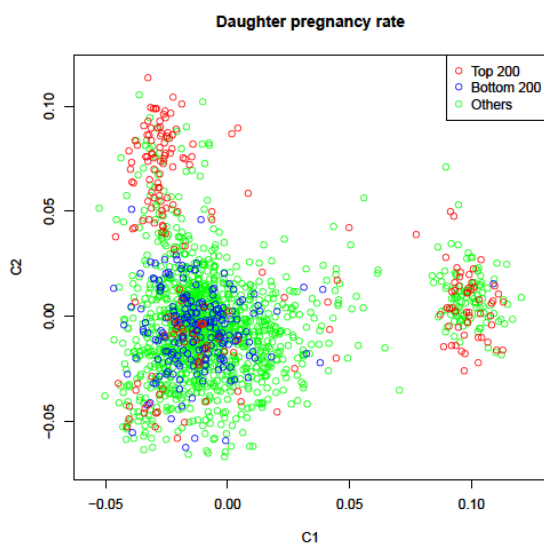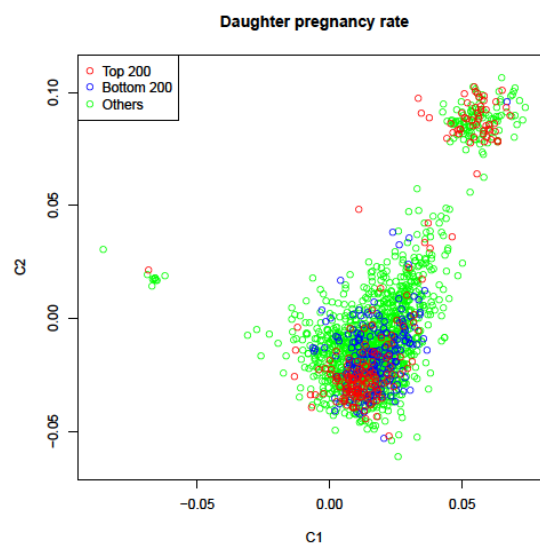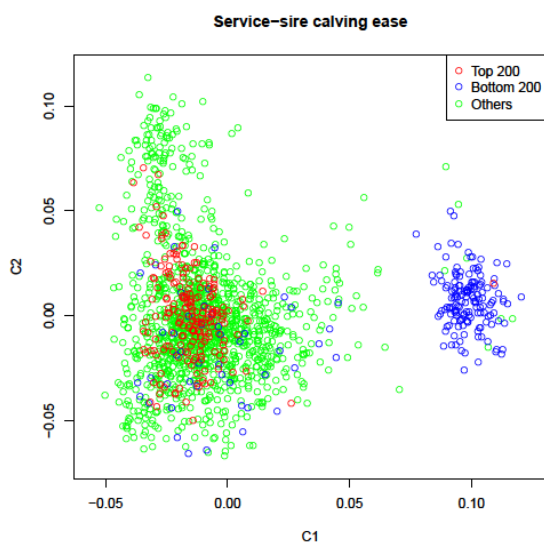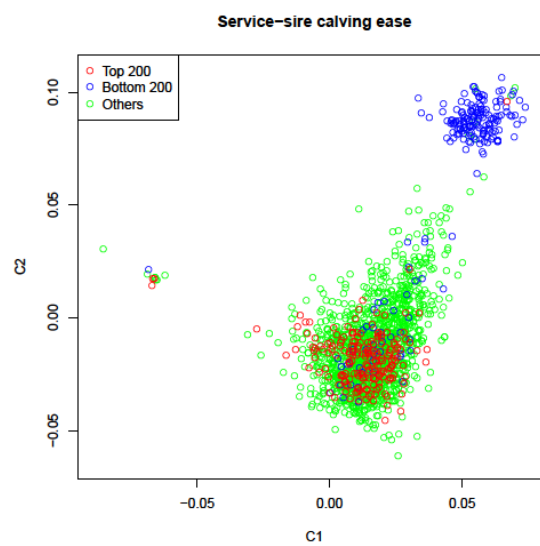

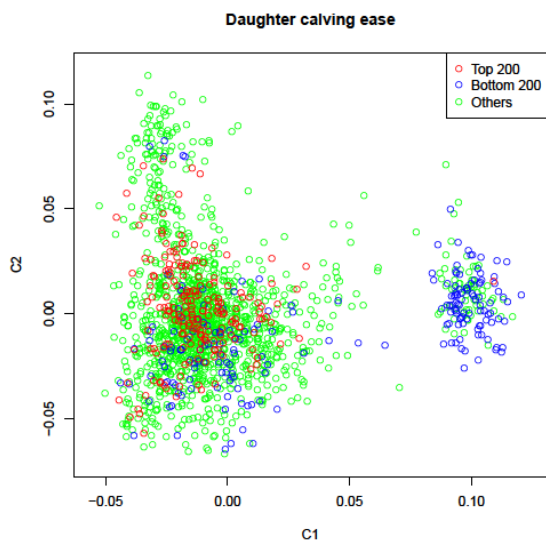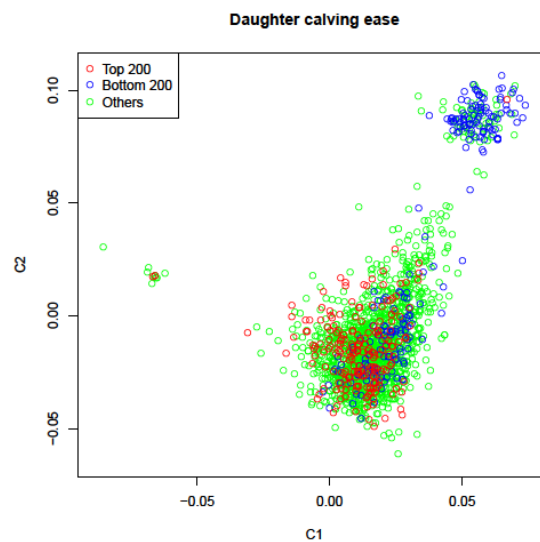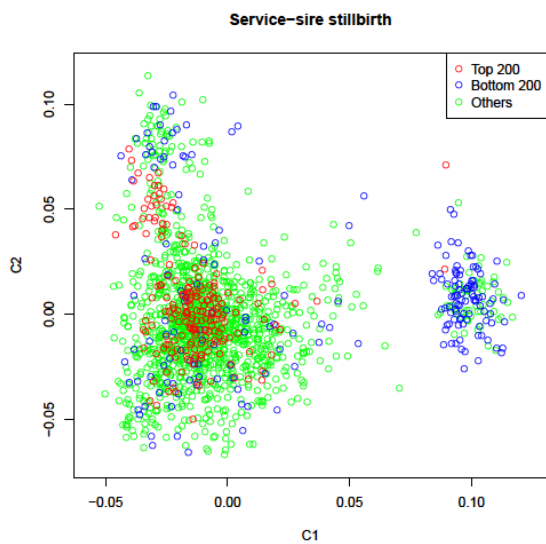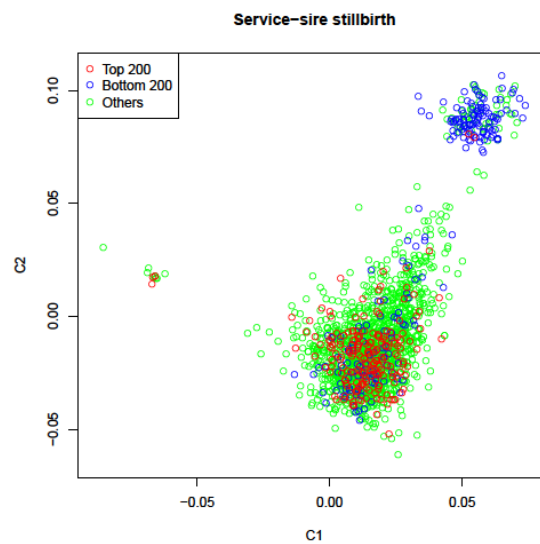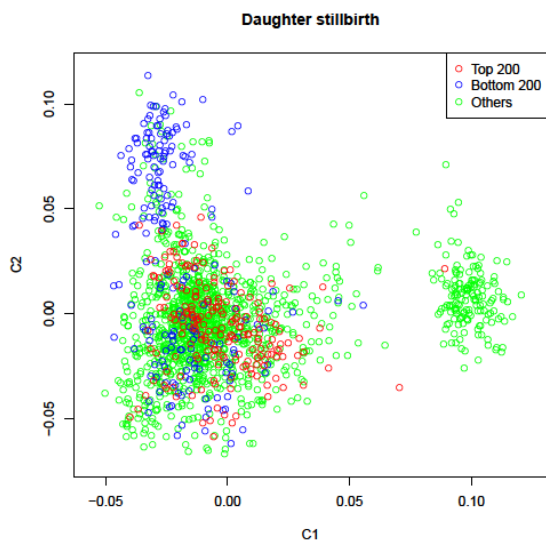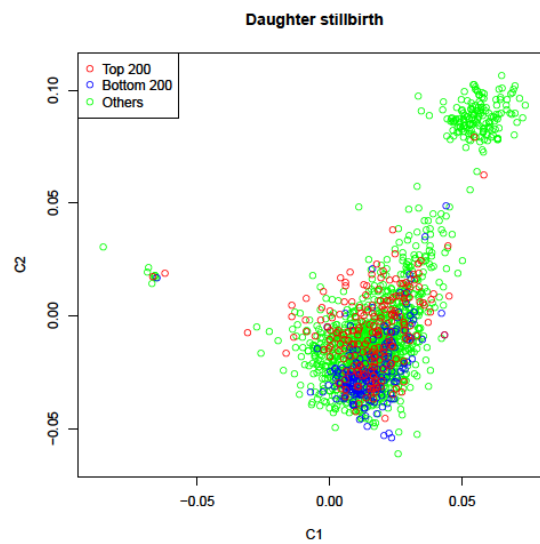

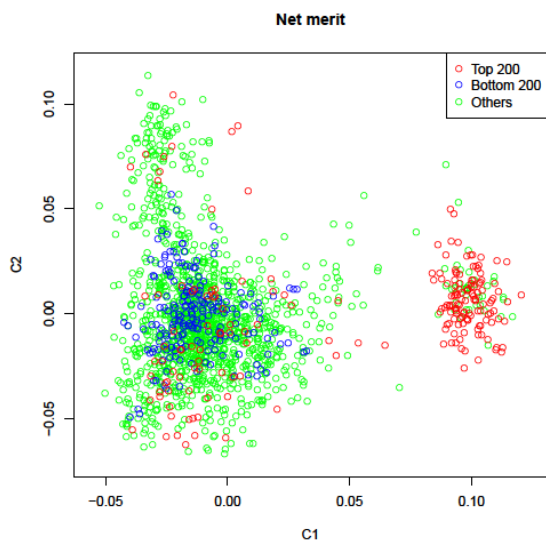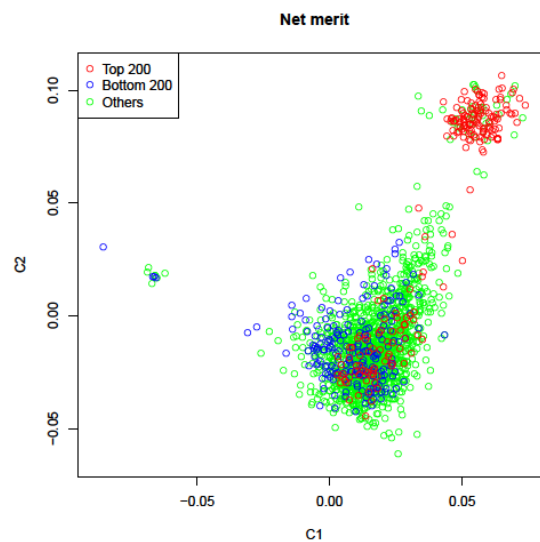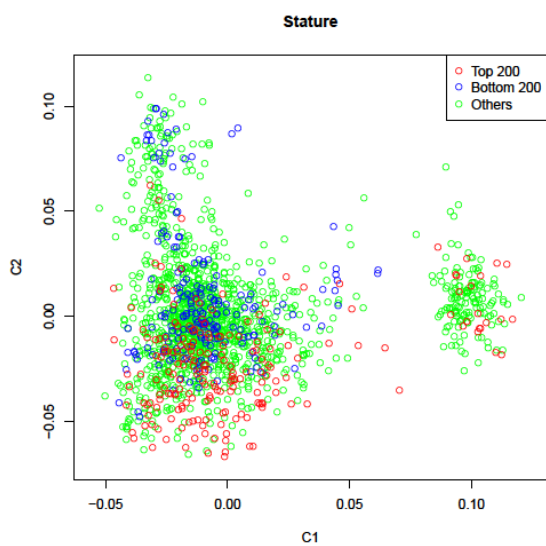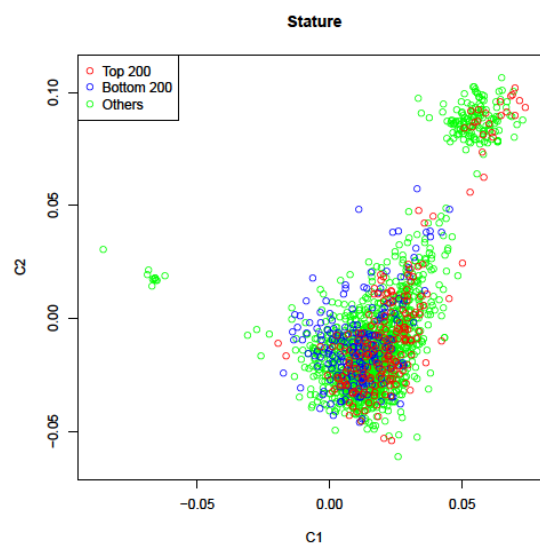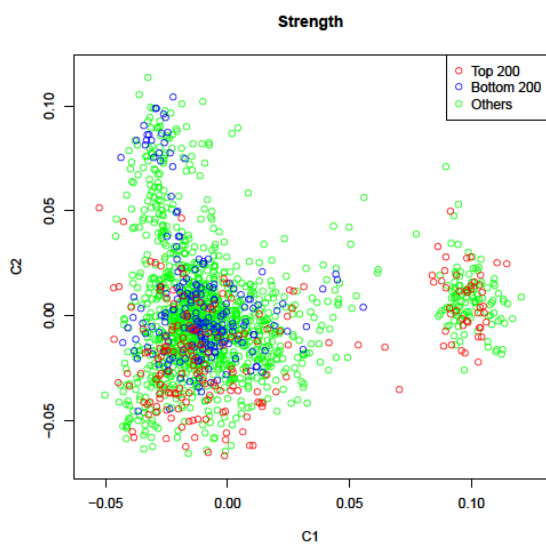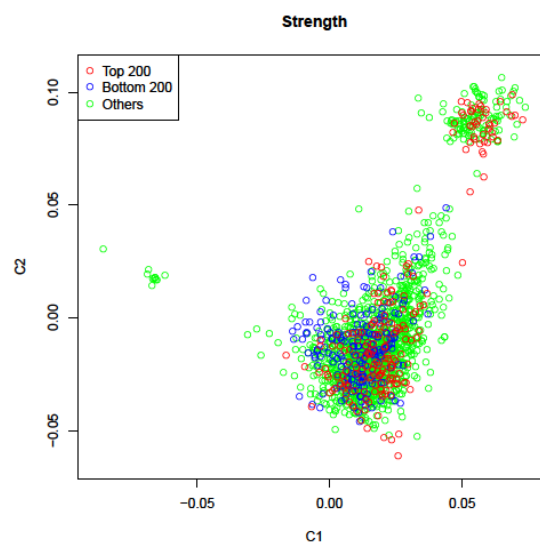

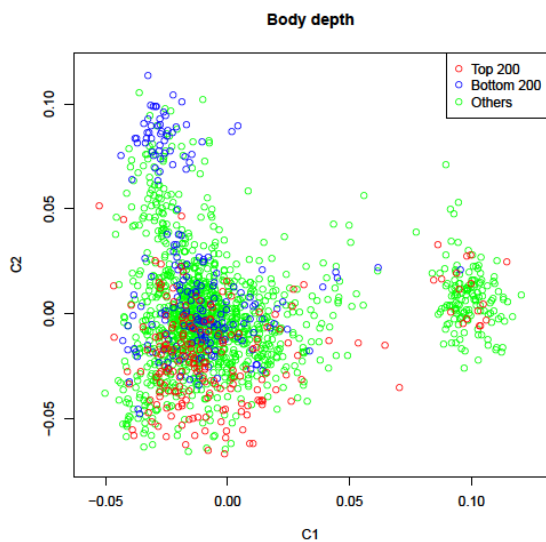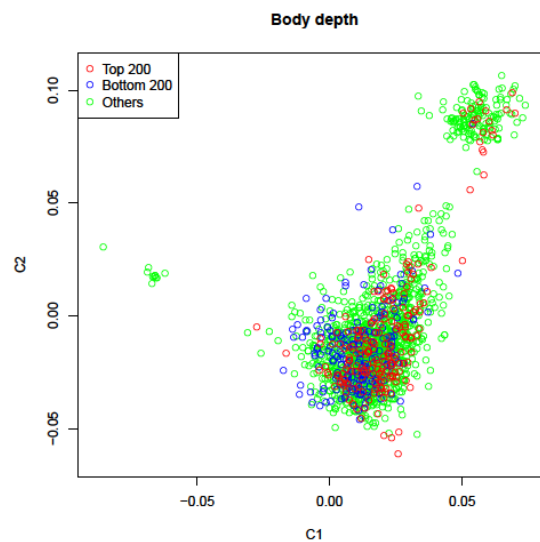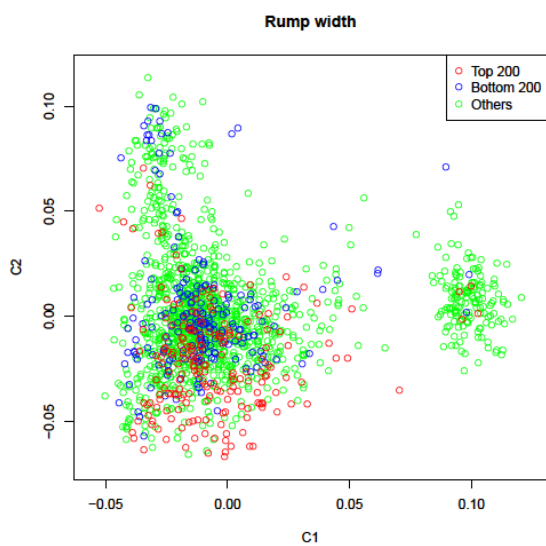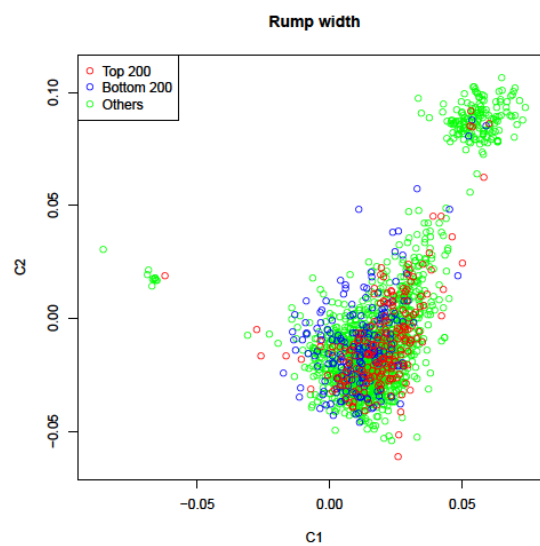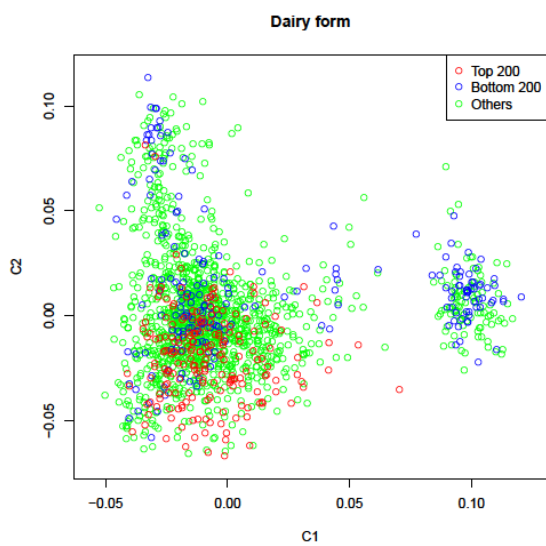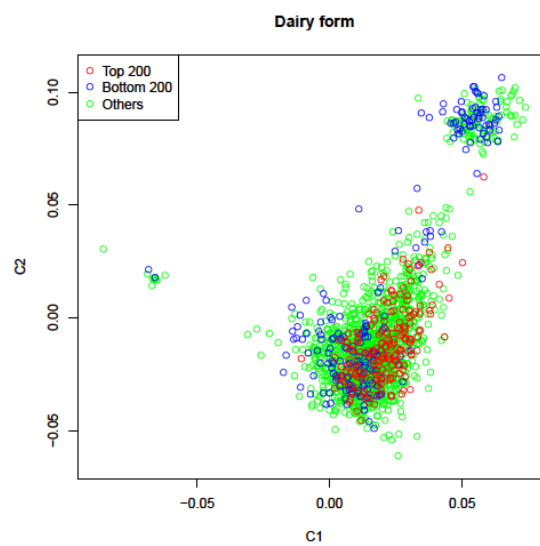

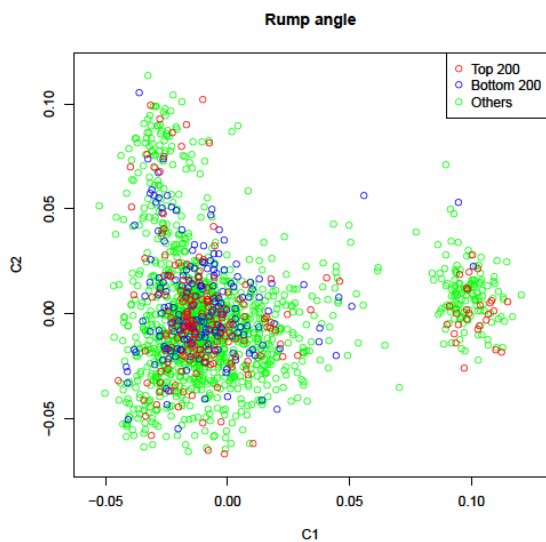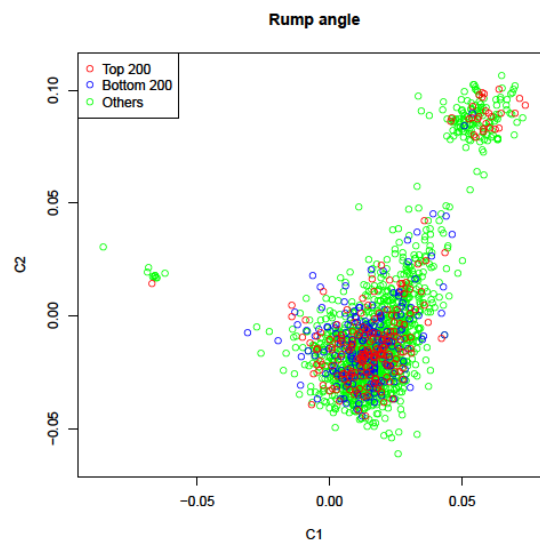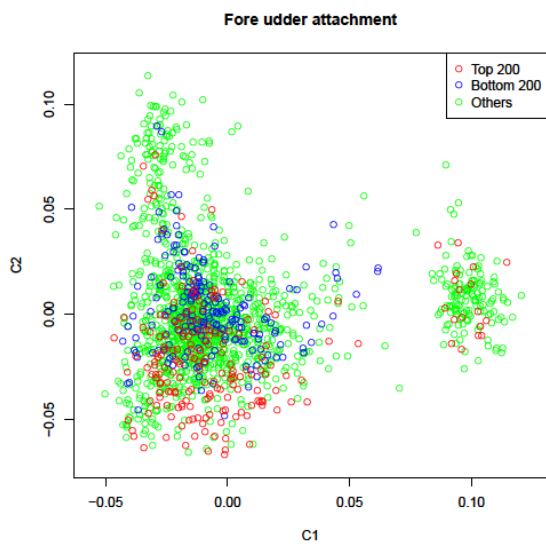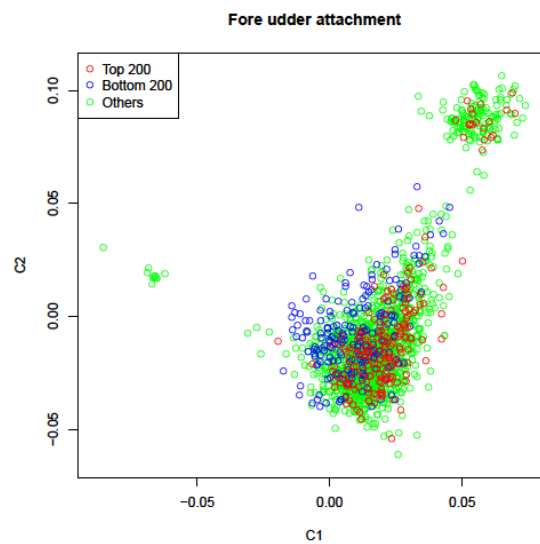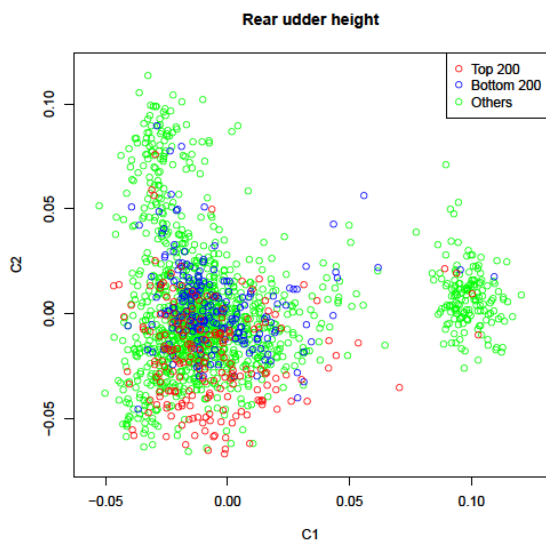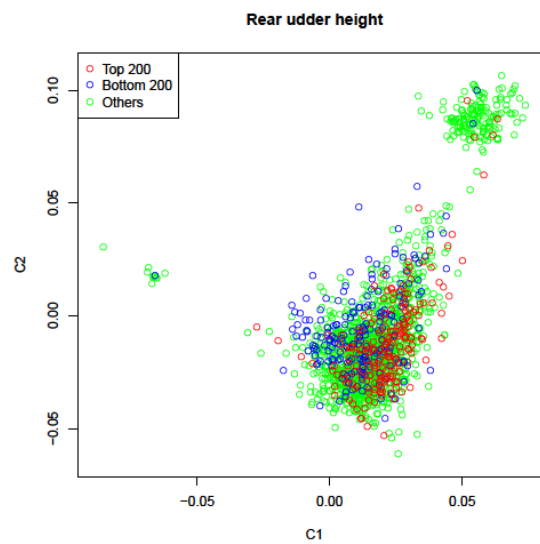

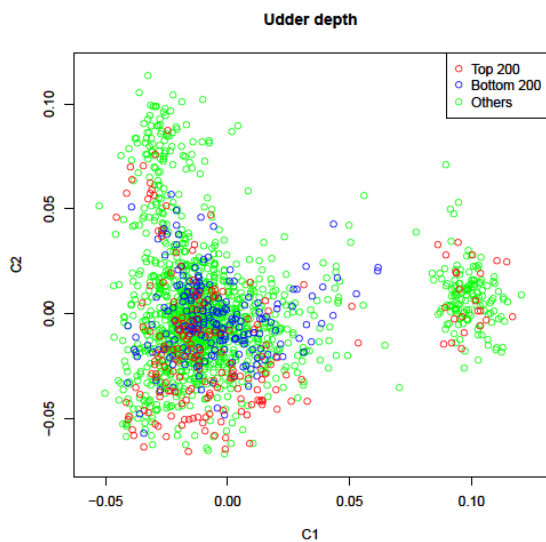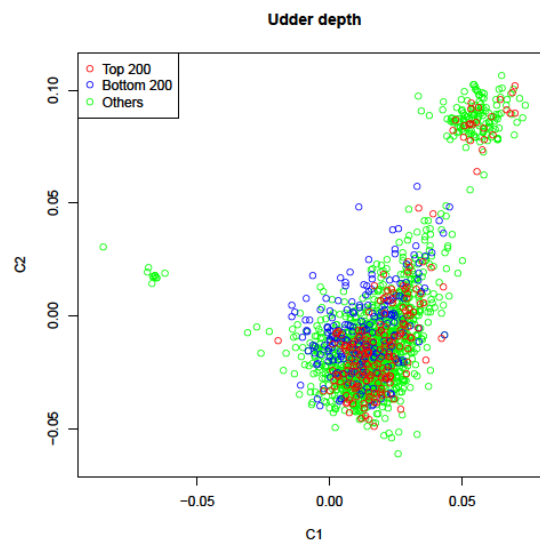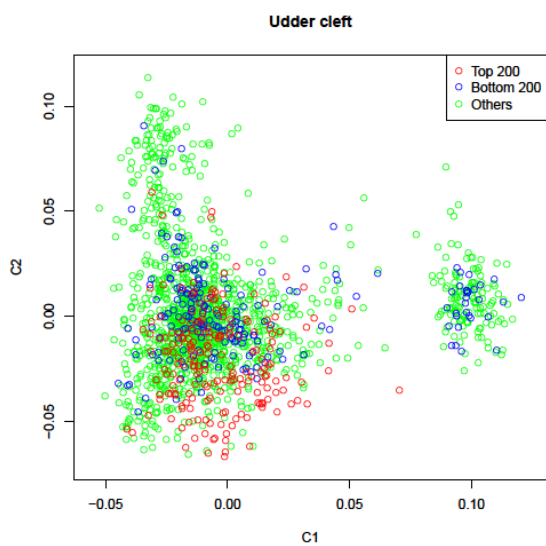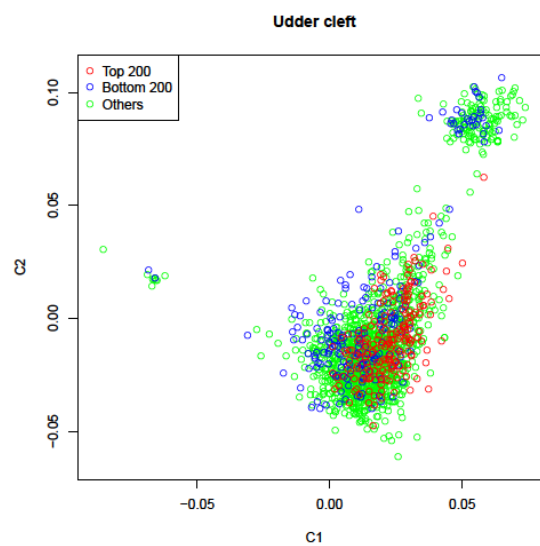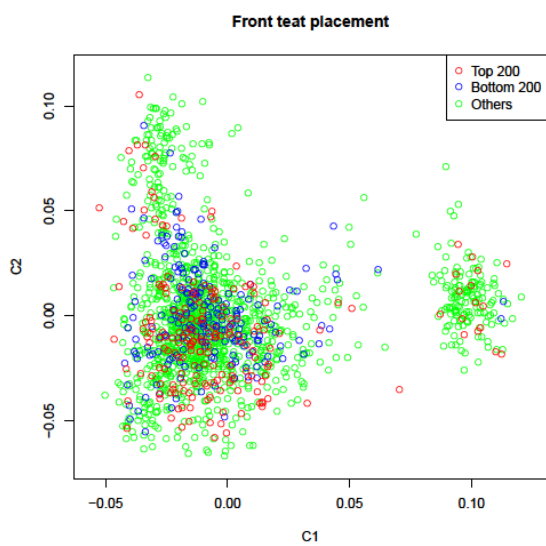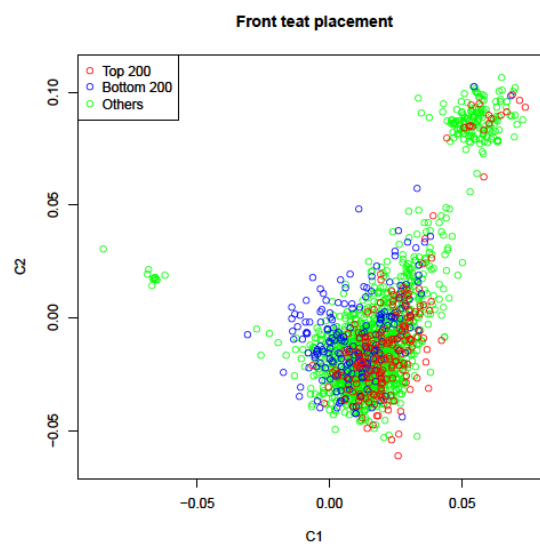

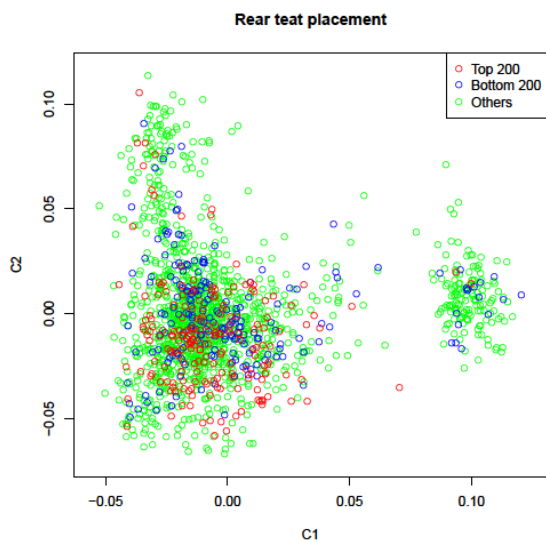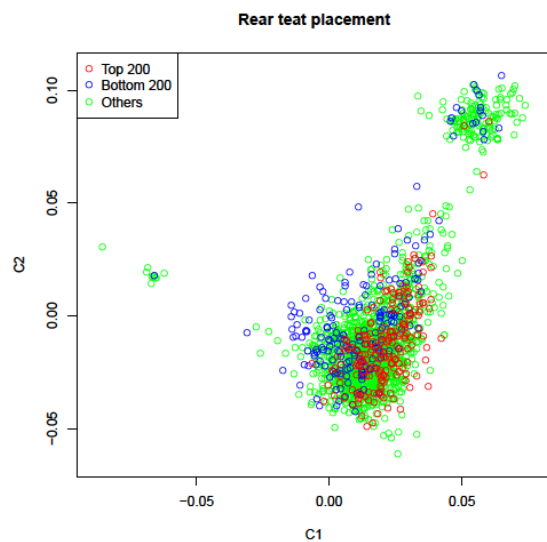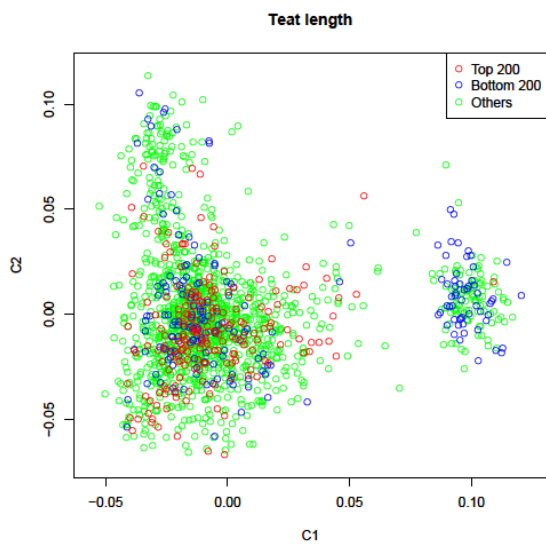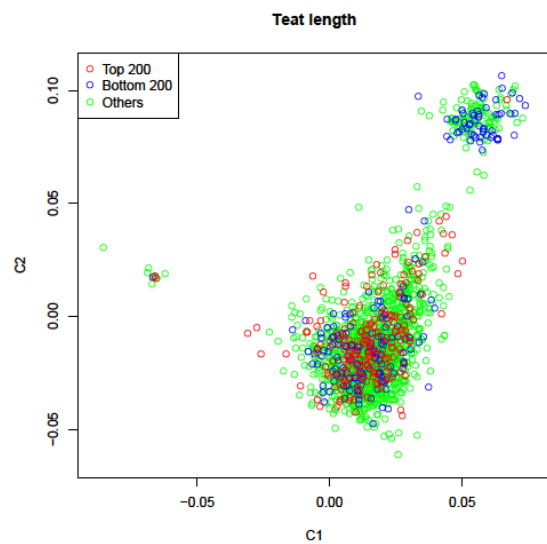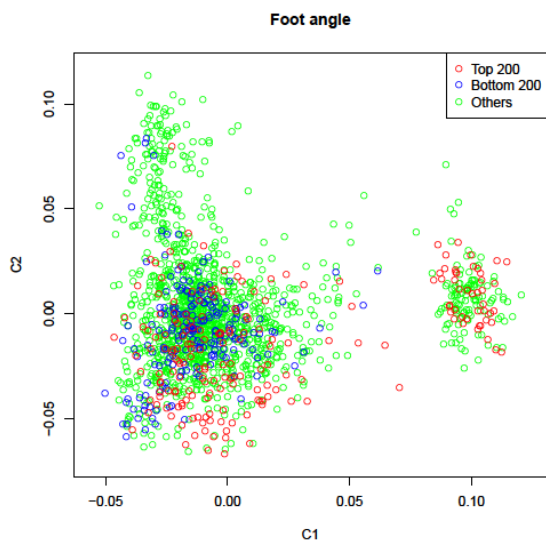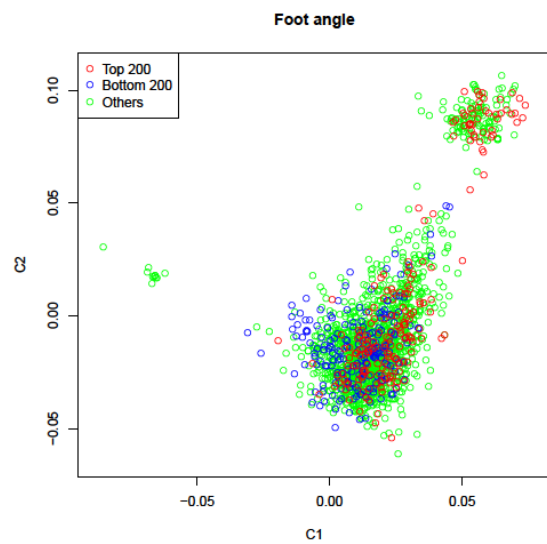

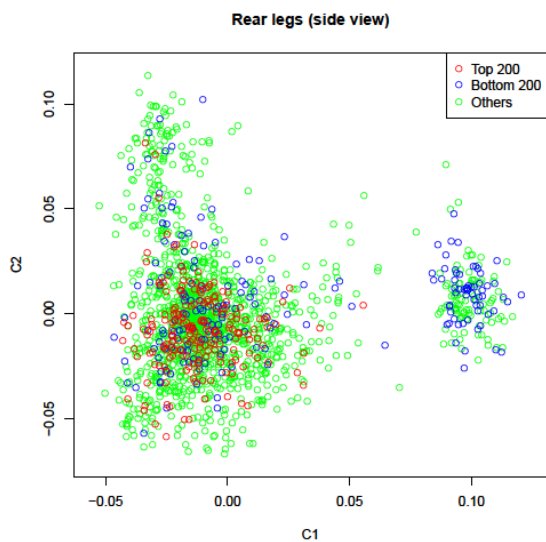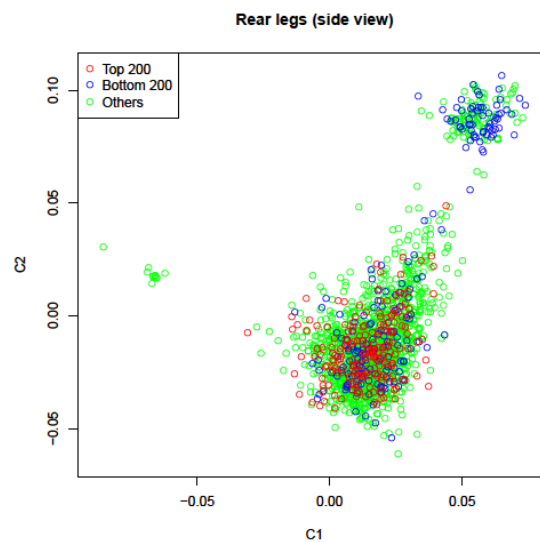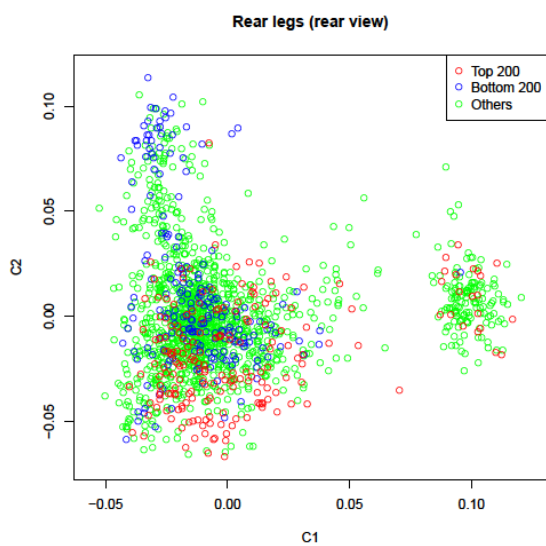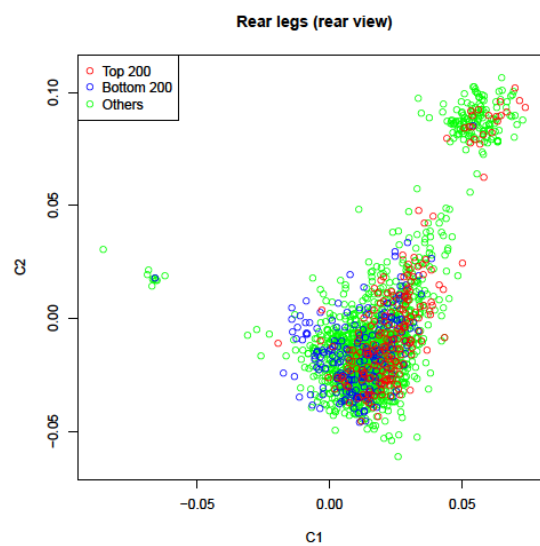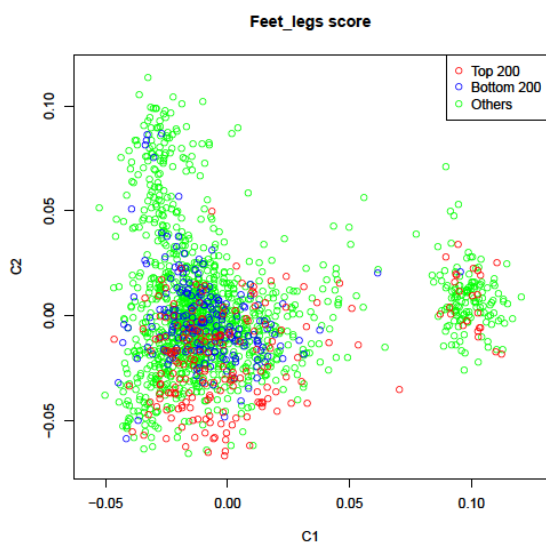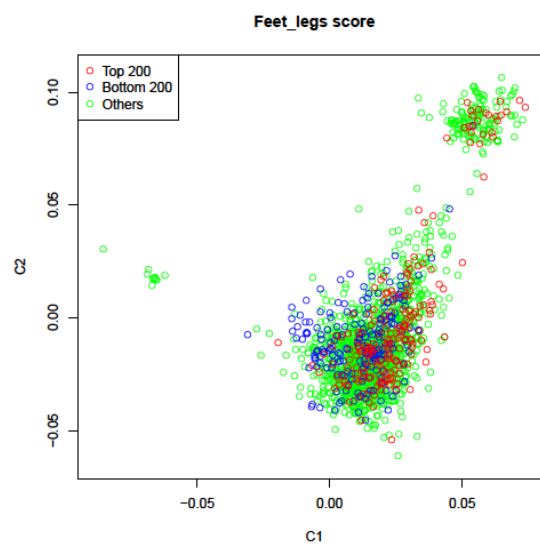

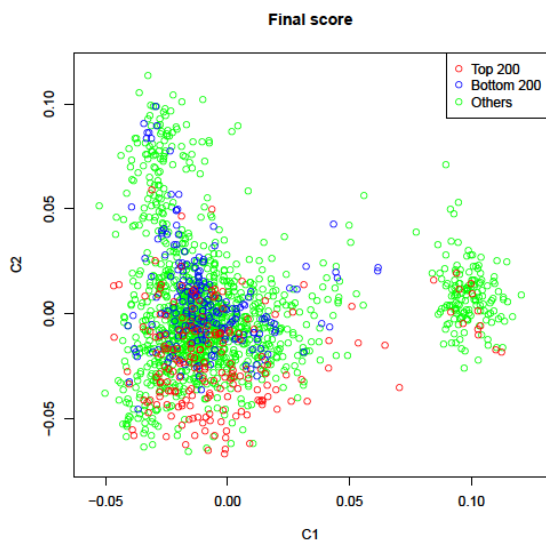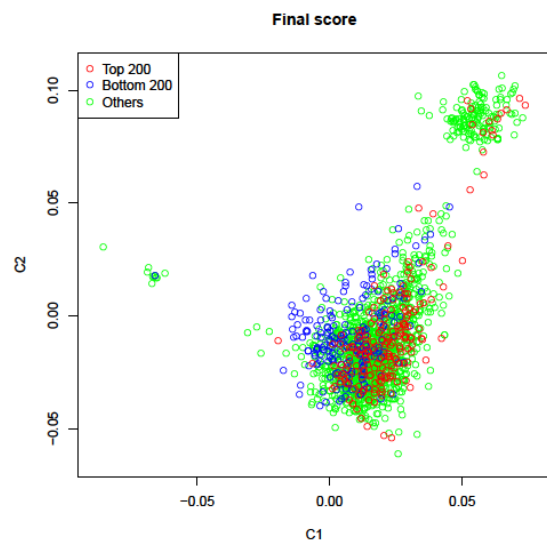

Supplement: Additional file 4 — Figure S4. Overlap between genome stratification and phenotypic stratification of 31 traits. C1 = dimension 1, C2 = dimension 2. Left column: C1 and C2 values were calculated using 1,654 contemporary Holstein cows. Right column: C1 and C2 values were calculated using 2,366 contemporary and historical Holstein cattle, including the University of Minnesota Holstein control line that remained unselected since 1964. ‘Top 200’ are the 200 cows with the highest PTA values for the trait, ‘Bottom 200’ are the 200 cows with the lowest PTA values for the trait, and ‘Other’ are cows with PTA values between top 200 and bottom 200. [file 1471-2164-13-536-S4.pdf]

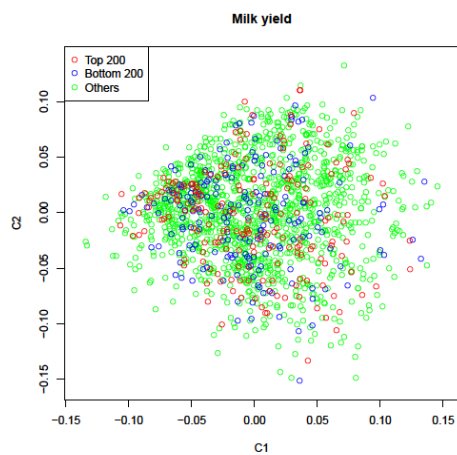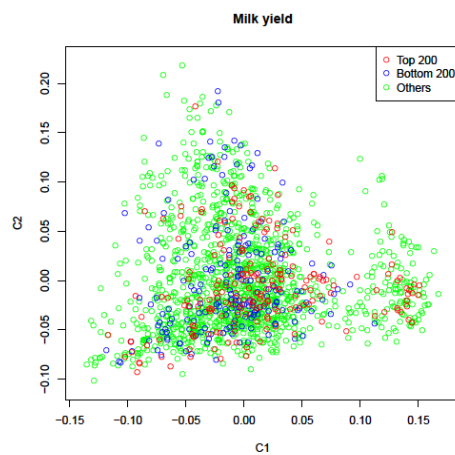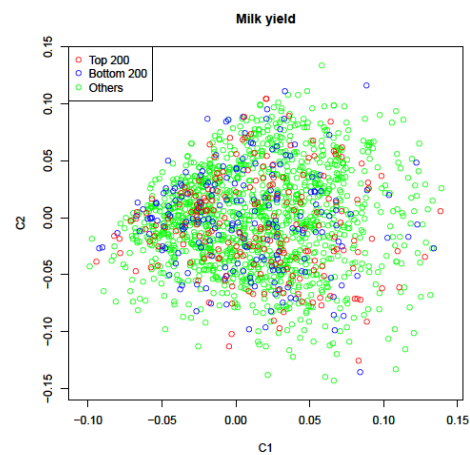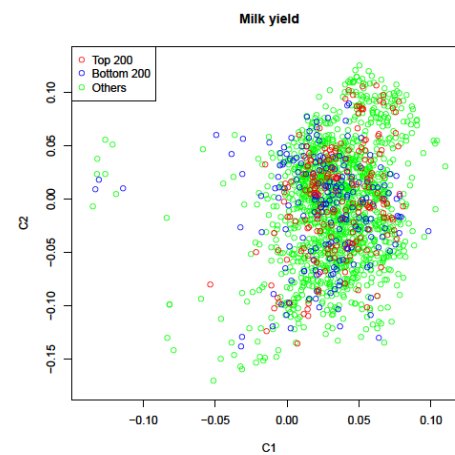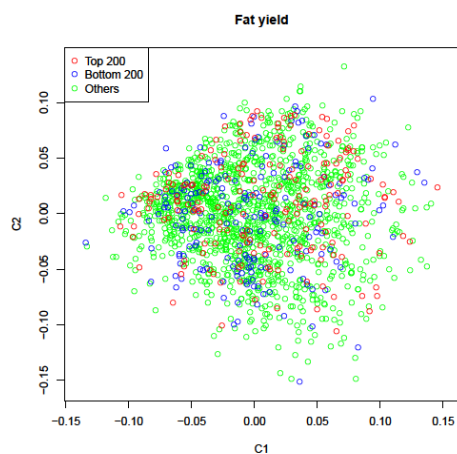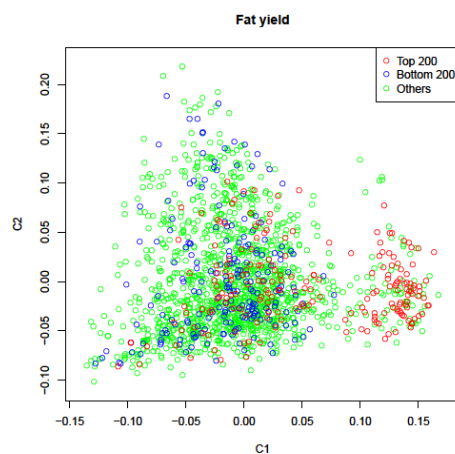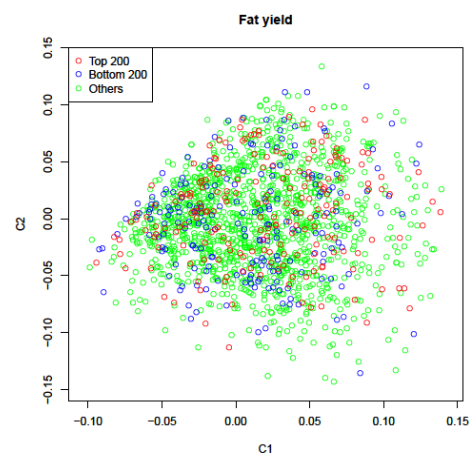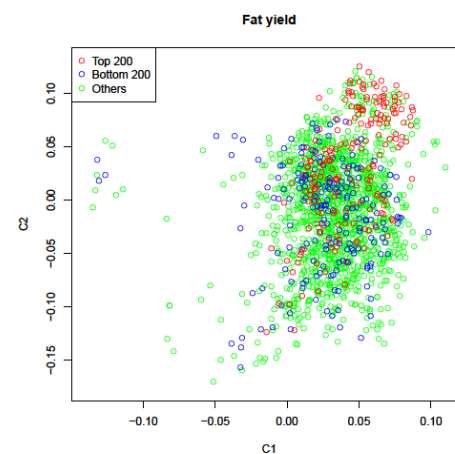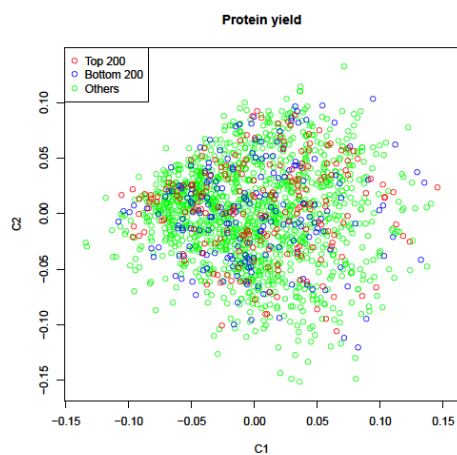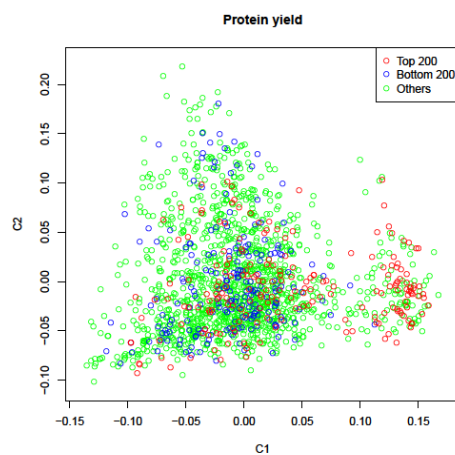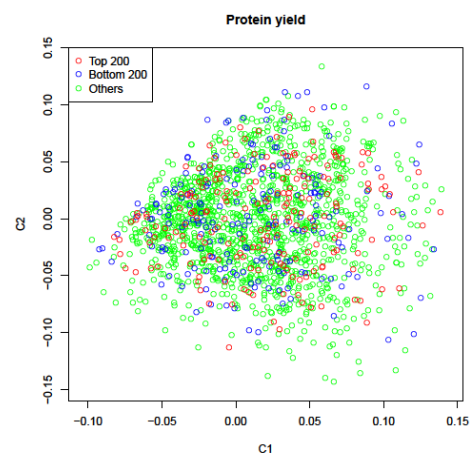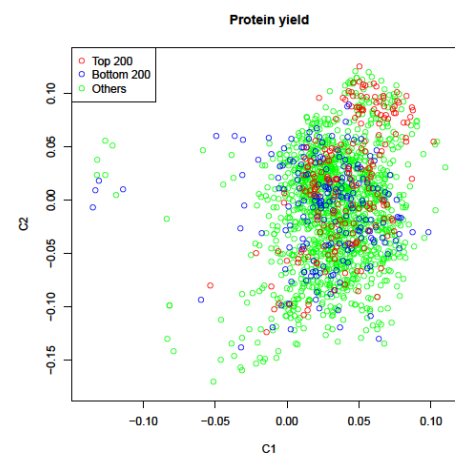

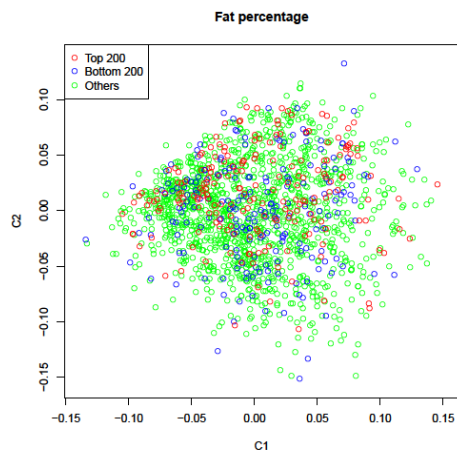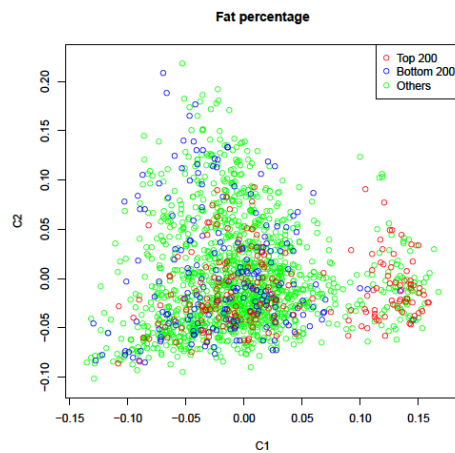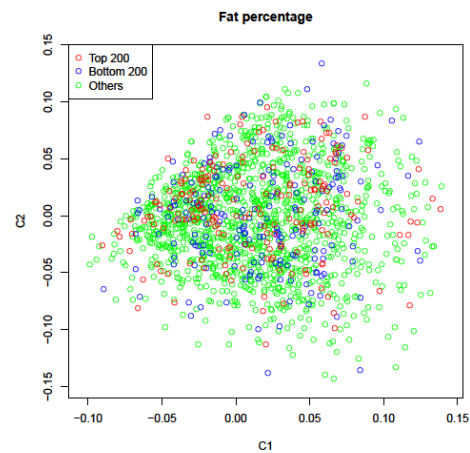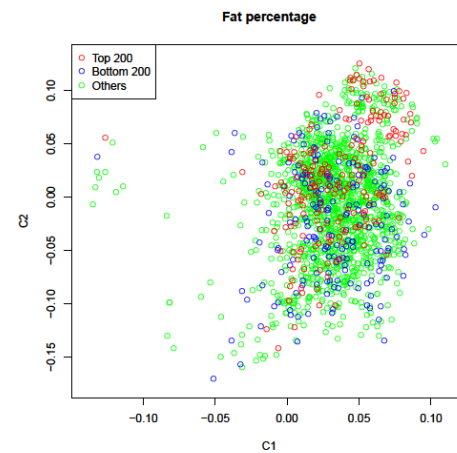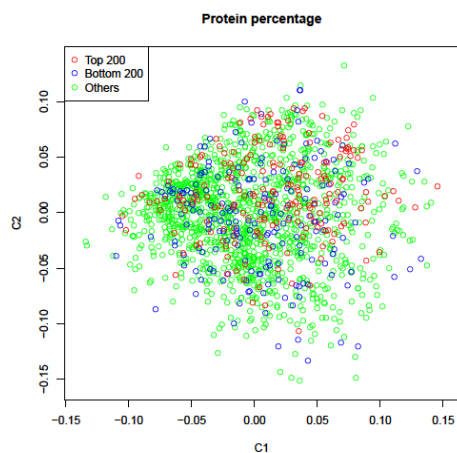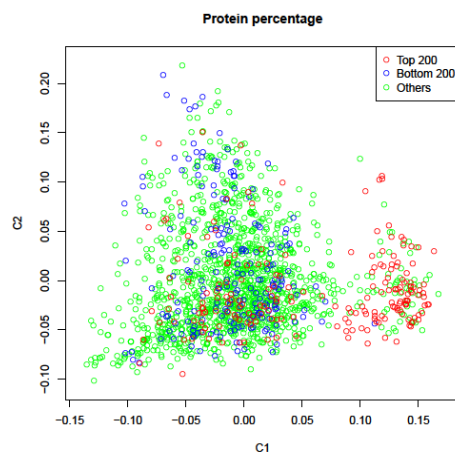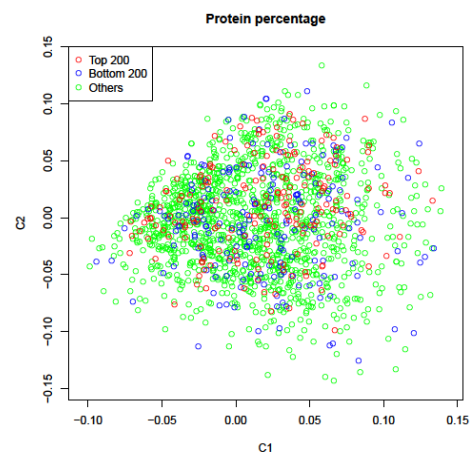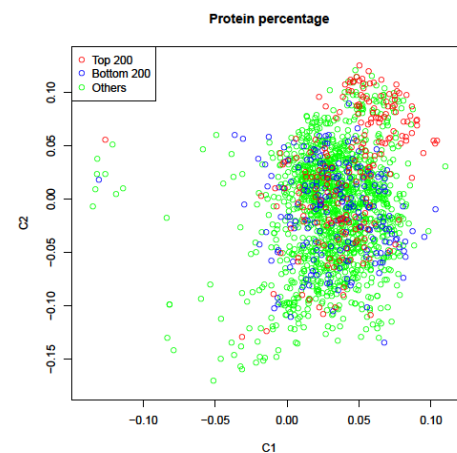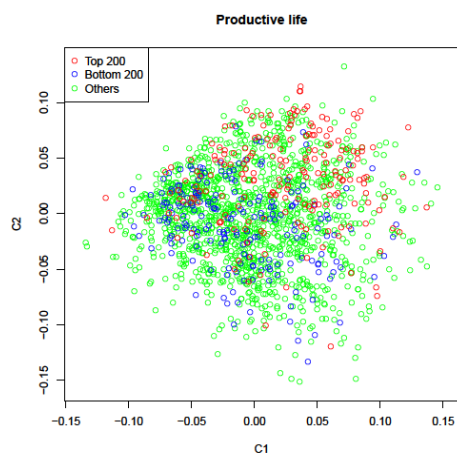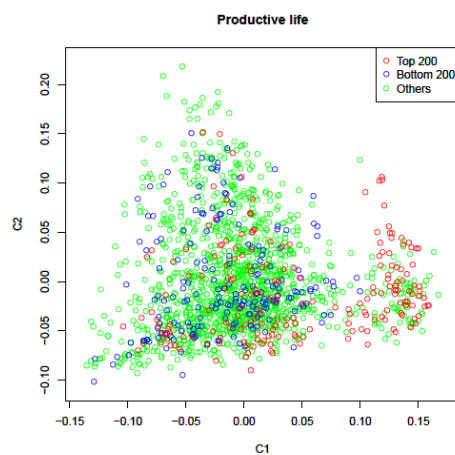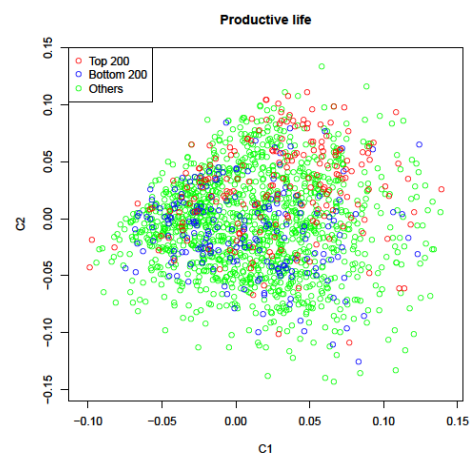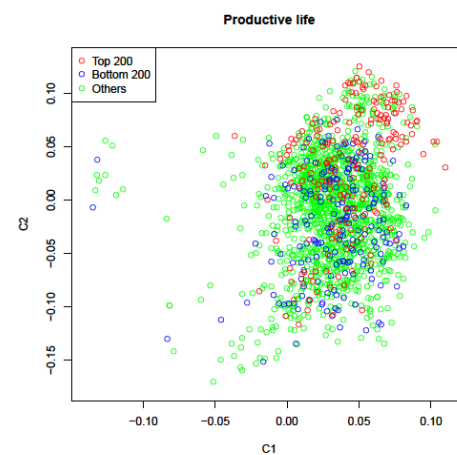

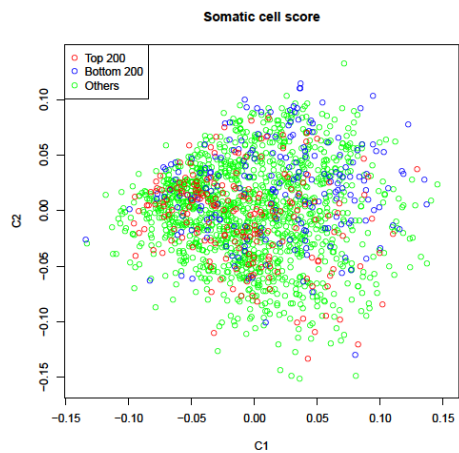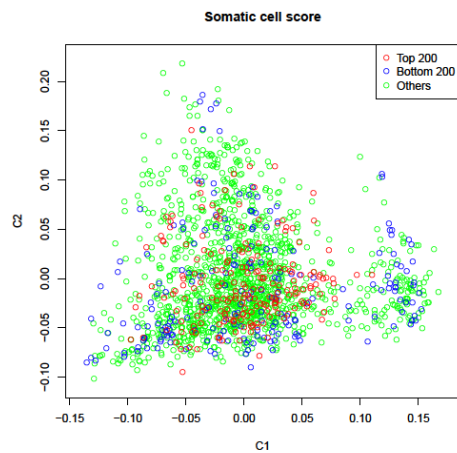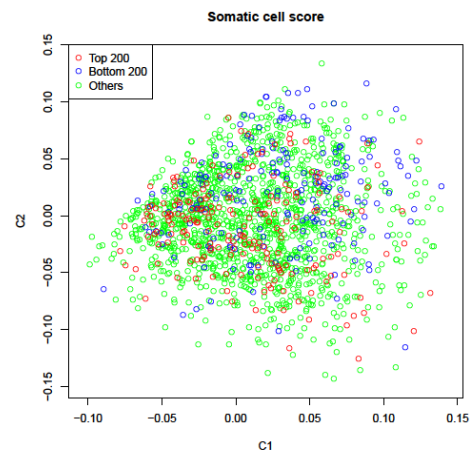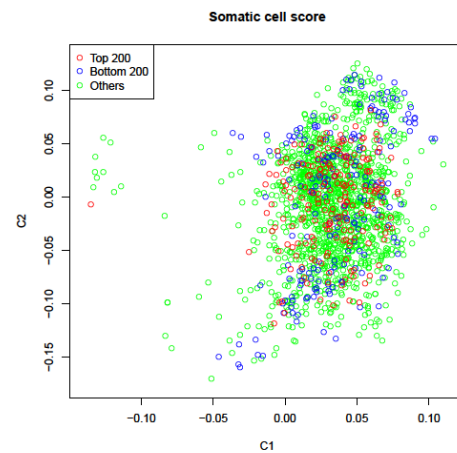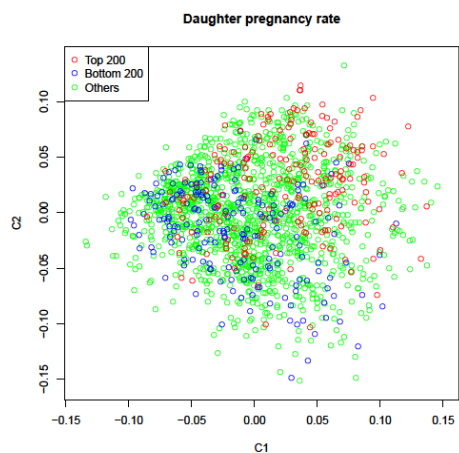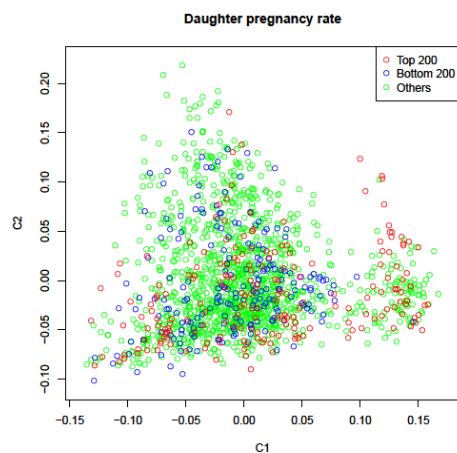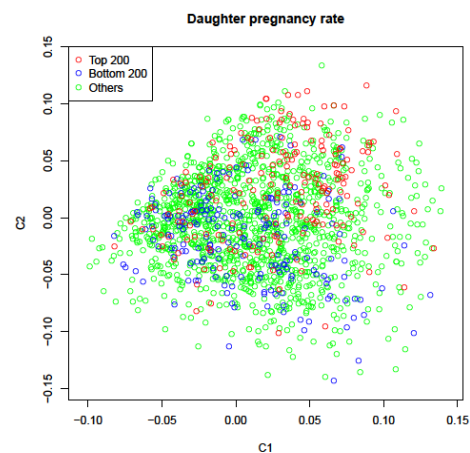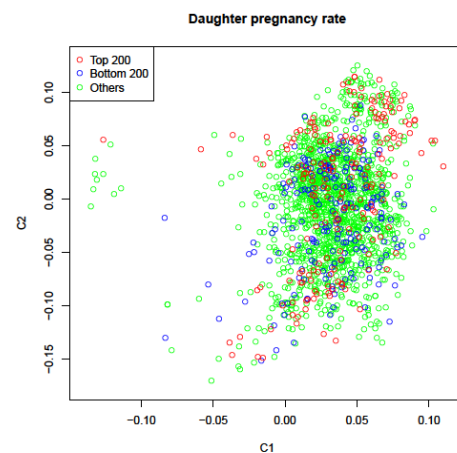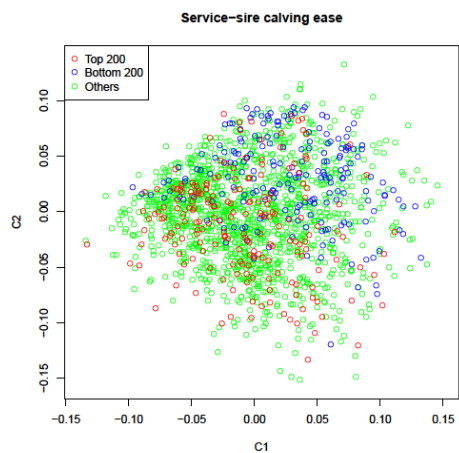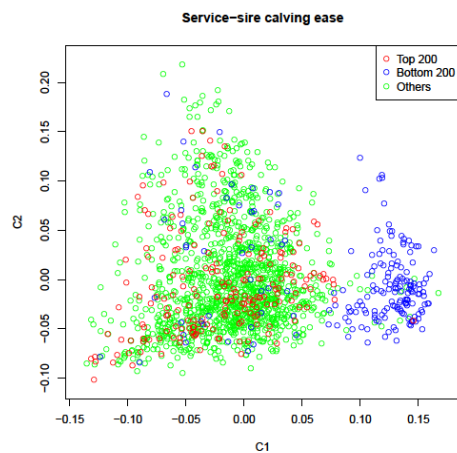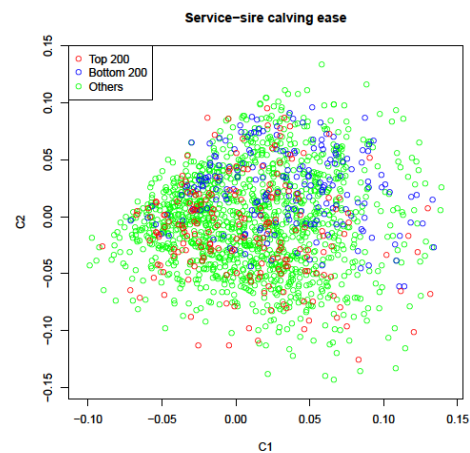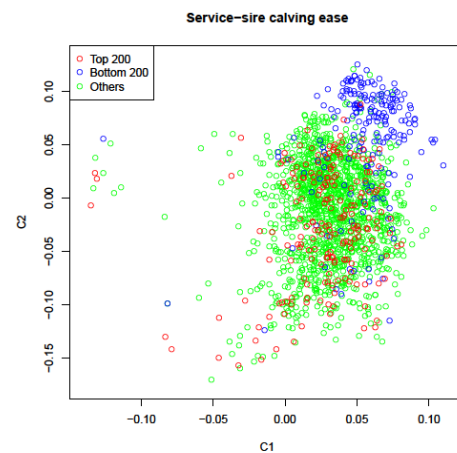

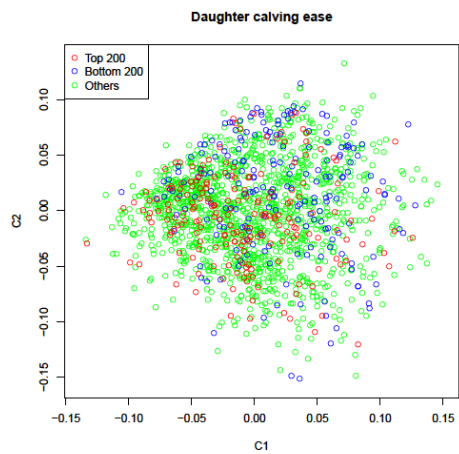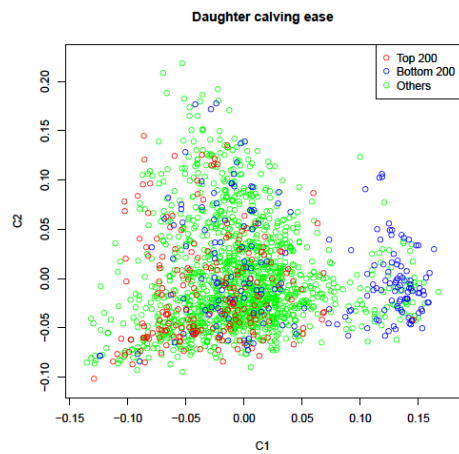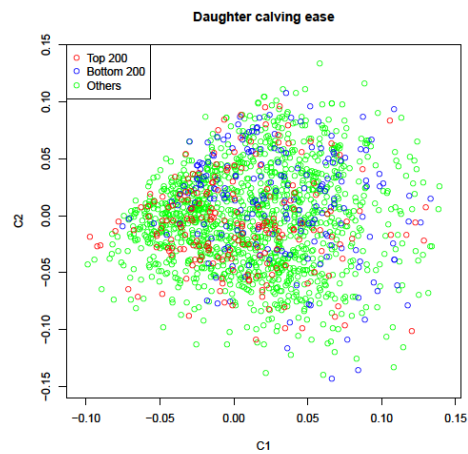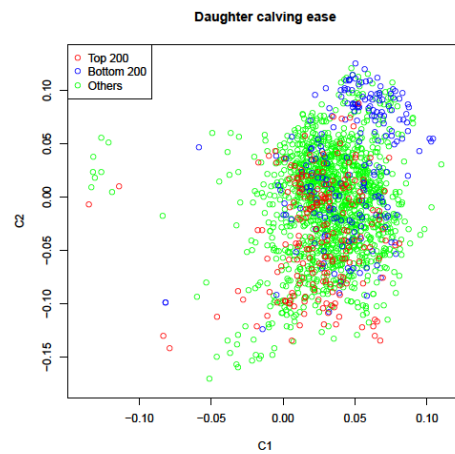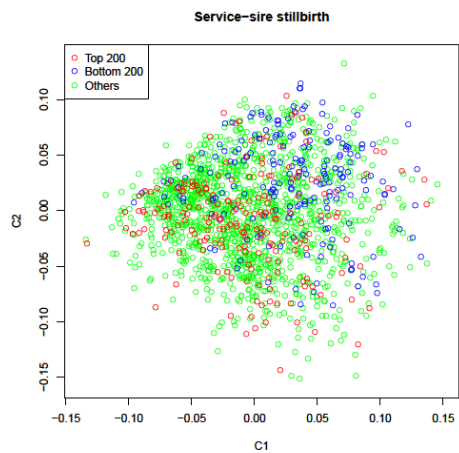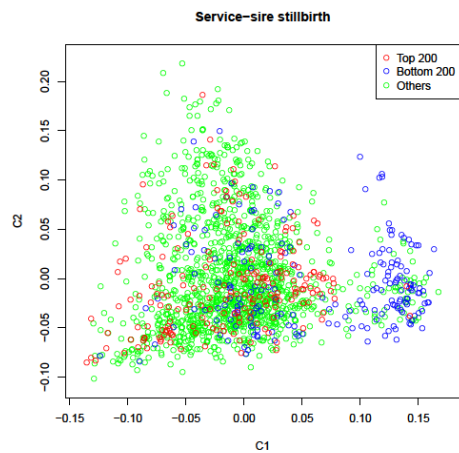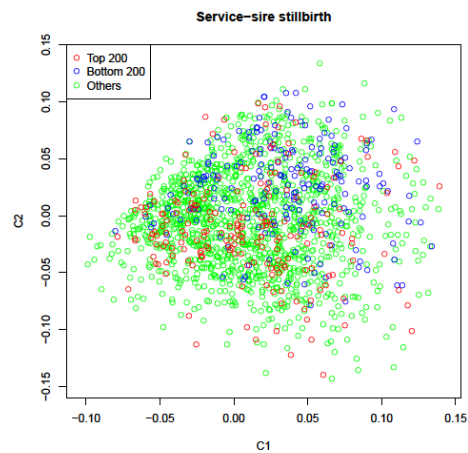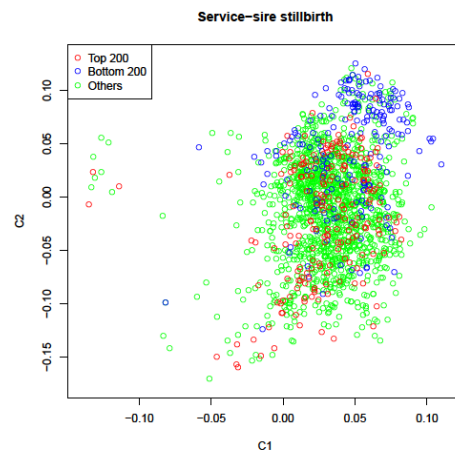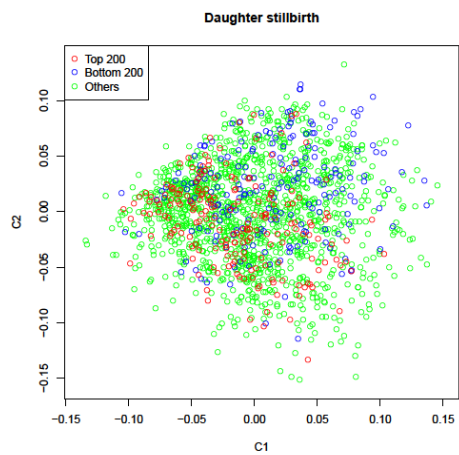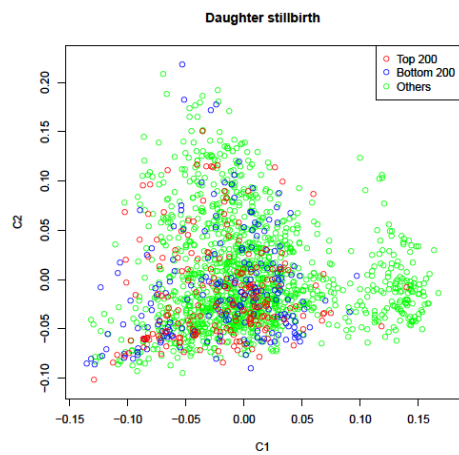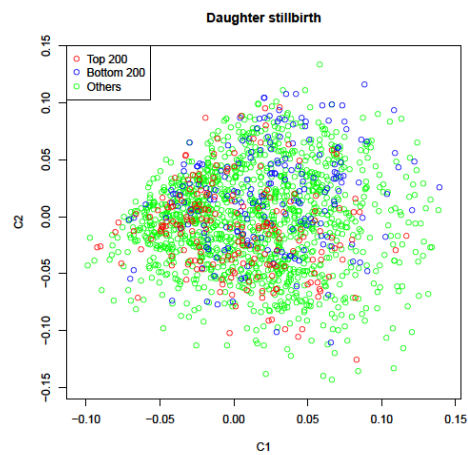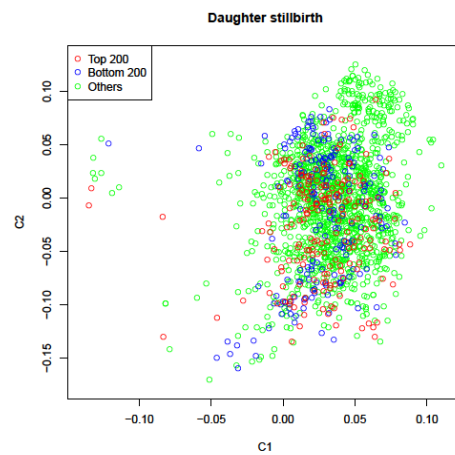

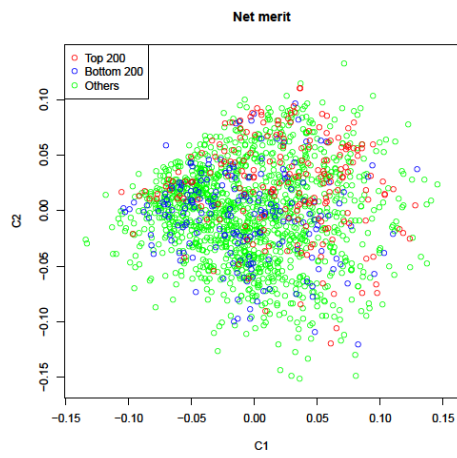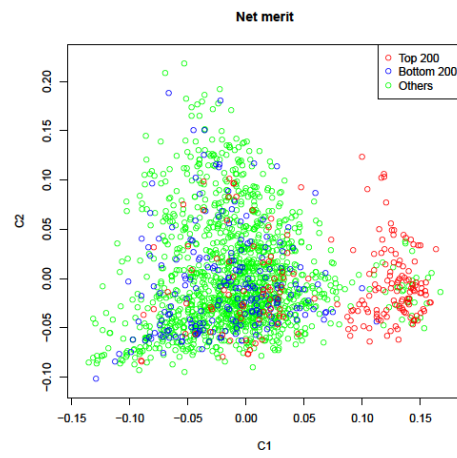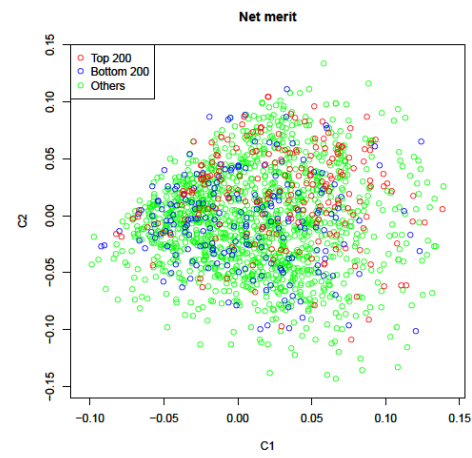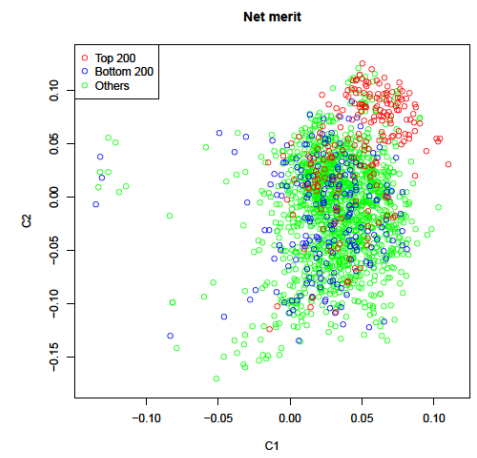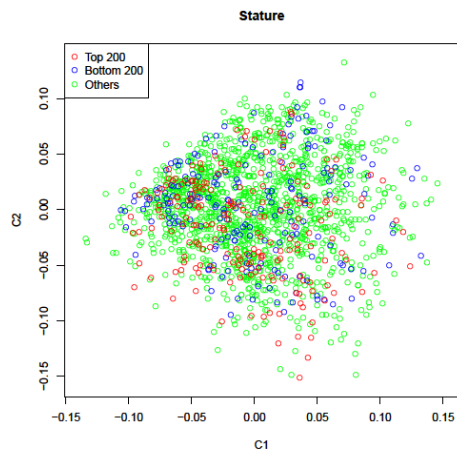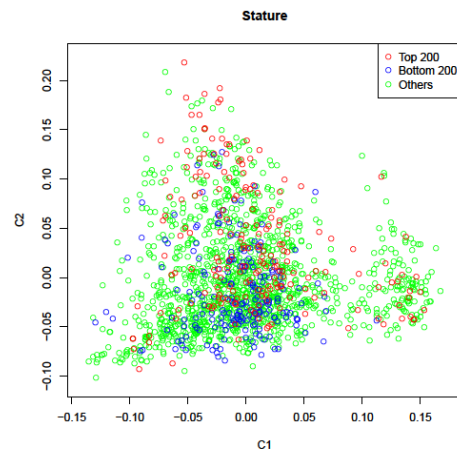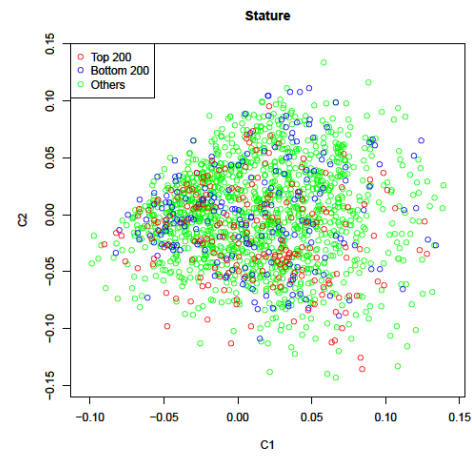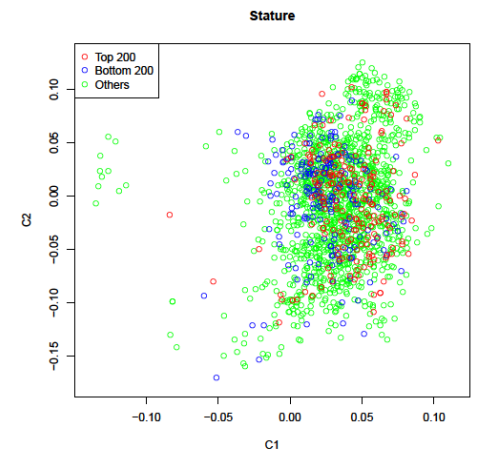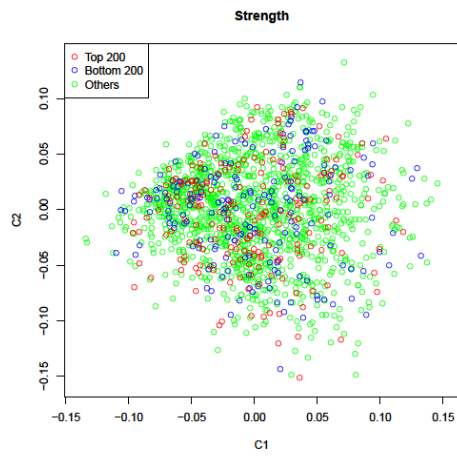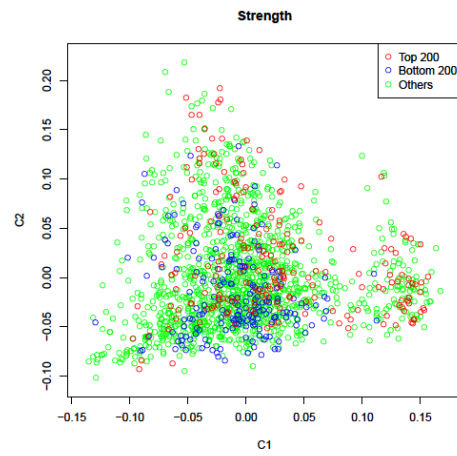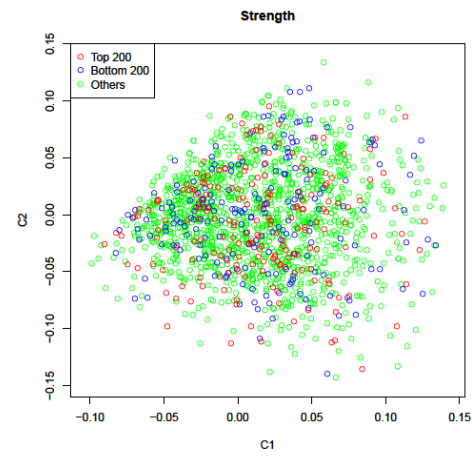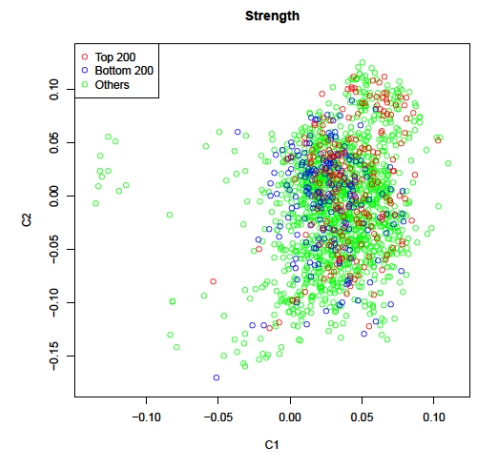

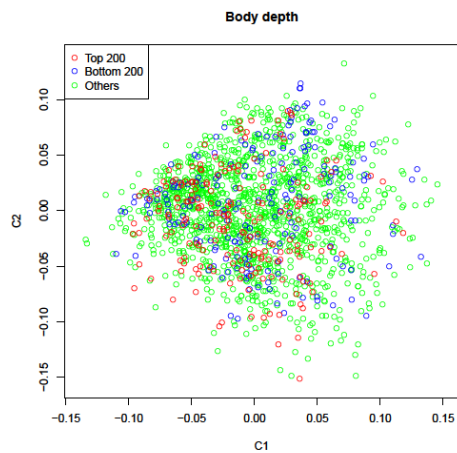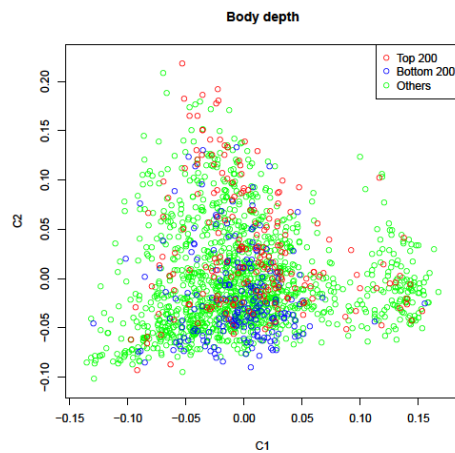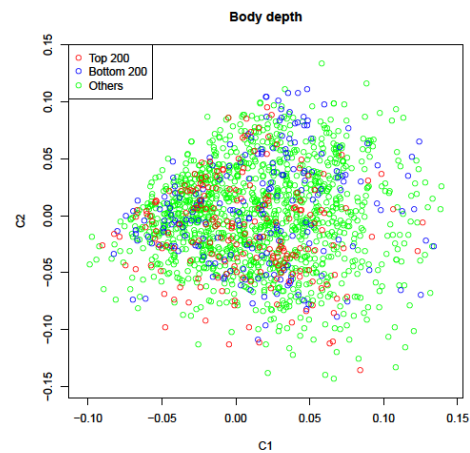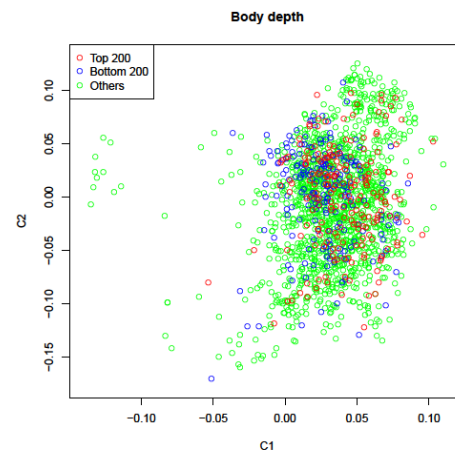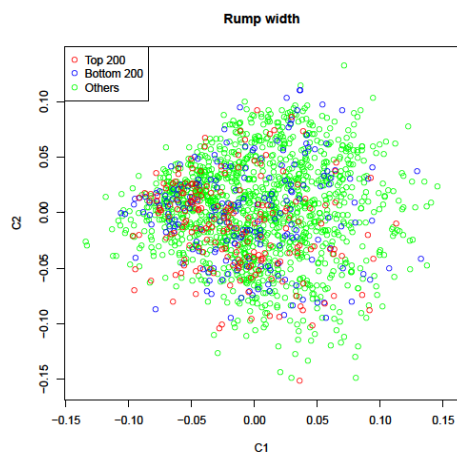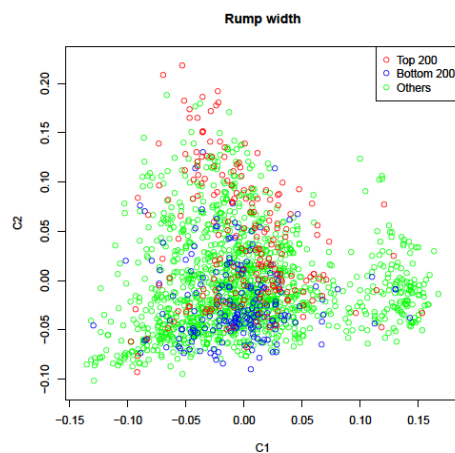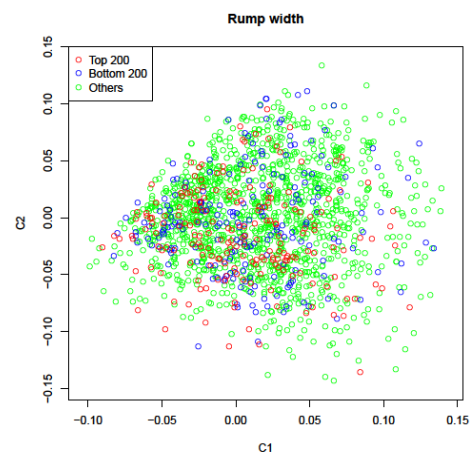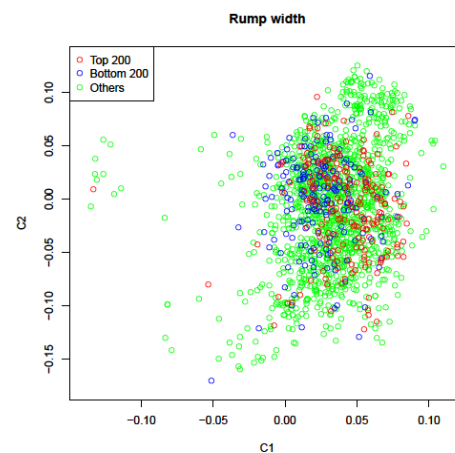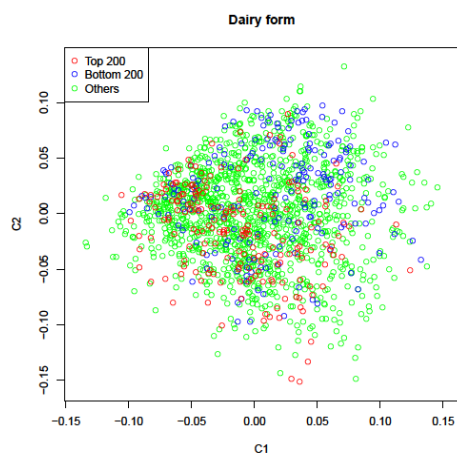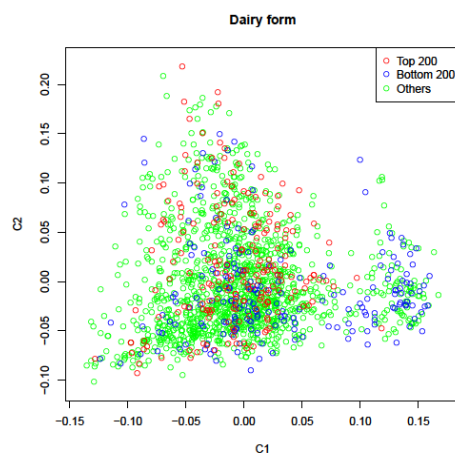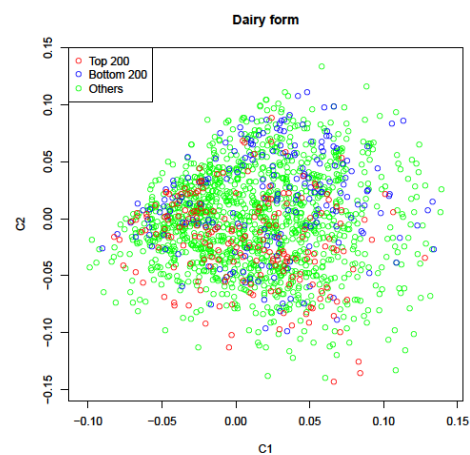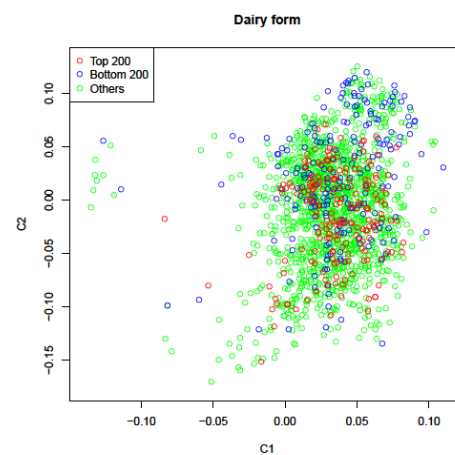

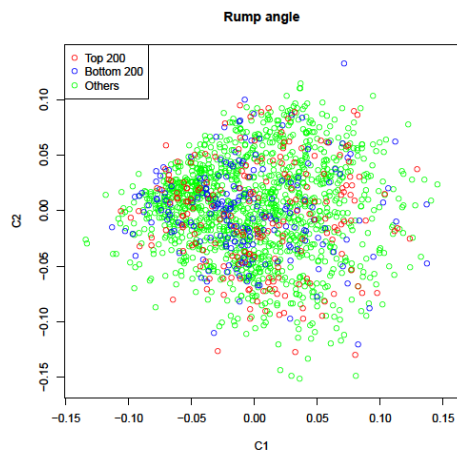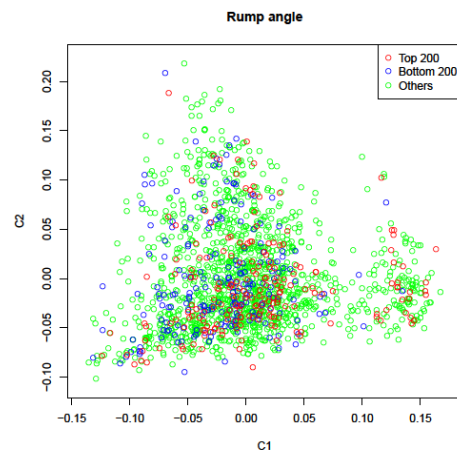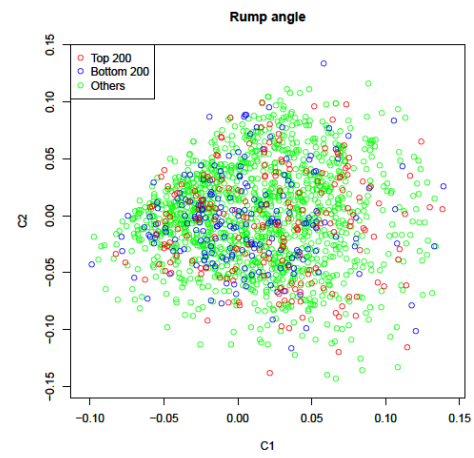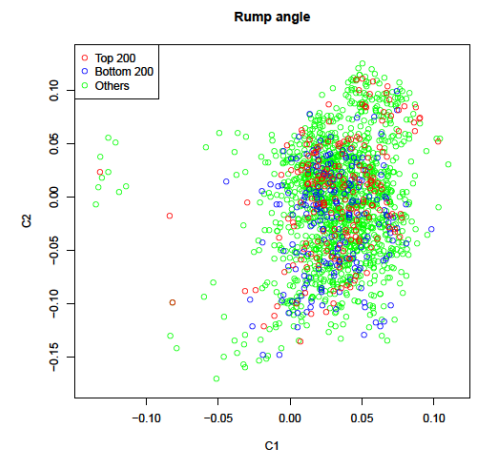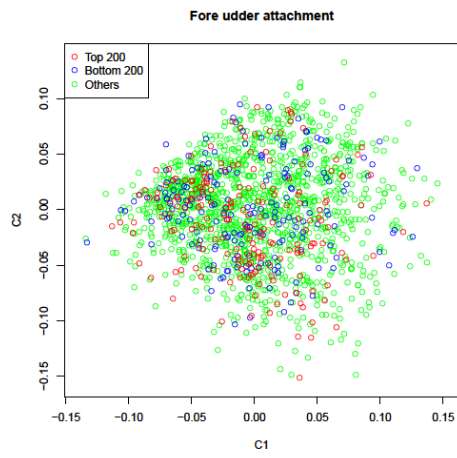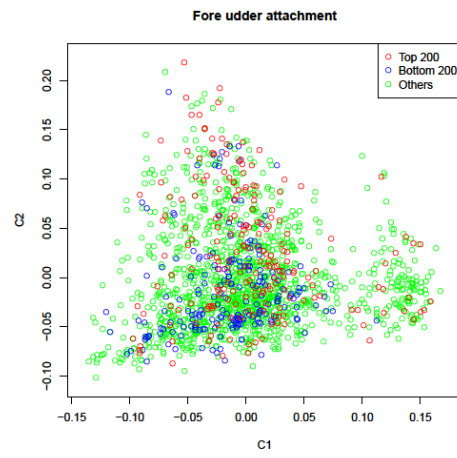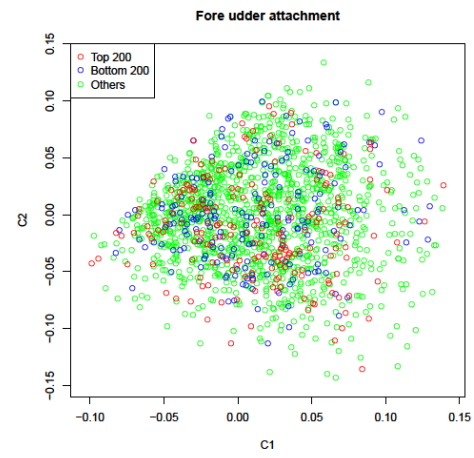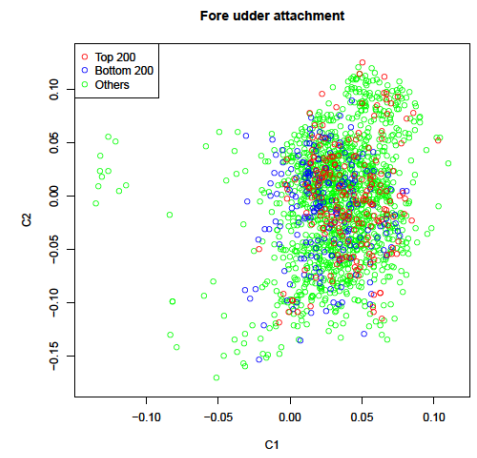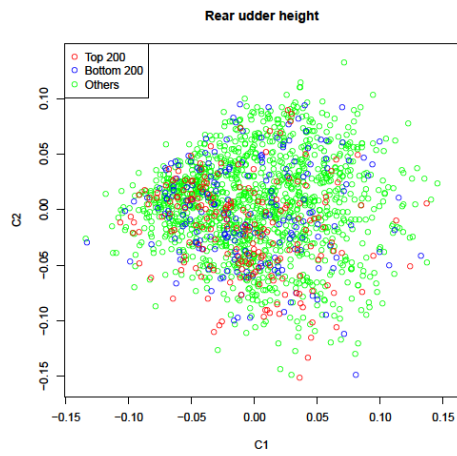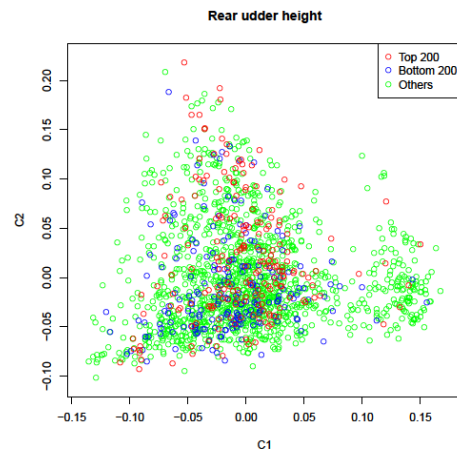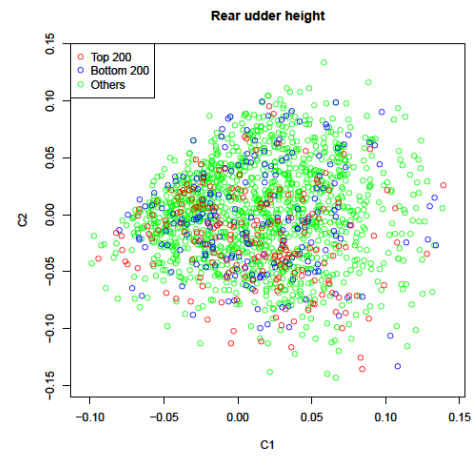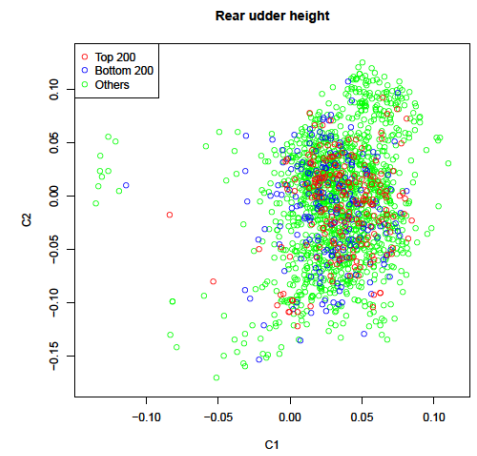

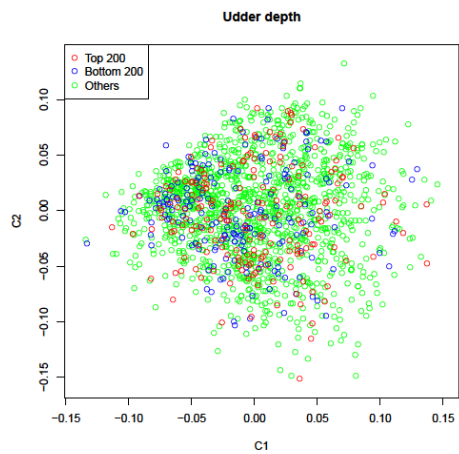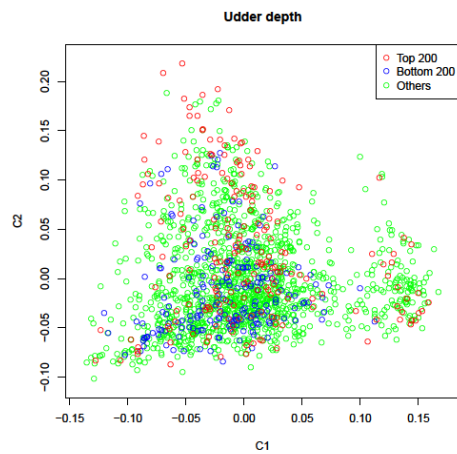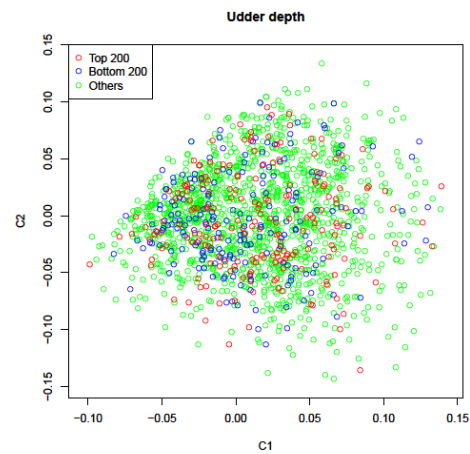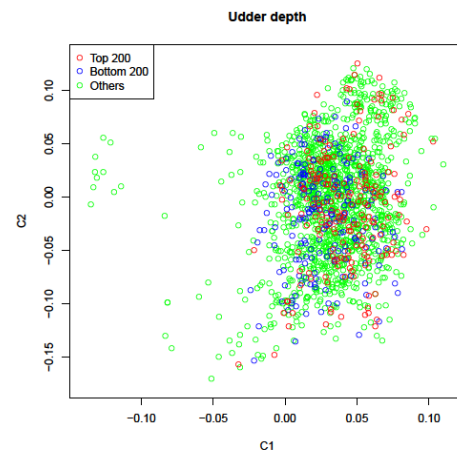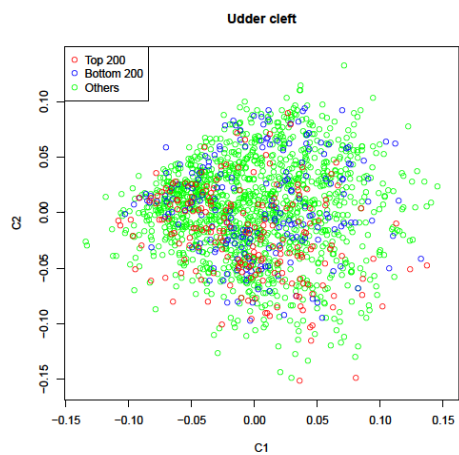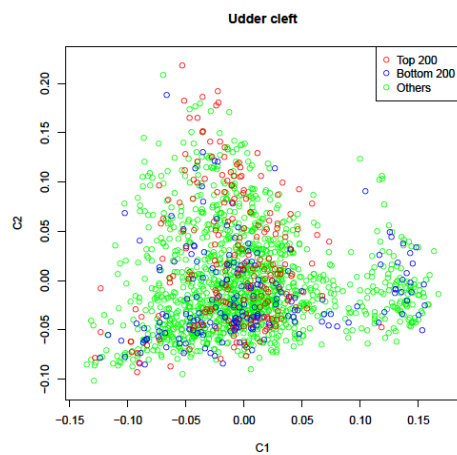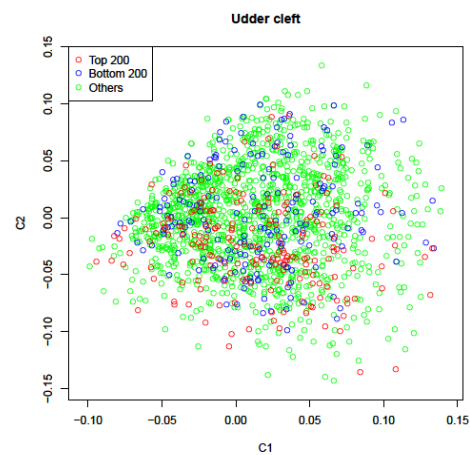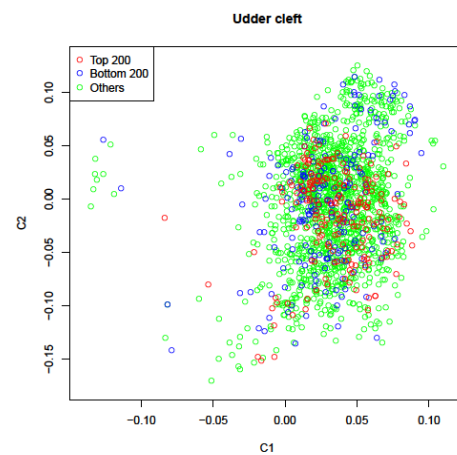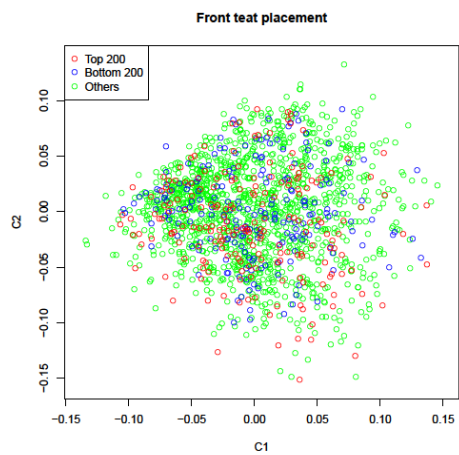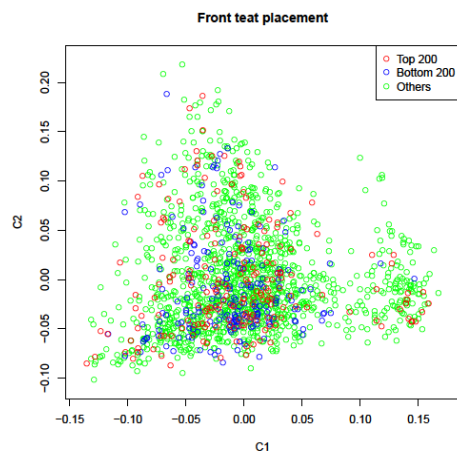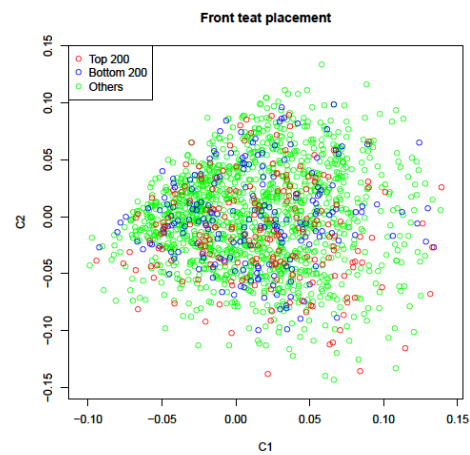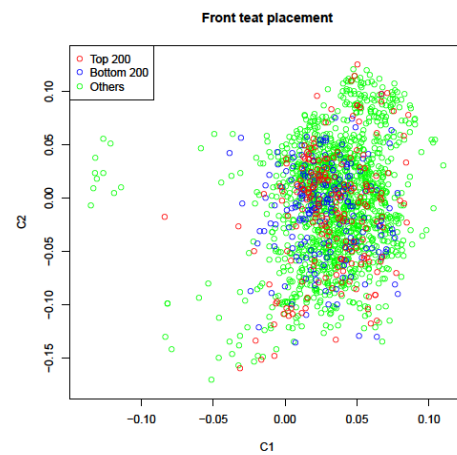

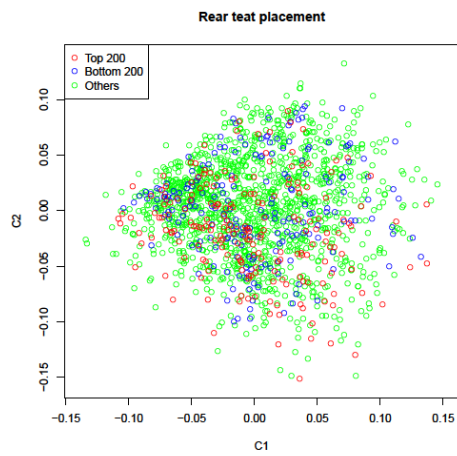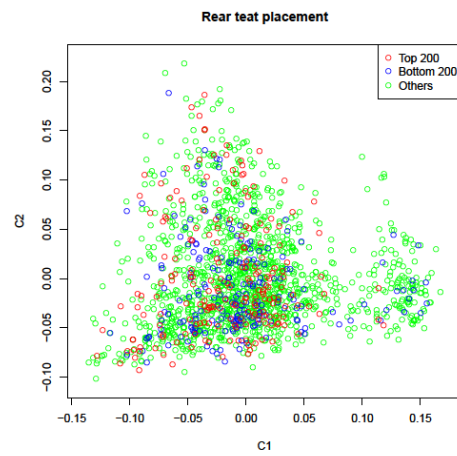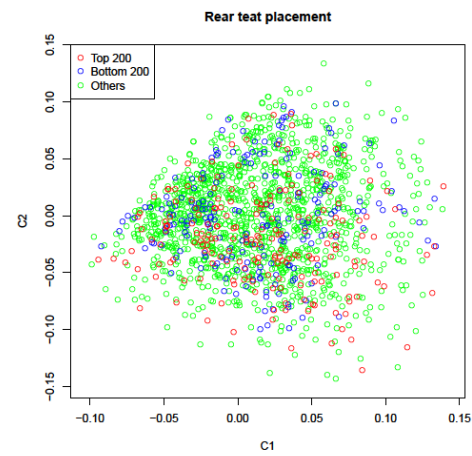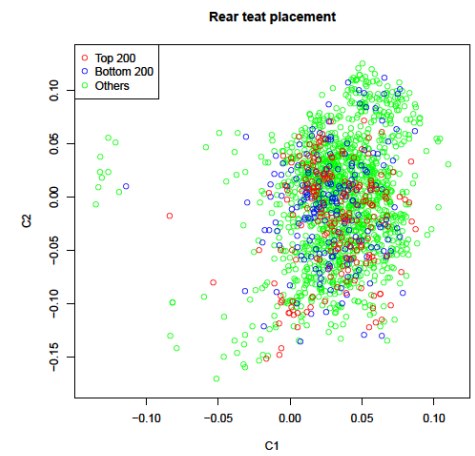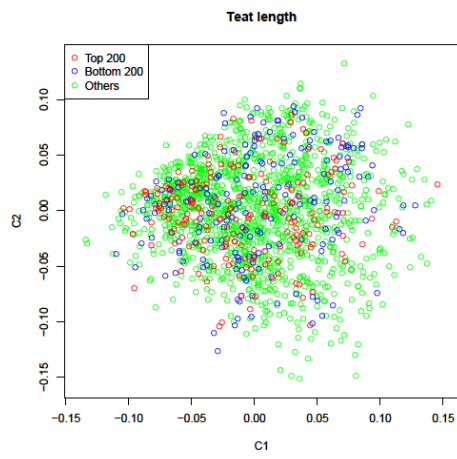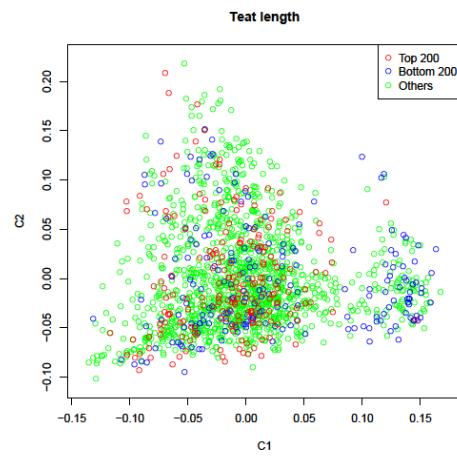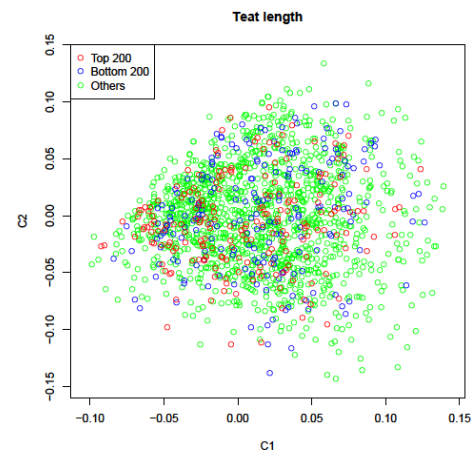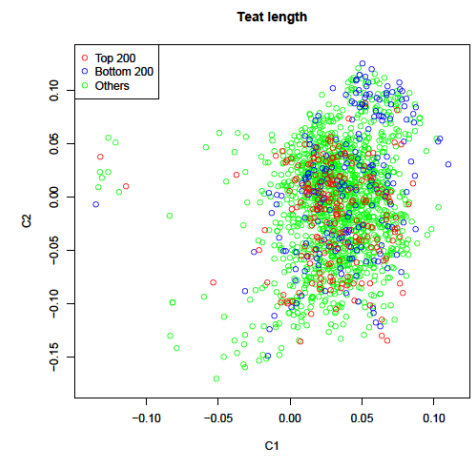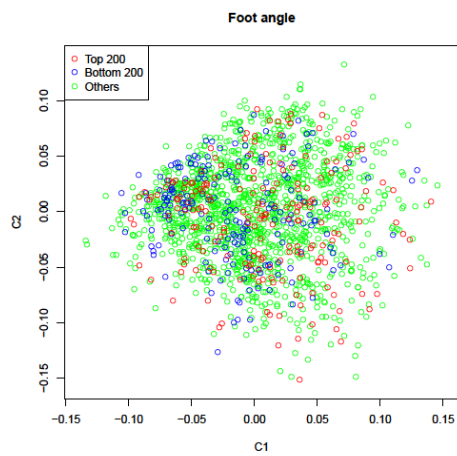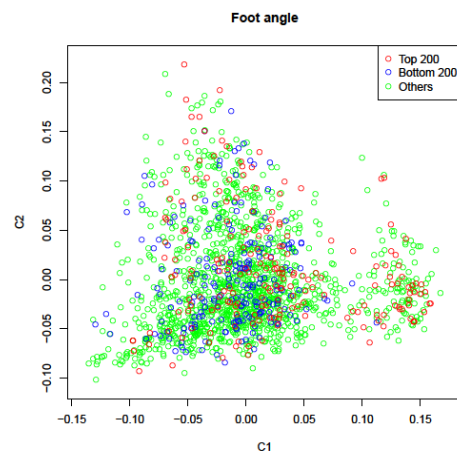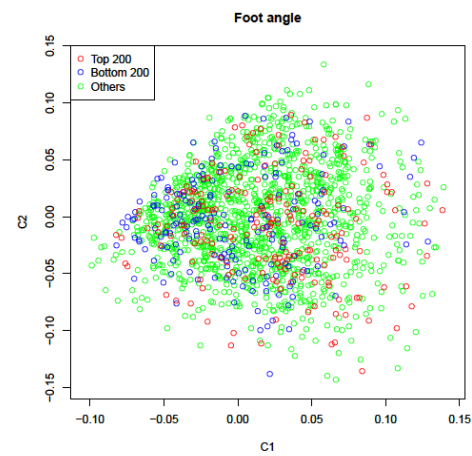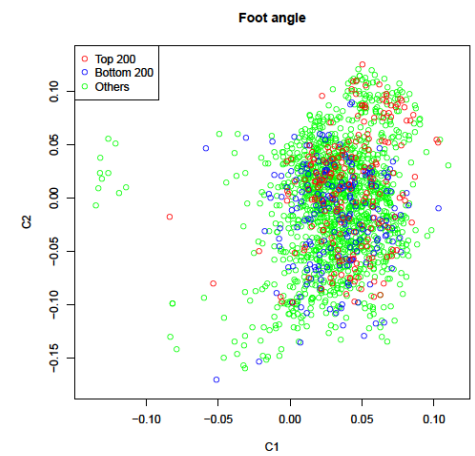

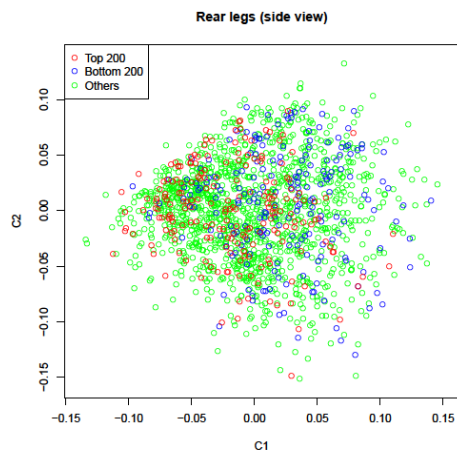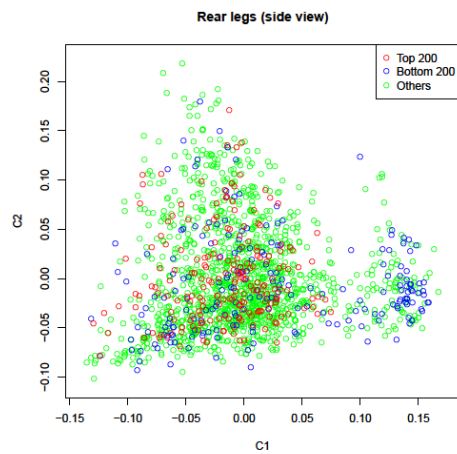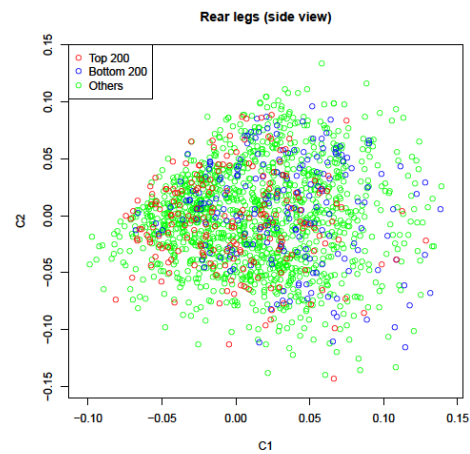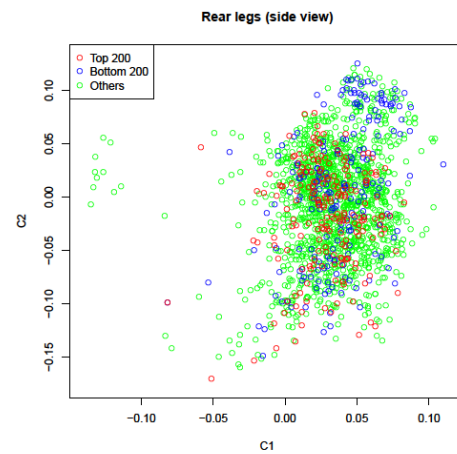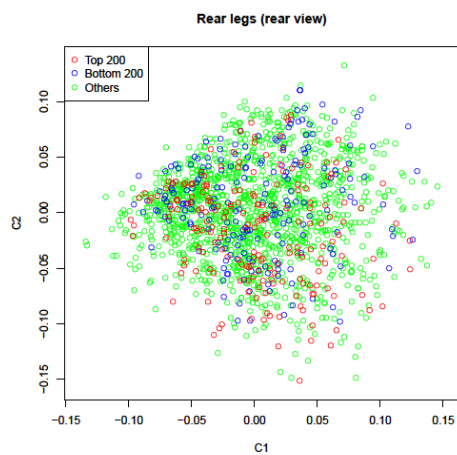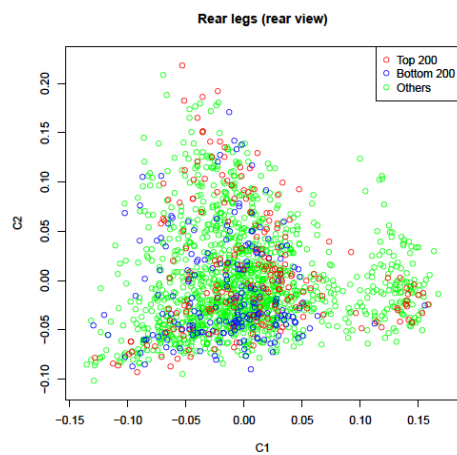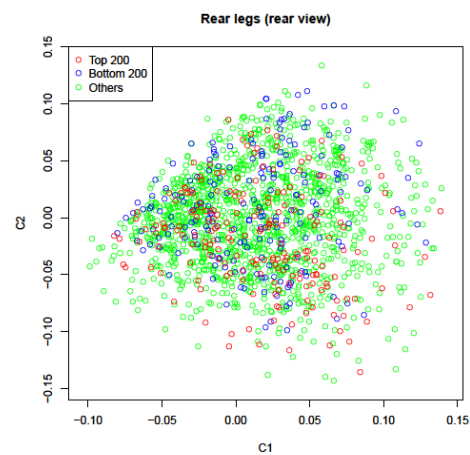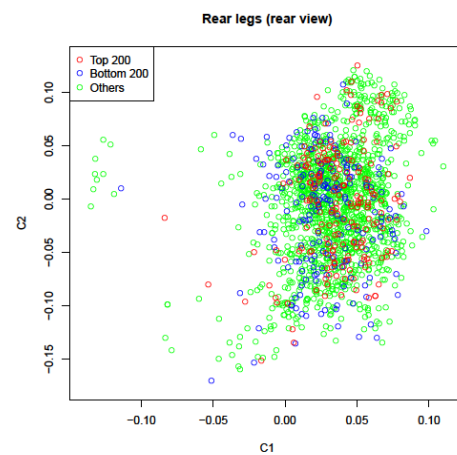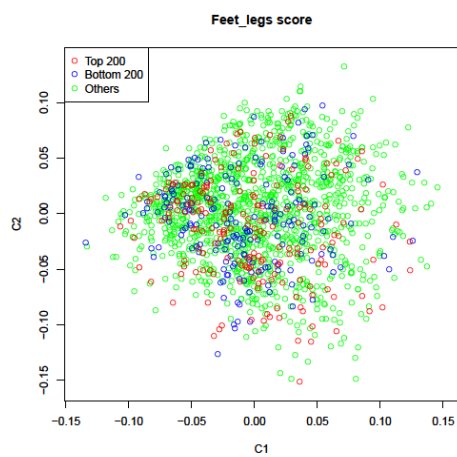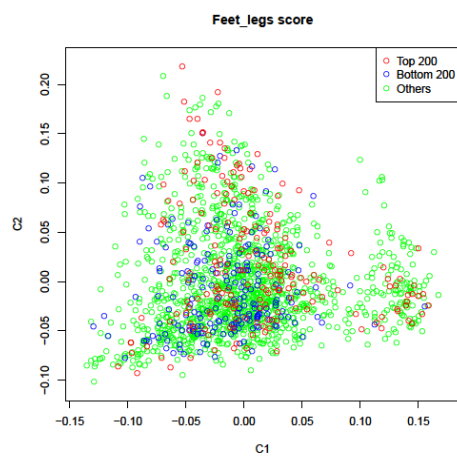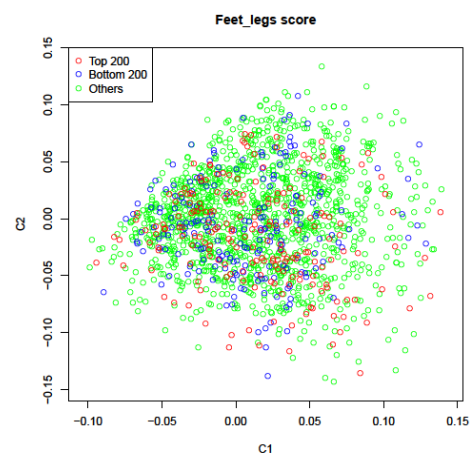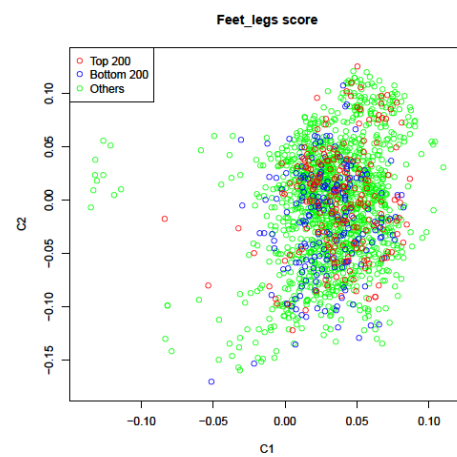

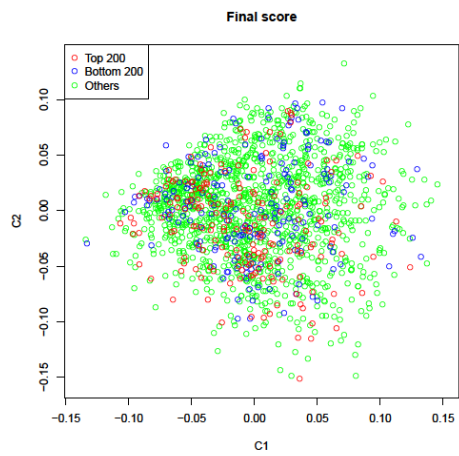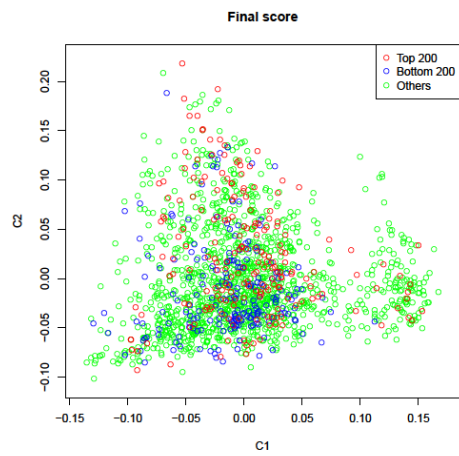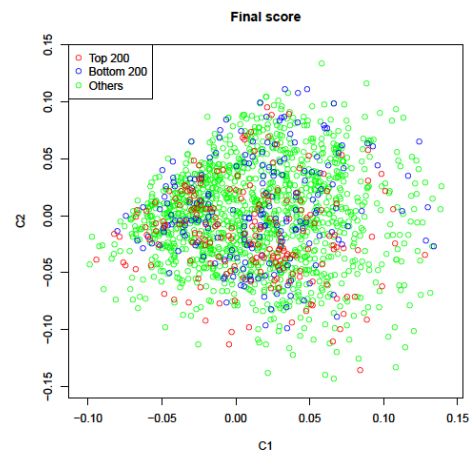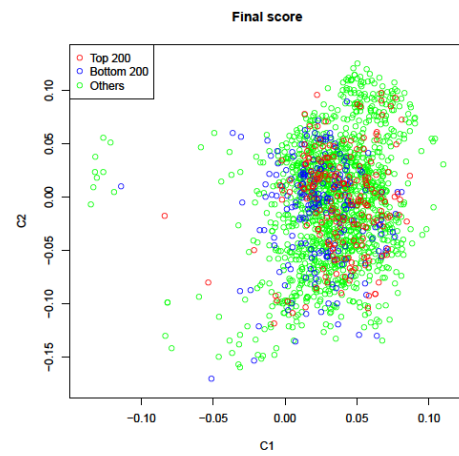

Supplement: Additional file 5 — Figure S5. Overlap between genome stratification and phenotypic stratification for chromosome 1 and the X chromosome. Column 1: chromosome 1; Column 2: X chromosome; C1 and C2 values were calculated using 1,654 contemporary Holstein cows. Column 3: chromosome 1; Column 4: X chromosome; C1 and C2 values were calculated using 2,366 Holstein cattle, including the University of Minnesota Holstein control line that remained unselected since 1964. C1 = dimension 1, C2 = dimension 2; ‘Top 200’ are the 200 cows with the highest PTA values for the trait, ‘Bottom 200’ the 200 cows with the lowest PTA values for the trait, and ‘Other’ are cows with PTA values between top 200 and bottom 200. [file 1471-2164-13-536-S5.pdf]

## EMMAX-IBS

## EMMAX-BN

## GLS

## PCA

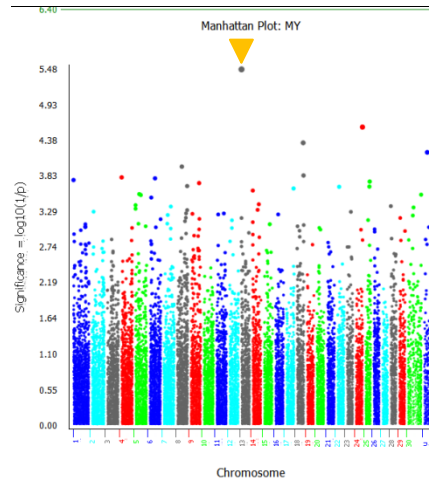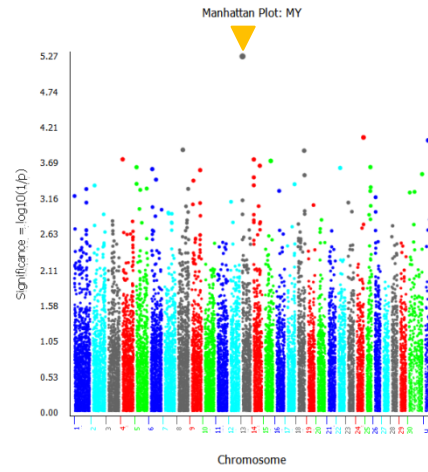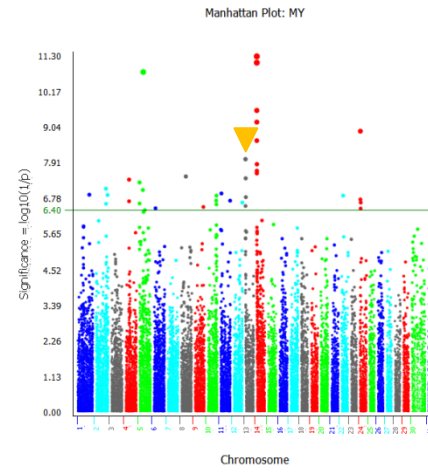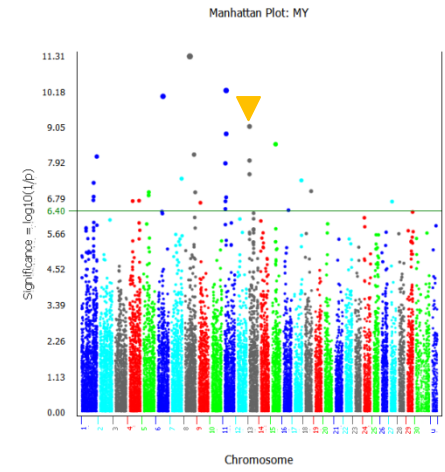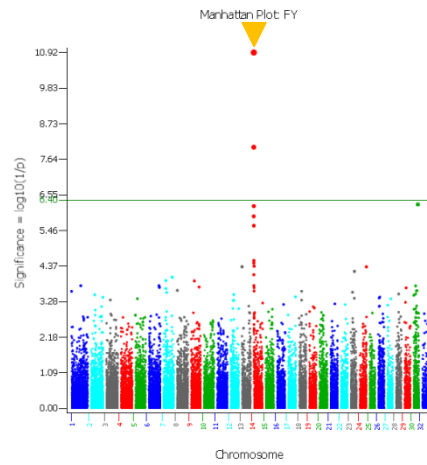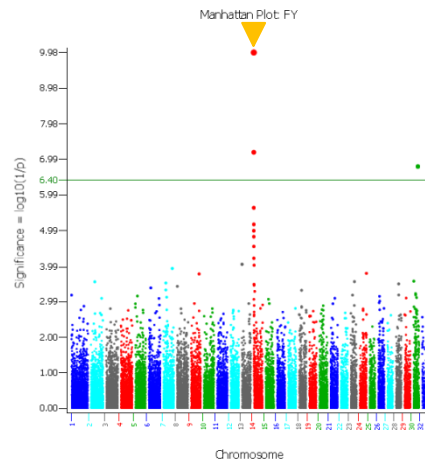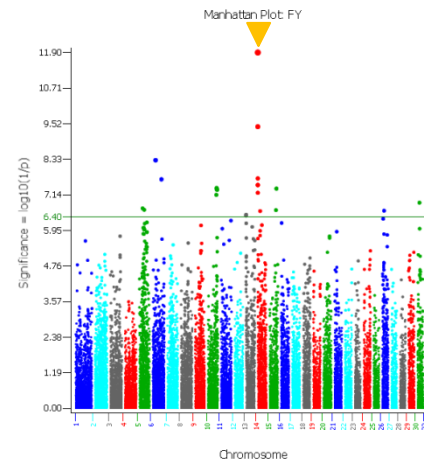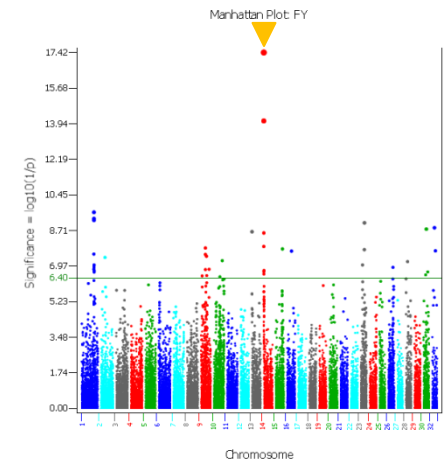

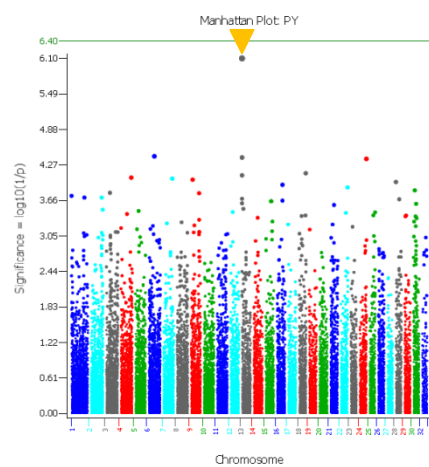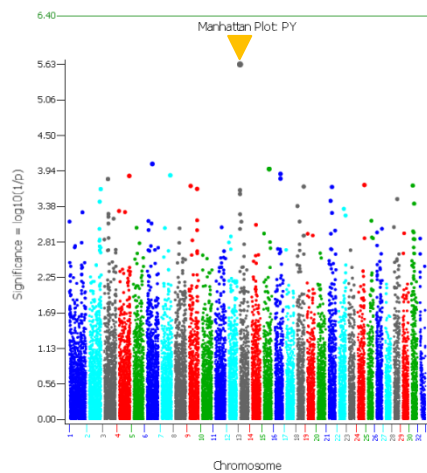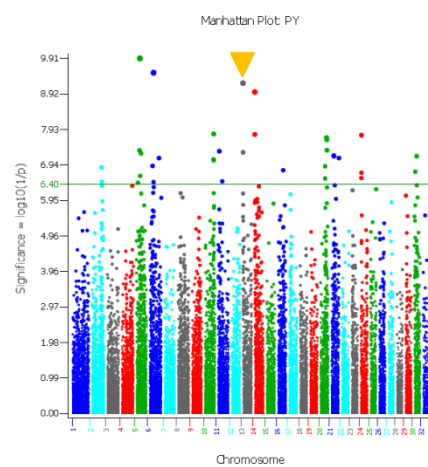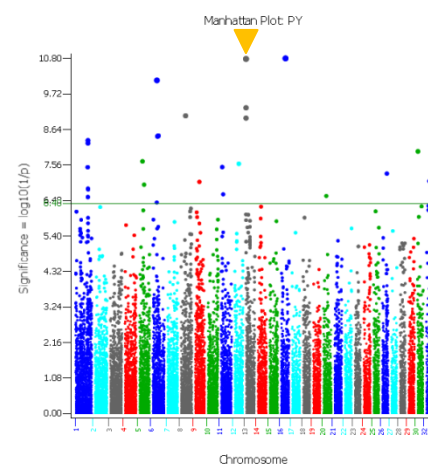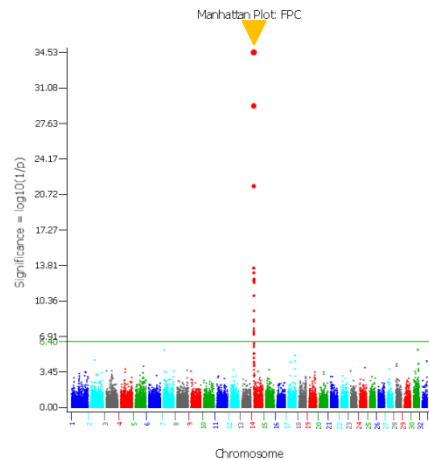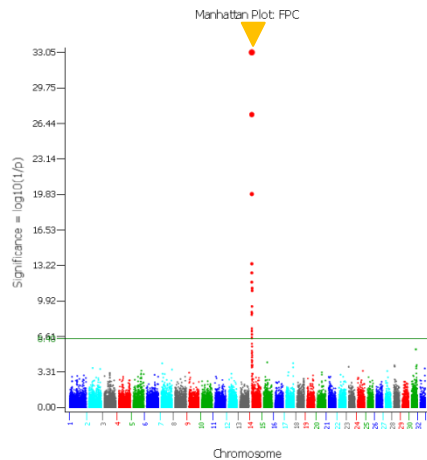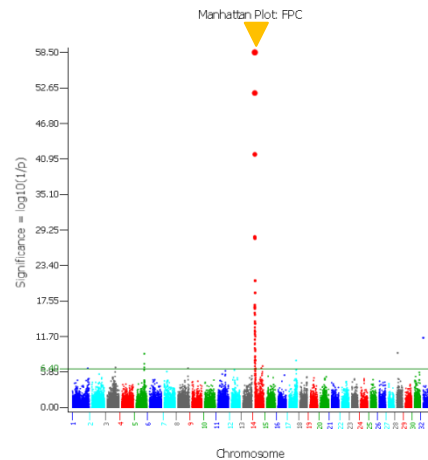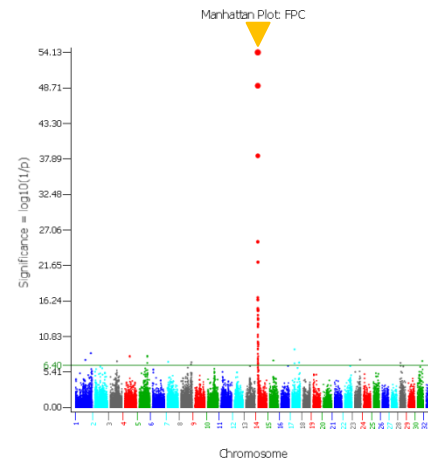

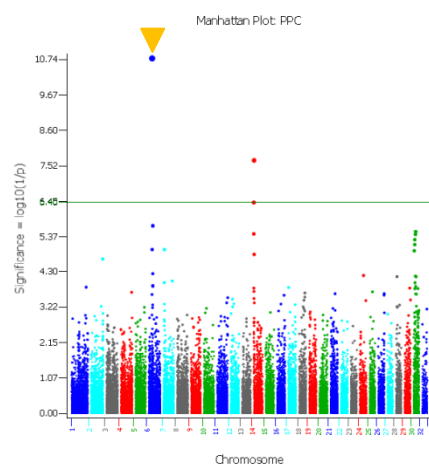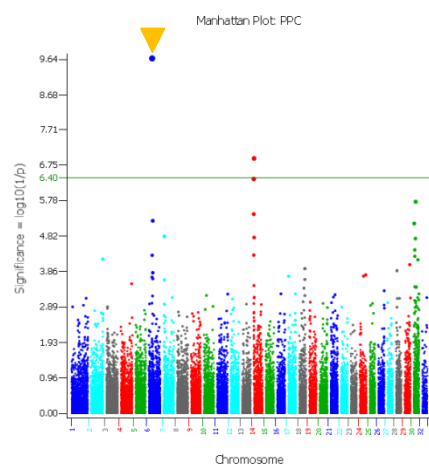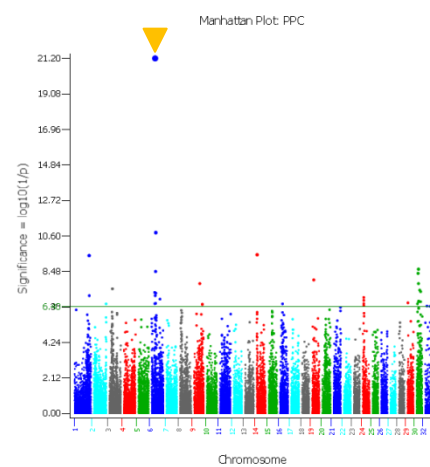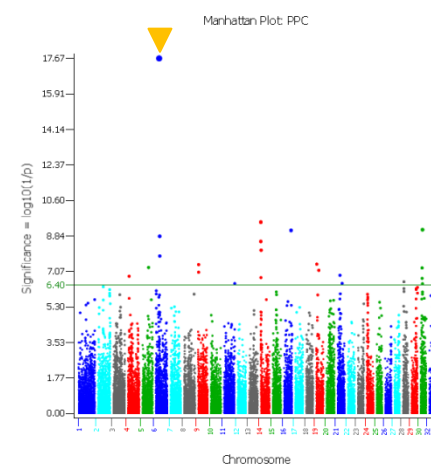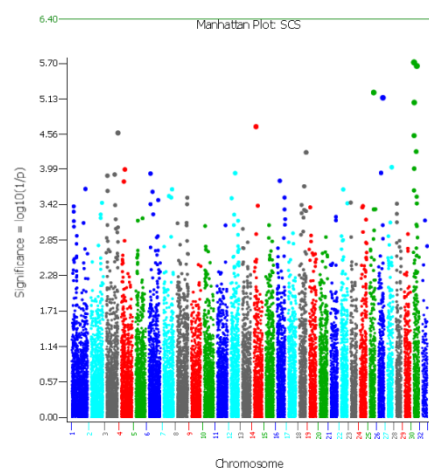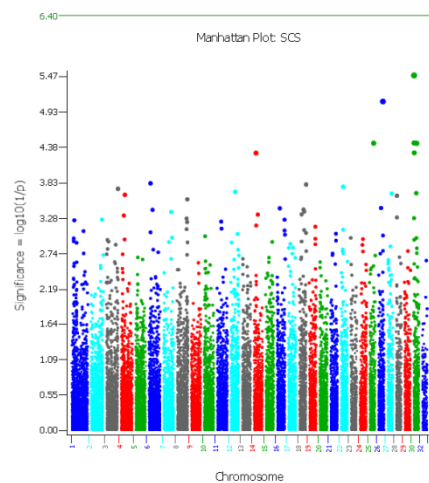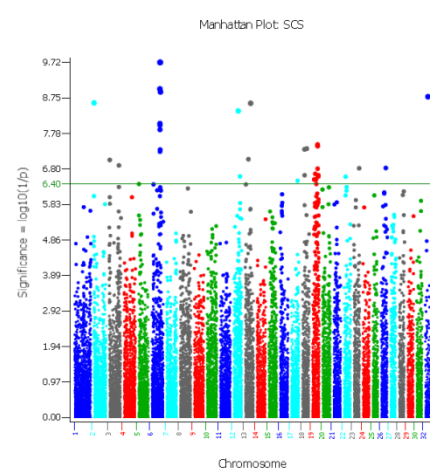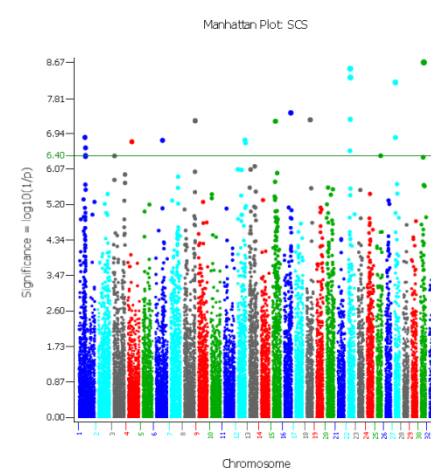

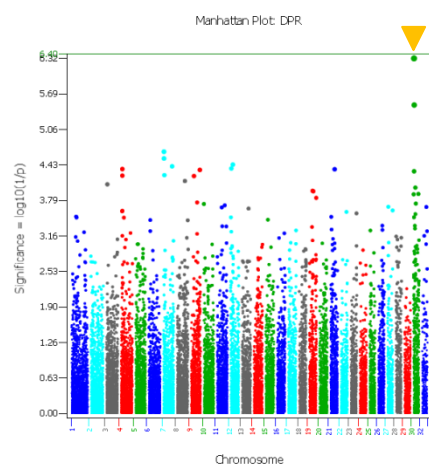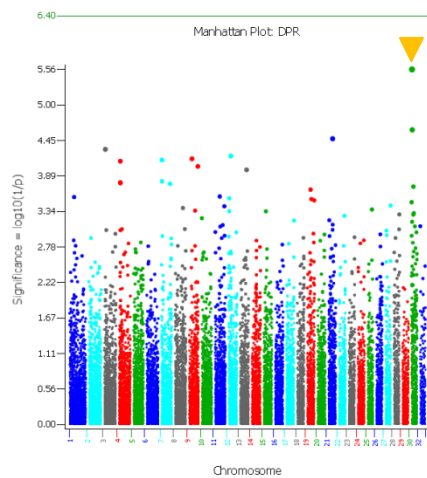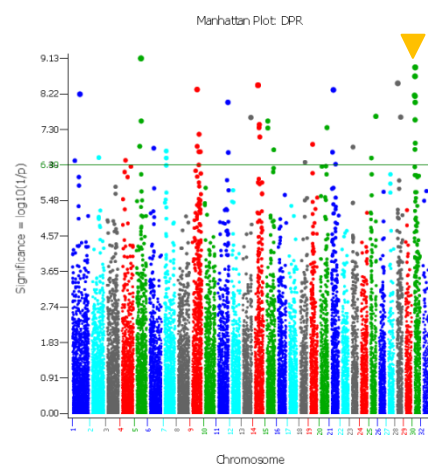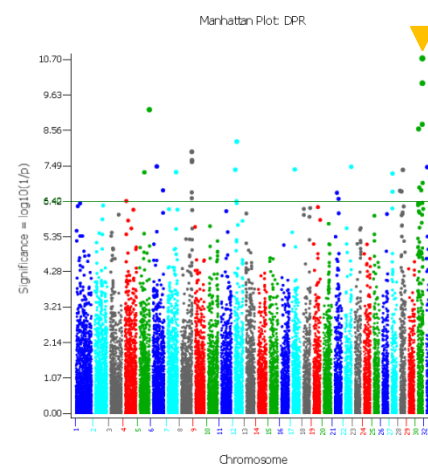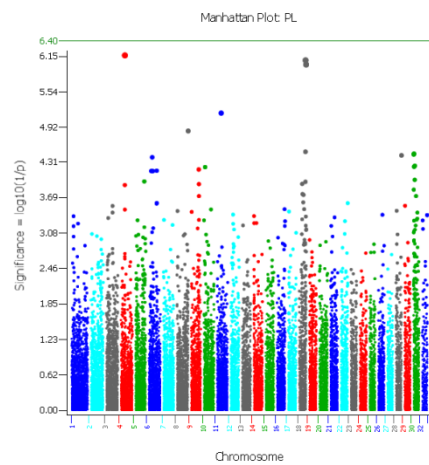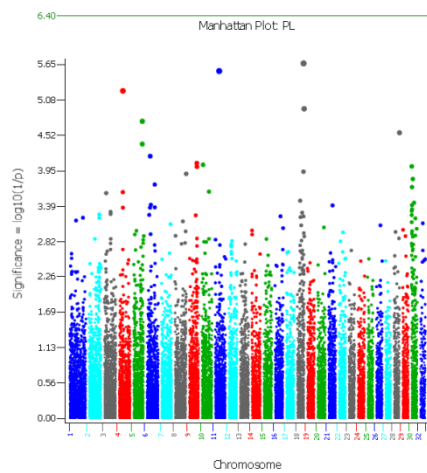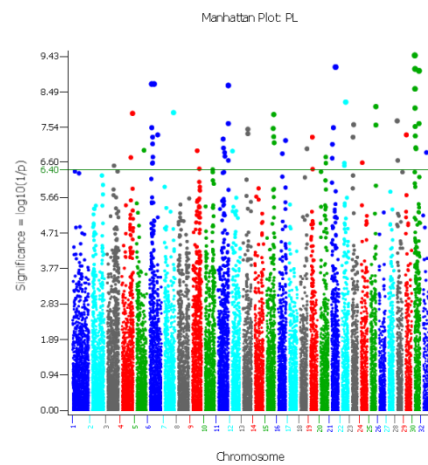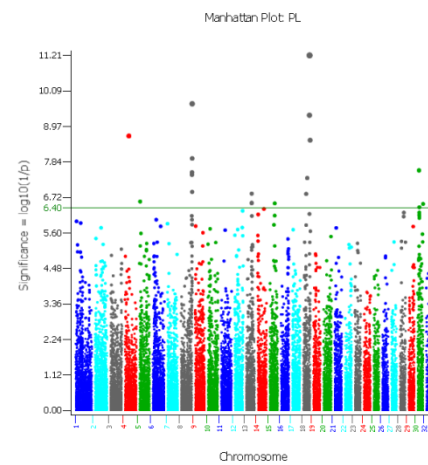

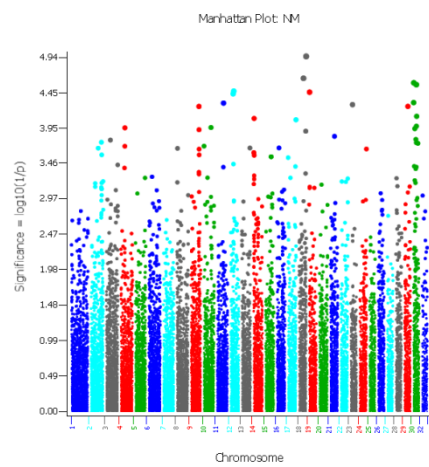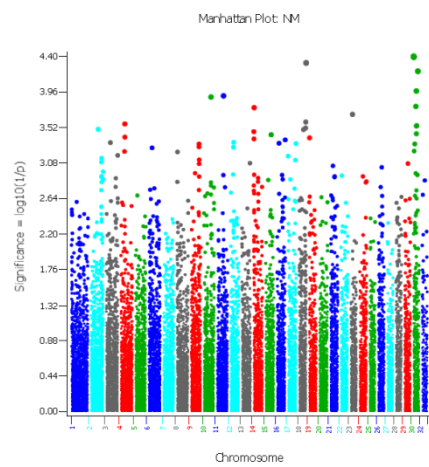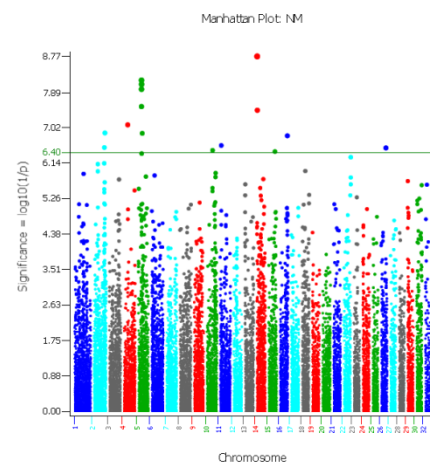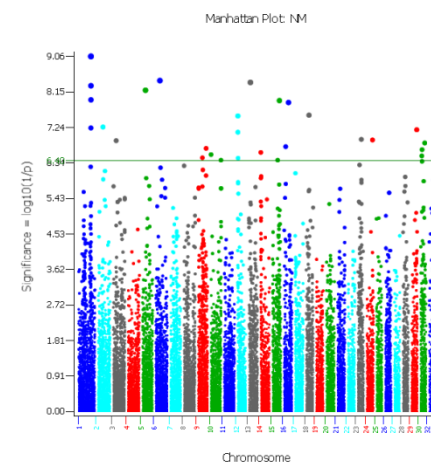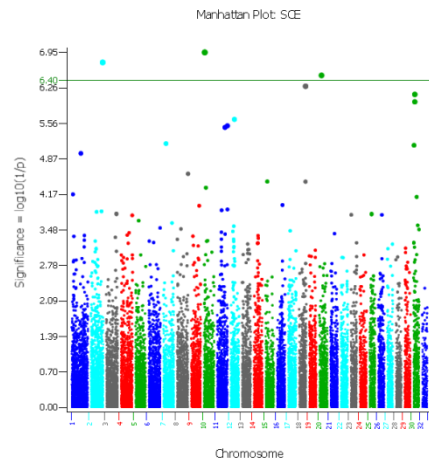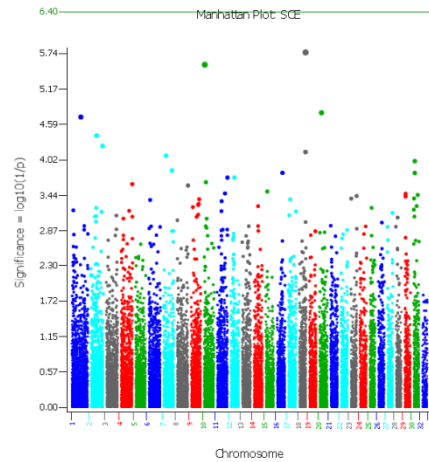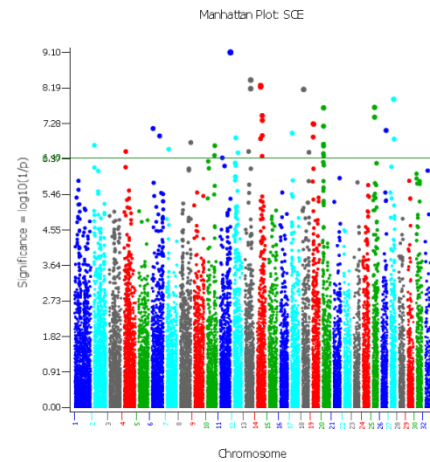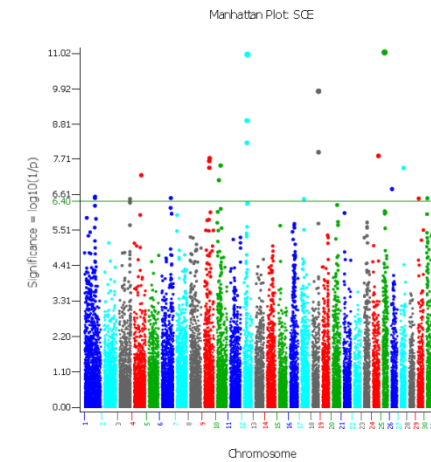

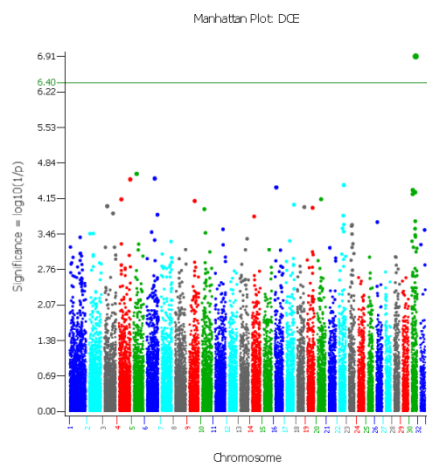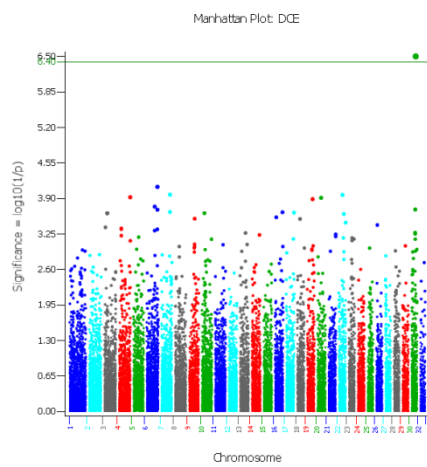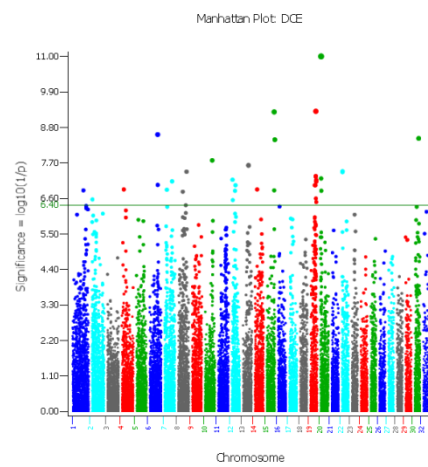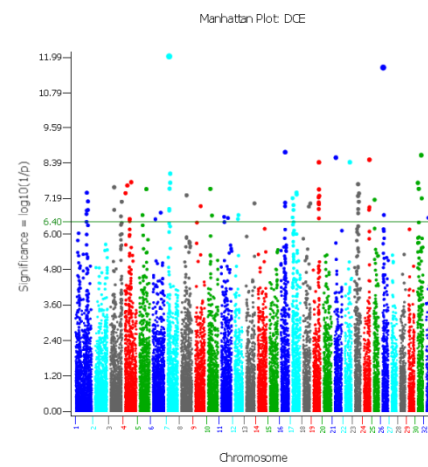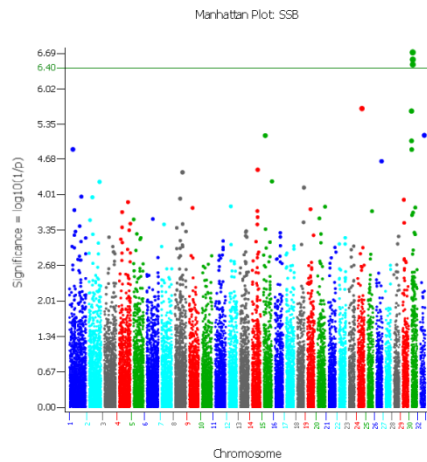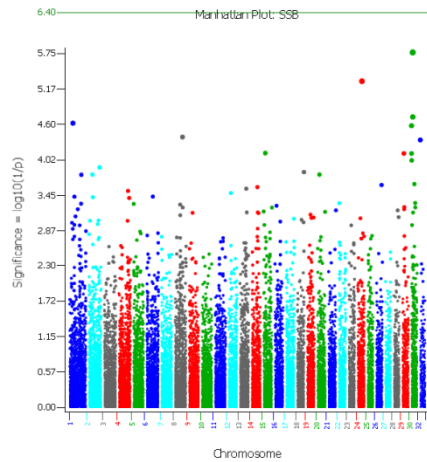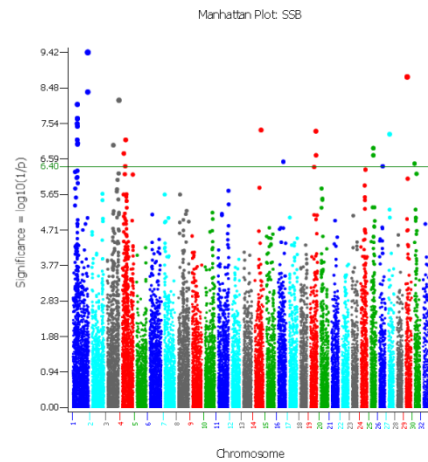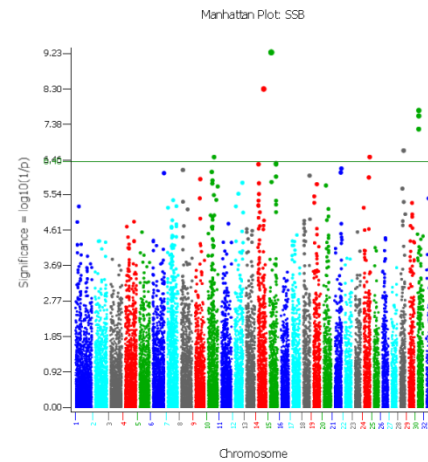

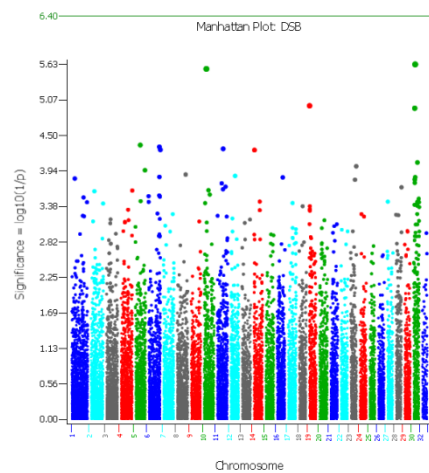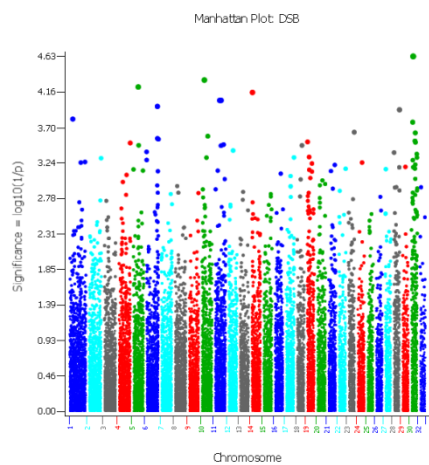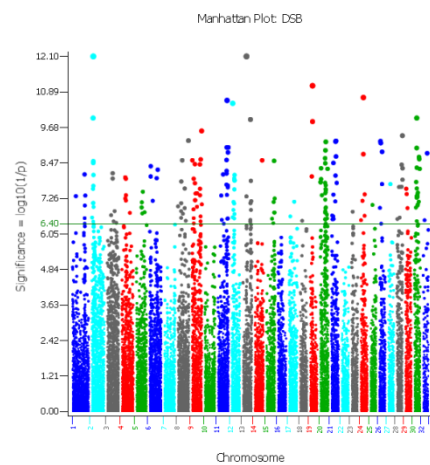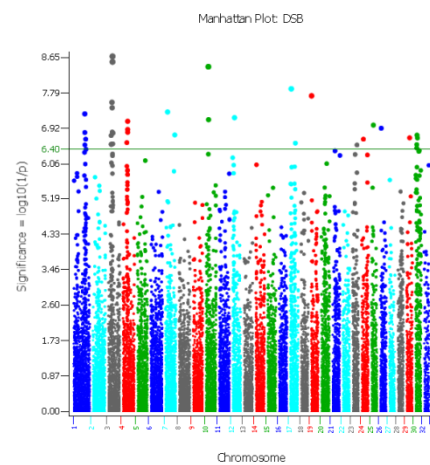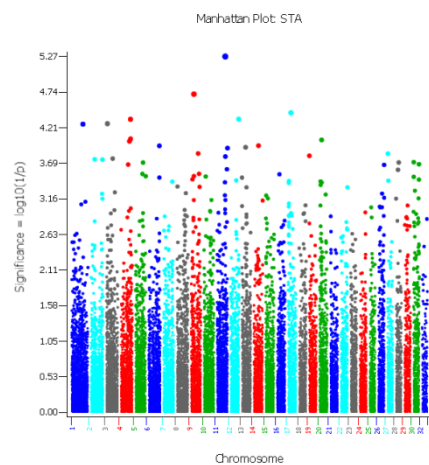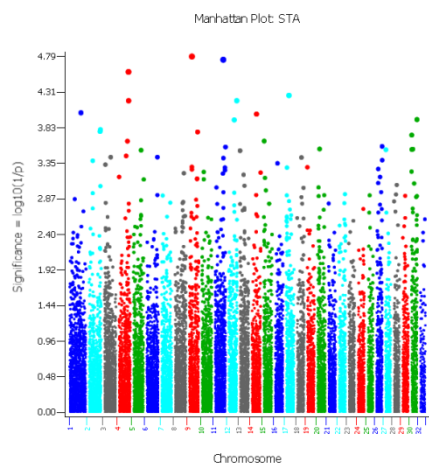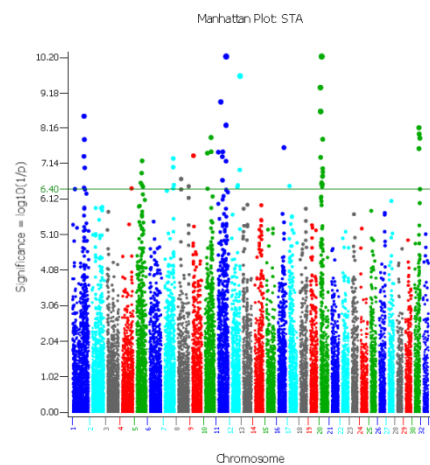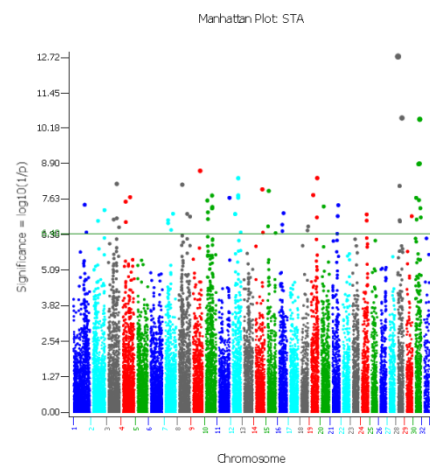

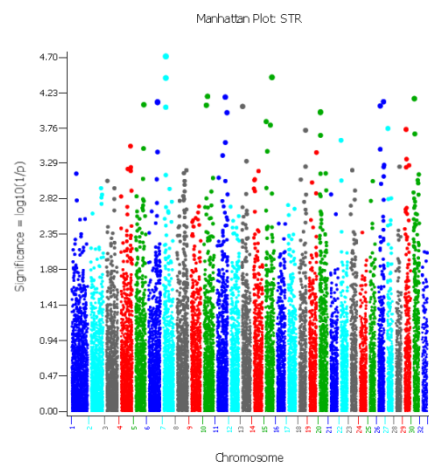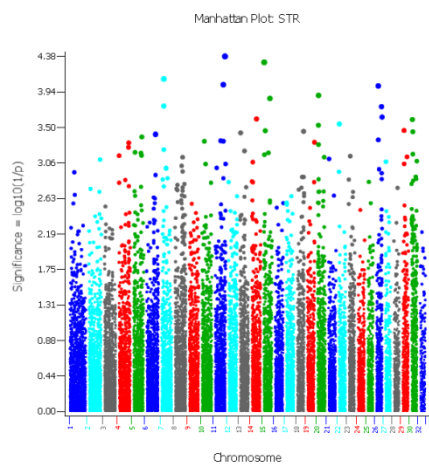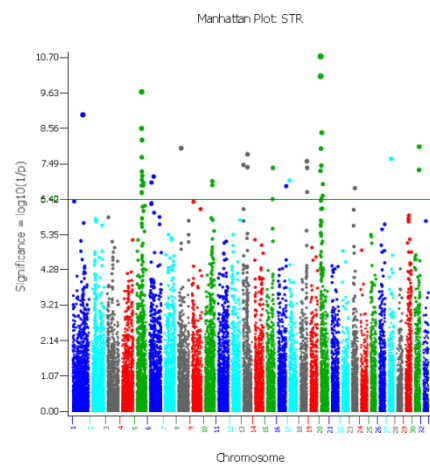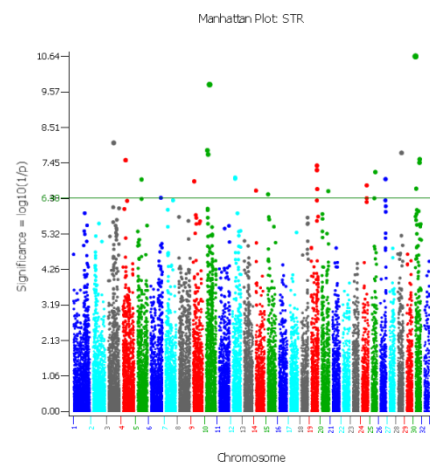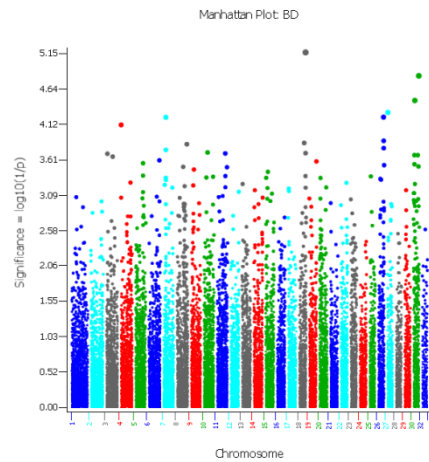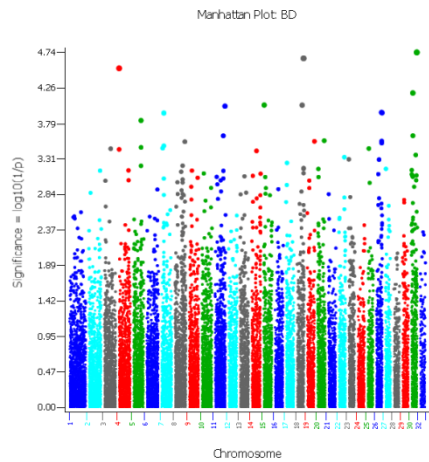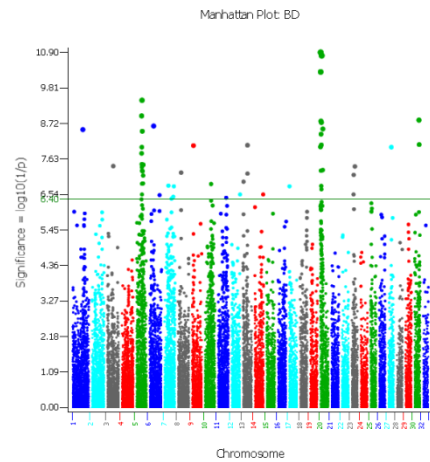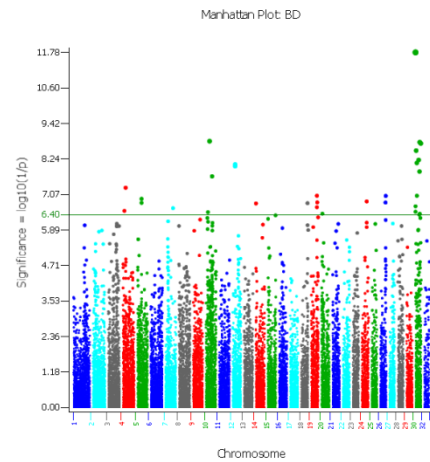

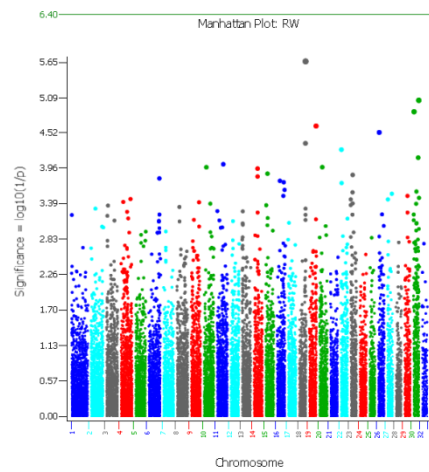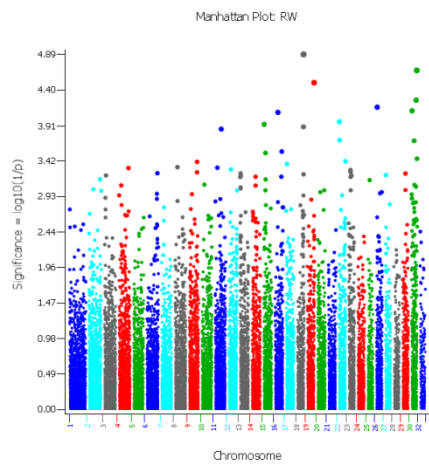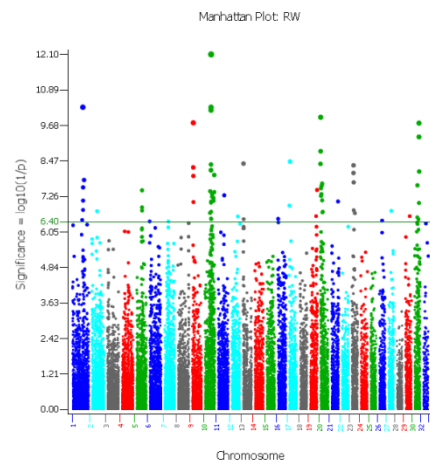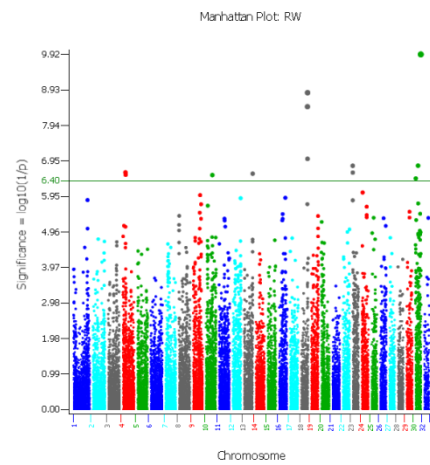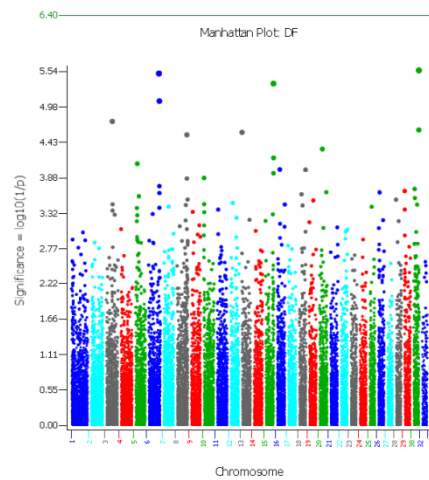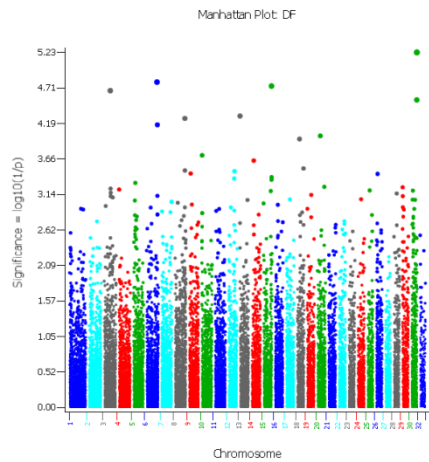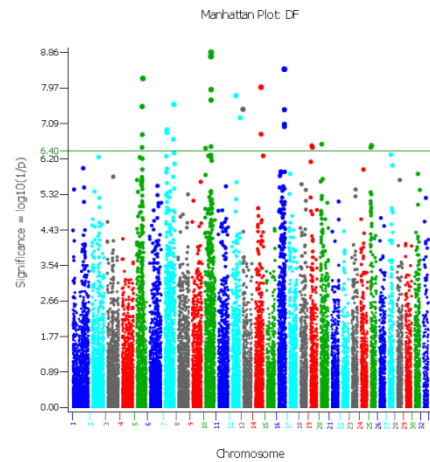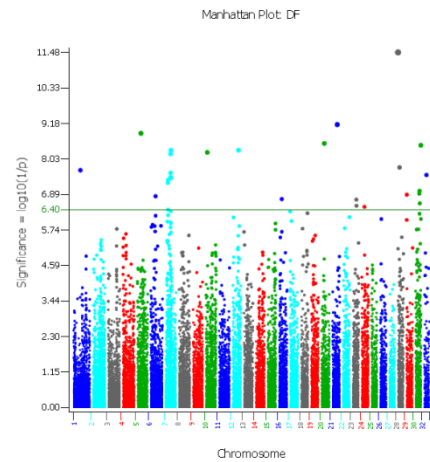

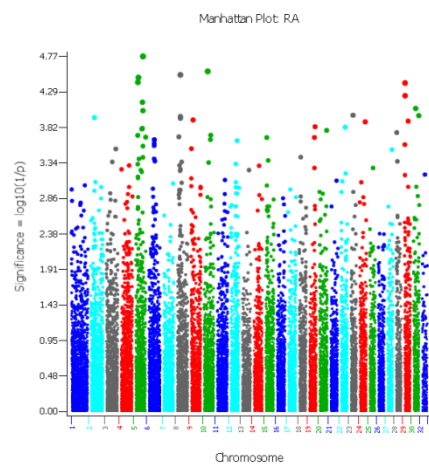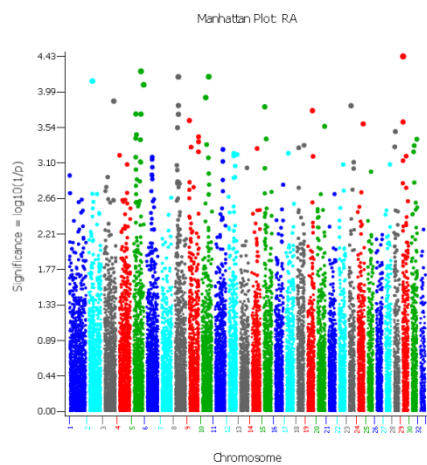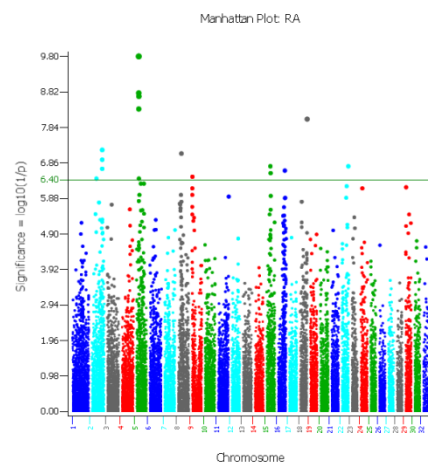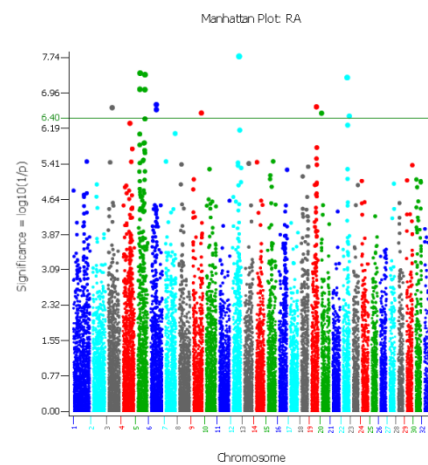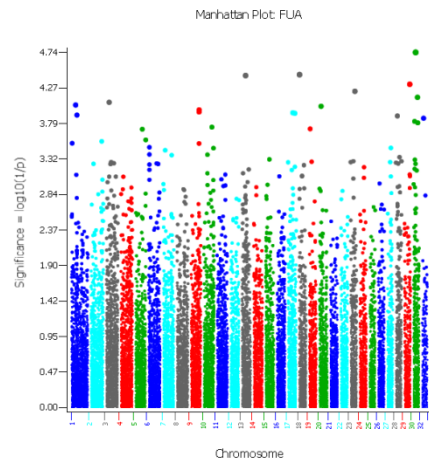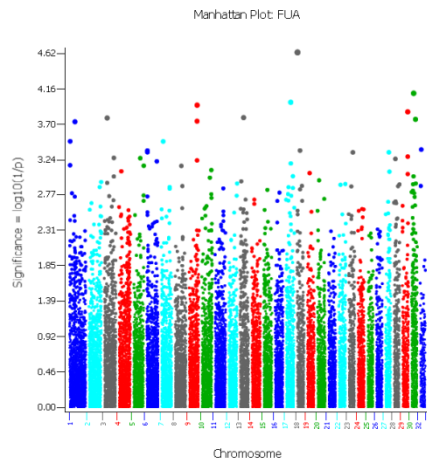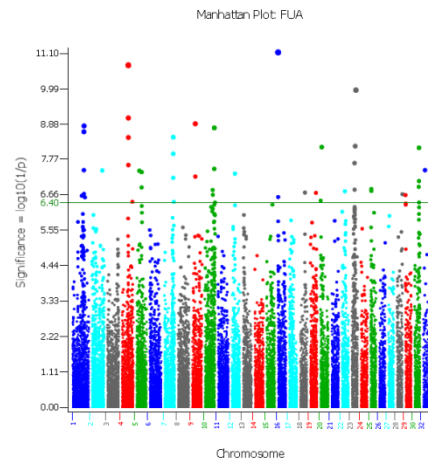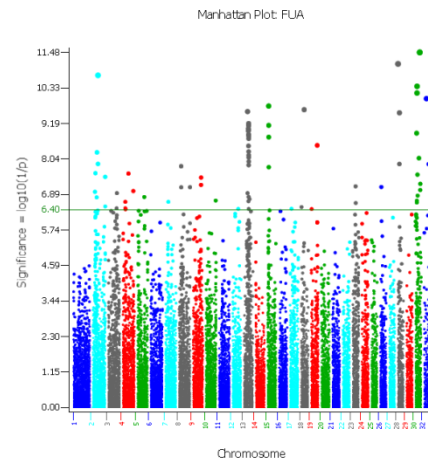

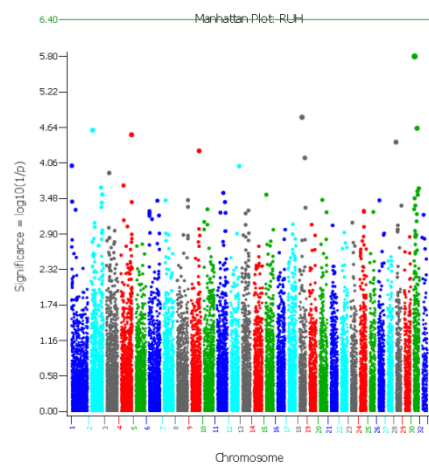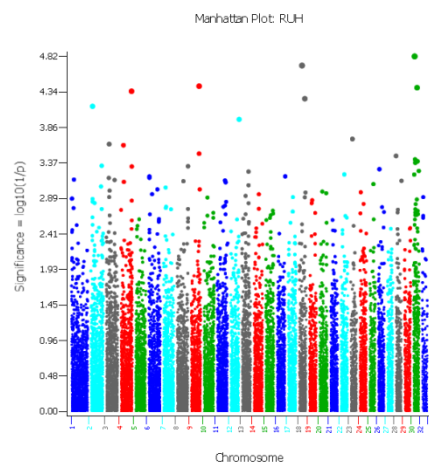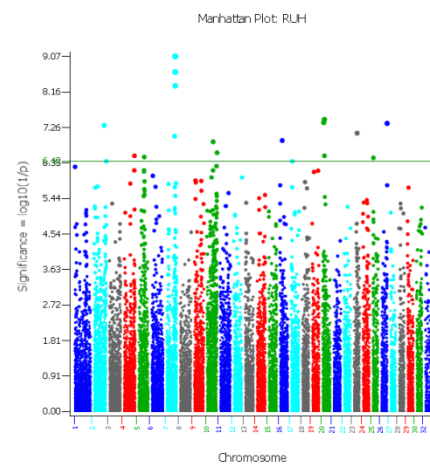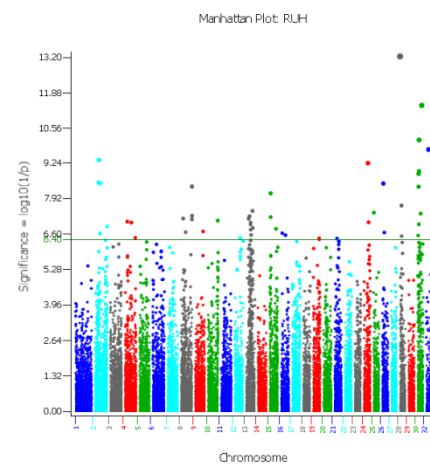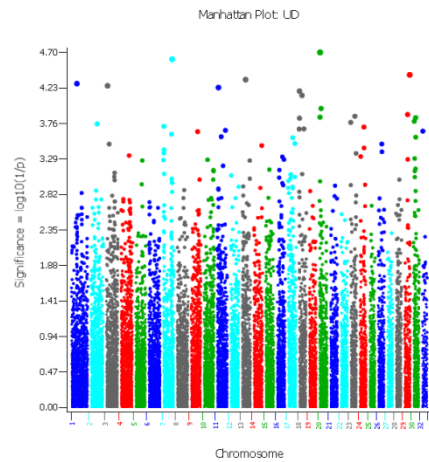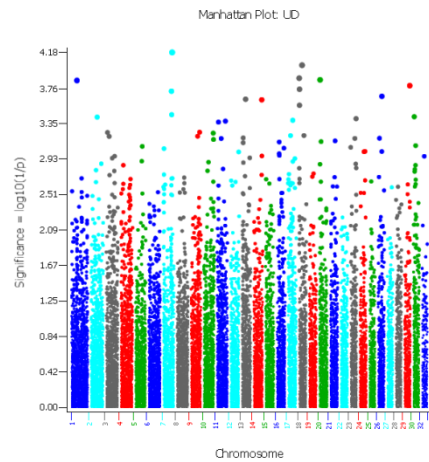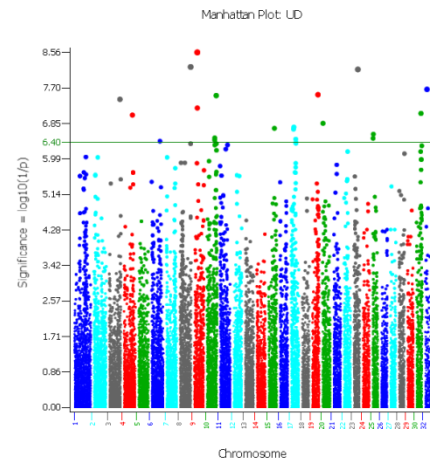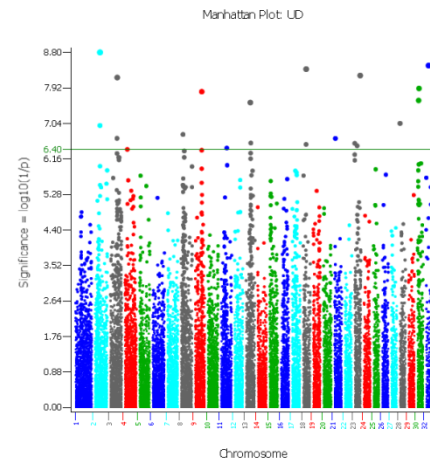

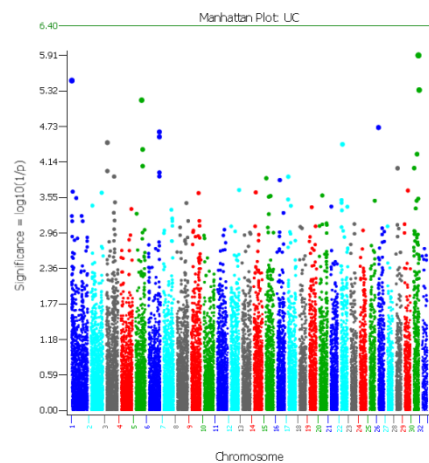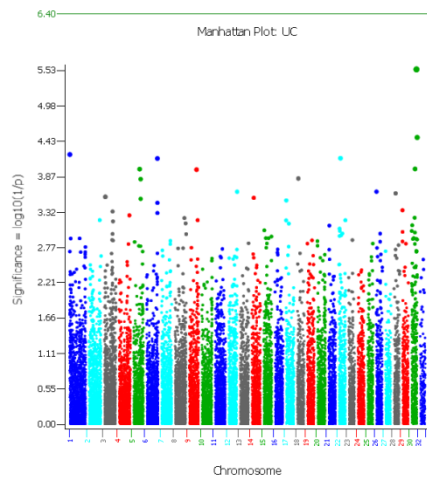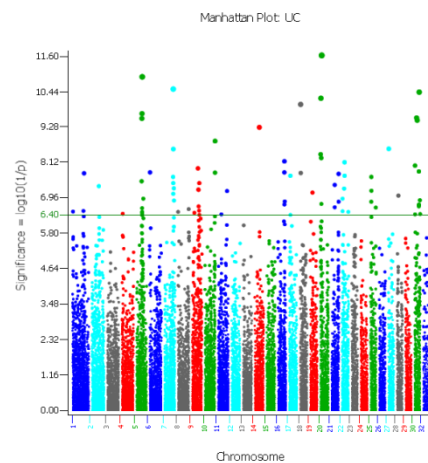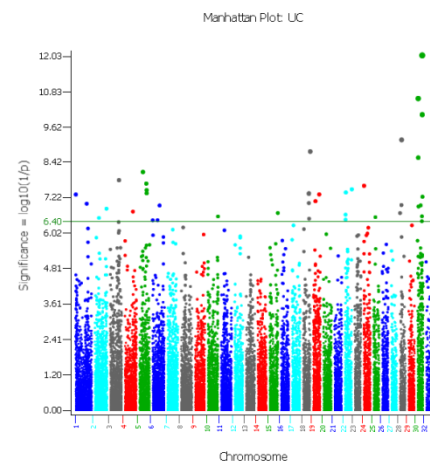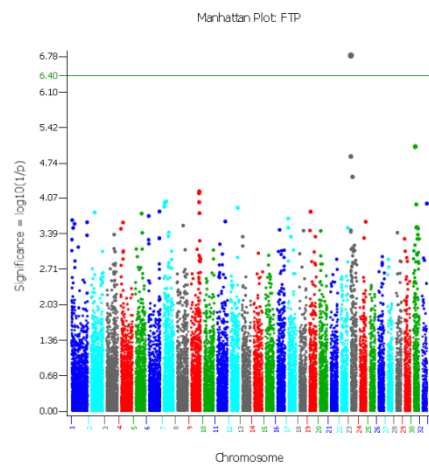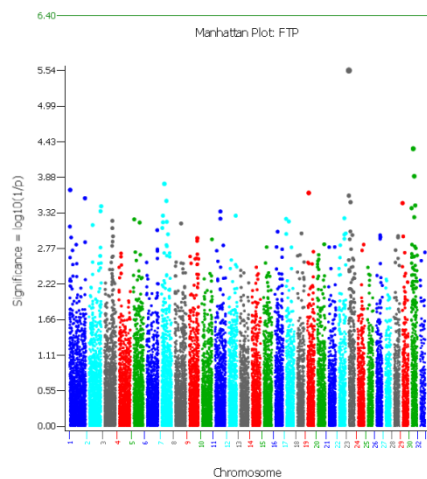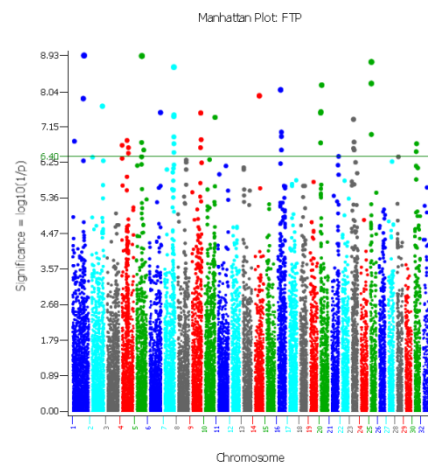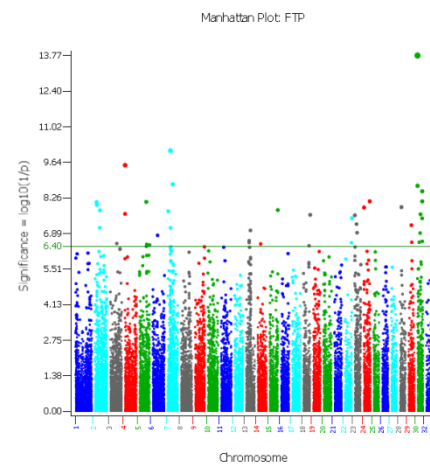

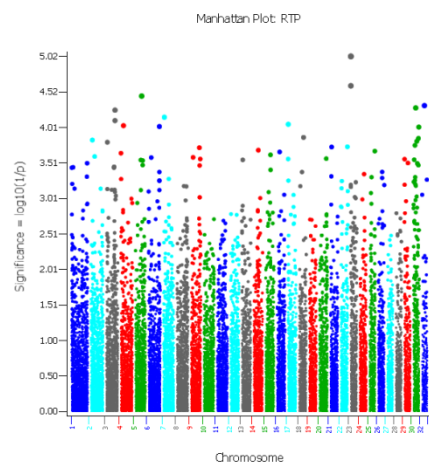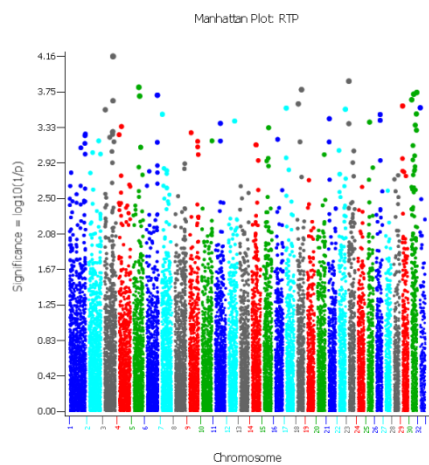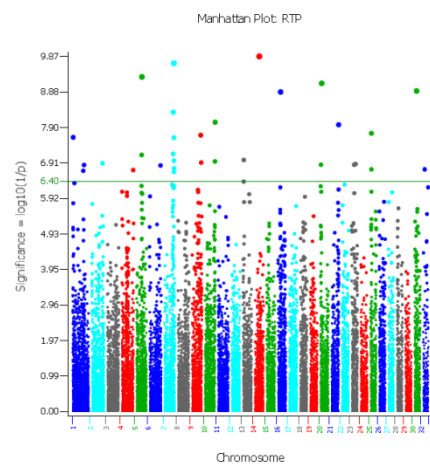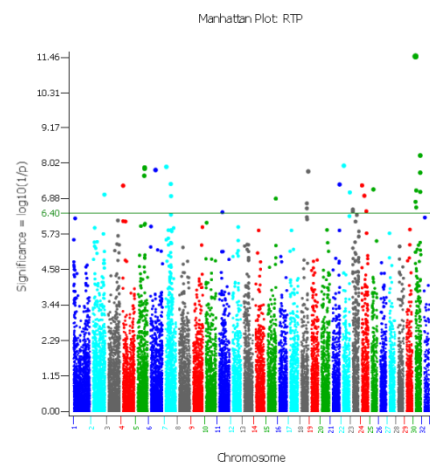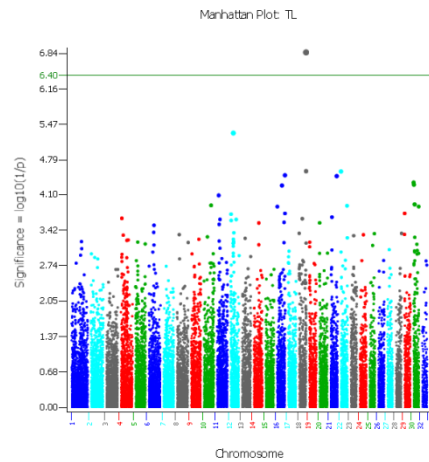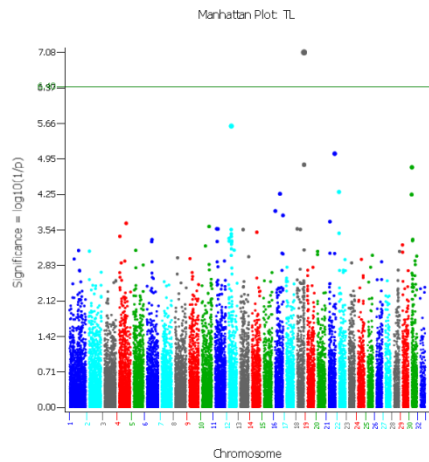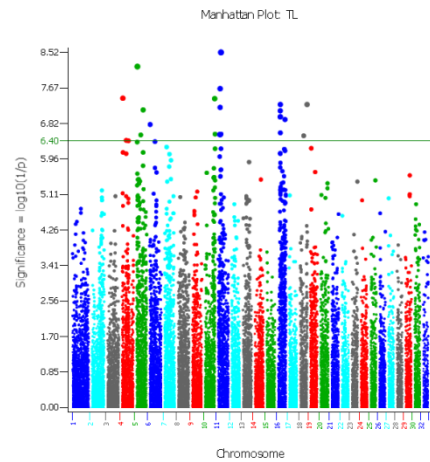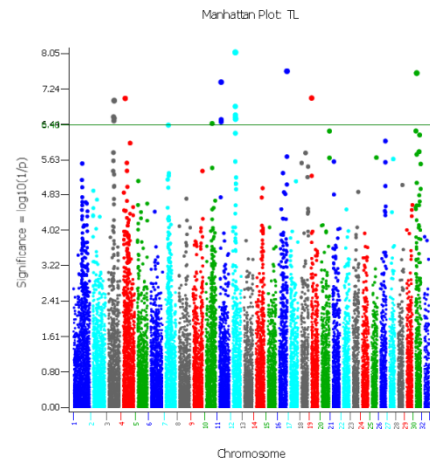

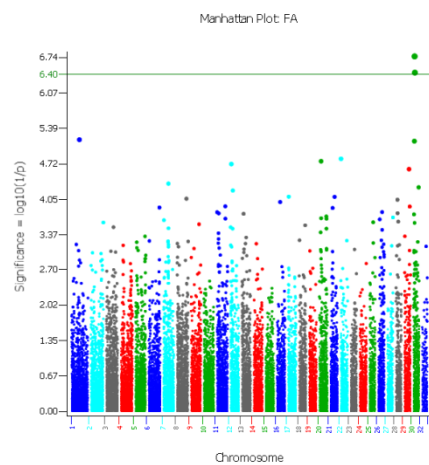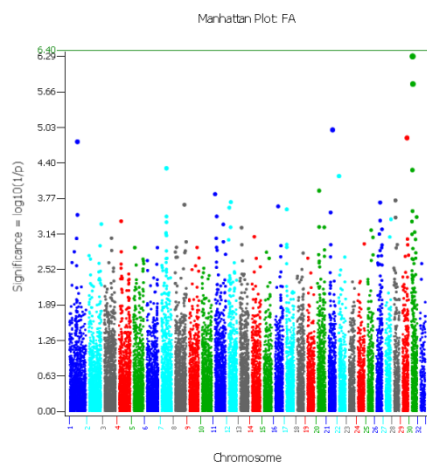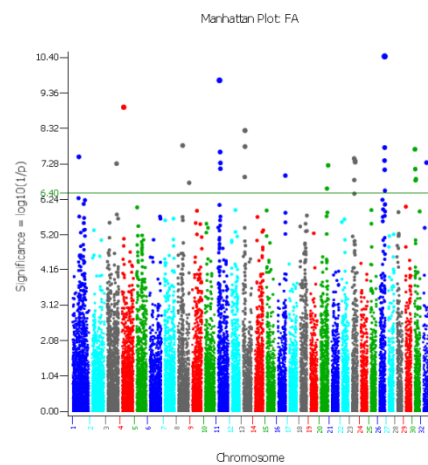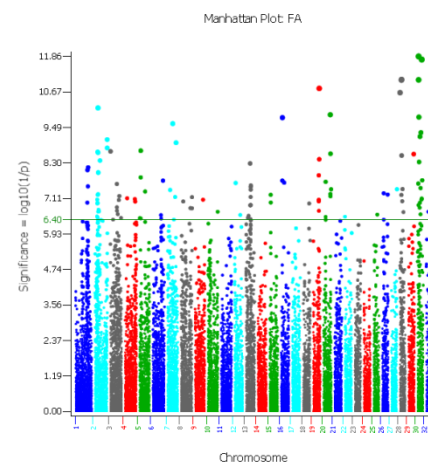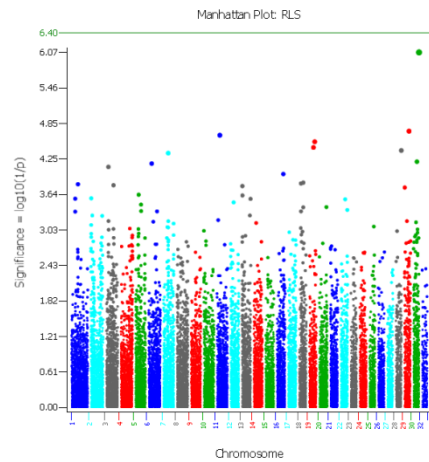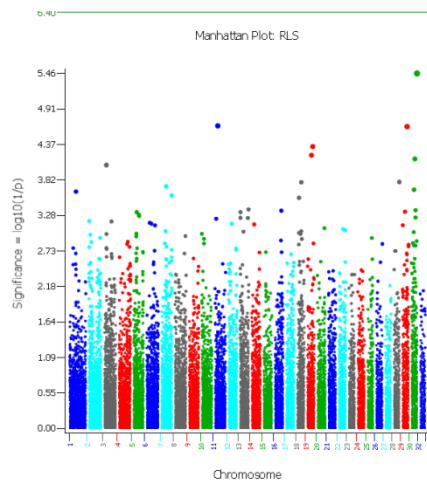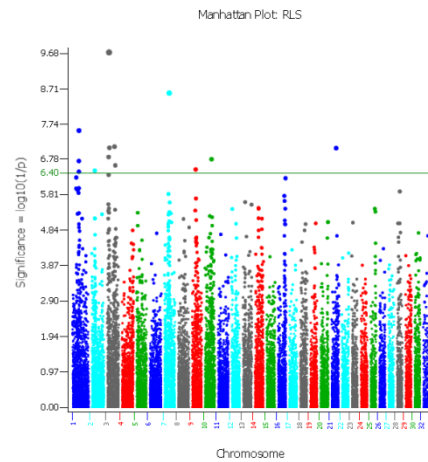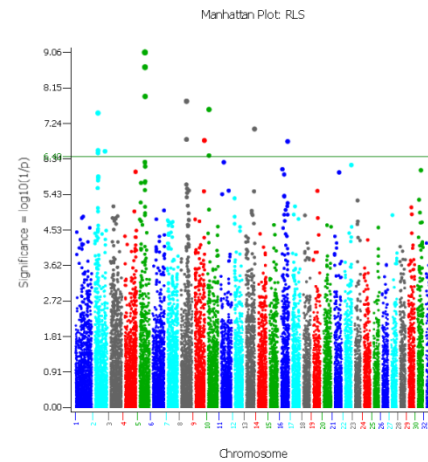

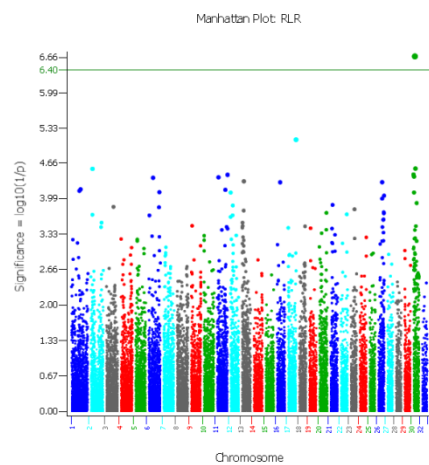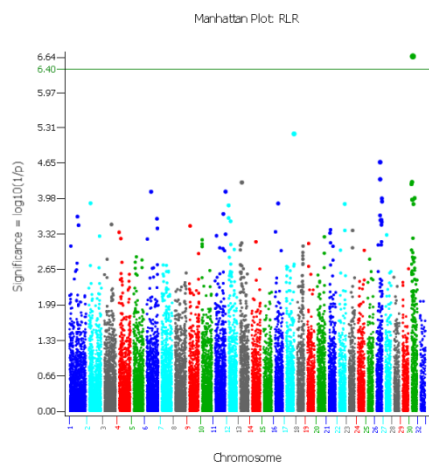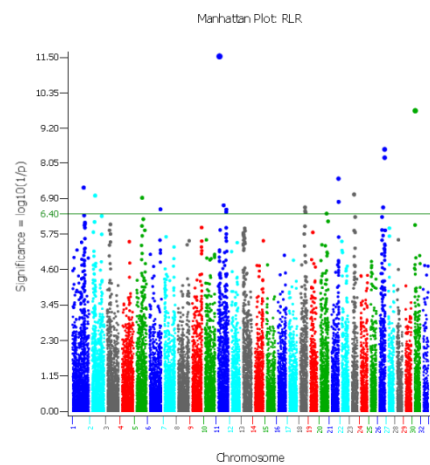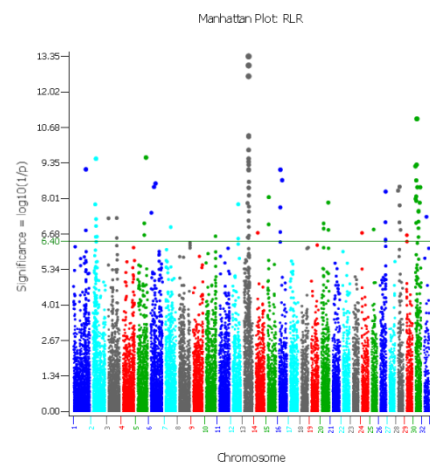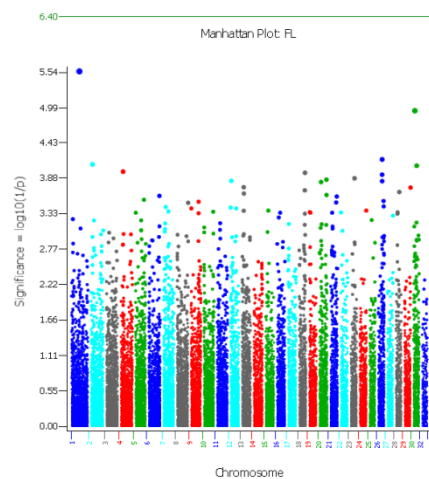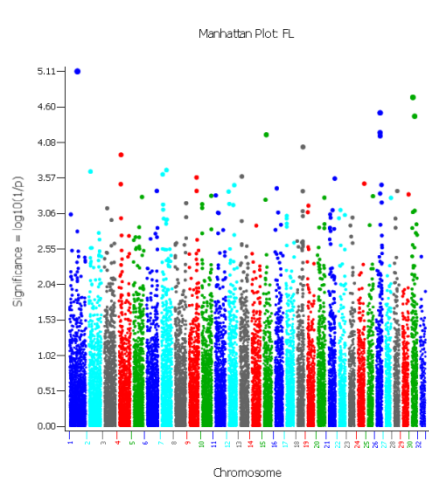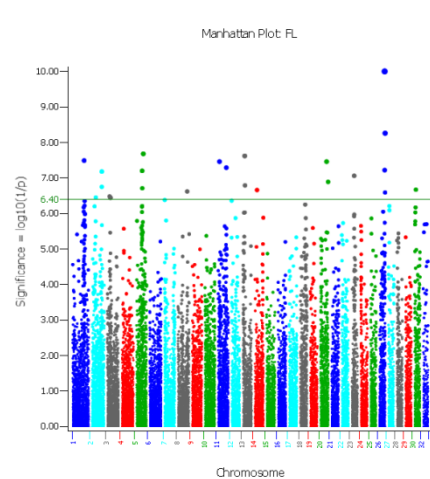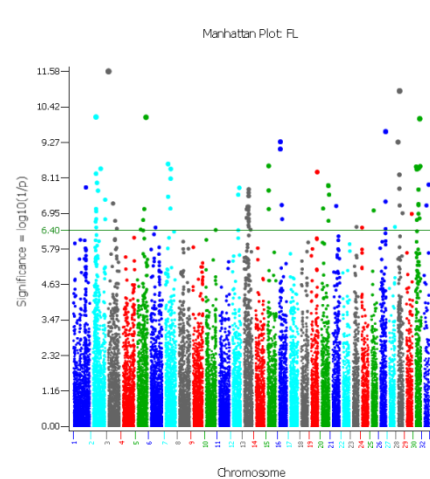

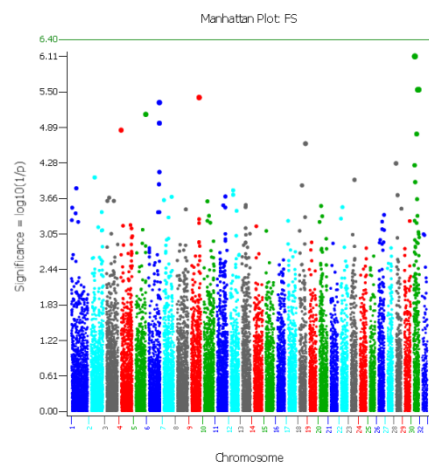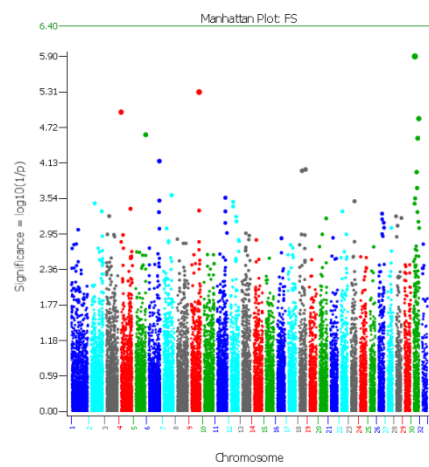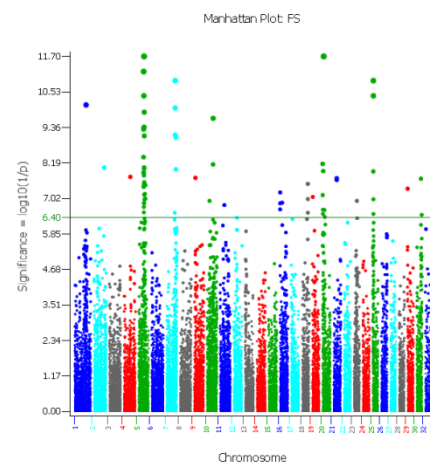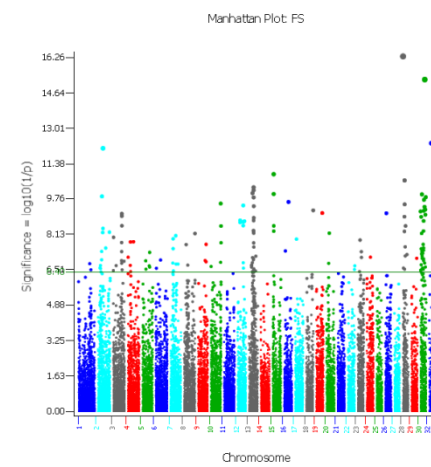

Supplement: Additional file 6 — Figure S6. Global view of P-values of 45,878 SNP effects per trait for 31 production, health, reproduction and body conformation traits by three methods for stratification correction. MY, milk yield; FY, fat yield; PY, protein yield; FPC, fat percentage; PPC, protein percentage; PL, productive life; SCS, somatic cell score; DPR, daughter pregnancy rate; SCE, service-sire calving ease; DCE, daughter calving ease; SSB, service-sire stillbirth; DSB, daughter stillbirth; NM, net merit; STA, stature; STR, strength; BD, body depth; DF, dairy form; RA, rump angle; RW, rump width; FUA, fore udder attachment; RUH, rear udder height; UD, udder depth; UC, udder cleft; FTP, front teat placement; RTP, rear teat placement; TL, teat length; FA, foot angle; RLS, rear legs (side view); RLR, rear legs (rear view); FL, feet and legs; FS, final score. Yellow triangle indicates confirmation among all for methods for stratification correction. [file 1471-2164-13-536-S6.pdf]
