# Supplementary material for: Effect of sample stratification on dairy GWAS results
Source: BMC Genomics. 2012 Oct 6;13:536. doi: 10.1186/1471-2164-13-536 (PMC3496570; doi:10.1186/1471-2164-13-536)
Supplement: Additional file 11 — Figure S7. Manhattan plots of the AIPL effect distribution, and results from three sets of analysis: 1) LS, GLS, EMMAX-IBS using the full data set of 1,654 cows; 2) adding PCA to GLS and EMMAX-IBS using 1,654 cows; and 3) LS, GLS and EMMAX using 1,494 cows by removing the 160 elite cows. Red triangle indicates confirmation between effect size and significance test(s). Black triangle indicates confirmation of the AIPL effect by a nearby SNP marker. Black triangle indicates confirmation of the AIPL effect by a nearby SNP marker. Yellow triangle indicates confirmation between EMMAX and GLS. Green triangle indicates eliminated or reduced significance due to add PCA to GLS or EMMAX, or due to removing the 160 elite cows from the analysis. Blue triangle indicates increased significance due to add PCA to GLS or EMMAX, or due to removing the 160 elite cows from the analysis. [file 1471-2164-13-536-S11.pdf]

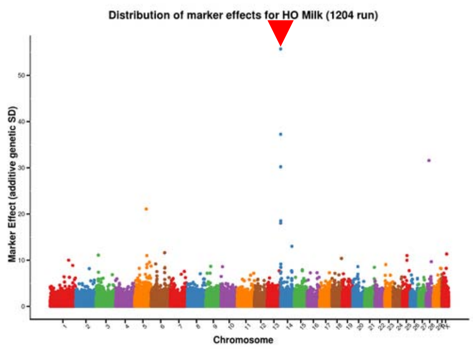

AIPL

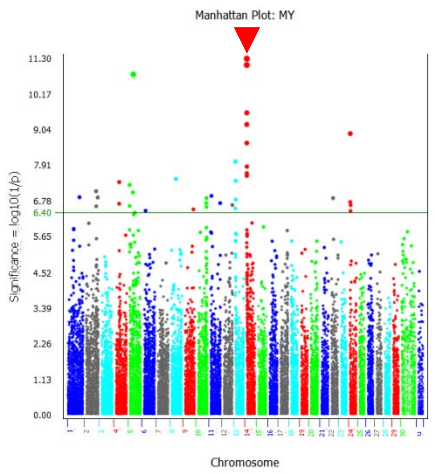

GLS

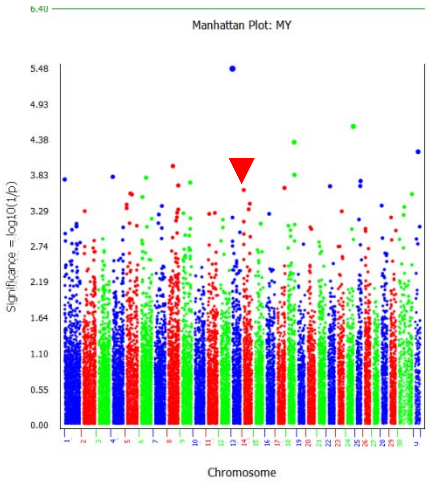

EMMAX-IBS

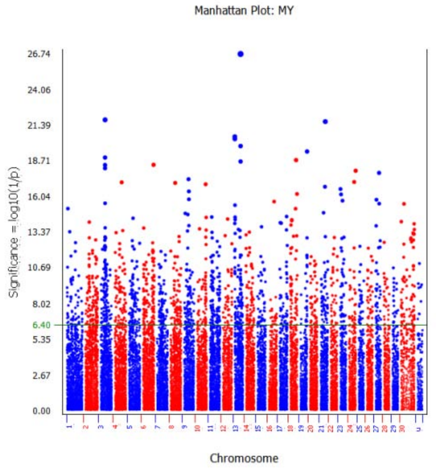

LS

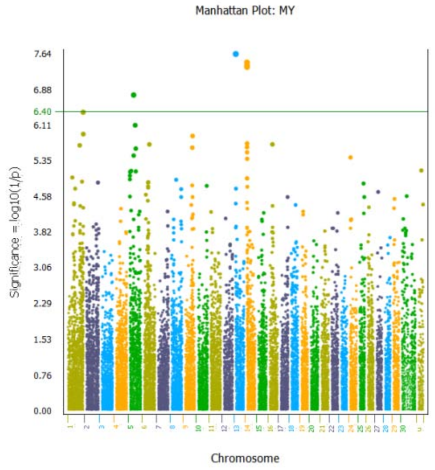

GLS+PCA

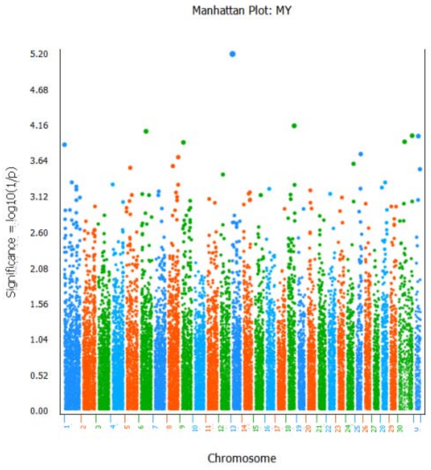

EMMAX+PCA

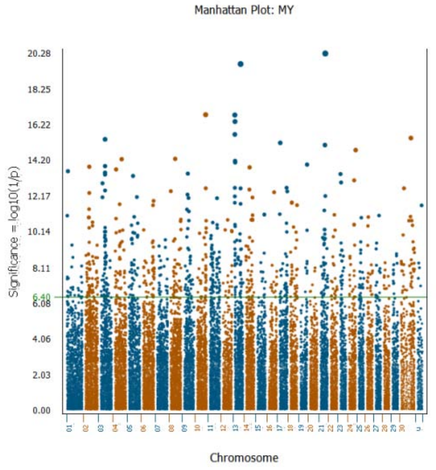

LS_1494

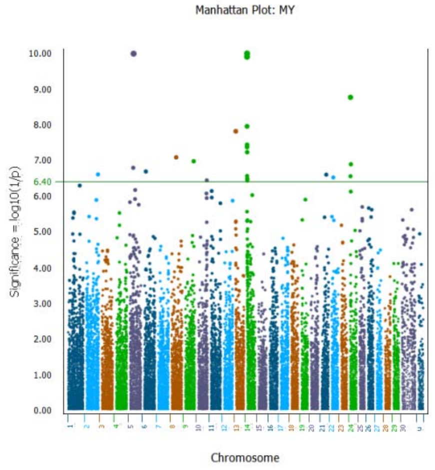

GLS_1494

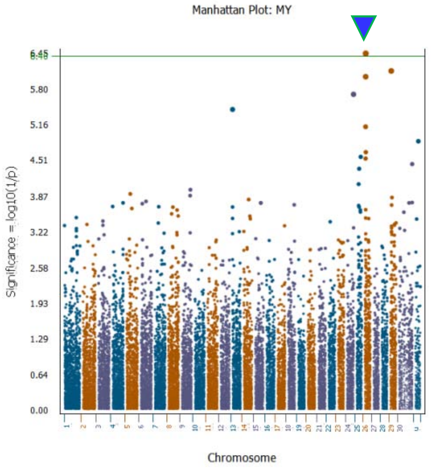

EMMAX_1494

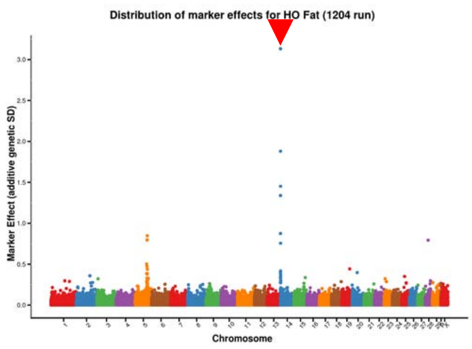

AIPL

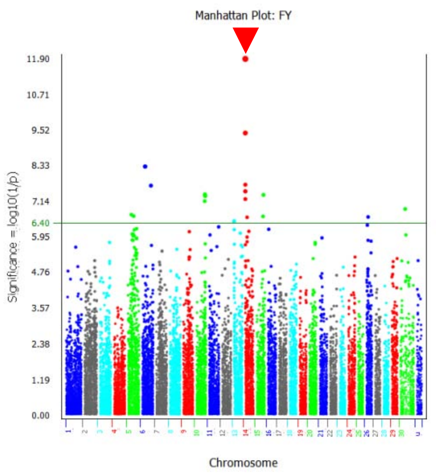

GLS

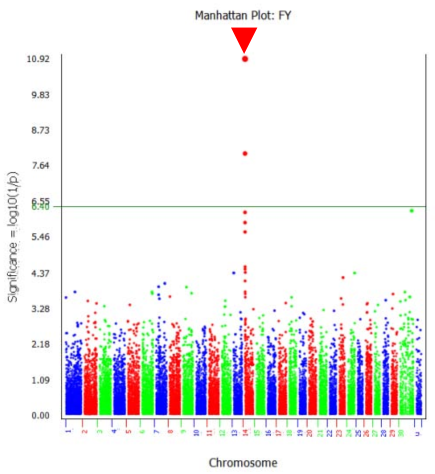

EMMAX-IBS

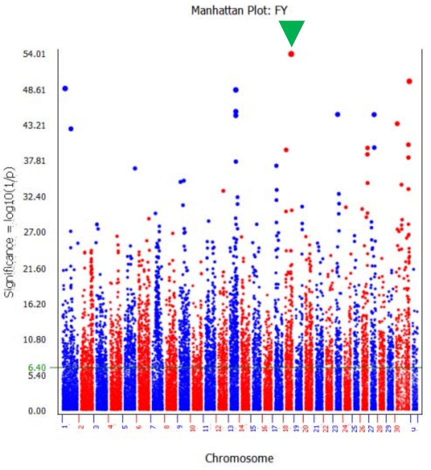

LS

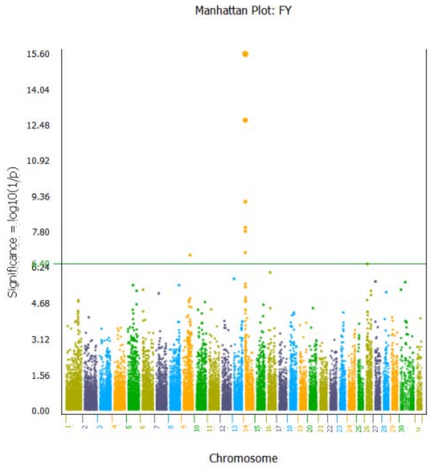

GLS+PCA

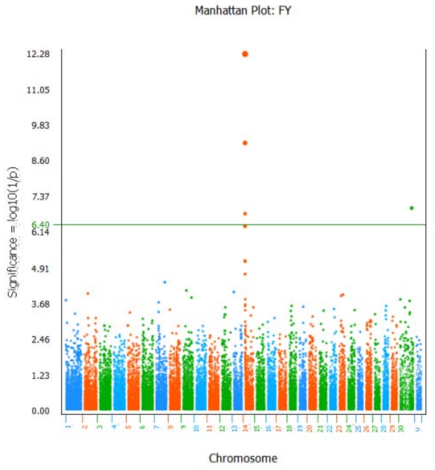

EMMAX+PCA

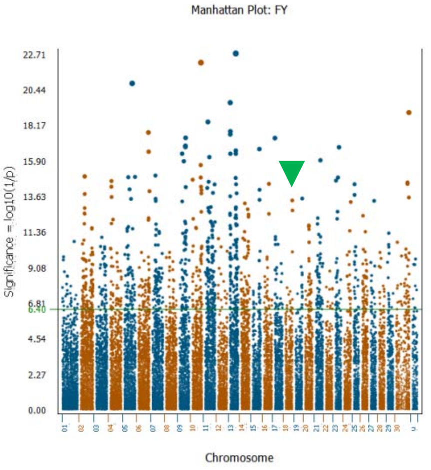

LS_1494

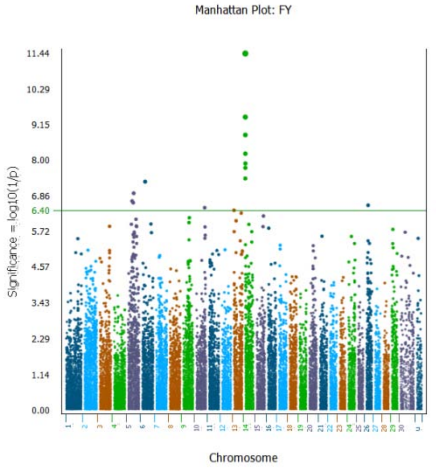

GLS_1494

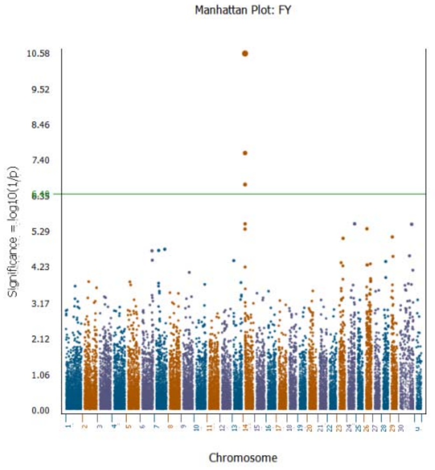

EMMAX_1494

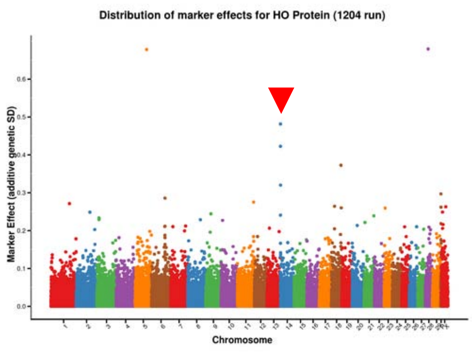

AIPL

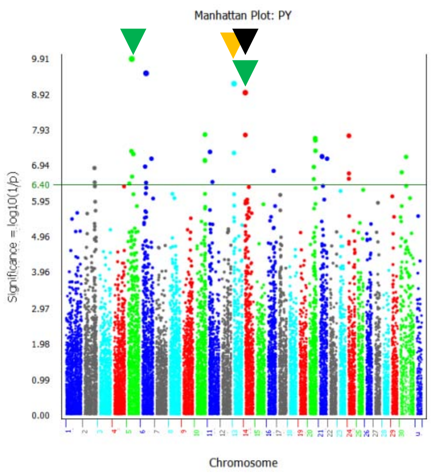

GLS

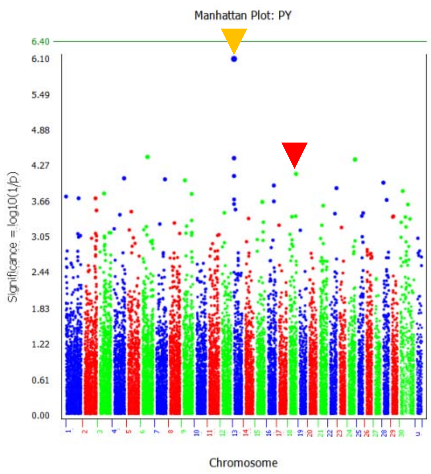

EMMAX-IBS

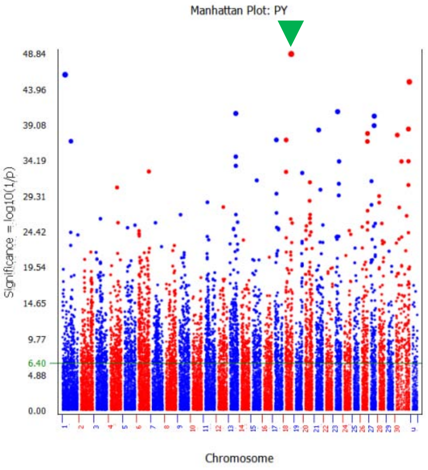

LS

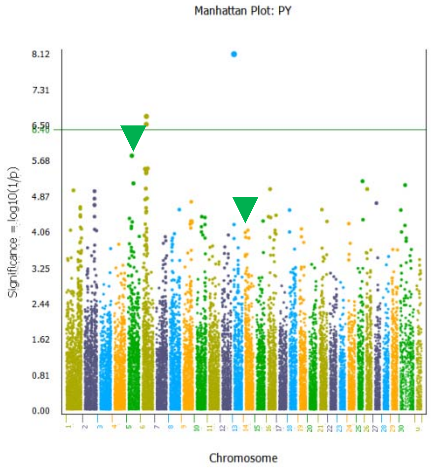

GLS+PCA

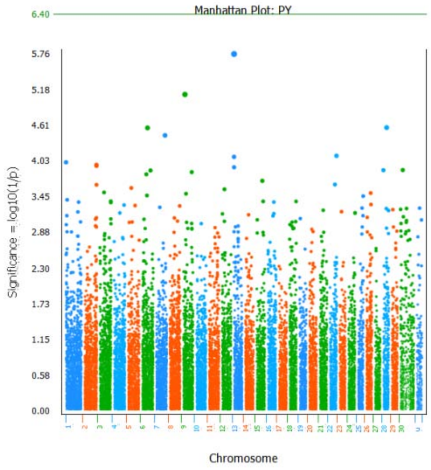

EMMAX+PCA

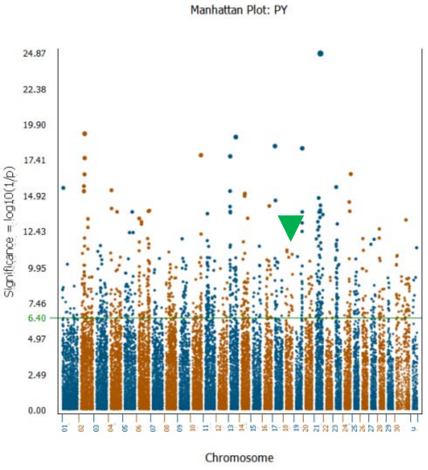

LS_1494

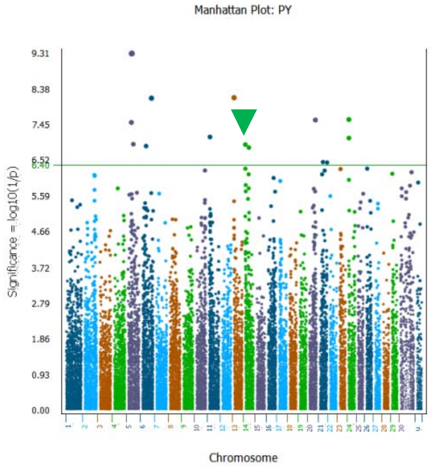

GLS_1494

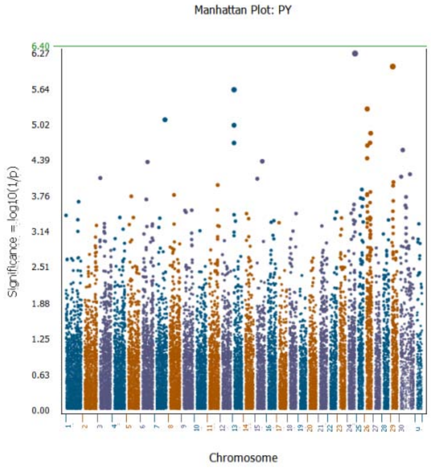

EMMAX_1494

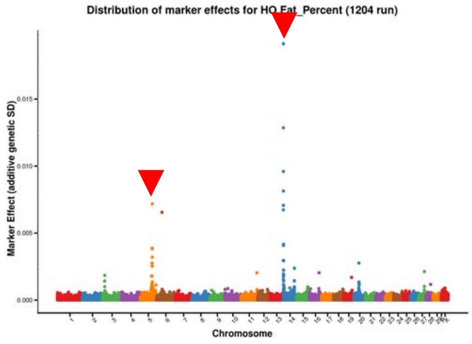

AIPL

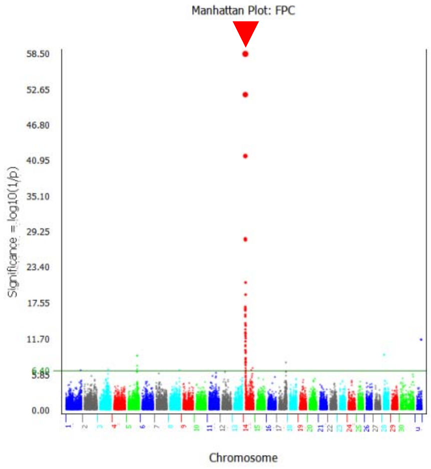

GLS

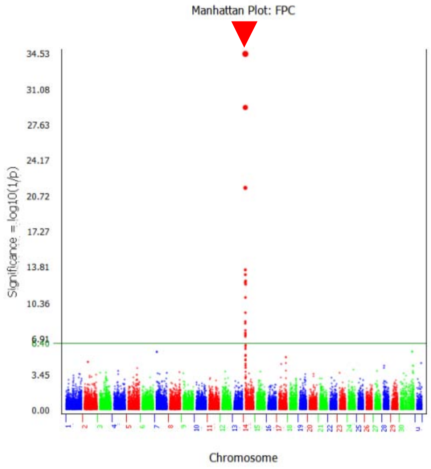

EMMAX-IBS

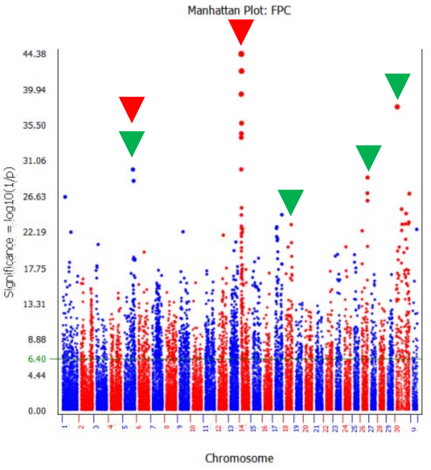

LS

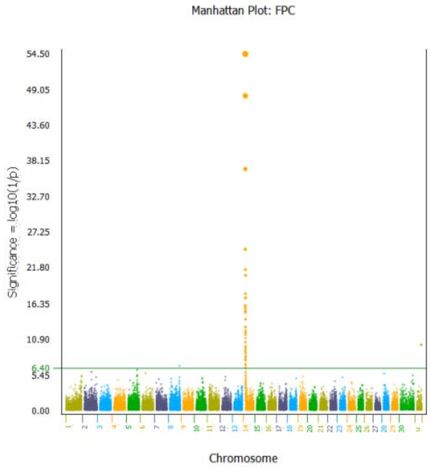

GLS+PCA

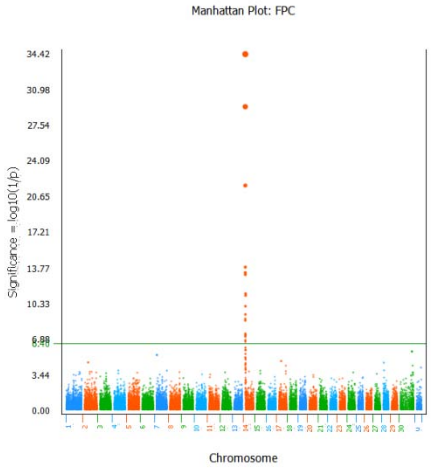

EMMAX+PCA

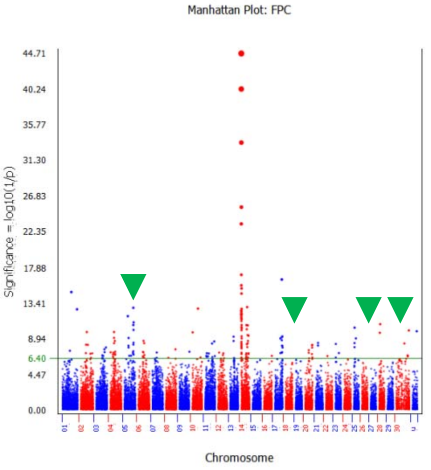

LS_1494

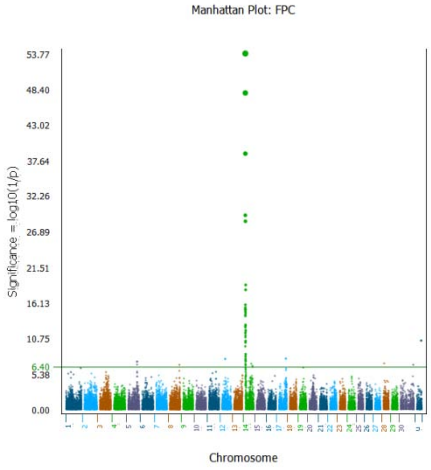

GLS_1494

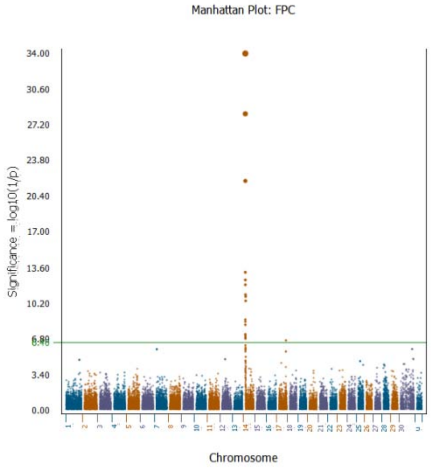

EMMAX_1494

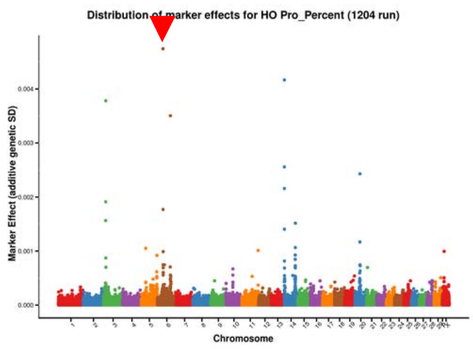

AIPL

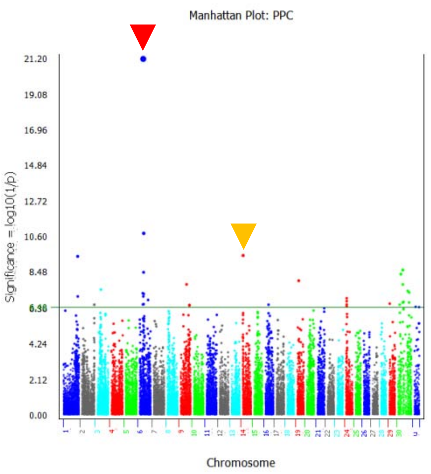

GLS

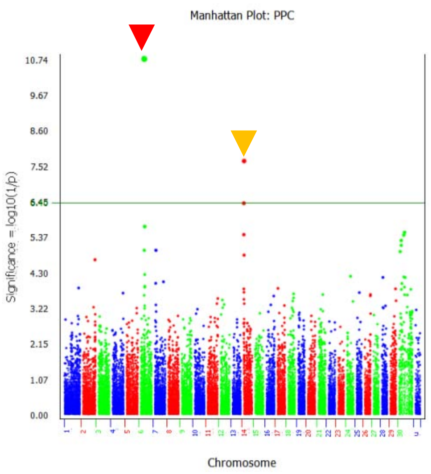

EMMAX-IBS

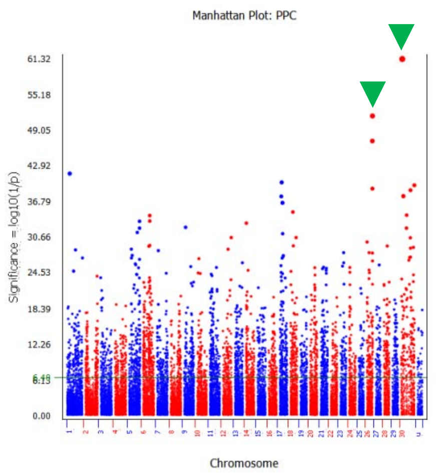

LS

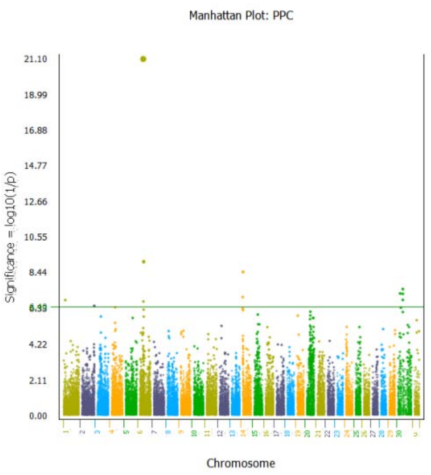

GLS+PCA

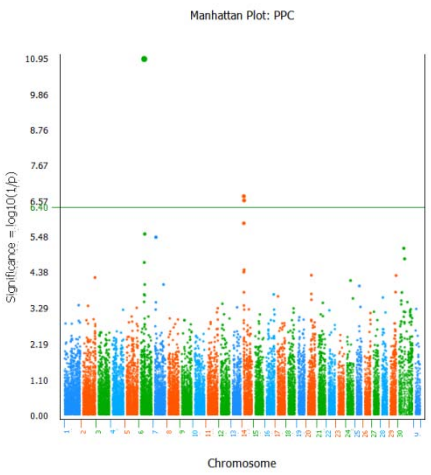

EMMAX+PCA

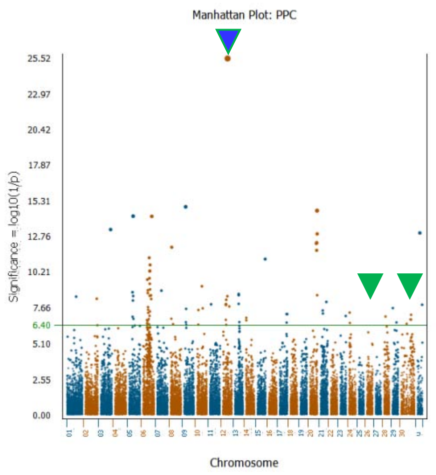

LS_1494

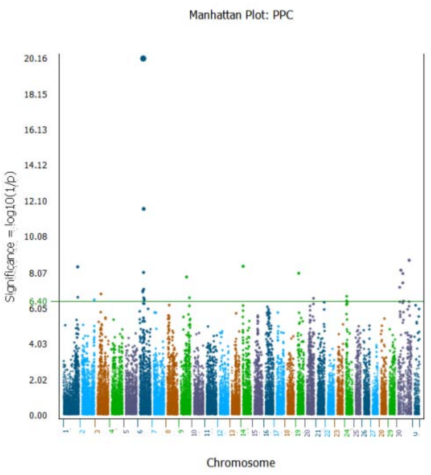

GLS_1494

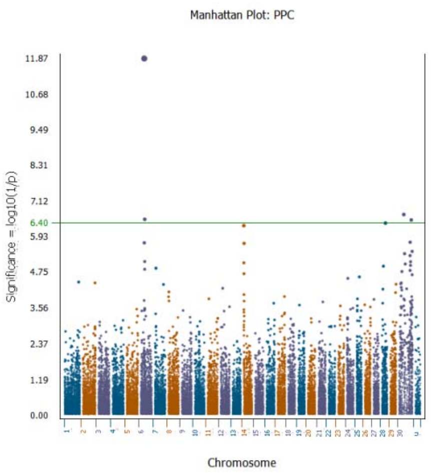

EMMAX_1494

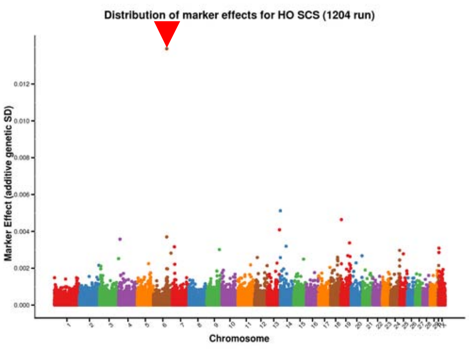

AIPL

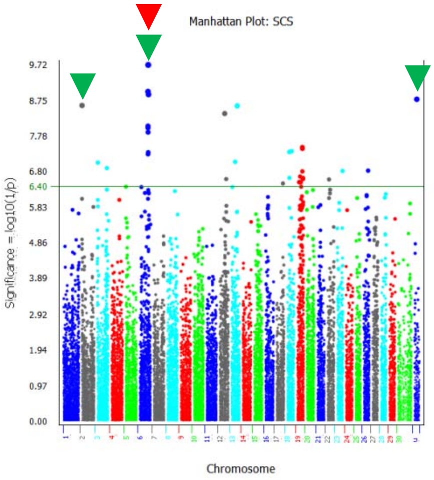

GLS

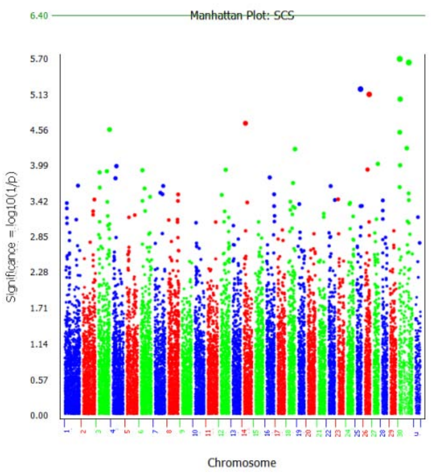

EMMAX-IBS

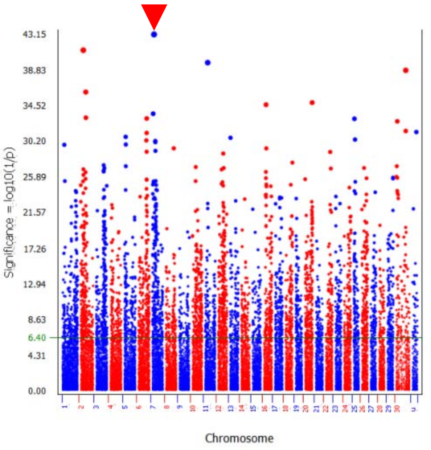

LS

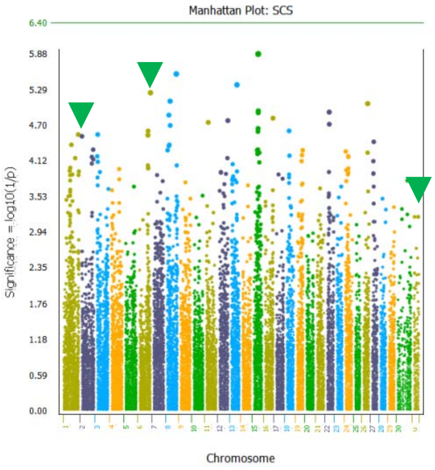

GLS+PCA

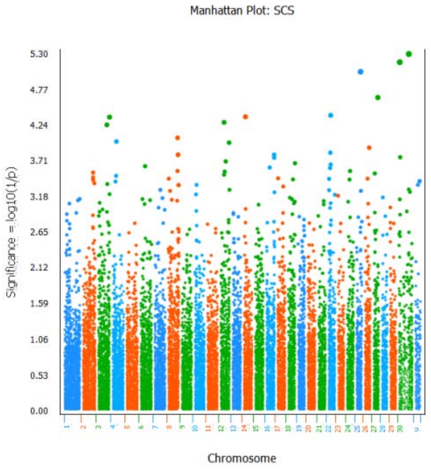

EMMAX+PCA

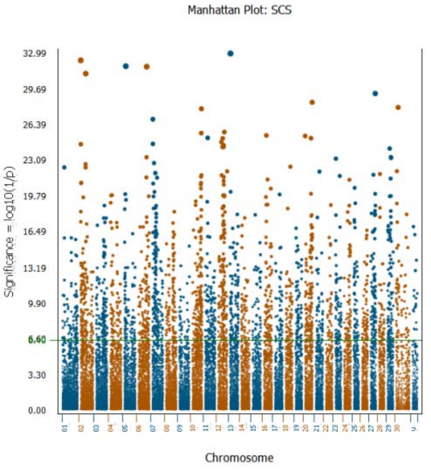

LS_1494

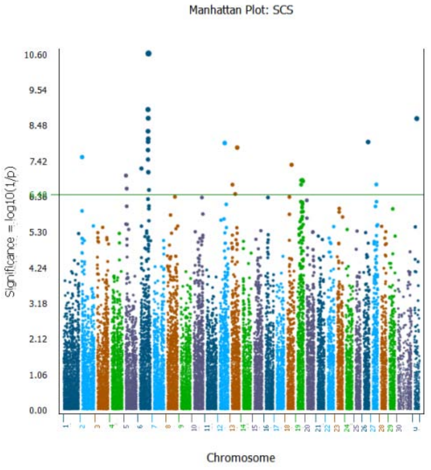

GLS_1494

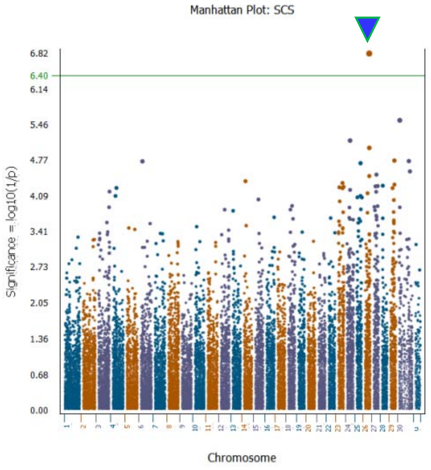

EMMAX_1494

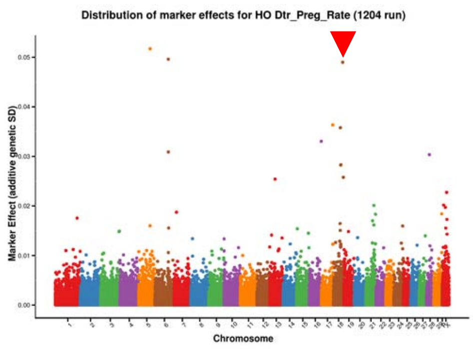

AIPL

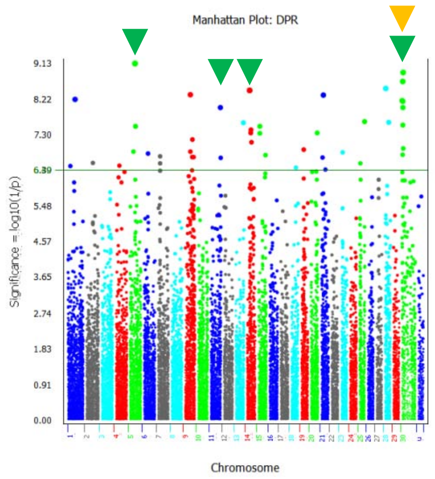

GLS

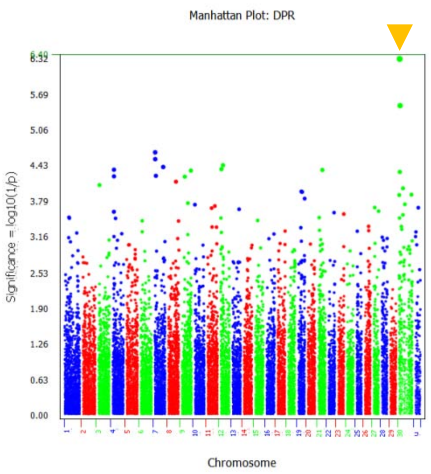

EMMAX-IBS

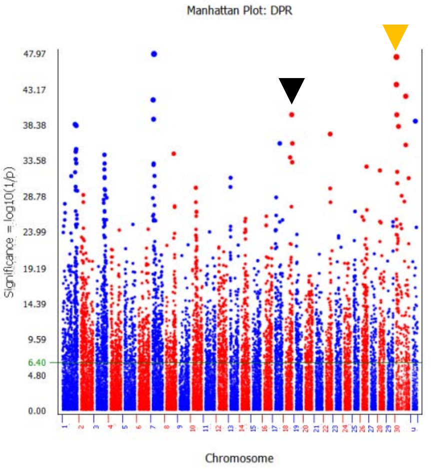

LS

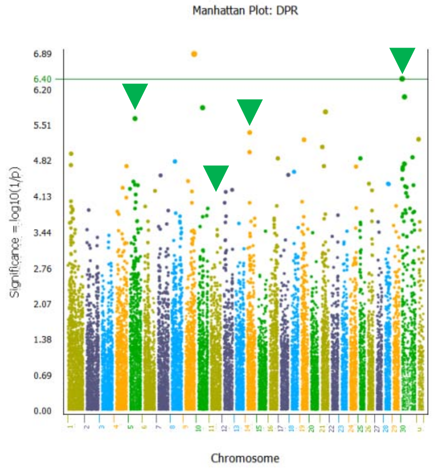

GLS+PCA

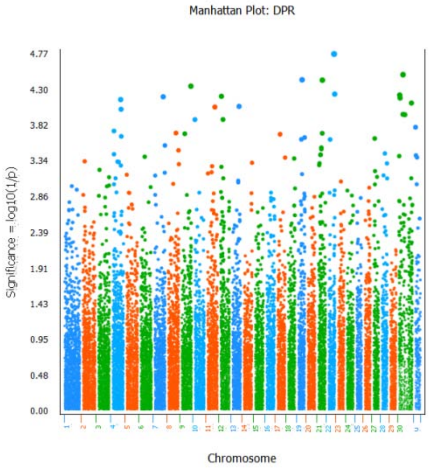

EMMAX+PCA

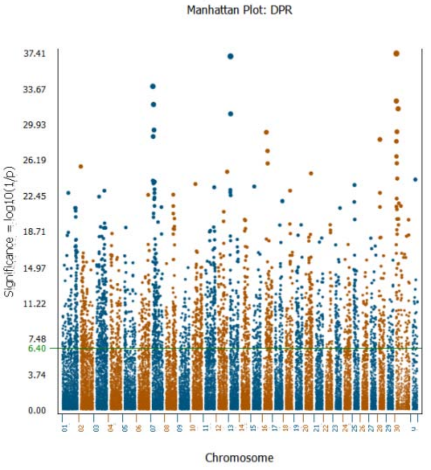

LS_1494

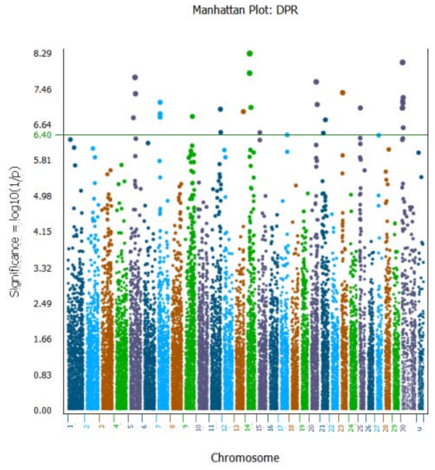

GLS_1494

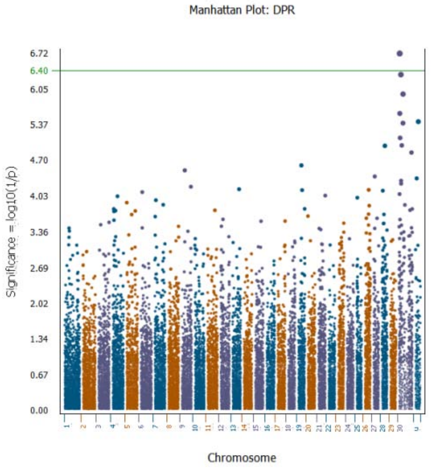

EMMAX_1494

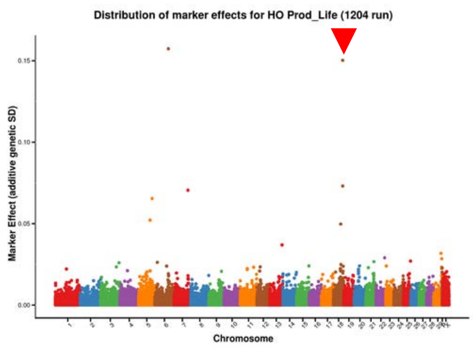

AIPL

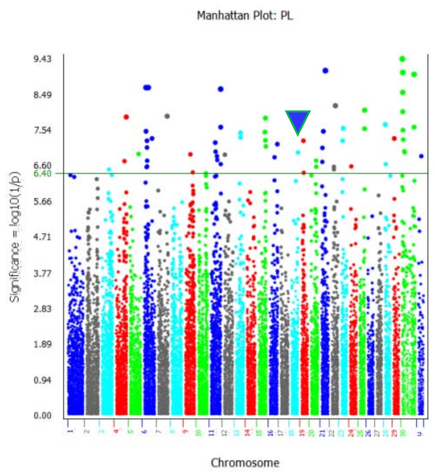

GLS

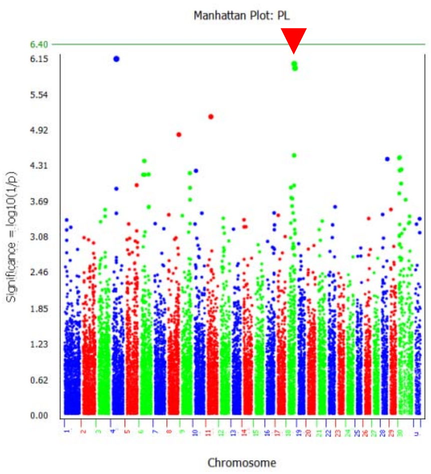

EMMAX-IBS

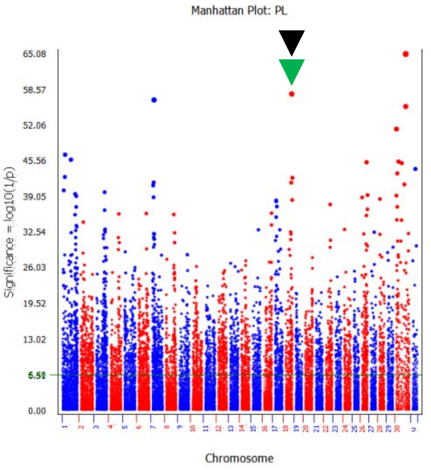

LS

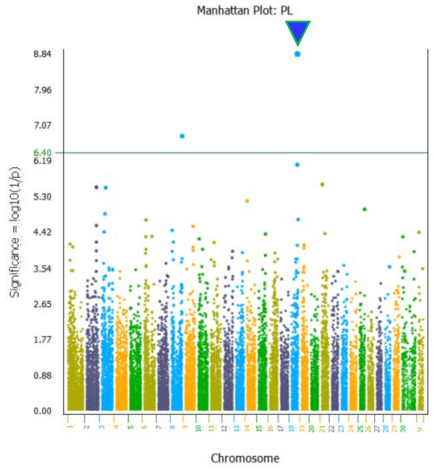

GLS+PCA

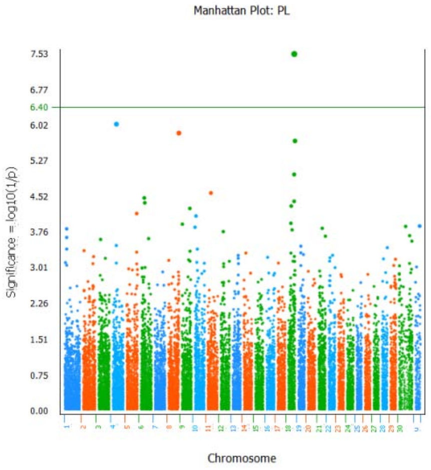

EMMAX+PCA

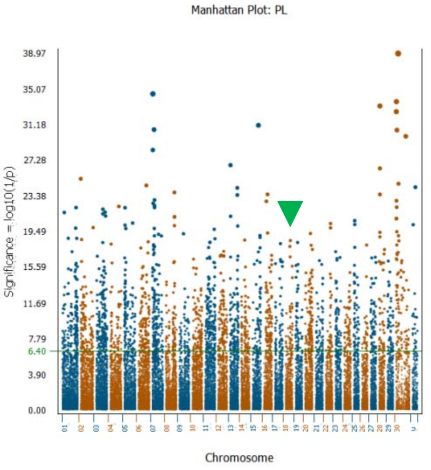

LS_1494

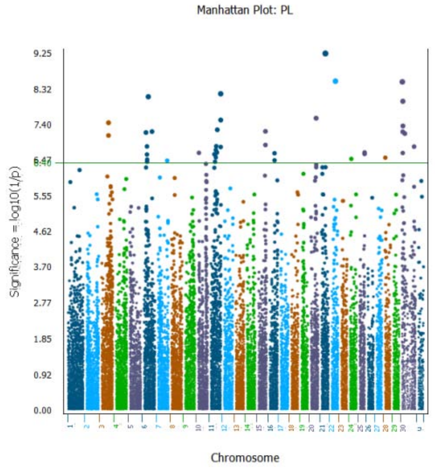

GLS_1494

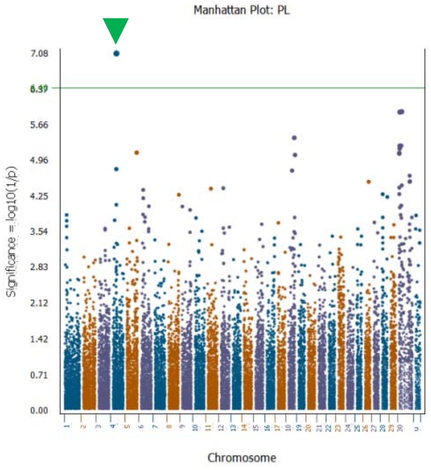

EMMAX_1494

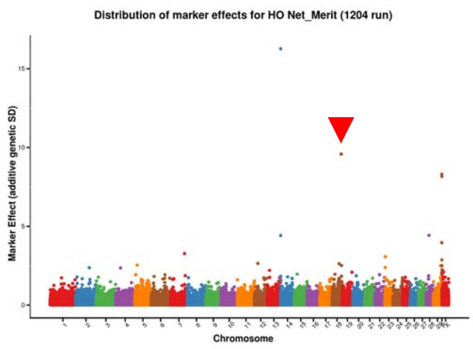

AIPL

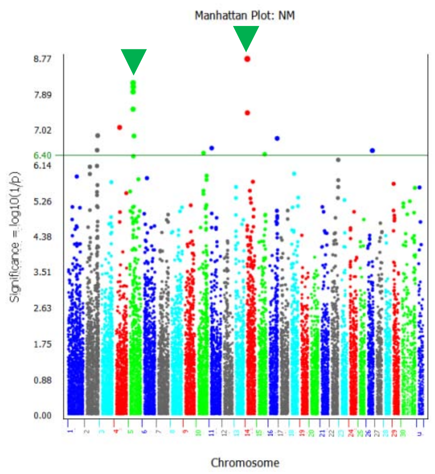

GLS

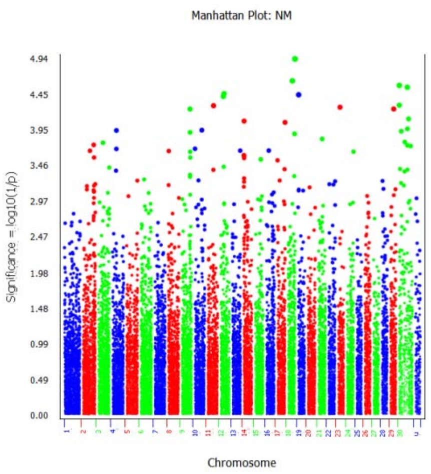

EMMAX-IBS

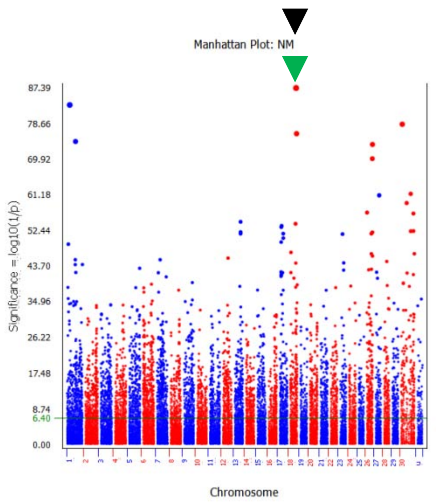

LS

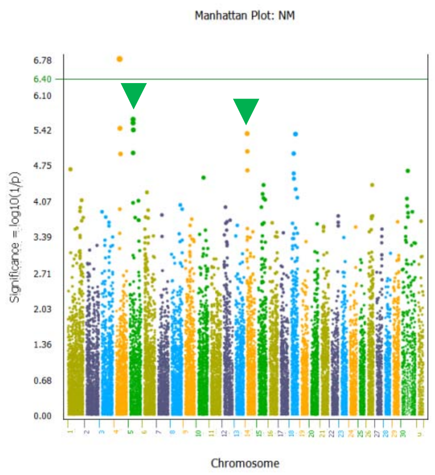

GLS+PCA

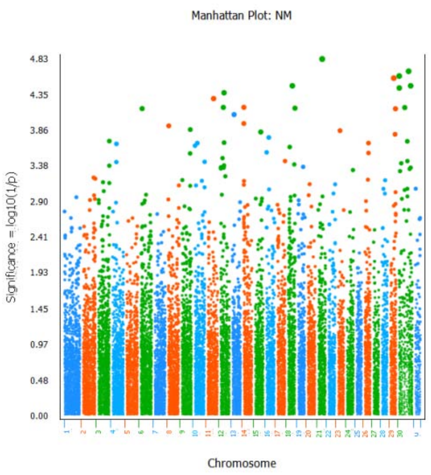

EMMAX+PCA

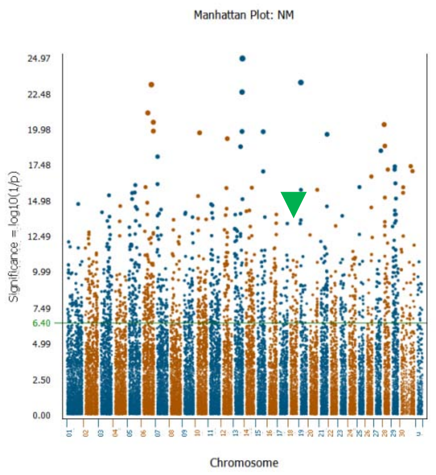

LS_1494

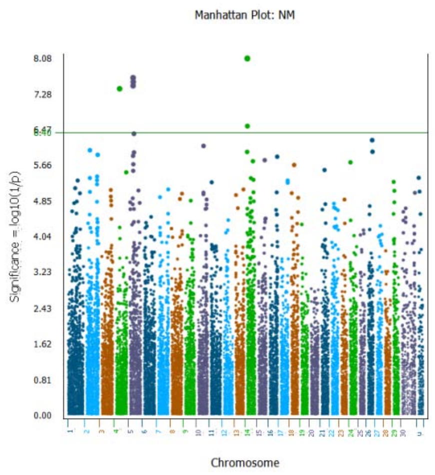

GLS_1494

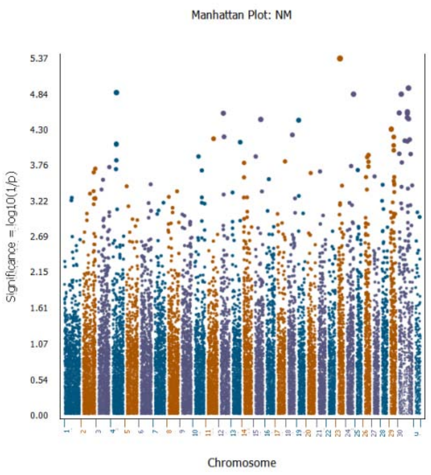

EMMAX_1494

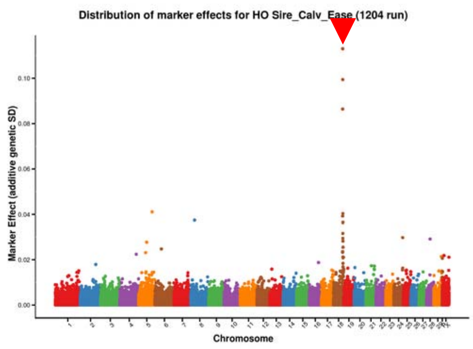

AIPL

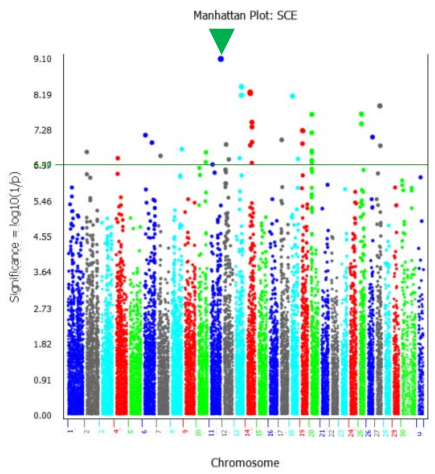

GLS

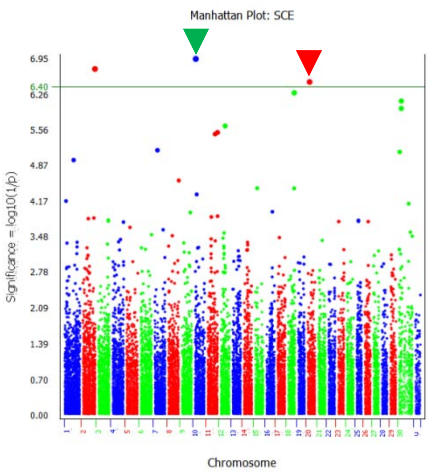

EMMAX-IBS

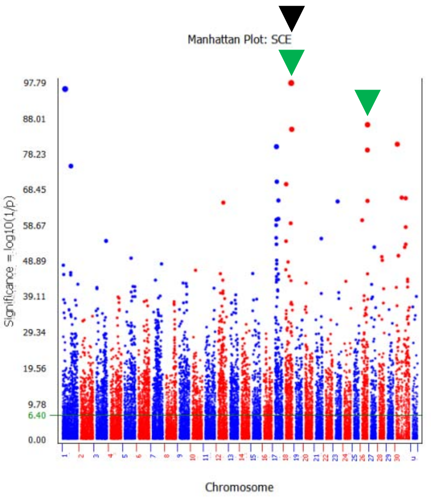

LS

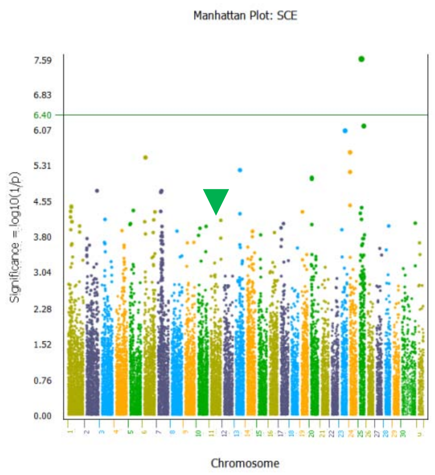

GLS+PCA

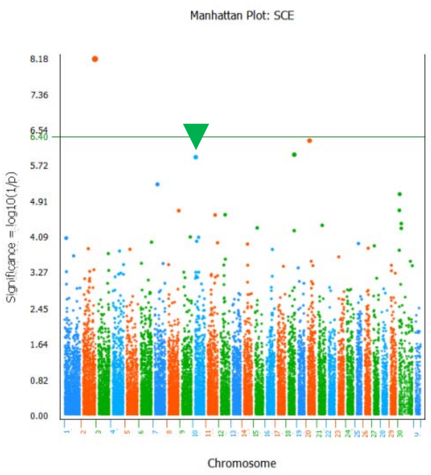

EMMAX+PCA

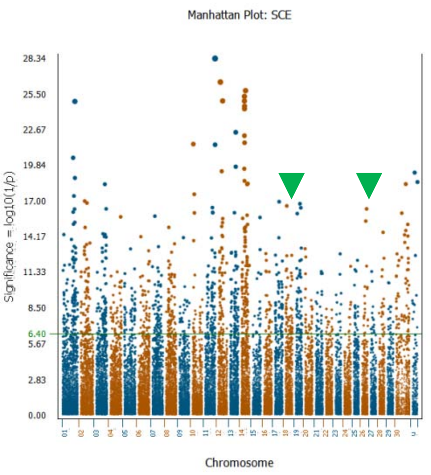

LS_1494

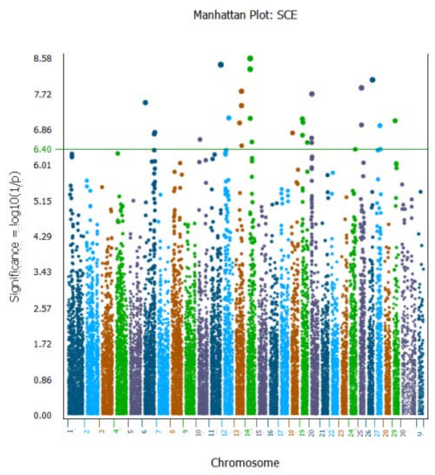

GLS_1494

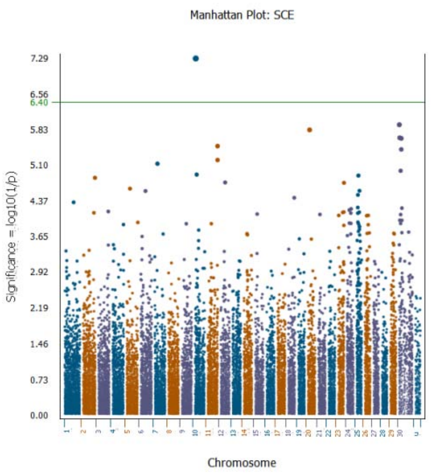

EMMAX_1494

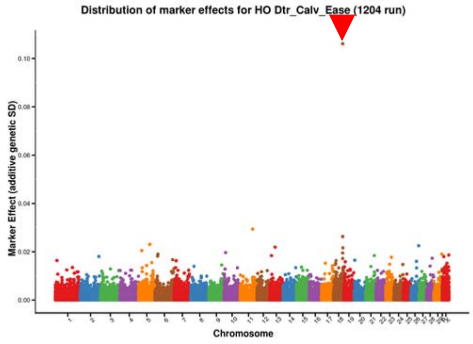

AIPL

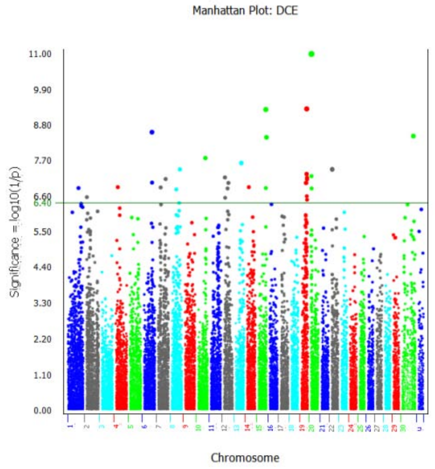

GLS

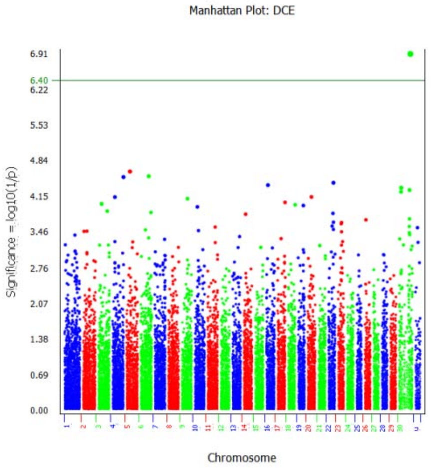

EMMAX-IBS

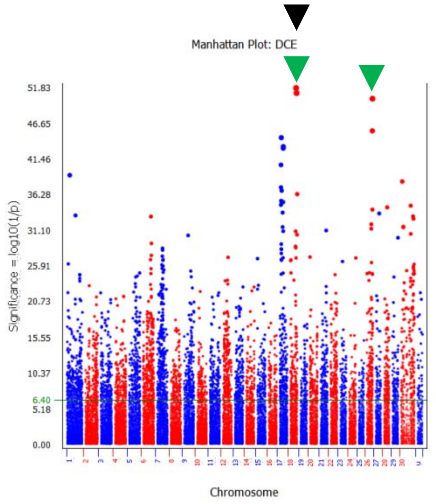

LS

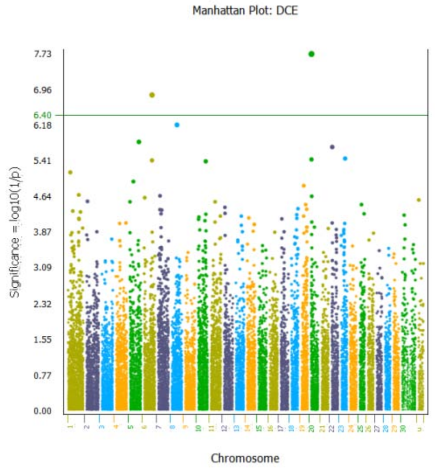

GLS+PCA

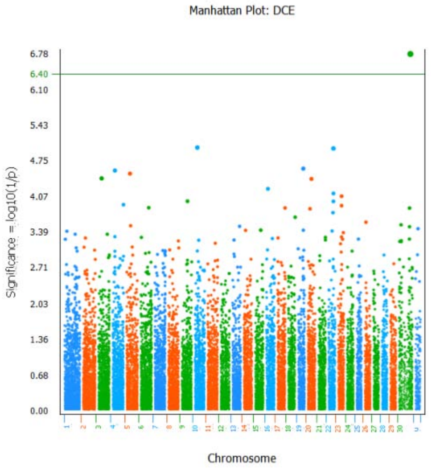

EMMAX+PCA

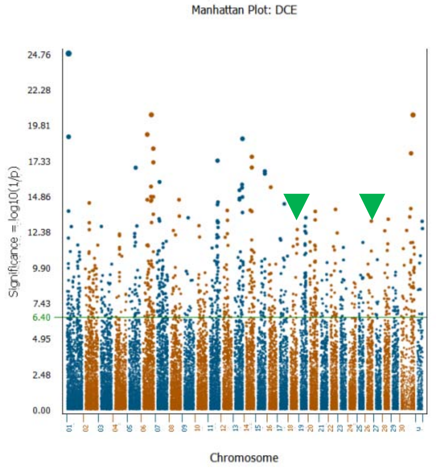

LS_1494

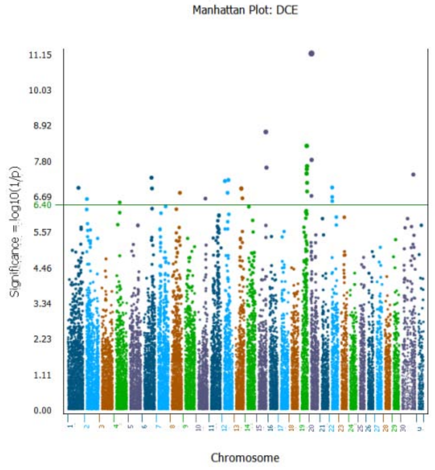

GLS_1494

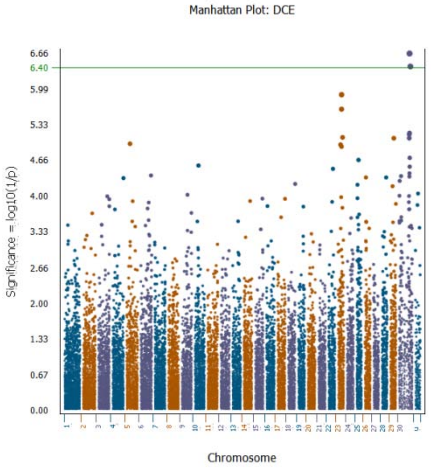

EMMAX_1494

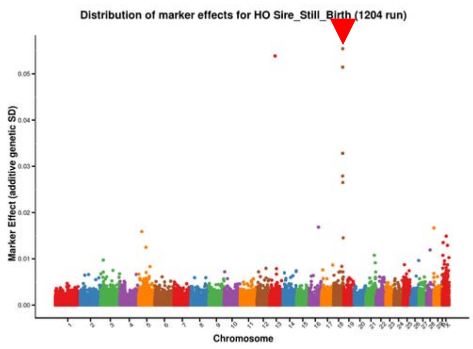

AIPL

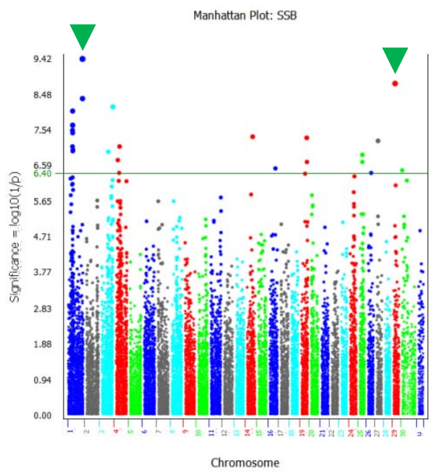

GLS

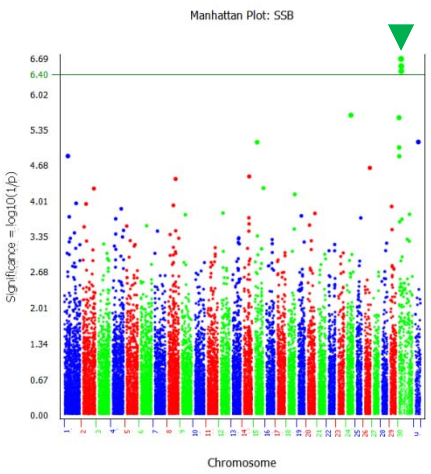

EMMAX-IBS

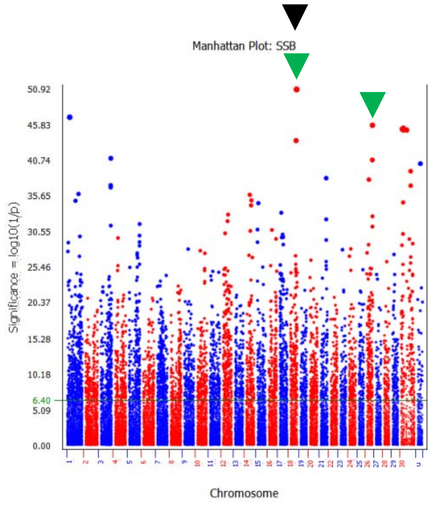

LS

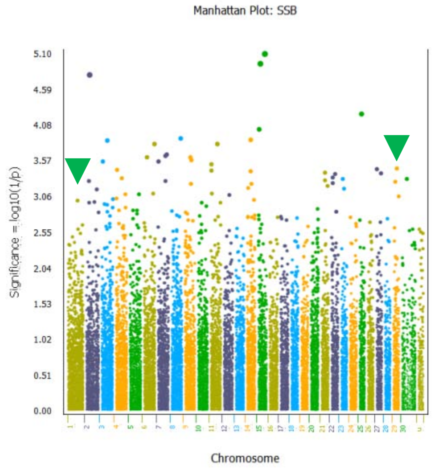

GLS+PCA

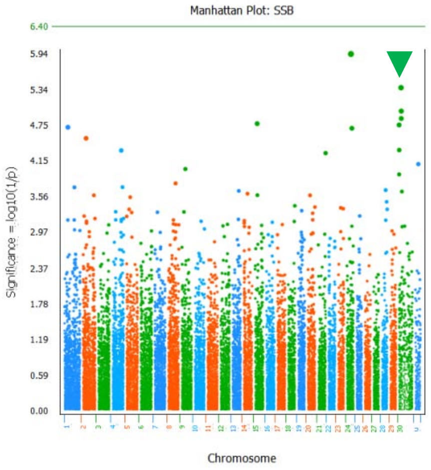

EMMAX+PCA

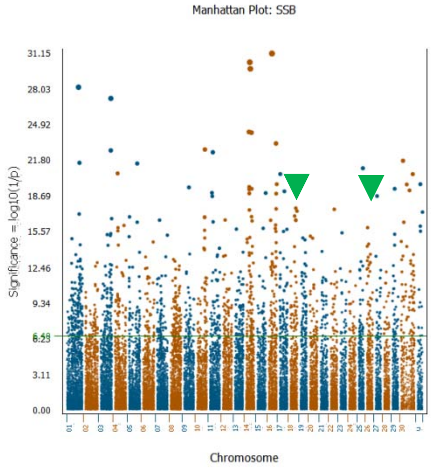

LS_1494

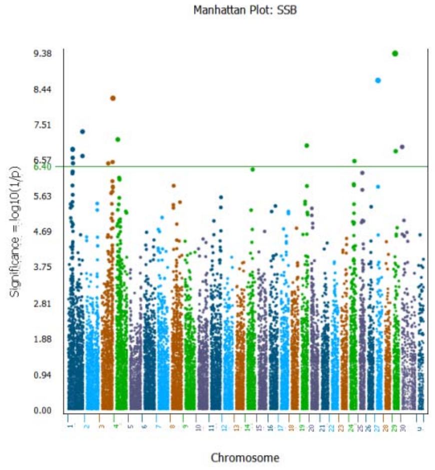

GLS_1494

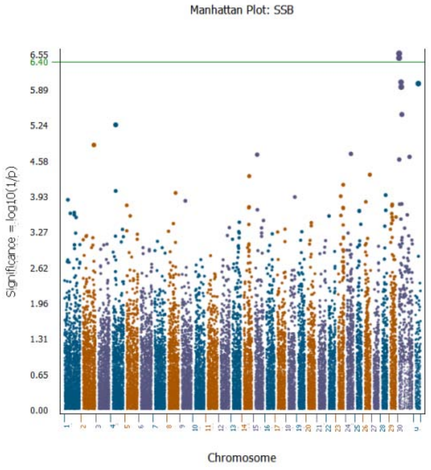

EMMAX_1494

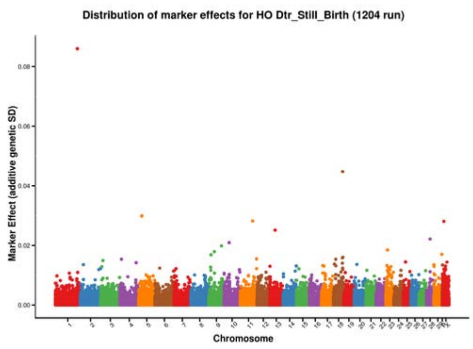

AIPL

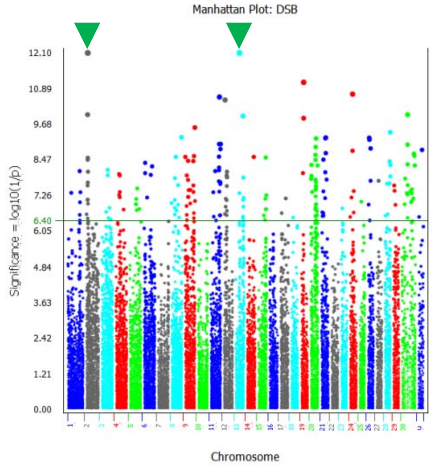

GLS

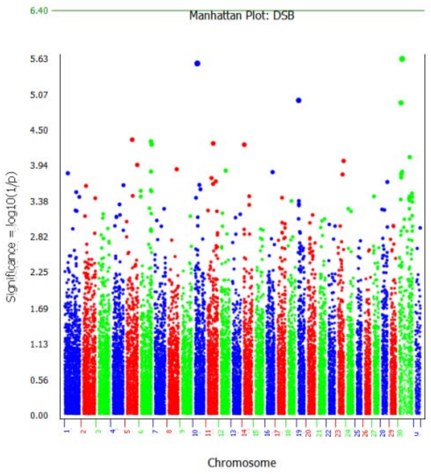

EMMAX-IBS

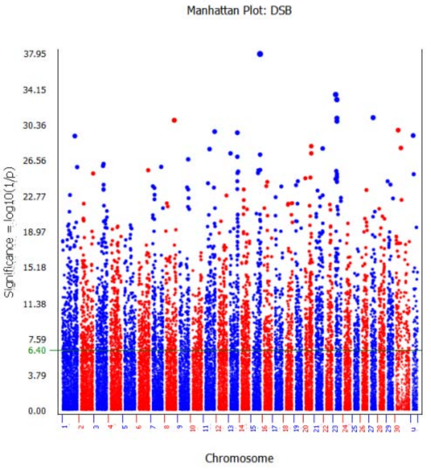

LS

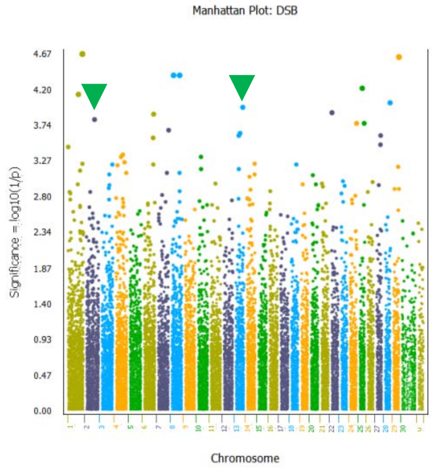

GLS+PCA

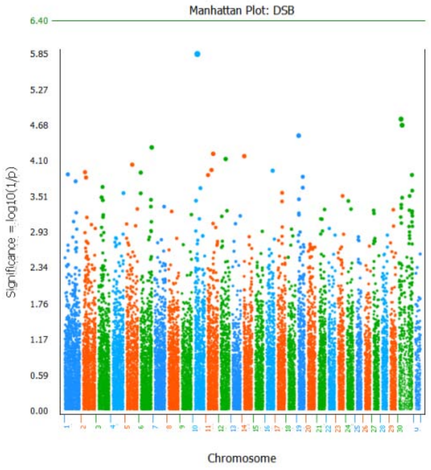

EMMAX+PCA

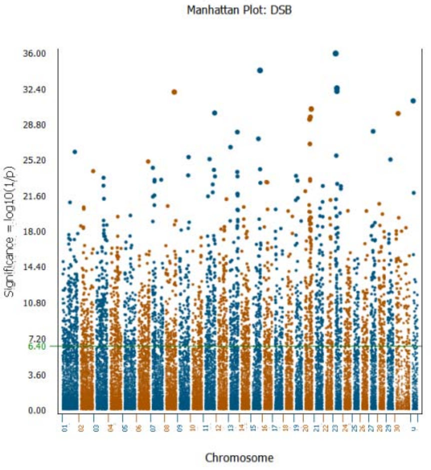

LS_1494

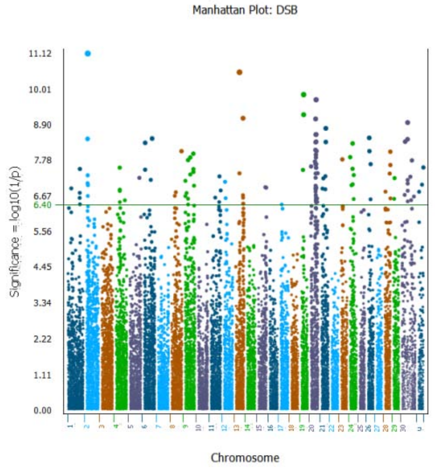

GLS_1494

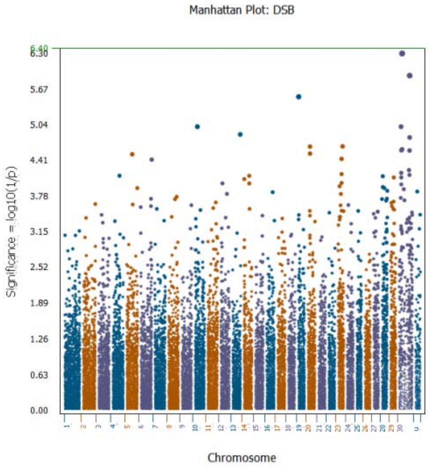

EMMAX_1494

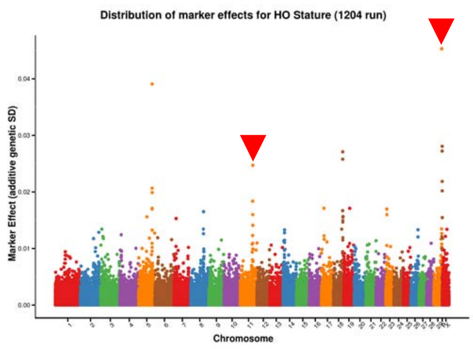

AIPL

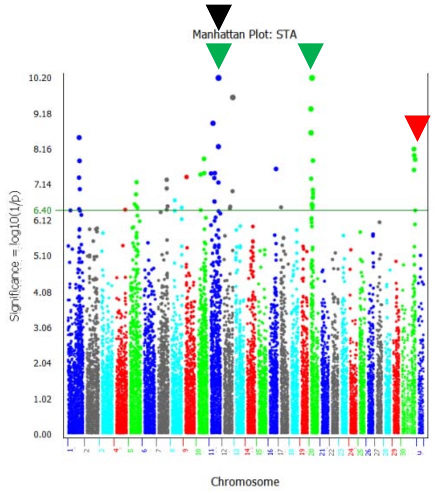

GLS

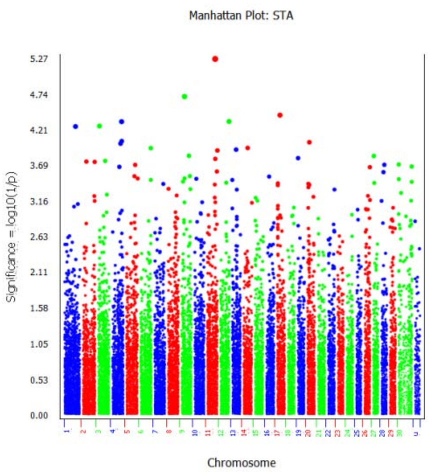

EMMAX-IBS

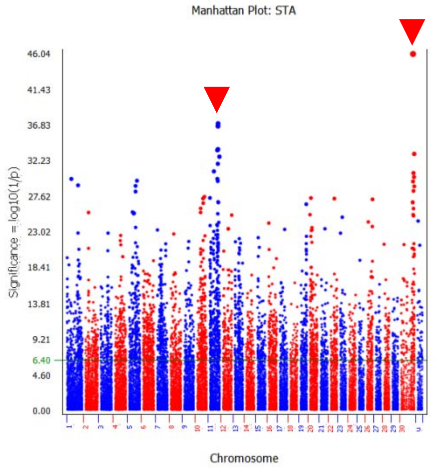

LS

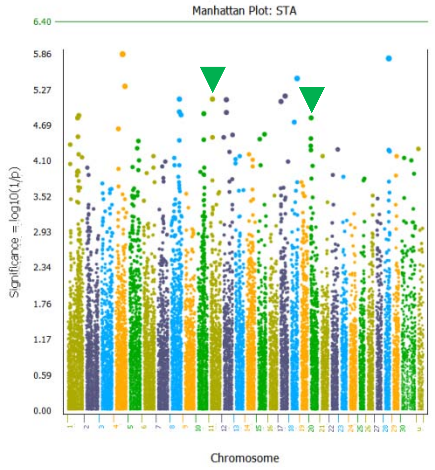

GLS+PCA

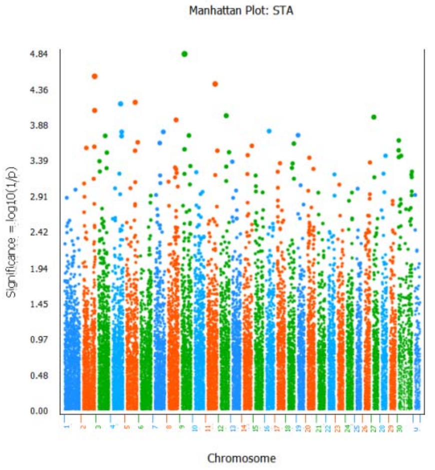

EMMAX+PCA

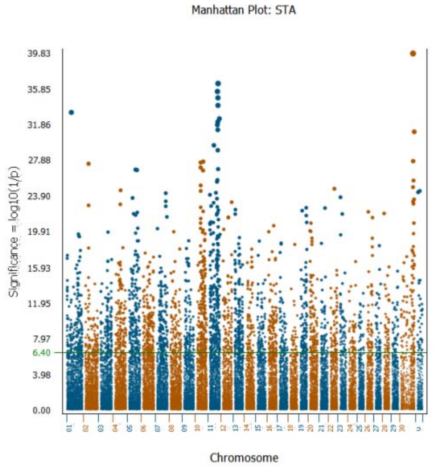

LS_1494

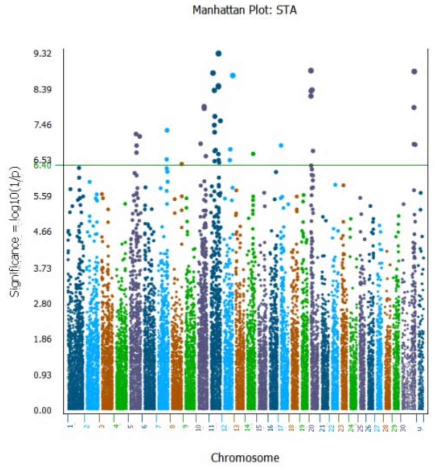

GLS_1494

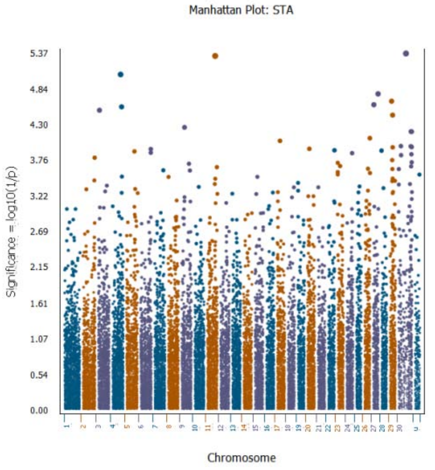

EMMAX_1494

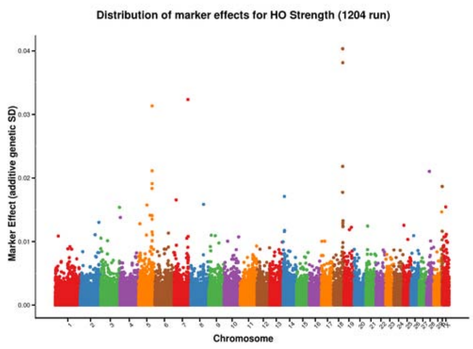

AIPL

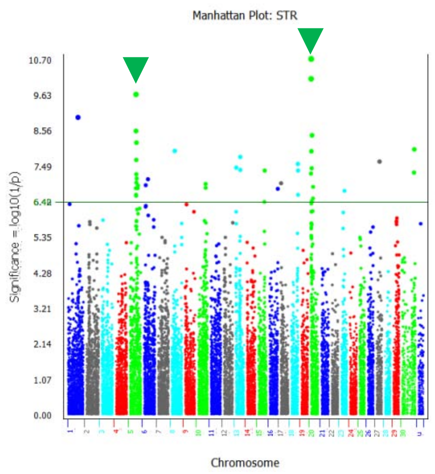

GLS

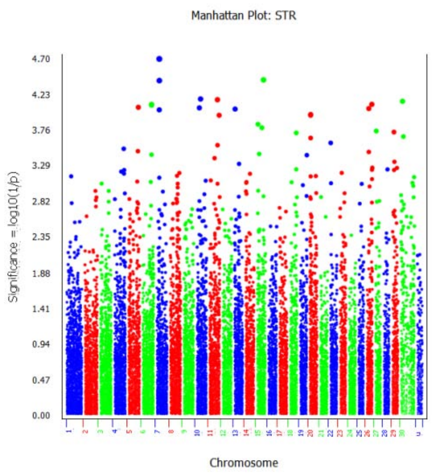

EMMAX-IBS

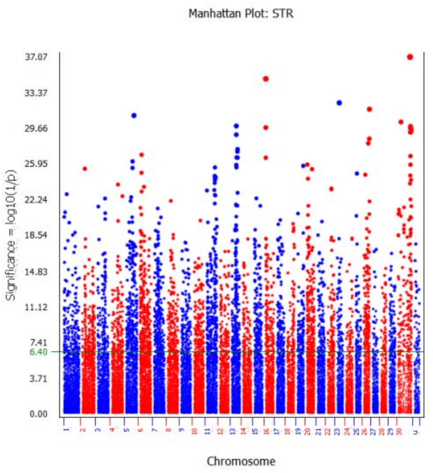

LS

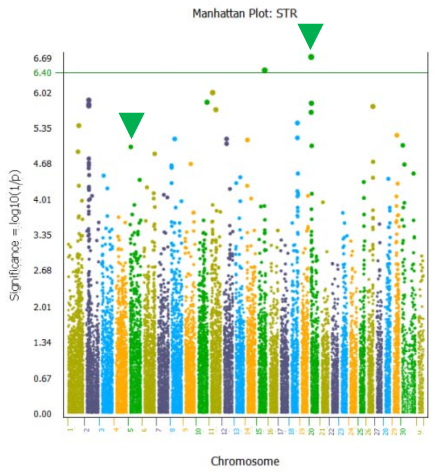

GLS+PCA

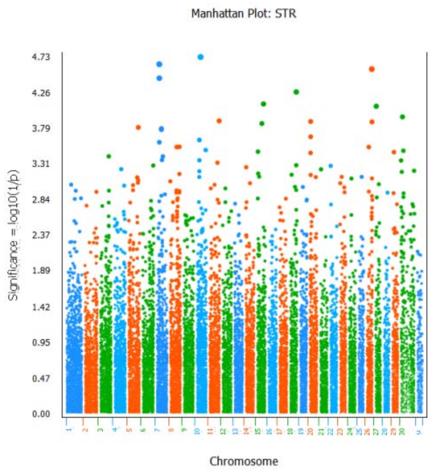

EMMAX+PCA

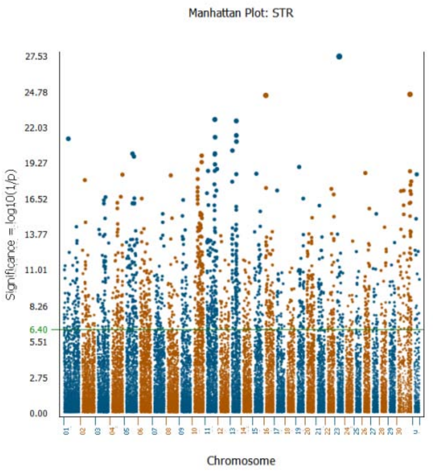

LS_1494

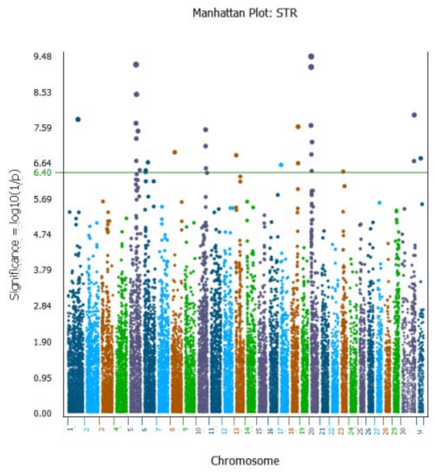

GLS_1494

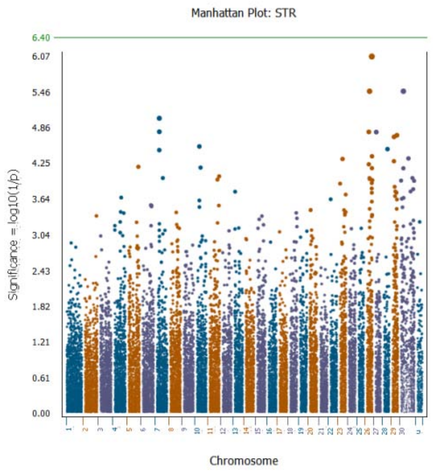

EMMAX_1494

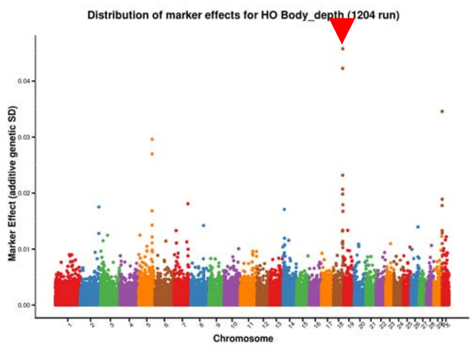

AIPL

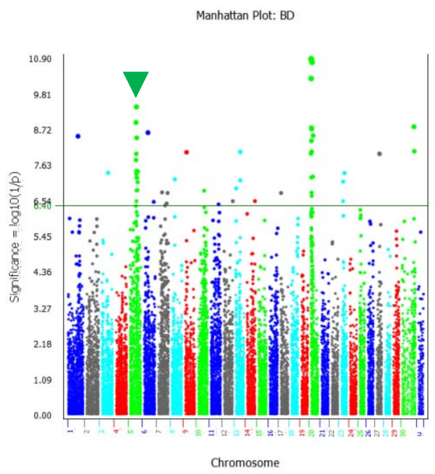

GLS

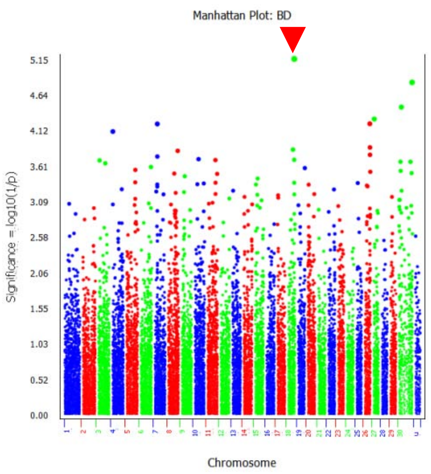

EMMAX-IBS

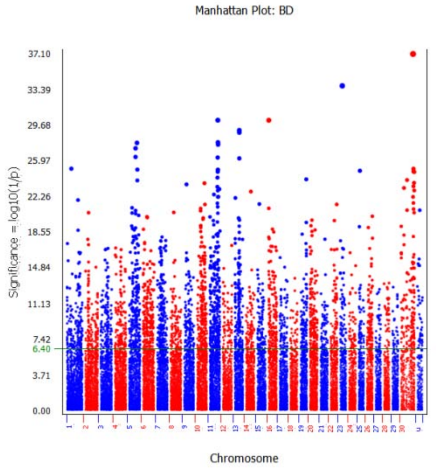

LS

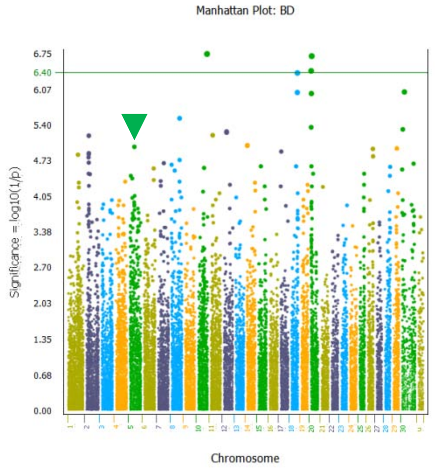

GLS+PCA

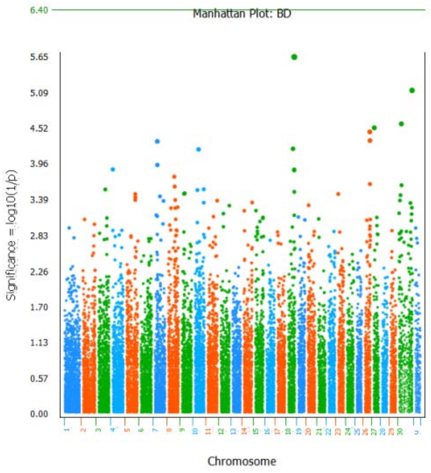

EMMAX+PCA

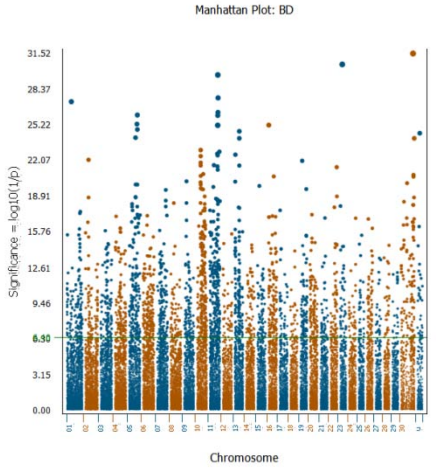

LS_1494

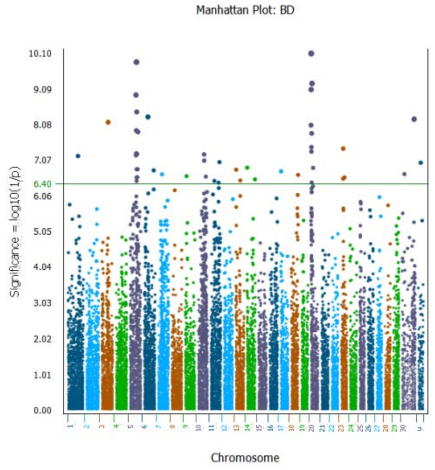

GLS_1494

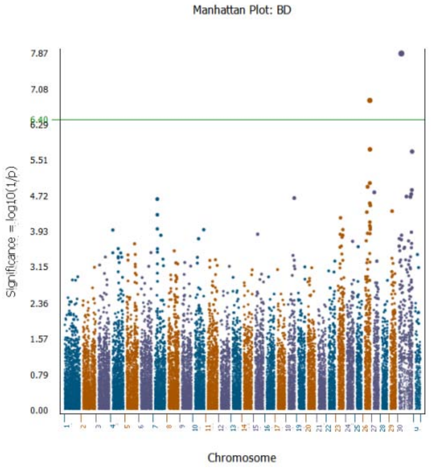

EMMAX_1494

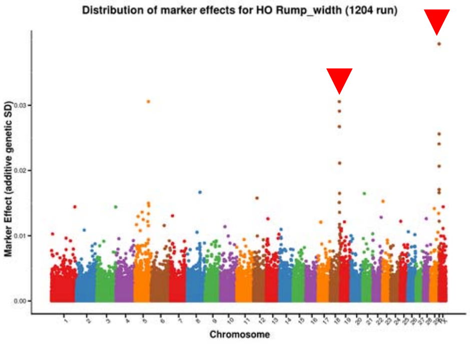

AIPL

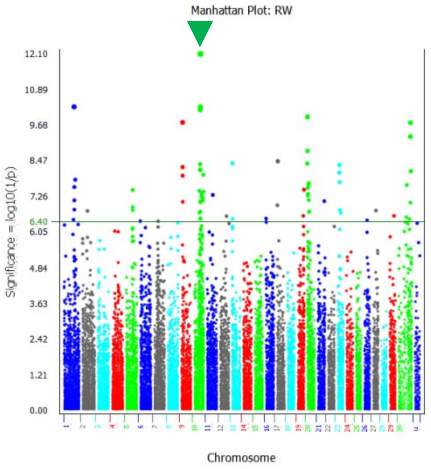

GLS

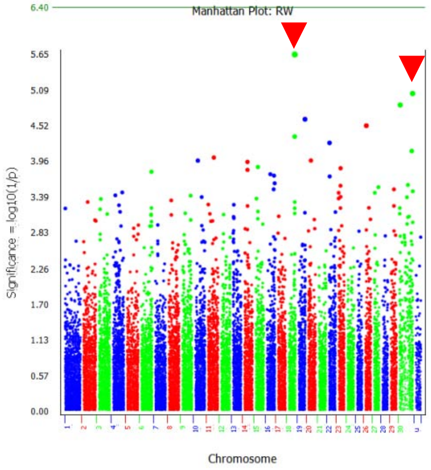

EMMAX-IBS

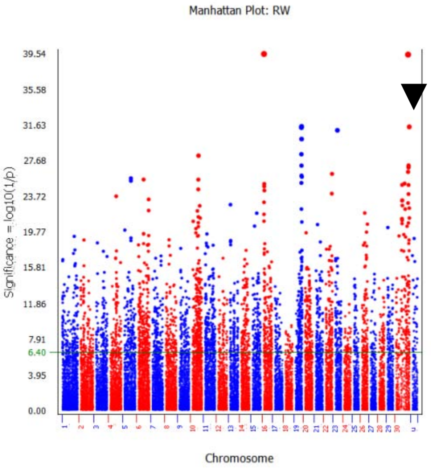

LS

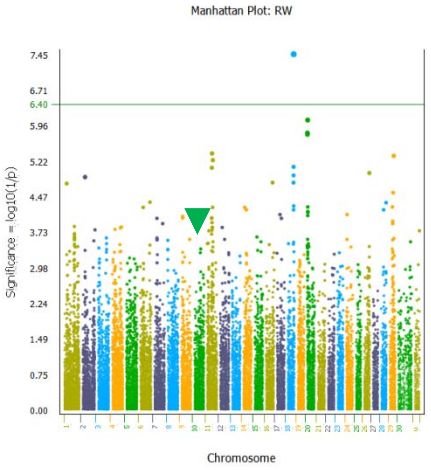

GLS+PCA

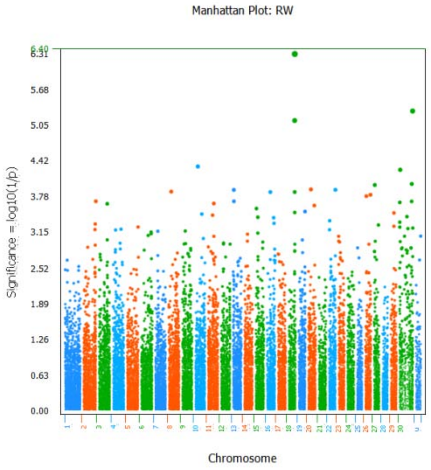

EMMAX+PCA

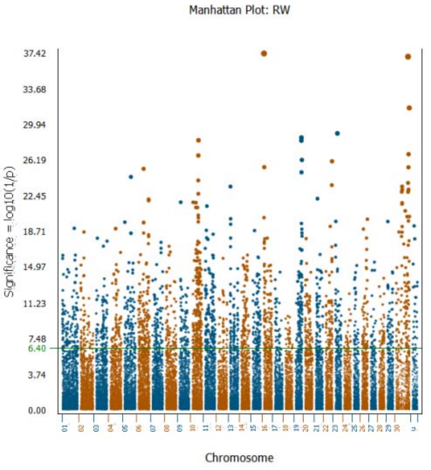

LS_1494

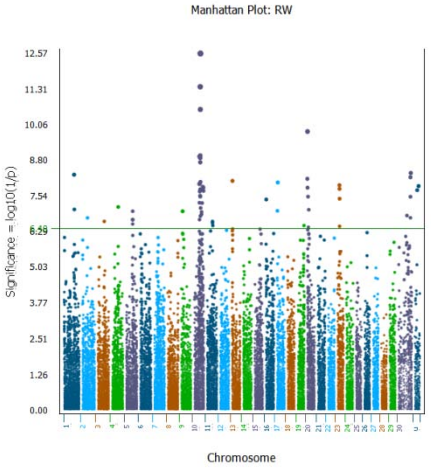

GLS_1494

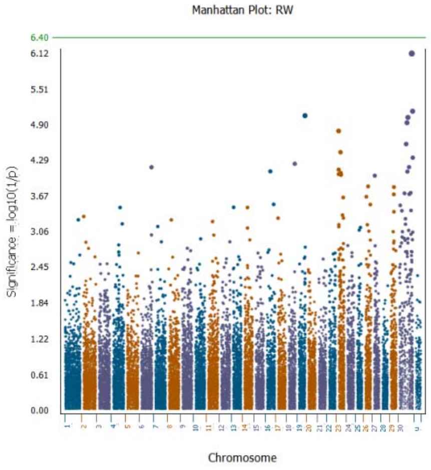

EMMAX_1494

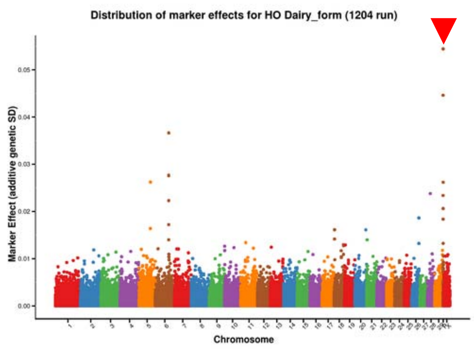

AIPL

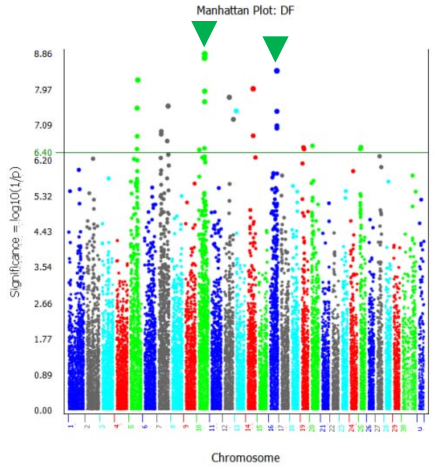

GLS

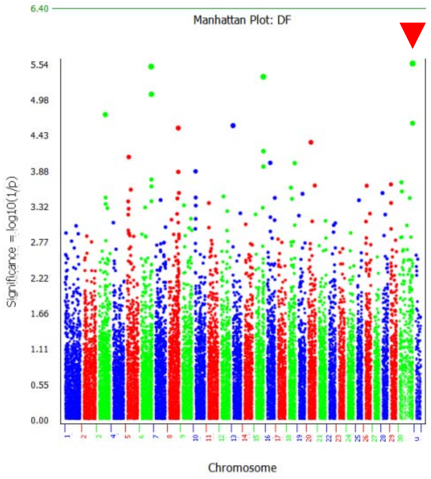

EMMAX-IBS

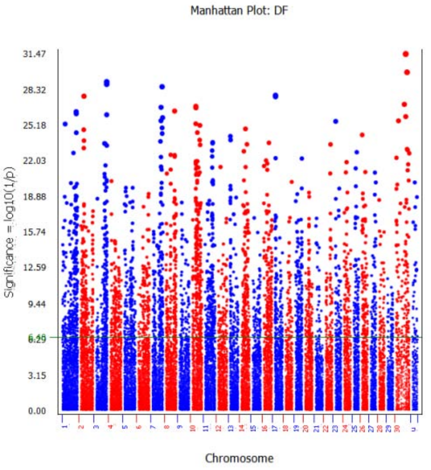

LS

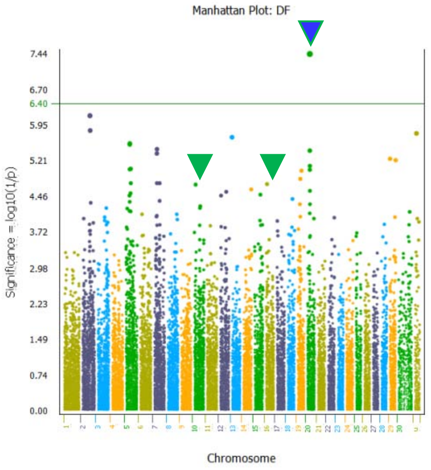

GLS+PCA

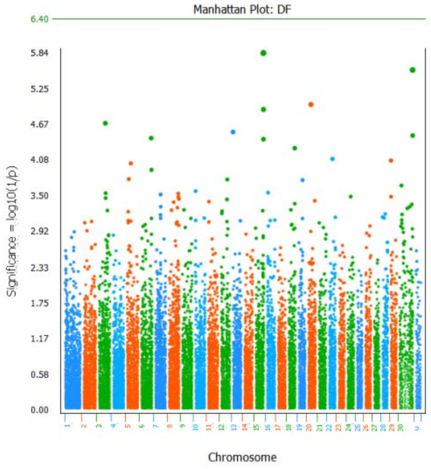

EMMAX+PCA

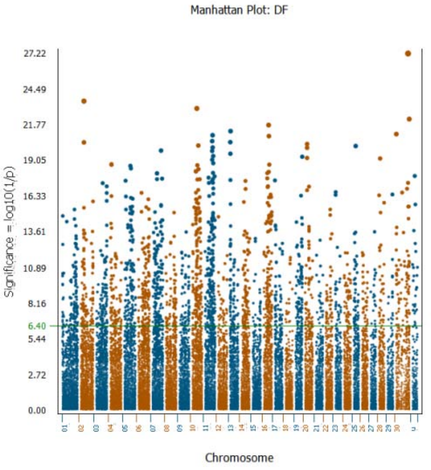

LS_1494

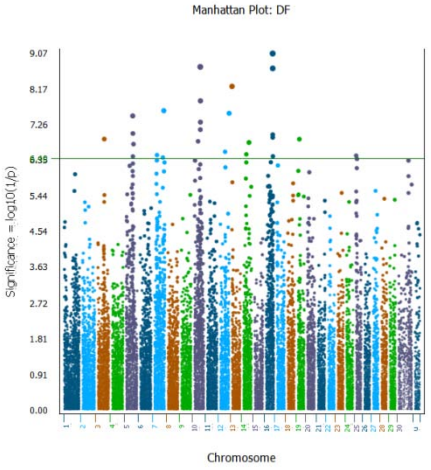

GLS_1494

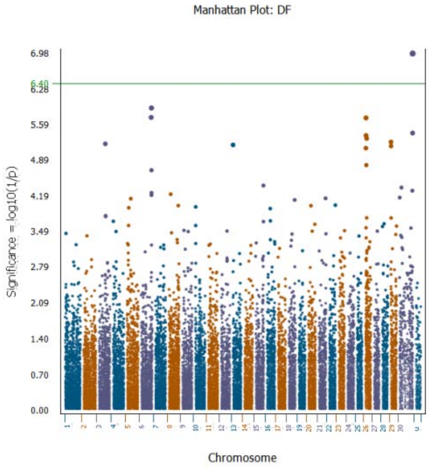

EMMAX_1494

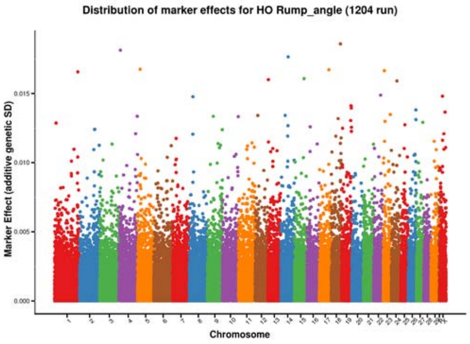

AIPL

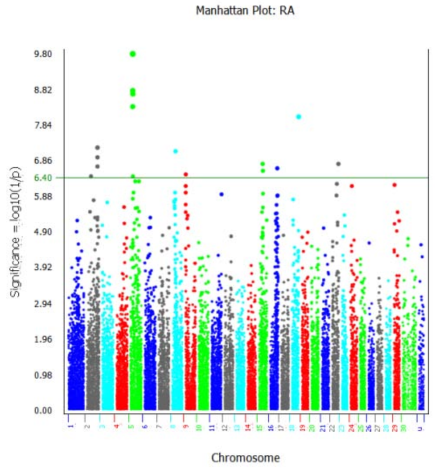

GLS

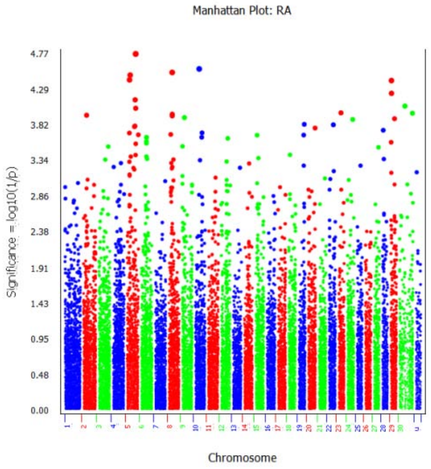

EMMAX-IBS

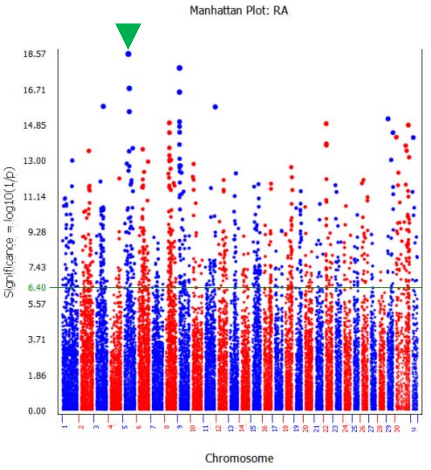

LS

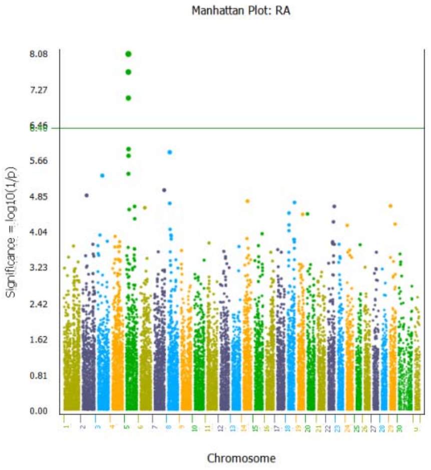

GLS+PCA

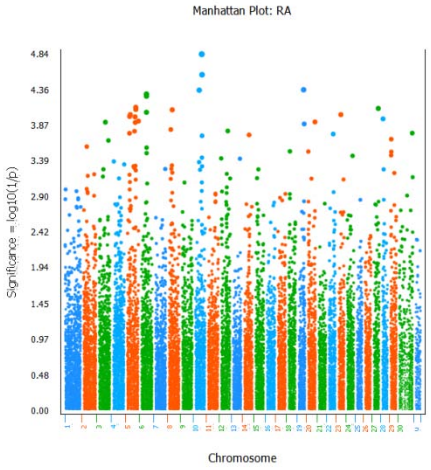

EMMAX+PCA

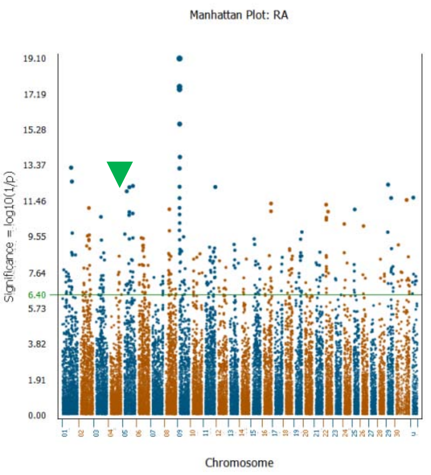

LS_1494

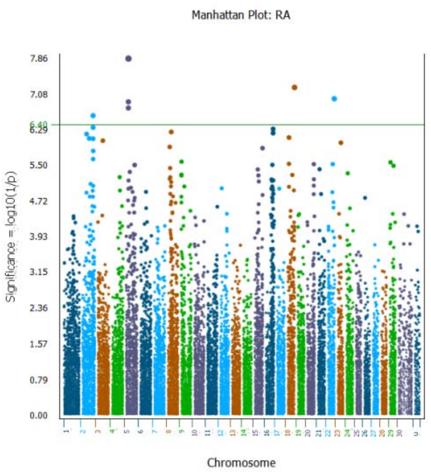

GLS_1494

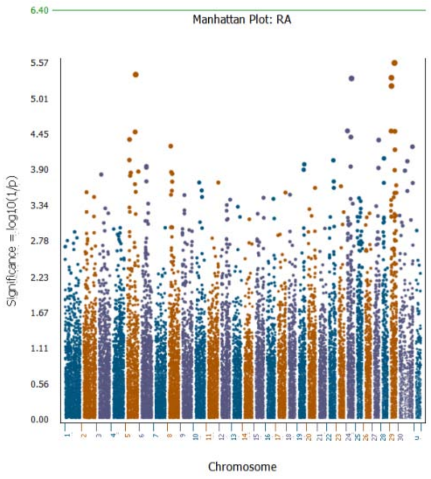

EMMAX_1494

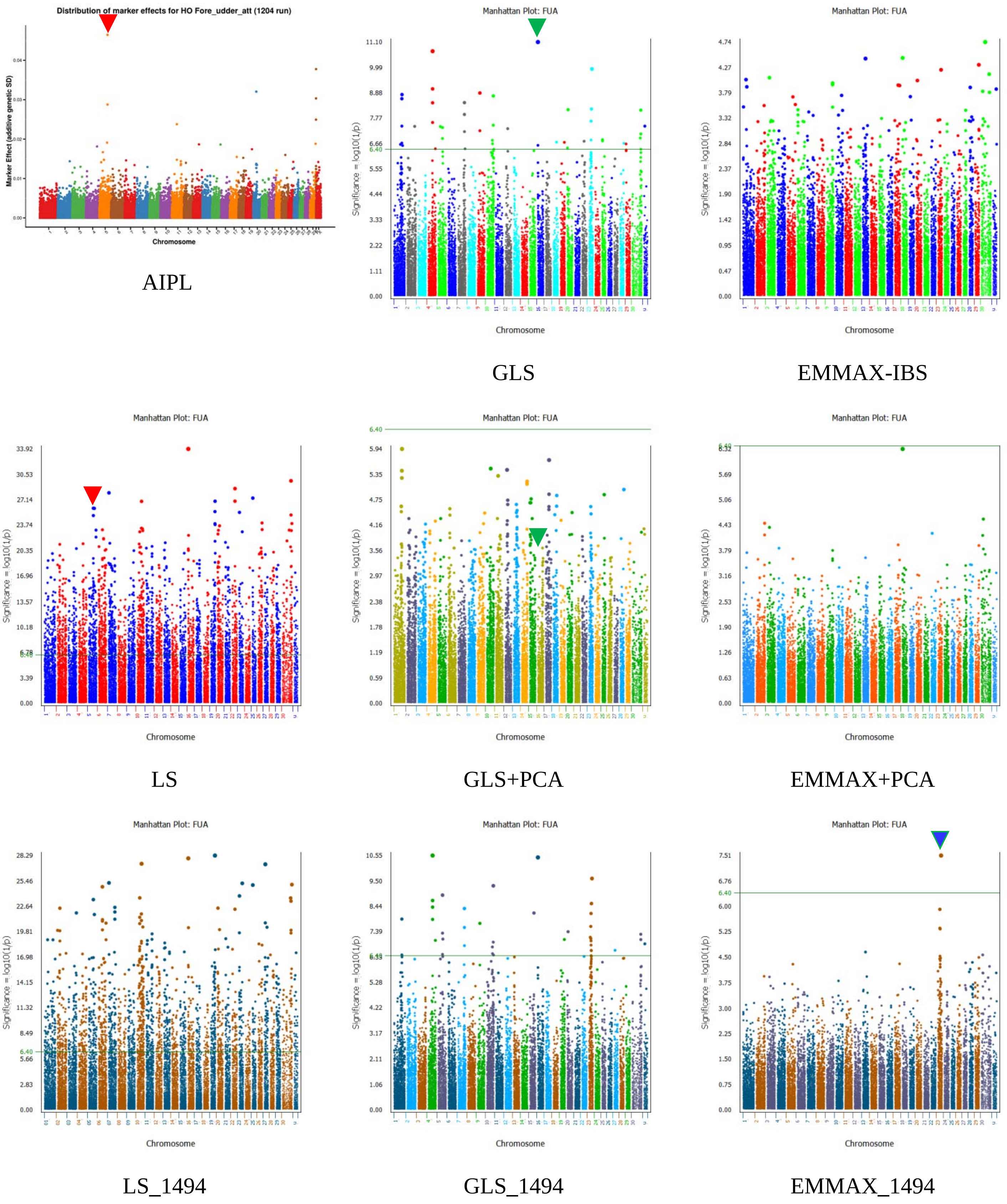

Distribution of marker effects for HO Fore_udder_att (1204 run)
Marker Effect (additive genetic SD)
Chromosome
AIPL
Manhattan Plot: FUA
Significance = -log10(1/p)
Chromosome
GLS
Manhattan Plot: FUA
Significance = -log10(1/p)
Chromosome
EMMAX-IBS
Manhattan Plot: FUA
Significance = -log10(1/p)
Chromosome
LS
Manhattan Plot: FUA
Significance = -log10(1/p)
Chromosome
GLS+PCA
Manhattan Plot: FUA
Significance = -log10(1/p)
Chromosome
EMMAX+PCA
Manhattan Plot: FUA
Significance = -log10(1/p)
Chromosome
LS_1494
Manhattan Plot: FUA
Significance = -log10(1/p)
Chromosome
GLS_1494
Manhattan Plot: FUA
Significance = -log10(1/p)
Chromosome
EMMAX_1494

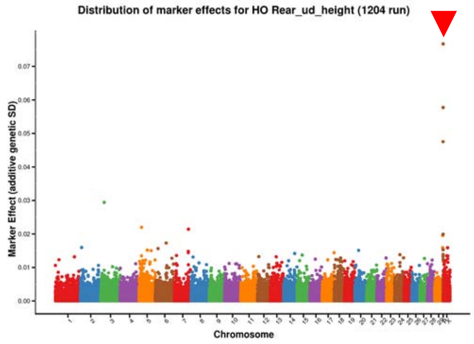

AIPL

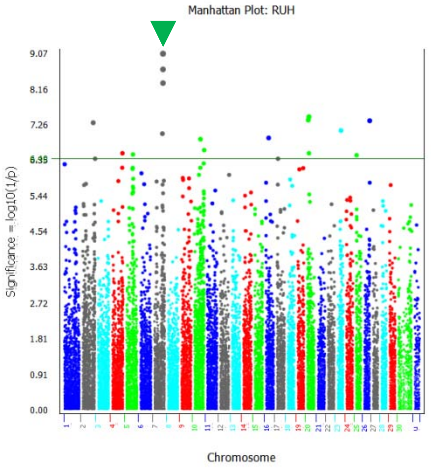

GLS

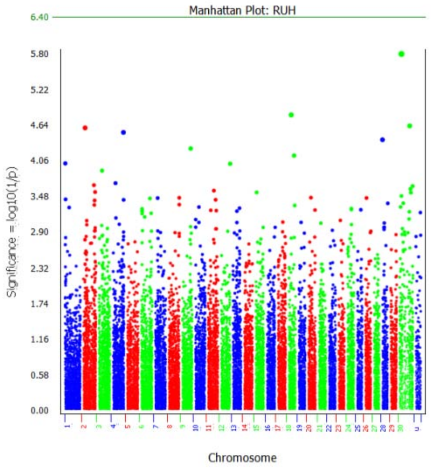

EMMAX-IBS

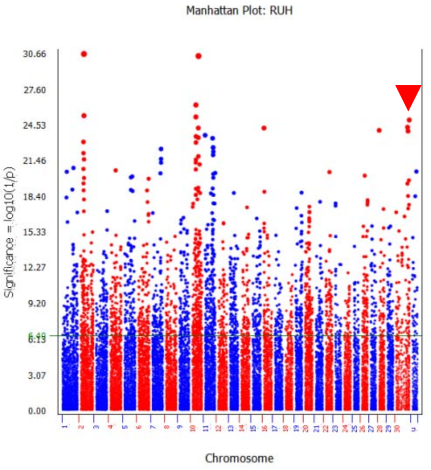

LS

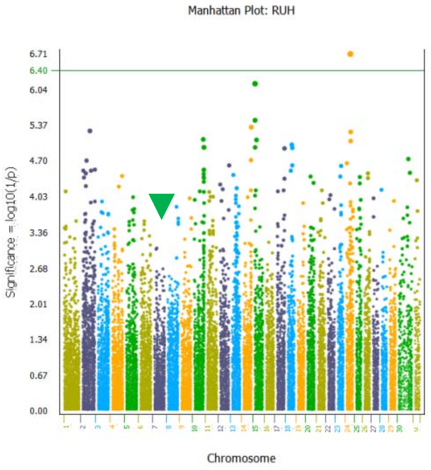

GLS+PCA

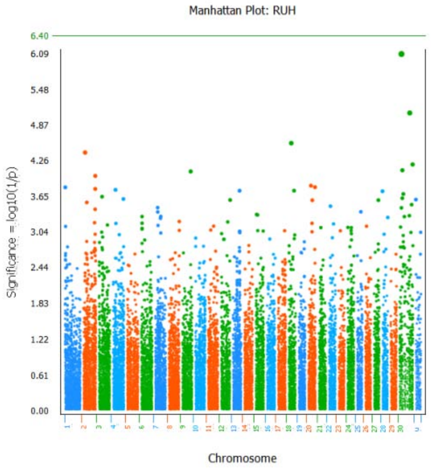

EMMAX+PCA

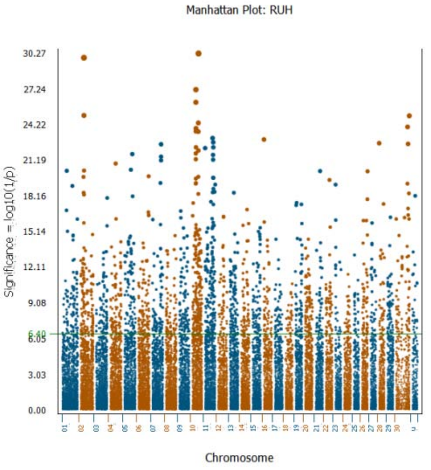

LS_1494

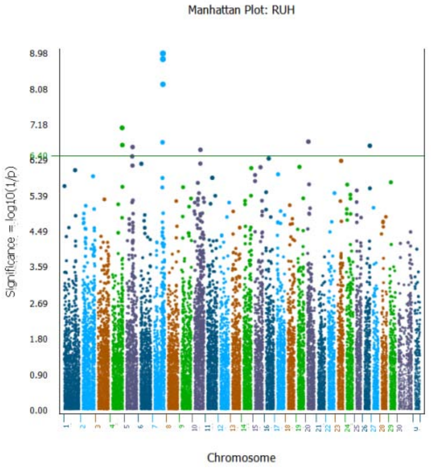

GLS_1494

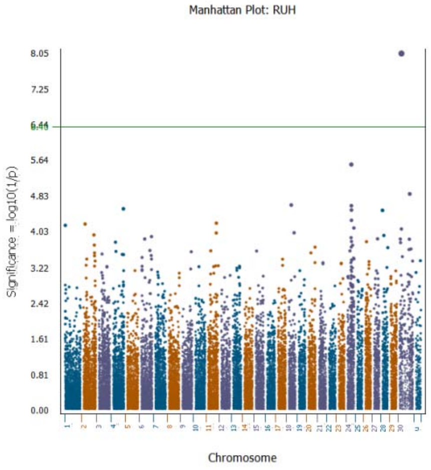

EMMAX_1494

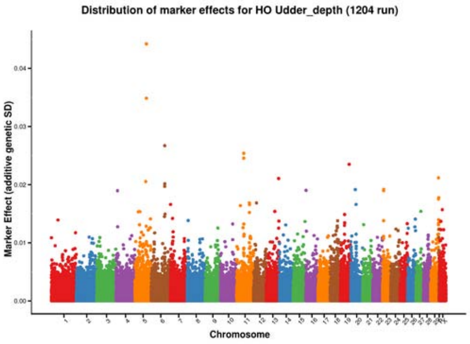

AIPL

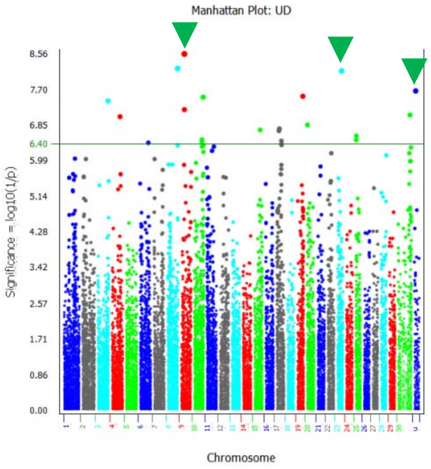

GLS

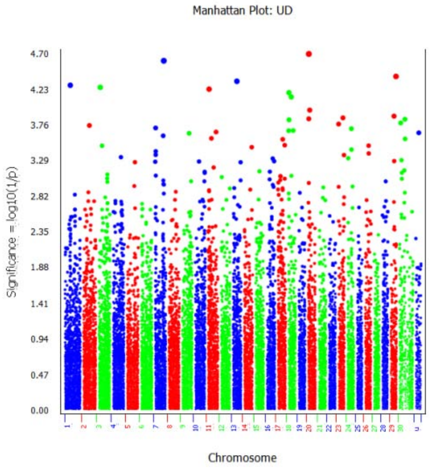

EMMAX-IBS

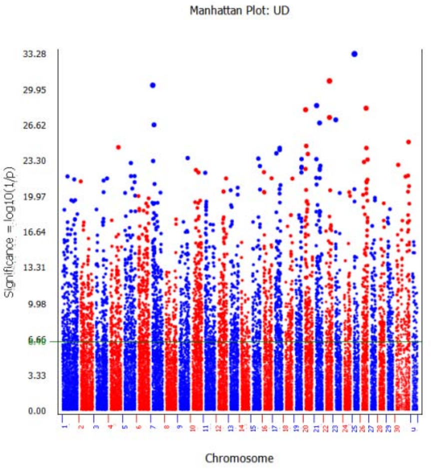

LS

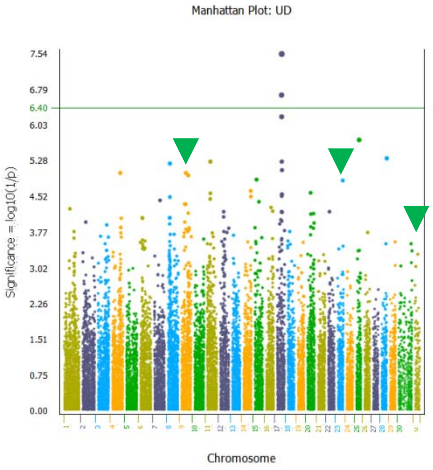

GLS+PCA

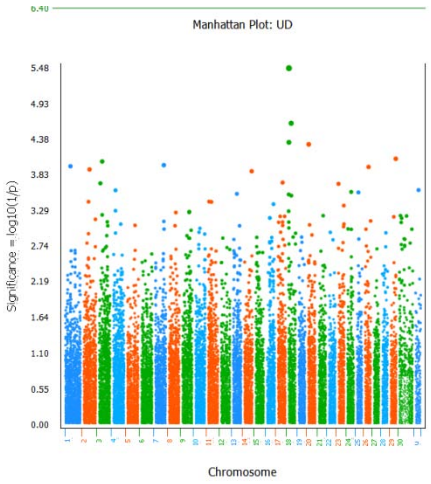

EMMAX+PCA

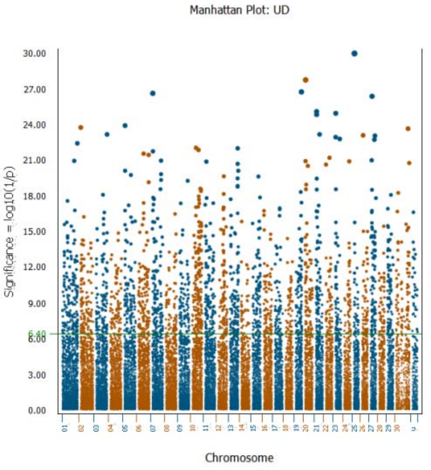

LS_1494

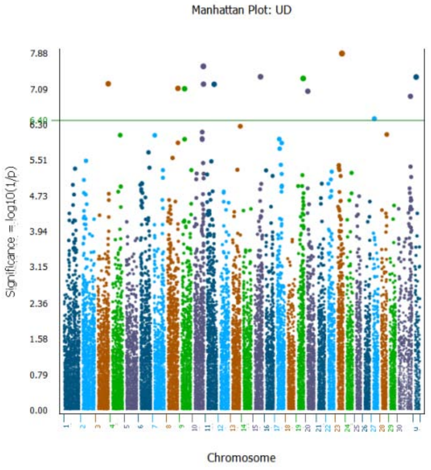

GLS_1494

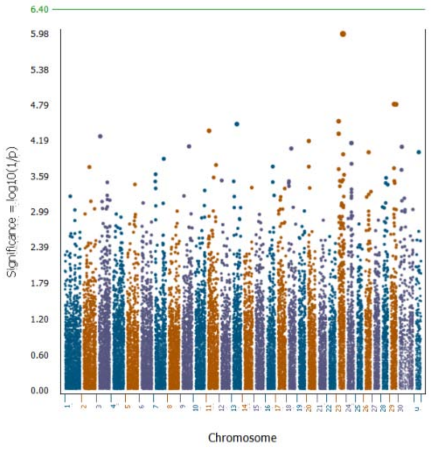

EMMAX_1494

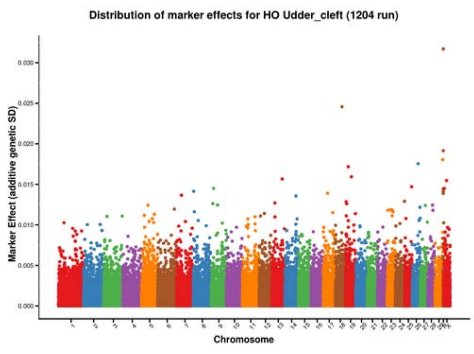

AIPL

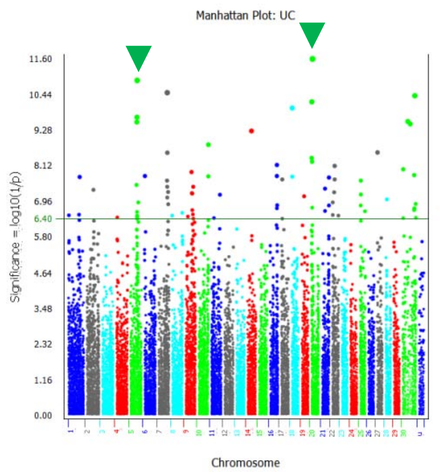

GLS

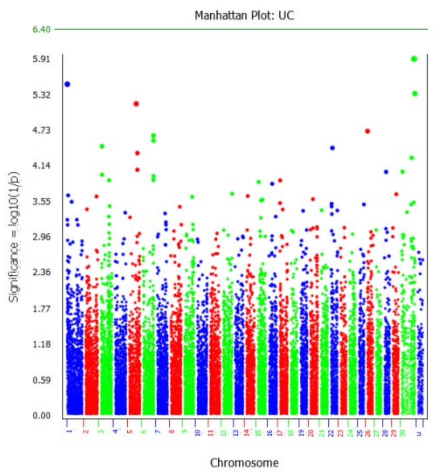

EMMAX-IBS

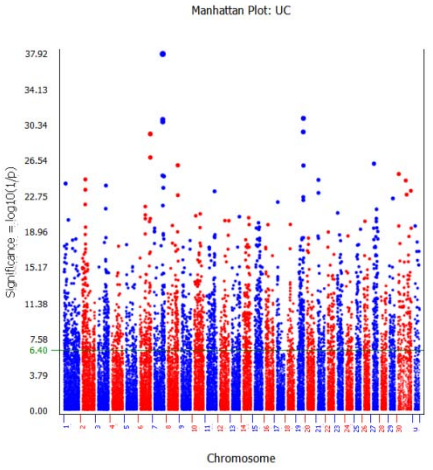

LS

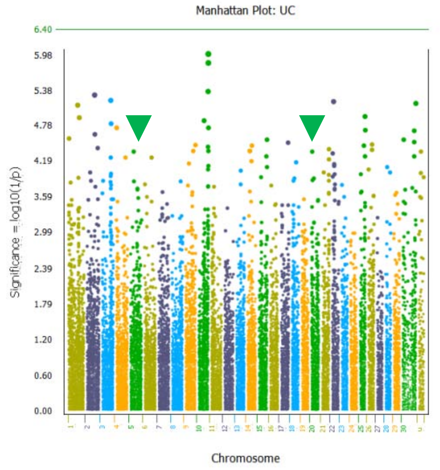

GLS+PCA

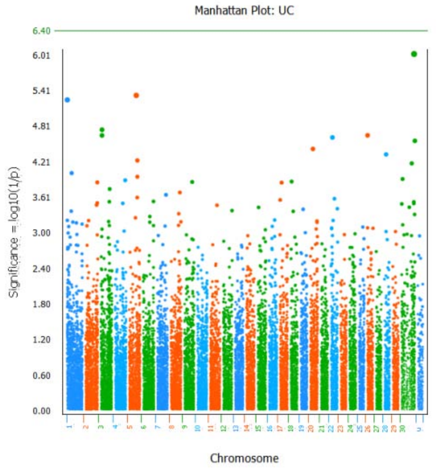

EMMAX+PCA

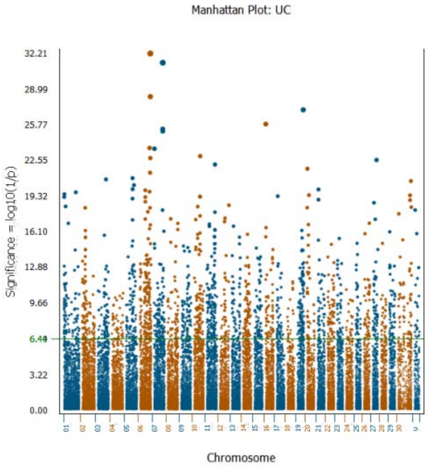

LS_1494

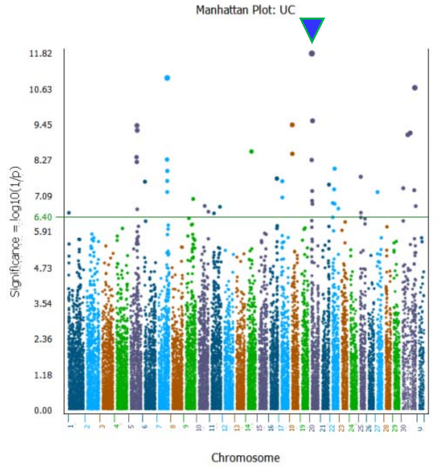

GLS_1494

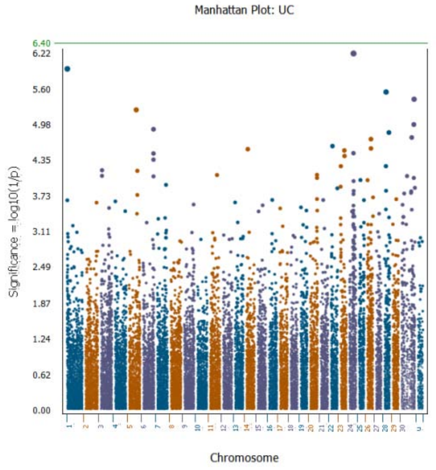

EMMAX_1494

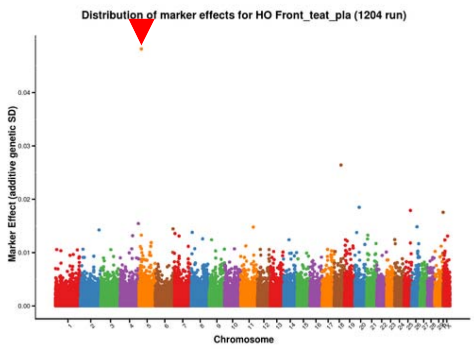

AIPL

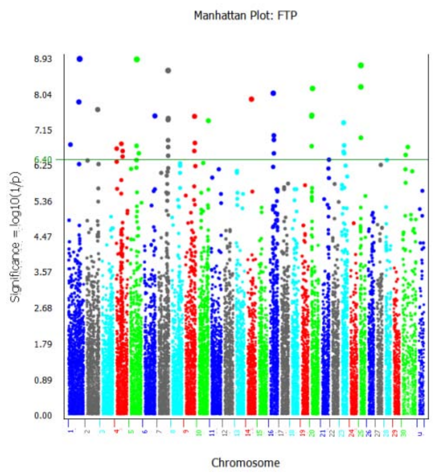

GLS

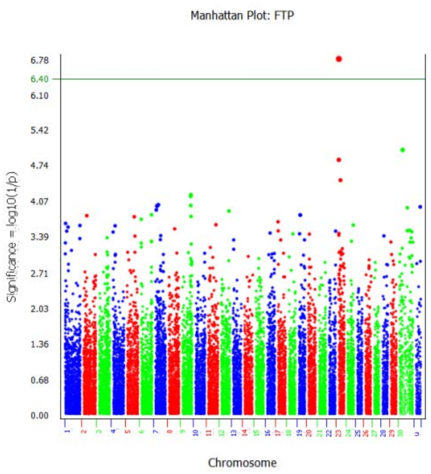

EMMAX-IBS

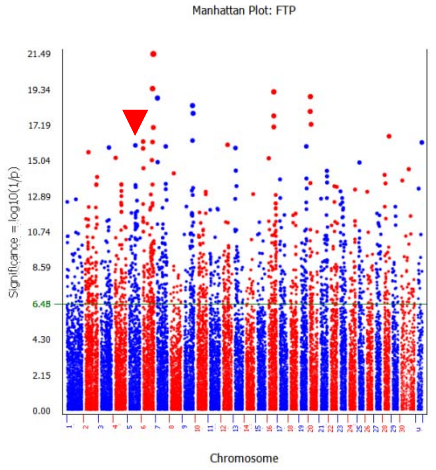

LS

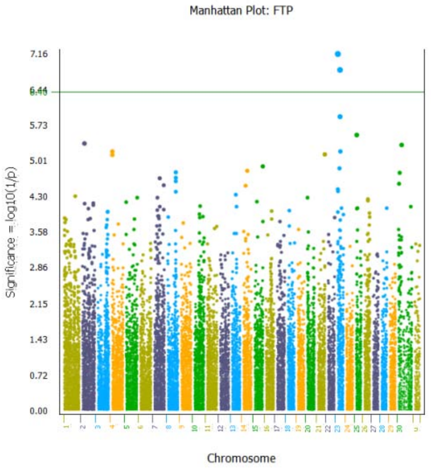

GLS+PCA

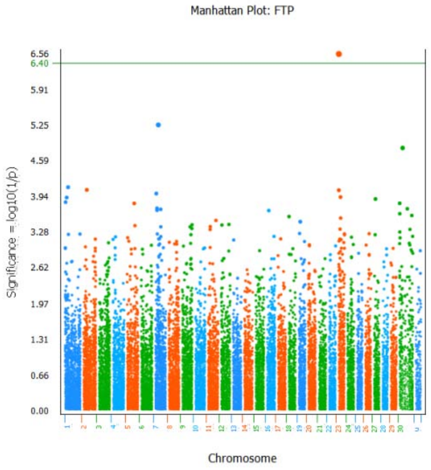

EMMAX+PCA

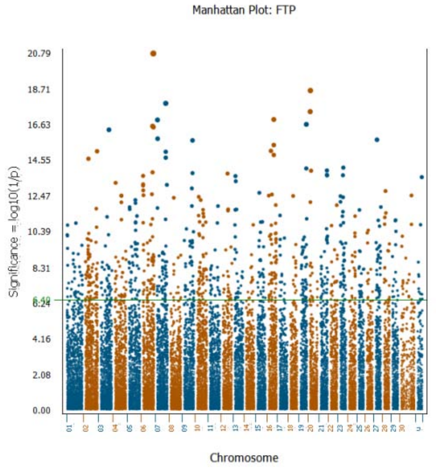

LS_1494

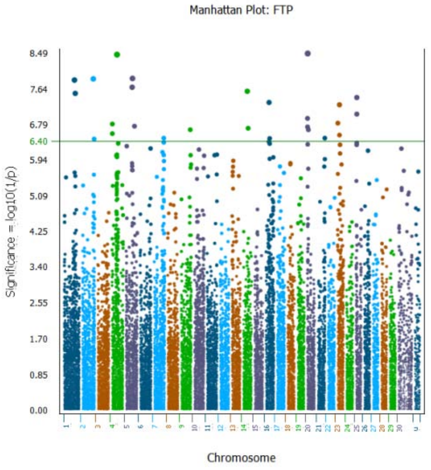

GLS_1494

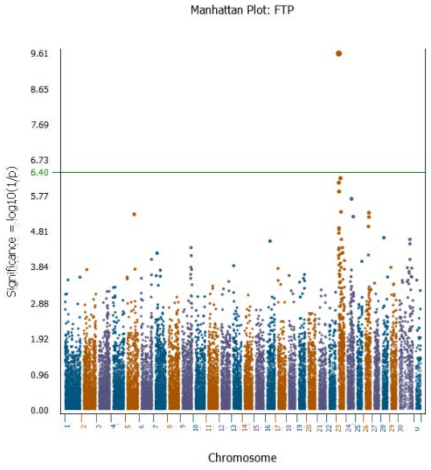

EMMAX_1494

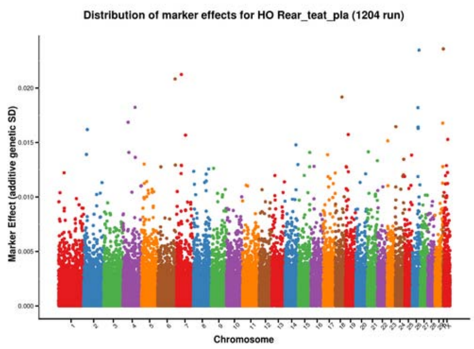

AIPL

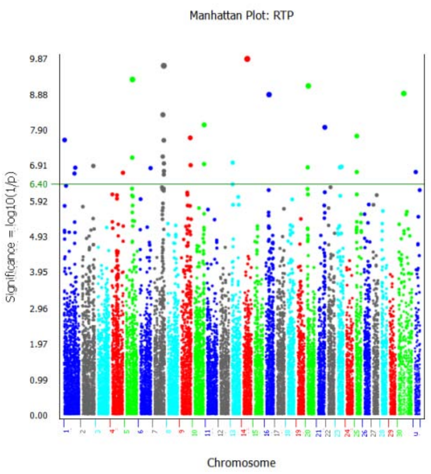

GLS

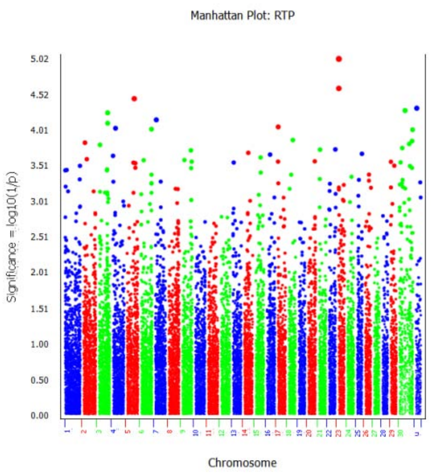

EMMAX-IBS

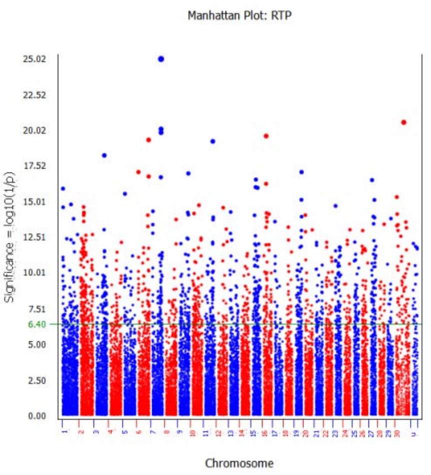

LS

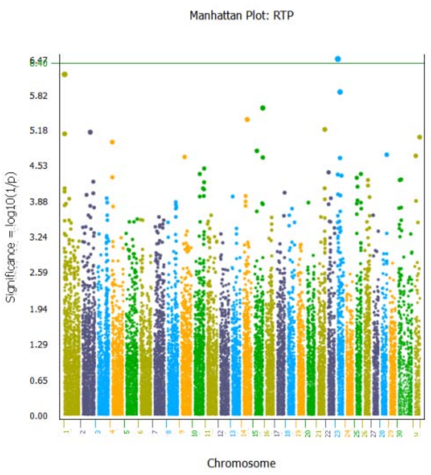

GLS+PCA

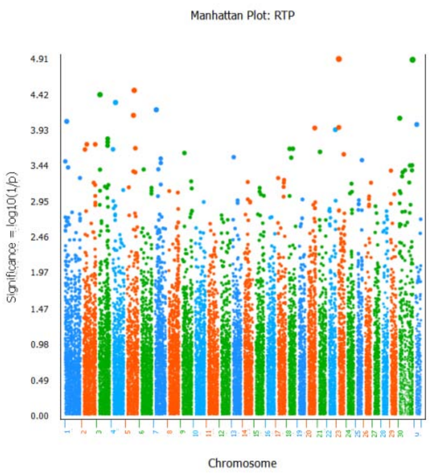

EMMAX+PCA

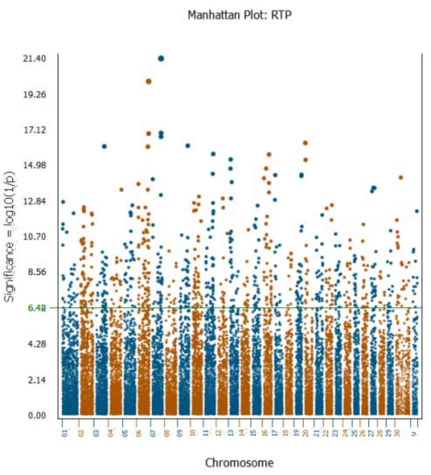

LS_1494

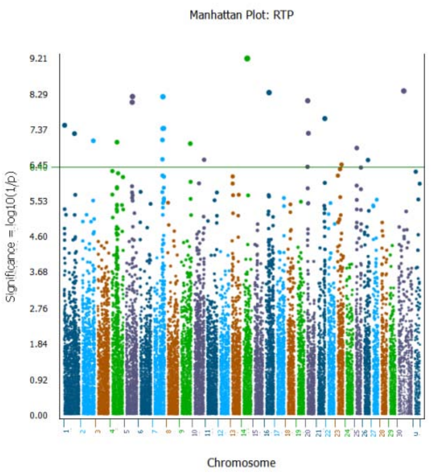

GLS_1494

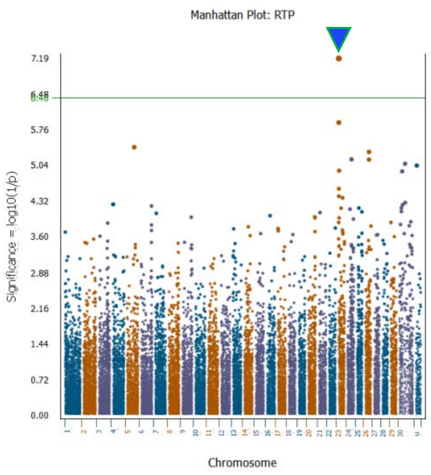

EMMAX_1494

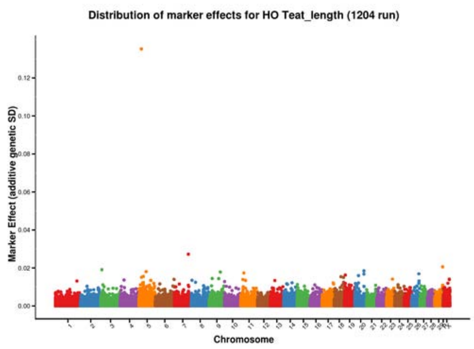

AIPL

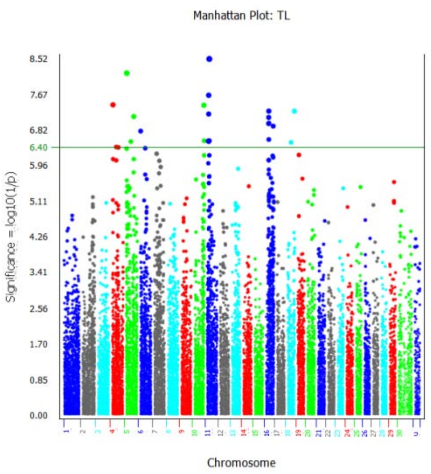

GLS

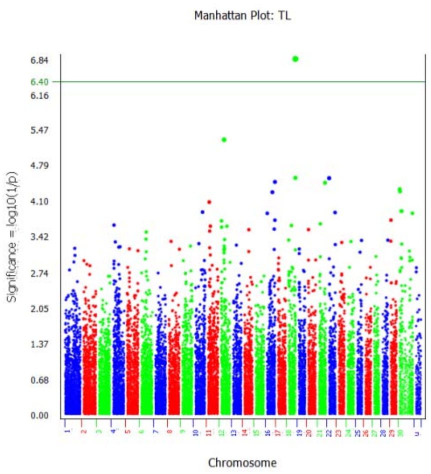

EMMAX-IBS

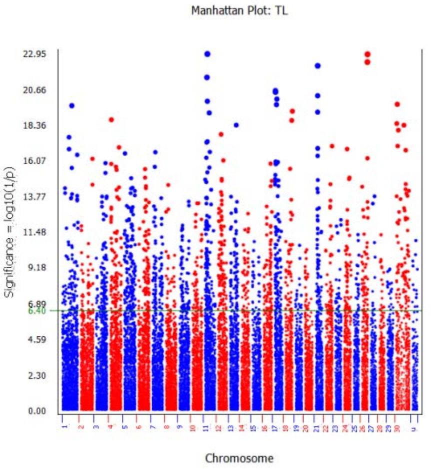

LS

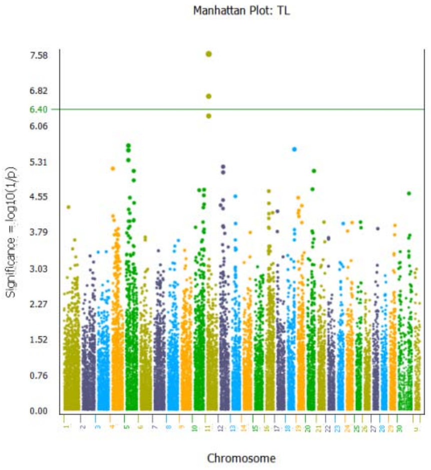

GLS+PCA

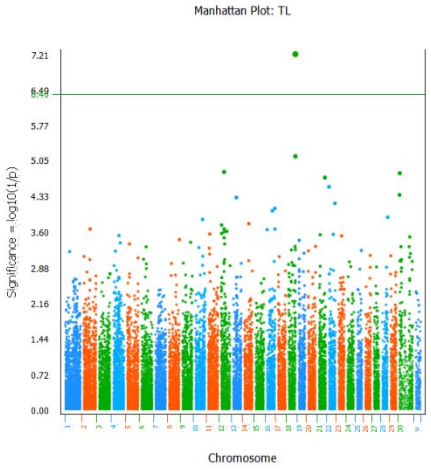

EMMAX+PCA

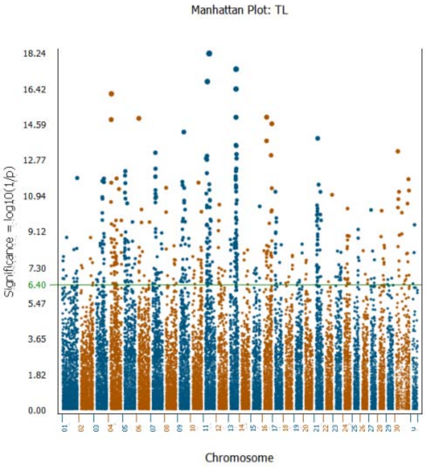

LS_1494

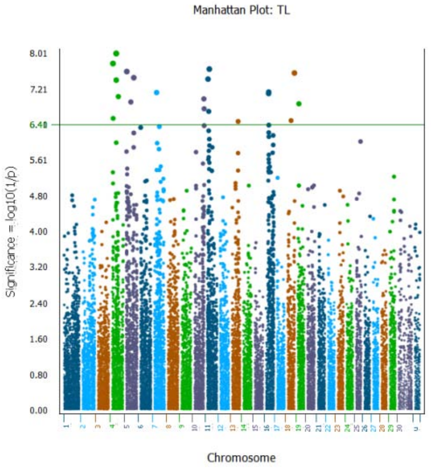

GLS_1494

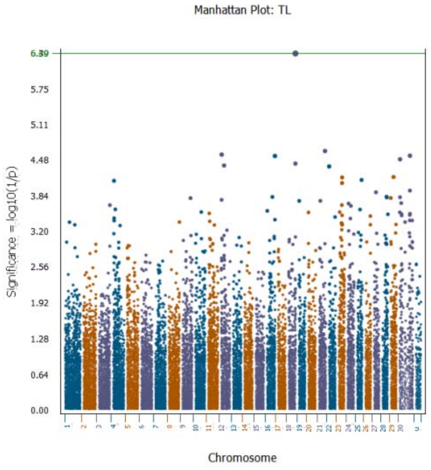

EMMAX_1494

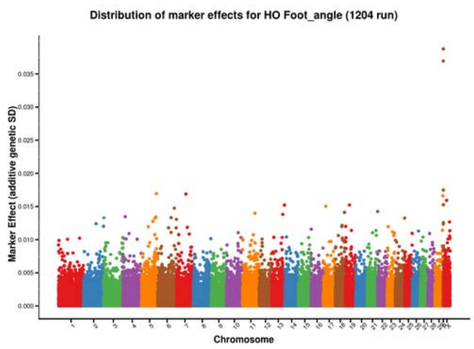

AIPL

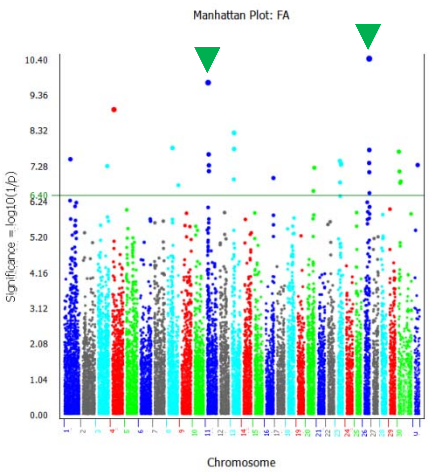

GLS

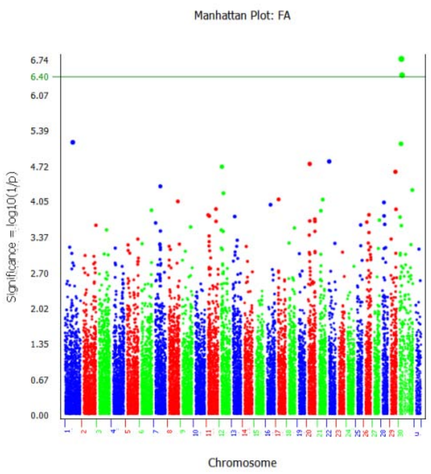

EMMAX-IBS

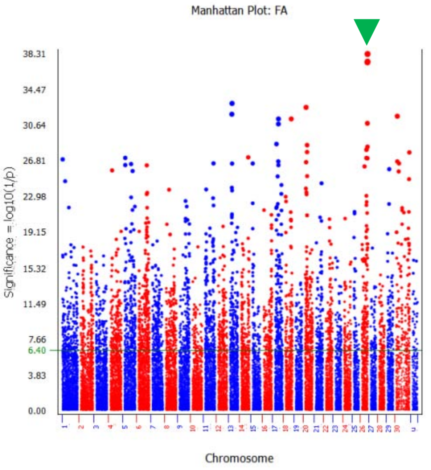

LS

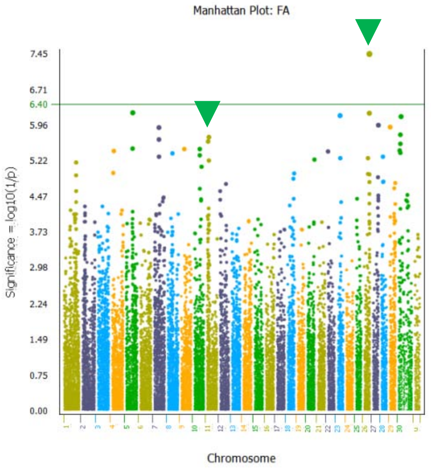

GLS+PCA

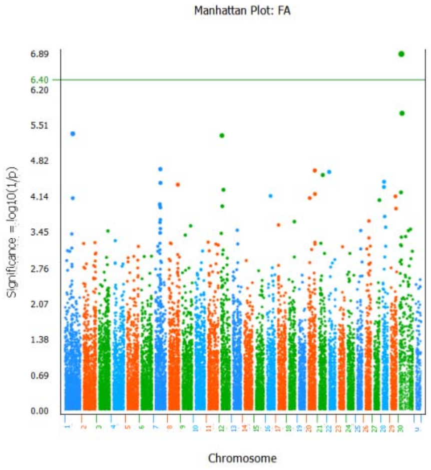

EMMAX+PCA

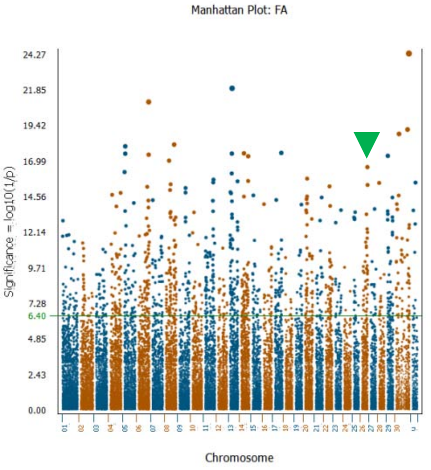

LS_1494

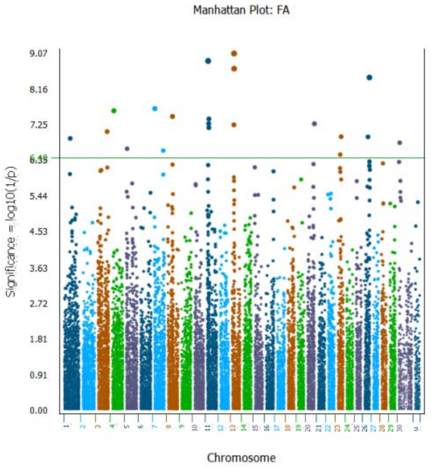

GLS_1494

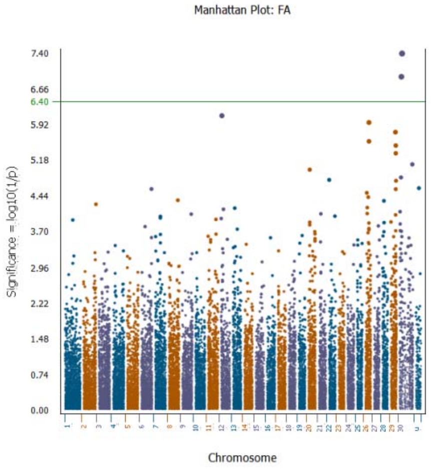

EMMAX_1494

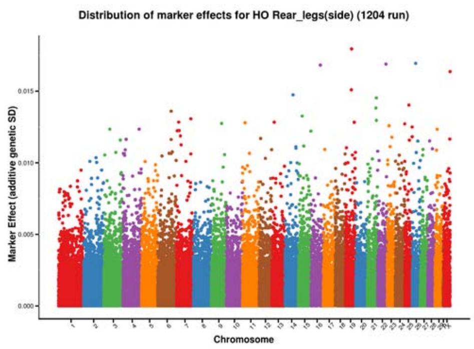

AIPL

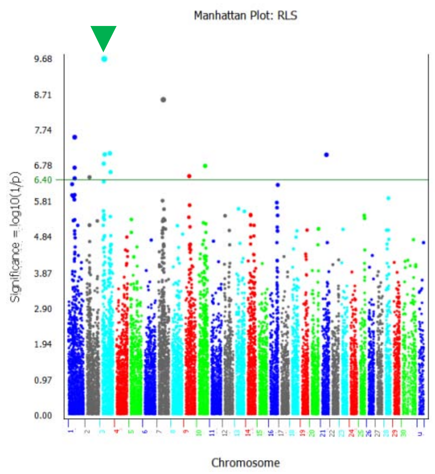

GLS

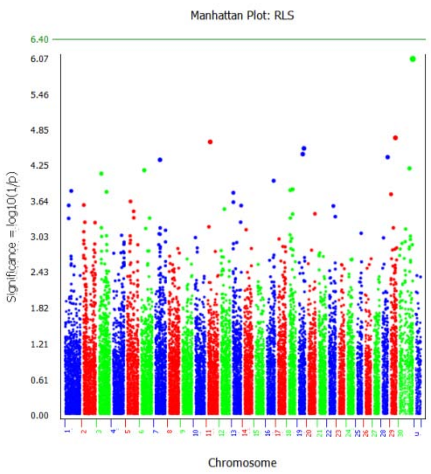

EMMAX-IBS

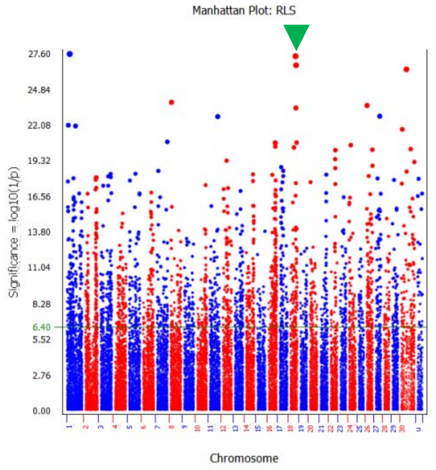

LS

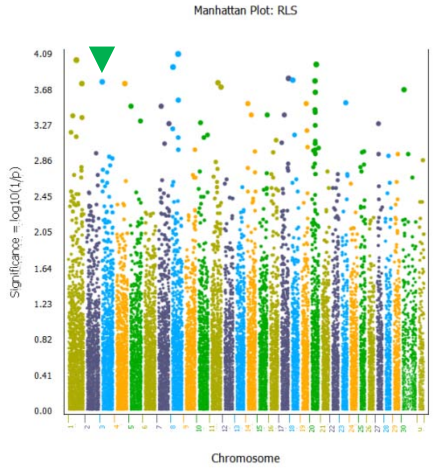

GLS+PCA

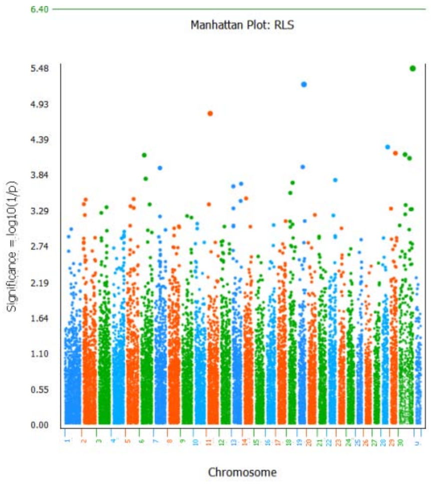

EMMAX+PCA

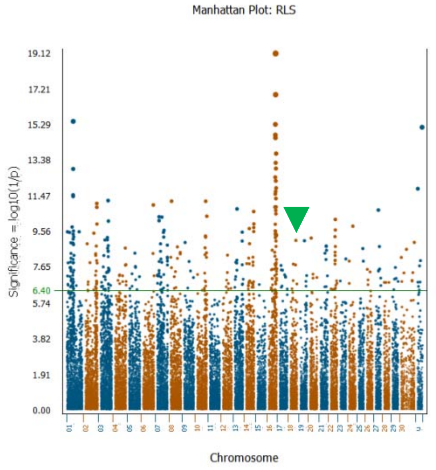

LS_1494

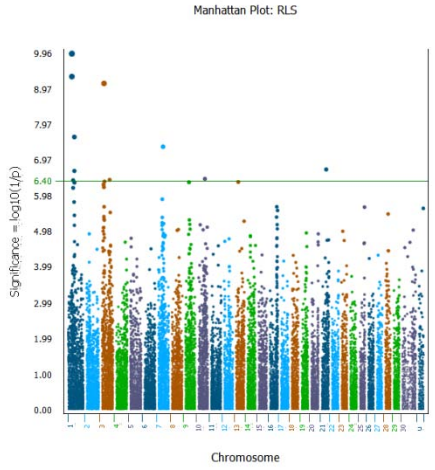

GLS_1494

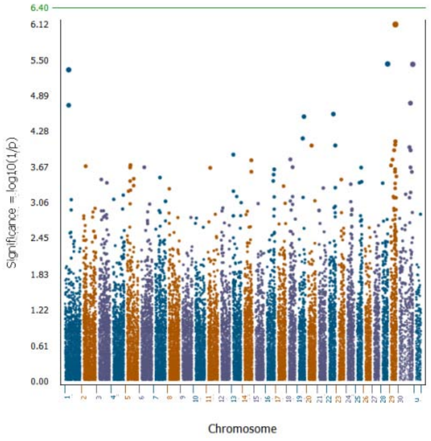

EMMAX_1494

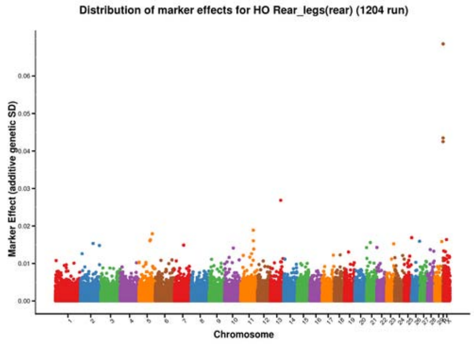

AIPL

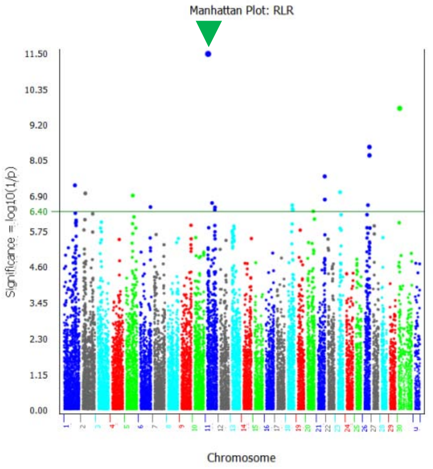

GLS

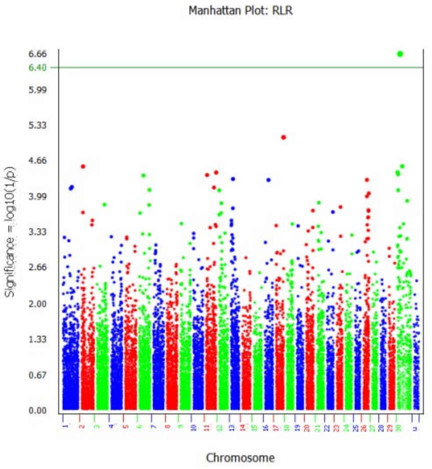

EMMAX-IBS

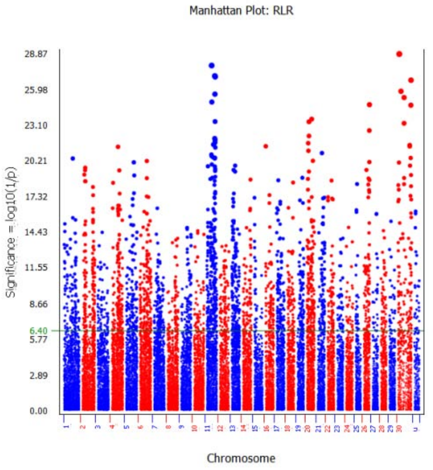

LS

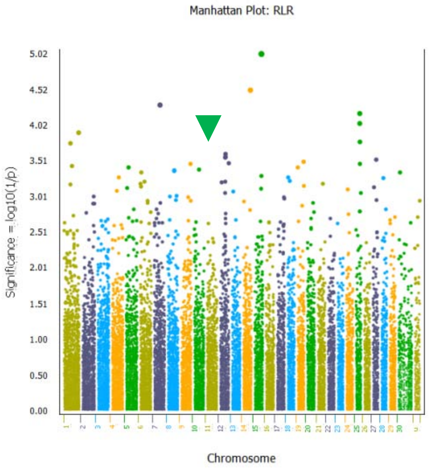

GLS+PCA

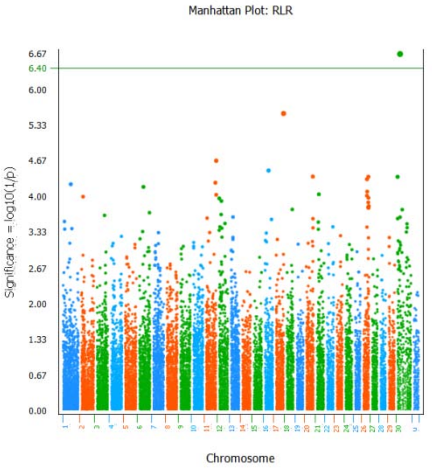

EMMAX+PCA

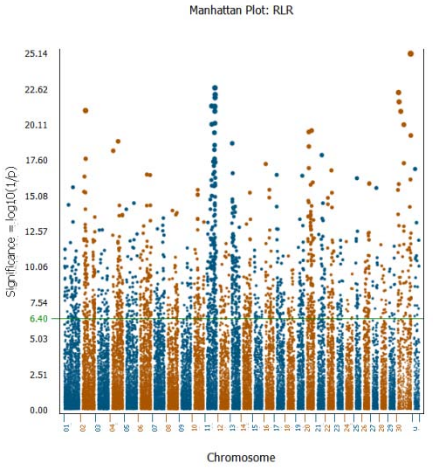

LS_1494

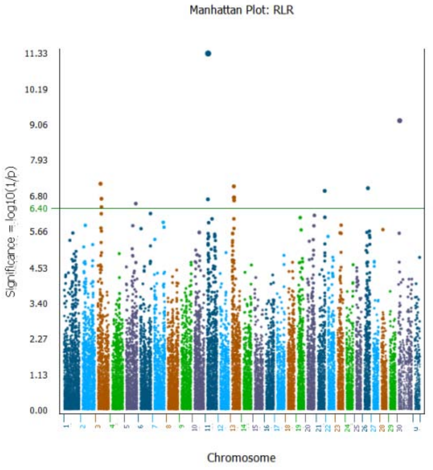

GLS_1494

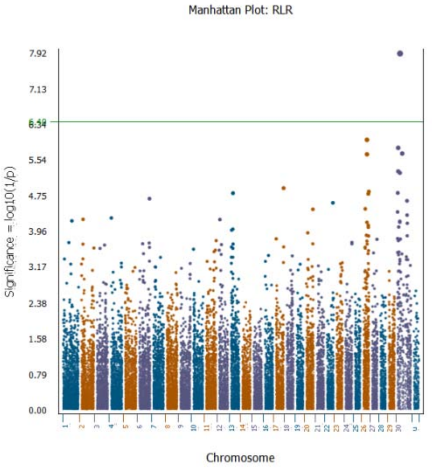

EMMAX_1494

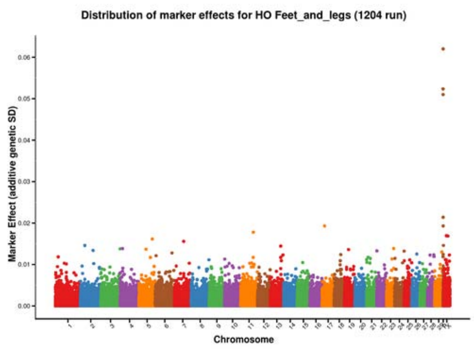

AIPL

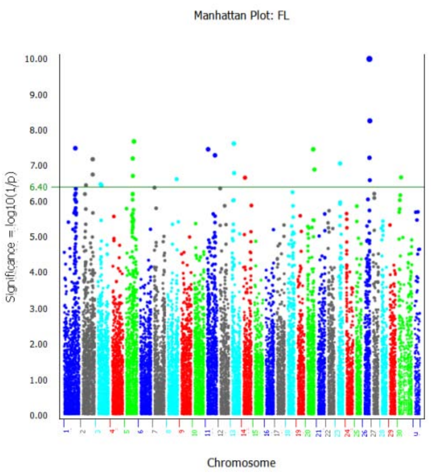

GLS

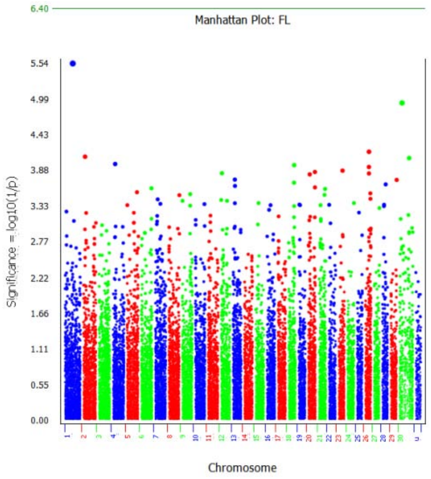

EMMAX-IBS

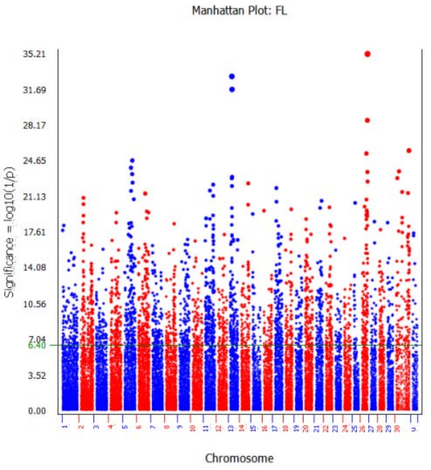

LS

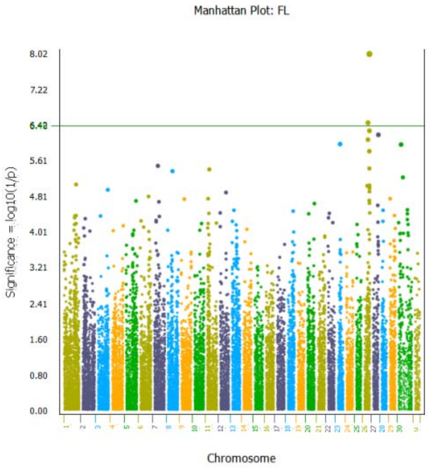

GLS+PCA

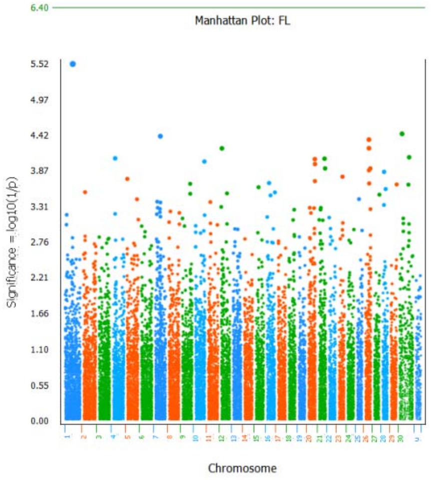

EMMAX+PCA

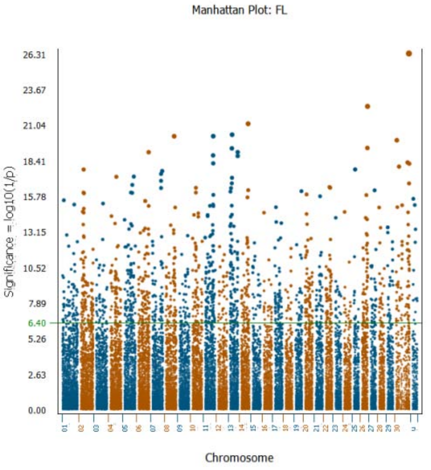

LS_1494

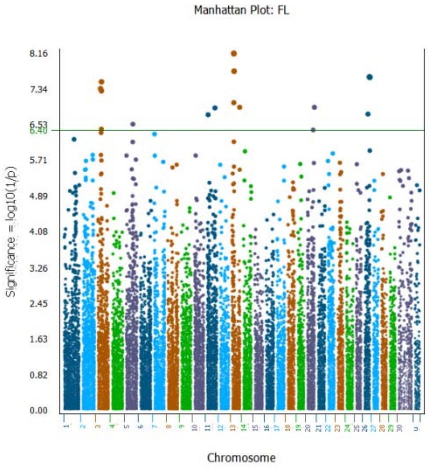

GLS_1494

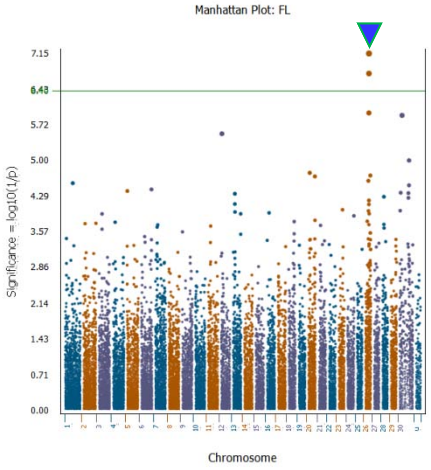

EMMAX_1494

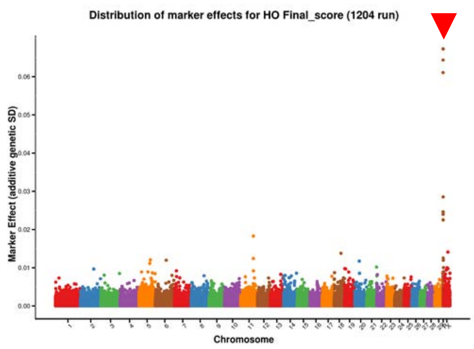

AIPL

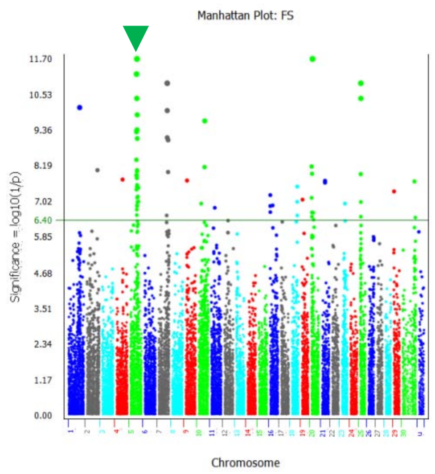

GLS

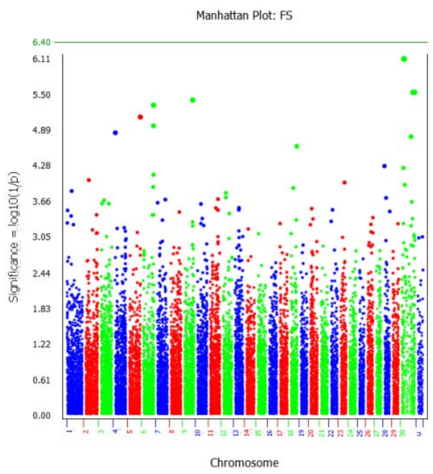

EMMAX-IBS

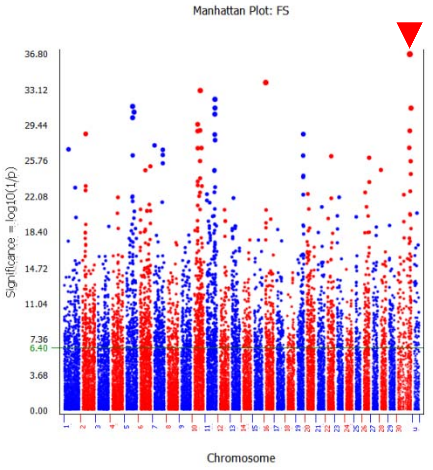

LS

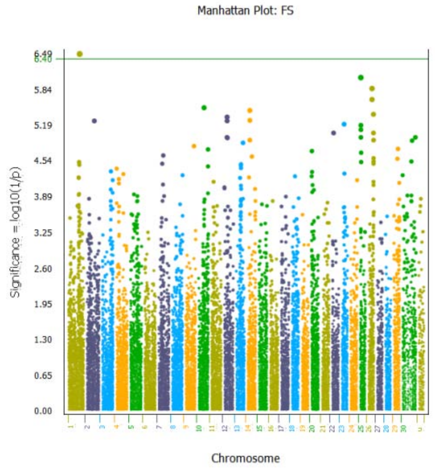

GLS+PCA

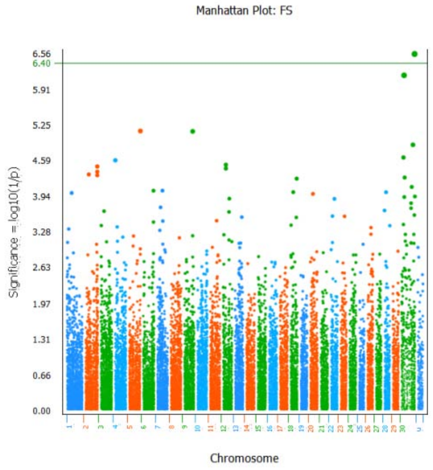

EMMAX+PCA

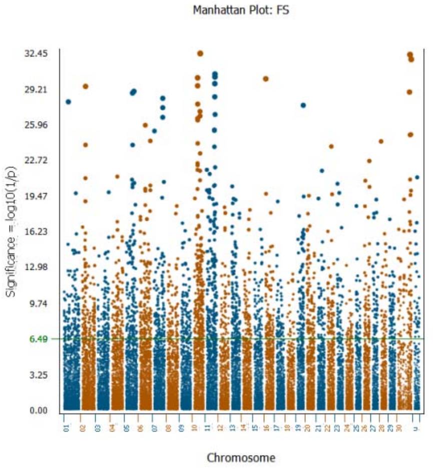

LS_1494

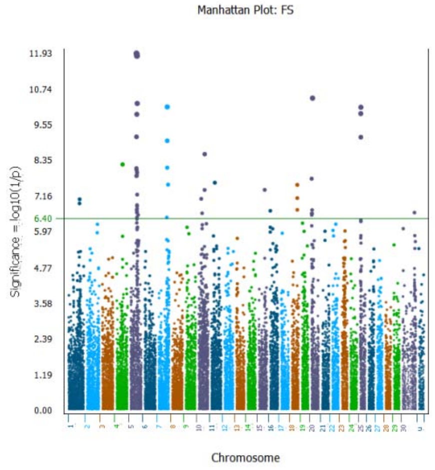

GLS_1494

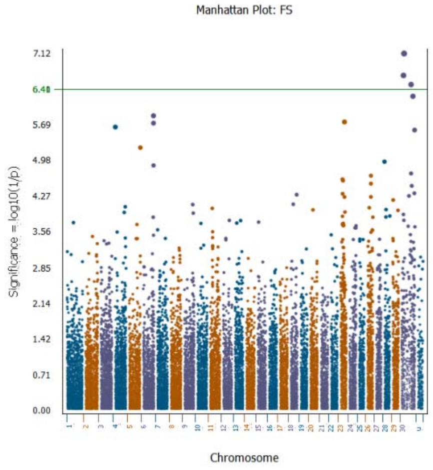

EMMAX_1494
