# Supplementary material for: Effect of sample stratification on dairy GWAS results
Source: BMC Genomics. 2012 Oct 6;13:536. doi: 10.1186/1471-2164-13-536 (PMC3496570; doi:10.1186/1471-2164-13-536)

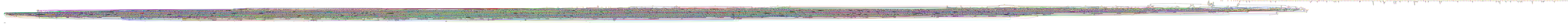

Supplement: Additional file 2 — Figure S2. Pedigree of the 1,654 contemporary cows tracing back to ancestors born in 1930’s (approximately 10–15 generations). Circles in gold color are the 1,654 cows used in the genome-wide association analysis. The pedigree shows that all 1,654 cows are related. [file 1471-2164-13-536-S2.pdf]
